# Whole genome methylation and transcriptome analyses to identify risk for cerebral palsy (CP) in extremely low gestational age neonates (ELGAN)

**DOI:** 10.1038/s41598-021-84214-9

**Published:** 2021-03-05

**Authors:** An N. Massaro, Theo K. Bammler, James W. MacDonald, Krystle M. Perez, Bryan Comstock, Sandra E. Juul

**Affiliations:** 1 Department of Neonatology, Pediatrics - Division of Neonatology, Children’s National Health Systems, The George Washington University School of Medicine, 111 Michigan Avenue, NW, Washington, DC 20010 USA; 2 Department of Environmental and Occupational Health Sciences, University of Washington, Seattle, WA USA; 3 Pediatrics - Division of Neonatology, University of Washington, Seattle, WA USA; 4 Department of Biostatistics, University of Washington, Seattle, WA USA

**Keywords:** Biomarkers, Diseases, Medical research, Neurology, Pathogenesis

## Abstract

Preterm birth remains the leading identifiable risk factor for cerebral palsy (CP), a devastating form of motor impairment due to developmental brain injury occurring around the time of birth. We performed genome wide methylation and whole transcriptome analyses to elucidate the early pathogenesis of CP in extremely low gestational age neonates (ELGANs). We evaluated peripheral blood cell specimens collected during a randomized trial of erythropoietin for neuroprotection in the ELGAN (PENUT Trial, NCT# 01378273). DNA methylation data were generated from 94 PENUT subjects (n = 47 CP vs. n = 47 Control) on day 1 and 14 of life. Gene expression data were generated from a subset of 56 subjects. Only one differentially methylated region was identified for the day 1 to 14 change between CP versus no CP, without evidence for differential gene expression of the associated gene RNA Pseudouridine Synthase Domain Containing 2. iPathwayGuide meta-analyses identified a relevant upregulation of JAK1 expression in the setting of decreased methylation that was observed in control subjects but not CP subjects. Evaluation of whole transcriptome data identified several top pathways of potential clinical relevance including thermogenesis, ferroptossis, ribosomal activity and other neurodegenerative conditions that differentiated CP from controls.

## Introduction

Despite advances in neonatal intensive care, survivors of preterm birth continue to suffer high rates of long term intellectual and/or physical impairment. Babies born prior to 28 weeks gestational age (Extremely Low Gestational Age Neonates—ELGANs) are at particularly high risk, with severe neurodevelopmental impairment reported in almost half of survivors and cerebral palsy (CP) in up to 10%^1–3^. Preterm birth remains the leading identifiable risk factor for CP, estimated to account for more than 50% of cases from population-based studies^4^. While promising neuroprotective therapies for ELGANs are under investigation, advancing neuroprotective care in the neonatal intensive care unit (NICU) is limited by the absence of biomarkers of brain injury that can identify infants *early* in the course of injury progression and help elucidate *specific* causal pathways to injury.

Prematurity-related neurologic injury is multifactorial and has been associated with various perinatal stressors including inflammation, intermittent hypoxia/hyperoxia, ischemia, pain, and nutritional deficiencies^5^. Any and all of these environmental triggers can lead to differential gene expression leading to risk for adverse outcomes. Modification of gene expression can occur by various epigenetic mechanisms, including DNA methylation^6^. Studies in preterm infants have demonstrated multiple differentially methylated regions (DMRs) compared to term controls^7–14^. Differences in DNA methylation, and associated differences in gene expression, may contribute to the risk for neurological sequelae in this high risk population.

We evaluated biological samples collected as a part of an NIH-funded Phase III Randomized Controlled Trial assessing the efficacy of erythropoietin (Epo) for neuroprotection in preterm infants (PENUT Trial, NCT01378273)^15,16^. Neonatal peripheral blood cell (PBC) samples from selected PENUT subjects were analyzed to determine whether examination of DMR and gene expression profiles in ELGAN survivors will lead to early neonatal biomarkers of CP. We hypothesized that early stressors of extrauterine life result in DMR and/or differentially expressed gene signatures that can distinguish infants with and without CP.

## Results

We identified total of 76 CP cases after completion of PENUT follow-up. High quality DNA was isolated from 94 PENUT subjects (n = 47 CP vs. n = 47 Control) at both timepoints of interest. The CP cohort consisted of babies with mild (n = 24), moderate (n = 16) and severe (n = 7) levels of motor impairment. Characteristics of the study population are summarized in Table 1. The majority of patients with CP had diplegia (n = 33, 70%), followed by spastic quadriplegia (n = 10, 21%) and hemiplegia (n = 4, 9%). While most CP subjects had a Gross Motor Function Classification Scale (GMFCS) level of 0 (n = 19, 40%), various levels of functional impairment were represented in the cohort (Table 1). Gene expression data were generated from a subset of subjects with adequate RNA (RIN > 5); 56 subjects (n = 29 CP vs n = 27 Control) on day 1 and 23 subjects (n = 12 CP vs n = 11 Control) on day 14.

**Table 1:** Characteristics of the Study Population.

	Cerebral Palsy (n = 47)	No Cerebral Palsy (n = 47)	P Value
Gestational Age (mean ± SD weeks)	25.88 ± 1.29	25.93 ± 1.26	0.853
Birthweight (mean ± SD grams)	753.09 ± 190.56	837.91 ± 190.09	0.033
Sex (n, %male)	23 (49)	23 (49)	1.000
Severe bronchopulmonary dysplasia	19 (42)	22 (47)	0.680
Intraventricular hemorrhage, Grade 3 or 4	12 (25)	1 (2)	0.002
Severe retinopathy of prematurity (requiring intervention)	9 (19)	2 (4)	0.050
Necrotizing enterocolitis, Stage 2b or 3	2 (4)	3 (6)	1.000
Sepsis	4 (8)	2 (4)	0.677
**GMFCS level**			< 0.001
0	19 (40)	45 (96)	
0.5	8 (17)	1 (2)	
1	10 (21)	1 (2)	
2	7 (15)	0 (0)	
4	3 (7))	0 (0)	

Data presented as n(%) unless otherwise specified.

### DNA methylation analyses

**Figure 1:**
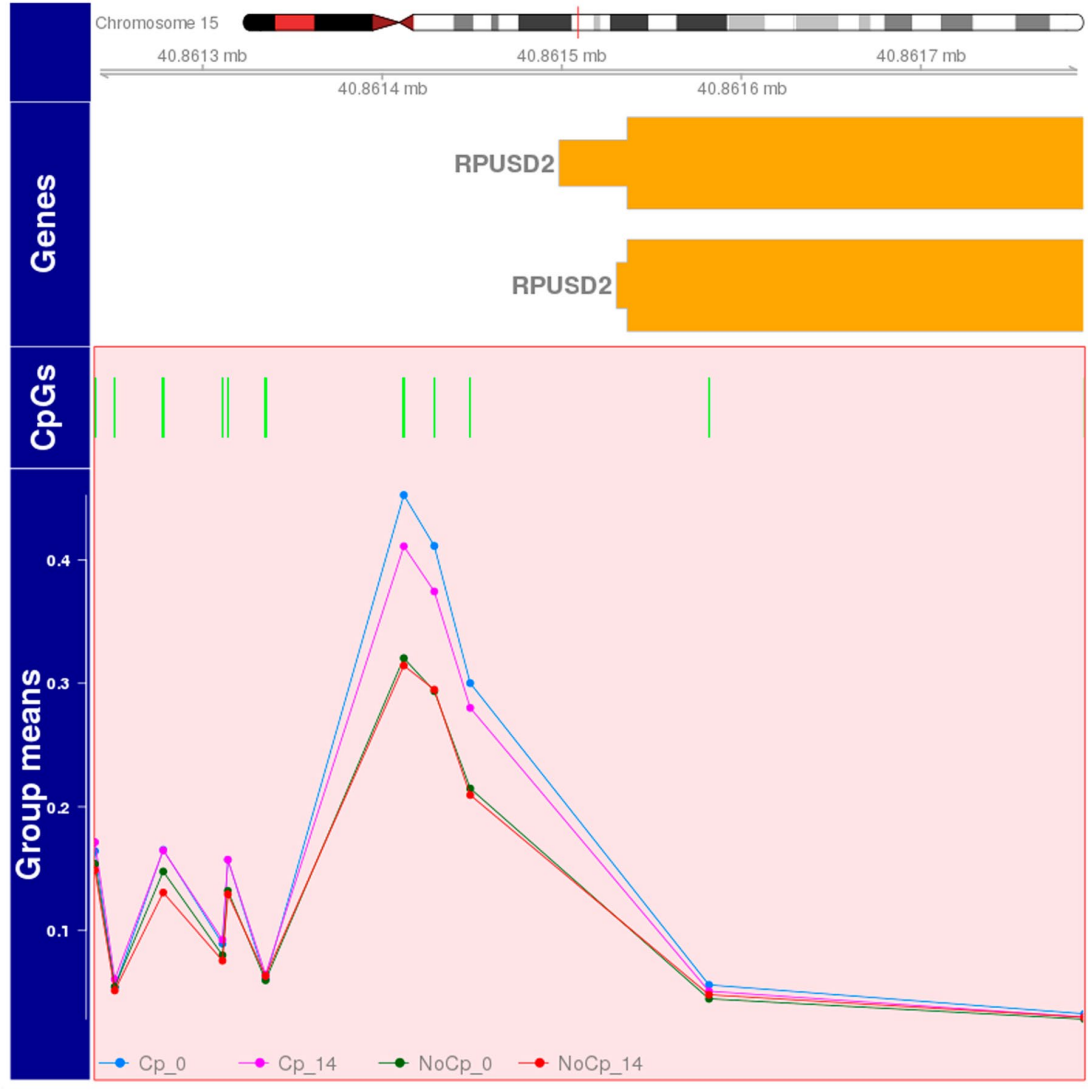
Significant DMR on chromosome 15 for the interaction (day 1 to 14 change) in CP vs Control. Methylation decreases in CP babies on day 14 (pink) compared to day 1 (blue), whereas appears stable in Control babies (red and green). Individual CpG numbers (denoted by green vertical bars) are listed as follows: cg09167084; cg18988510; cg12754238; cg20139049; cg17356999; cg15454857; cg21874278; cg14760714; cg12307764; cg18013830; cg00066468. Figure generated with the Bioconductor Gviz package (version 1.28.3).

Only 22 CpGs for the CP versus control day 1 comparison and 5 CpGs on day 14 were identified spread across the genome, so there was no evidence for any DMRs that differentiated CP cases from controls. In testing the day 1 and 14 interaction, we identified only one DMR on chromosome 15 (chr15: 40861240–40861791; mean proportional change 0.015, p = 8.62 e−0.01) that included 11 CpGs and showed decreased methylation between day 1 and 14 in the CP cases, whereas stable methylation was observed over time in controls (Fig. 1). The single gene associated with this DMR, RNA Pseudouridine Synthase Domain Containing 2 (RPUSD2), is a protein coding gene without reported functional correlates to neurological disease. No additional DMRs were identified when the analyses were restricted to the cases with moderate or severe CP. Additionally, there was no evidence for differential gene expression for RPUSD2 in the subset of patients with available transcriptome data (log2 fold change −0.016; p = 0.994). For the between day comparisons, there were multiple DMRs identified within the CP (n = 302, Supplemental Table S1) and control groups (n = 339, Supplemental Table S2).

### Whole transcriptome analyses

**Figure 2:**
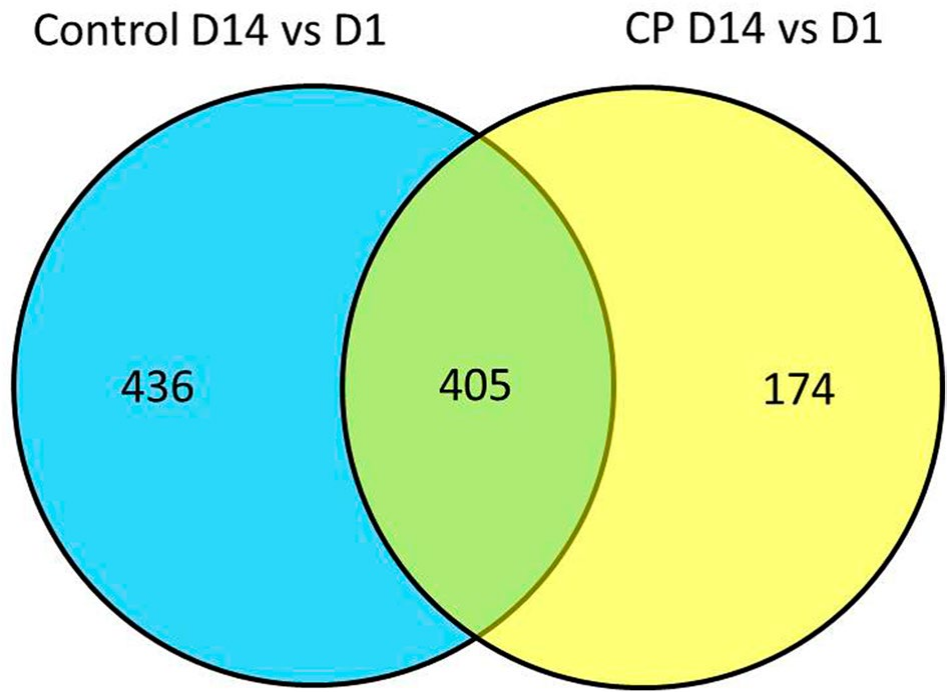
Venn diagram of differentially expressed genes between day 1 and 14 that are unique in CP (yellow) compared to control (blue) ELGANs. Figure generated using the CRAN package gplots (version 3.0.1.1).

There was no evidence for differentially expressed genes between CP and control infants at day 1, day 14 or the interaction (day 1 to 14 change). For the between day comparisons, numerous genes that were significantly upregulated or downregulated over time were identified in both CP (n = 579, Supplemental Table S3) and control infants (n = 841, Supplemental Table S4). In order to evaluate genes with differential expression between day 1 and 14 that were unique to CP versus control, we created a Venn diagram to demonstrate the overlap in significant genes between the two conditions (Fig. 2). iPathwayGuide analysis of differentially expressed genes between day 1 and 14 that were unique to each diagnosis identified several top pathways of potential clinical relevance (Table 2).

**Table 2:** Top pathways based on differentially expressed genes between day 1 and 14 in CP vs Control cases.

	Pathway Name	p-value	FDR	Perturbed Genes	Notes
Unique to CP	Huntington Disease	4.593e–5	0.008	Upregulated: VDAC2, COX5A, POLR2E Downregulated:GPX1, NDUFB3, NDUFA13, UQCR10, TGM2, COX7C	Interference with BDNF transcription/transport, destabilization of neuronal mitochondria
	Thermogenesis	2.889e–4	0.026	Upregulated:COX5A, ADCY7 Downregulated: GNAS, NDUFB3, NDUFA13, UQCR10, DPF3, COX7C, ADRB3, ADCY1-6, ADCY8-10	Process triggered by hypothermia whereby chemical energy is converted to heat in adipose tissue to ensure normal cellular processes
	Parkinson Disease	5.085e–4	0.030	Upregulated:CYCS, VDAC2, CASP9, CASP3, COX5A Downregulated:NDUFB3, NDUFA13, UQCR10, COX7C	Proteasome dysfunction, mitochondrial impairment and oxidative stress leading to loss of dopaminergic neurons
	Ferroptosis	7.290e–4	0.032	Upregulated: VDAC2, CYBB Downregulated: SLC11A2, GCLC	Regulated cell death characterized by production of reactive oxygen species from iron and lipid peroxidation; involved in neurodegenerative disease
Unique to control	Ribosome	2.035e–8	5.2e–6	Upregulated (in Control):RPL7A, RPL11, RPL19, RPL23A, RPL30, RPL37, RPL38, RPS5, RPS8, RPS11, RPS15A, RPS20, RPS29, MRPL20 Downregulated in Control): UBA52, MRPL1	Developmental increase in ribosomal biogenesis observed in controls but not CP subjects is consistent with prior reports of impaired ribosomal activity in CP

**Figure 3:**
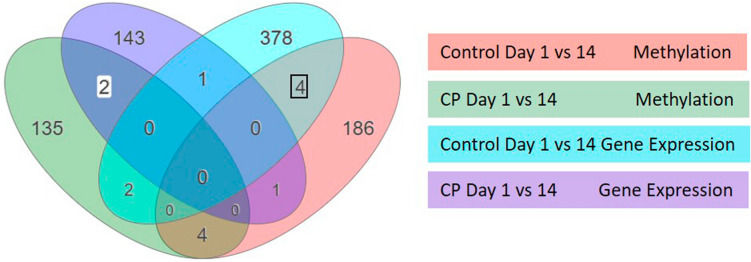
iPathwayGuide meta-analysis of DNA methylation and gene expression data identified 2 genes (highlighted in white) unique to CP, and 4 genes (black box) unique to Controls, that demonstrated evidence of differential methylation and gene expression. Figure generated using Advaita Bio’s iPathway Guide (https://www.advaitabio.com/ipathwayguide).

### Meta-analyses of DNA methylation and transcriptome analyses

Secondary iPathwayGuide meta-analyses were performed to identify genes and/or pathways that were unique to CP subjects or controls across both methylation and gene expression datasets. We extracted the DMRs that were significant for the day 1 to 14 comparisons that were unique to each diagnosis and output the set of associated genes for each DMR. We generated a Venn diagram to evaluate the overlap between genes associated with a DMR and genes that demonstrated differential expression (Fig. 3). We identified only 2 genes, and no relevant pathways, that were differentially expressed between day 1 and 14 only in CP cases, and 4 genes only in Controls, observed consistently across both DNA methylation and gene expression datasets (Fig. 3). These genes and functional information are summarized in Table 3.

**Table 3:** Gene information from iPathwayGuide meta-analysis.

	Gene symbol	Name	Methylation Log Fold Change; P-value	Gene expression Log Fold Change; P-value	Functional information
Unique to CP Cases	CSRP1	Cysteine and Glycine Rich Protein 1	− 0.209; p = 1.000e−6	+ 0.270; p = 0.015	Gene regulation, cell growth and somatic differentiation; promotes skeletal muscle growth
	USP44	Ubiquitin Specific Peptidase 44	+ 0.015; p = 1.000e–6	+ 0.261; p = 0.036	Deubiquitinating enzyme; regulates spindle assembly checkpoint by preventing anaphase onset; regulates cell cycle progression and genomic stability
Unique to Controls	CRELB2	cAMP responsive element binding protein like 2	− 0.003; P = 2.460e−4	− 0.302; P = 0.010	Protein with DNA binding capabilities; may act as tumor suppressor gene
	DCAF11	DDB1 and CUL4 associated factor 11	+ 0.006; P = 2.900e−5	− 0.469; P = 9.326e−4	Mediates degradation of stem-loop binding protein, regulates cell cycle/ viability
	FEM1A	Fem-1 homolog A	+ 0.007; P = 2.170e−4	− 0.504; P = 2.404e−4	Mediates degradation of stem-loop binding protein, regulates cell cycle/ viability
	JAK1	Janus kinase 1	− 6.115e−4; P = 1.370e−4	+ 0.235; P = 0.044	Protein-tyrosine kinase; key role in interferon-alpha, beta and gamma signal transduction

## Discussion

DNA methylation is known to play an important role in brain development^17–21^, with alterations described in infants born preterm and those with postnatal stress^14,22^. While epigenetic changes are tissue-specific and it is recognized that between-tissue variation in DNA methylation exceeds between-individual differences, some inter-individual variation is reflected across brain and blood indicating that peripheral tissues may have some utility in identifying biomarkers of disease phenotypes that manifest in the brain^23–25^. Given this biological context and limited plausibility for identifying PBC DMRs as epigenetic biomarkers of prematurity-related developmental brain injury, it is not surprising that our genome wide DNA methylation analyses demonstrated limited evidence for early epigenetic factors in this tissue type that relate to later CP in ELGANs. Meanwhile, whole transcriptome analyses identified a profile of genes that are uniquely differentially expressed between day 1 and 14 in patients who later developed CP. Further investigation of these pathways may elucidate important insights into the early pathogenesis of CP.

### DNA methylation and cerebral palsy

We identified one DMR in the interaction (day 1 to 14 change) comparison between CP and controls, without associated evidence of differential gene expression. In our secondary analyses, we identified 2 DMR-associated differentially expressed genes in CP cases (CSRP1, USP44) and 4 in Controls (CRELB2, DCAF11, FEM1A, JAK1). Apart from JAK1, these genes are widely expressed and generally involved in cell cycle, growth or differentiation without direct correlates to neurological disease. JAK1, however, is involved in regulating cytokine signaling, mediating innate immune response, and has been linked to the growth, differentiation and aging process of nerve cells. The JAK/STAT pathway has been implicated in the pathogenesis of CP and may serve as a mediator of Epo neuroprotection^26^. The role of methylation in regulating JAK1 expression may warrant further investigation.

Prior studies have suggested epigenetic mechanisms for CP pathogenesis in preterm infants. Two studies reported evidence for differential methylation in young children and adolescents with CP born preterm compared to term born controls^27,28^. These studies utilized sequencing data and evaluated individual CpG probes and/or evaluated differences among each gene, which may suffer from noise compared to DMRs which pool information across genomically adjacent probes in order to boost true signal^29–31^. These two studies also used fewer subjects (four pairs of discordant twins, and 22 CP and 21 controls, respectively). In addition to the methodological differences, our results may differ from these studies given our evaluation of methylation differences in the first 2 weeks of life. Thus, we cannot exclude the possibility of epigenetic changes that occur outside of the newborn period that can lead to CP risk in infants born preterm.

Two recent studies analyzed archived blood spots to evaluate neonatal DNA methylation differences between CP cases and controls. Bahado-Singh and colleagues reported a case–control study (n = 23 CP and n = 21 controls) in which they identified 230 differentially methylated CpG probes that were linked to canonical pathways involved in neuronal function and were found to be overall predictive of CP^32^. This study utilized a 450 K array which investigates half the CpGs as the EPIC array used in our study, requiring a less stringent correction for multiplicity. Their analyses, however, were based on comparisons using beta values (% methylation estimates) rather than the more customary M-values (used in the current study), which satisfy the assumptions of normality for statistical testing. While recent reports suggest that M-value preprocessing may not impact results in large scale datasets^33^, other reports suggest that M-value methods may allow more reliable identification of true positives, particularly in studies with smaller sample sizes^34,35^. More consistent with our results, Mohandas and colleagues reported a study evaluating neonatal blood spots from 15 CP-discordant monozygotic twin pairs (12 born preterm) and described 33 CpG probes and 2 DMRs^36^, although these did not meet significance threshold after adjustment for multiplicity. To our knowledge, this report represents the largest study to date evaluating DNA methylation in neonatal blood samples from preterm infants and the risk for later CP. Our data suggest that epigenetic changes reflected in PBCs in the first 2 weeks of life have a limited role in the pathogenesis of CP.

### Transcriptome profiles and cerebral palsy

Although we did not identify significant differentially expressed genes in our direct comparisons between CP and control groups, evaluation of genes with differential expression from day 1 to 14 that were unique to each group provided insights into pathways with potential relevance to neurological disease. Two of the top canonical pathways identified included Parkinson’s and Huntington’s disease, both neurodegenerative conditions affecting the basal ganglia with associated motor dysfunction. The link between thermogenesis and CP is less clear, although this process is regulated by dopaminergic signaling in the CNS^37^. Of interest is the identification of ferroptosis, which has recently emerged as an important oxidative stress-induced cell death pathway and has been implicated in the pathogenesis of neurodegenerative diseases such as Alzheimer’s, traumatic brain injury, and stroke^38^. As this pathway is uniquely upregulated in babies with CP, it warrants further investigation. Similarly, the upregulation of ribosomal genes observed only in control infants suggests that this developmental change may be an important mediator of CP risk and that impaired upregulation of genes encoding ribosomal machinery may contribute to the development of CP. This finding is consistent with prior reports that have linked ribosomal activity to CP and other neurological diseases^39–42^. While these results do not provide specific causal pathways to CP, further exploration of relevant pathways may provide novel insights and gene targets for future studies.

Prior studies have evaluated neonatal transcriptome data in mixed cohorts of preterm and term born children with CP. Ho and colleagues evaluated neonatal peripheral blood from 20 preterm-born (< 37 weeks gestational age) children with CP and 1:1 gender and gestational age matched controls^43^. They reported that inflammatory, hypoxic and thyroidal gene sets were upregulated in preterm-born CP cases compared to controls. Of note, RNA was extracted from archived neonatal blood spots and significant degradation was noted (average RIN 2.3 ± 0.7) which may have impacted reproducibility of these findings. Van Eyk and colleagues demonstrated transcriptional dysregulation of trophic signaling pathways in patient-derived immortalized B-cell lines of a large cohort of CP cases (n = 182) and network analyses demonstrated significant overlap with genes observed in autism^44^. Cell lines used in these analyses were derived from multiple separate biorepositories which may have contributed technical variability. While methodological differences may explain variable results across studies, these studies and ours suggests that investigating transcriptome profiles in babies with CP can provide insights into pathogenic mechanisms and potentially identify early therapeutic targets.

### Technical notes and limitations

We evaluated mixed PBC specimens to generate DNA methylation and RNA transcriptome data. It is acknowledged that several factors including age, prematurity^45^ and erythropoietin treatment^46^ may impact cell proportions. We chose to use the surrogate variable analysis approach, which ensures orthogonality of our covariates and has been shown to be a reasonable approach for cell-type mixture adjustment under most scenarios^47^. Another important technical note is to acknowledge that these PBC pellets were not primarily stored for RNA preservation. Thus, we faced significant RNA degradation which limited our sample size and introduced technical variability into our analyses. We limited the impact of this on our analyses by requiring a minimum RIN (> 5) and using robust analytic and QC approaches to increase confidence that differences observed were biological and not due to differential degradation or other technical confounders. Given the stringent significance criteria and multiplicity correction used for our primary analyses, we performed secondary pathway analyses to identify potentially relevant DMRs or differentially expressed genes that were uniquely changing within CP versus controls. These findings should be considered hypothesis generating and warrant further investigation in future studies. Likewise, future studies will need to investigate the impact of important clinical confounders that may impact methylation profiles. While our principle components analyses demonstrated that the selected clinical factors explored did not correlate with methylation changes in our dataset (supplemental figures), our study was not designed to investigate methylation in these co-morbidities. Finally, we included all severity levels and classifications of CP in order to increase sample size and power for our analyses. It is acknowledged that the pathogenesis of the more severe forms or subtypes of CP may differ and future investigations focusing on specific phenotypes may yield different results.

## Conclusion

While the role of methylation in regulating JAK1 expression warrants further investigation, we found limited evidence of distinguishing methylation changes in peripheral blood cells that related to the development of CP in this selected population of ELGANs. Whole transcriptome analyses may implicate ferroptosis and ribosomal activity as potential canonical pathways leading to CP risk in the preterm ELGAN which warrant further investigation.

## Methods

### Study population

This is an ancillary study to the NIH-funded PENUT Trial^15,16^, a randomized, placebo-controlled study of Epo treatment in 941 preterm infants 24-0/7 to 27–6/7 weeks of gestation. Infants were enrolled between December 2013 and September 2016 from 19 U.S. sites. Written informed consent was obtained from the parent of each participant and the study was approved by the institutional review boards at each participating study site. Infants were randomized to Epo treatment or placebo within 24 h of birth, with study drug continued until 32–6/7 weeks corrected age. Infants with major life-threatening or chromosomal anomalies hematopoietic crises such as DIC or hemolysis, polycythemia, congenital infection or prior use of Epo were excluded. Detailed characteristics of the study population have been previously reported^16^. Developmental assessment at 24 ± 2 month corrected age included diagnosis and classification of CP by standardized neurological examination based on ELGAN Neurological Exam Study protocol^48,49^ and the Gross Motor Function Classification System (GMFCS)^50,51^. The GMFCS is a 5-level system to aid in classification of CP on the basis of voluntary gross motor skills, with a level of 5 the most severe. For this study, the subset of infants with any diagnosis of CP by neurological exam were identified and included if they had adequate biological specimens available at both timepoints of interest. Severity of motor impairment was classified as mild, moderate or severe based on CP subtype and GMFCS level as prescribed by the parent study^15,16^. This CP cohort was distributionally matched 1:1 to a cohort of control infants without CP by gestational age (within 3 days), treatment allocation and sex. This study is reported in accordance with the Strengthening the Reporting of Observational Studies in Epidemiology (STROBE) guidelines and all methods were carried out in accordance with relevant guidelines and regulations^52^.

### Specimen processing, DNA and RNA isolation

Serial blood samples were obtained at baseline (day 1), day 7, 9 and 14 to measure circulating inflammatory and brain-specific biomarkers. Samples were spun for 8 min at 2000 G, plasma separated from PBC pellet and each stored at -70 °C. Stored day 1 and 14 PBC specimens from selected PENUT subjects were divided into 2 aliquots for DNA and RNA isolation. DNA isolation was performed using the commercially available QIAamp Blood DNA isolation kit (Qiagen, Inc., Valencia, CA) according to the manufacturer’s protocol. DNA purity was assessed by measuring OD260/280 and OD260/230 ratios. Samples with OD260/280 ratios and OD260/230 ratios ≥ 1.8 were deemed of good quality DNA. RNA isolation was performed using a commercially available RNA isolation kit (Macherey–Nagel's NucleoSpin RNA Blood kit, cat#740200.50; Macherey–Nagel, Bethlehem, PA) using the manufacturer's “RNA isolation from 200 uL blood” protocol. RNA quantities was determined by spectrometry using the NanoDrop 8000 Spectrophotometer (ThermoFisher Scientific, Waltham, MA) and quality assessment using an Agilent 2100 Bioanalyzer (Agilent, Santa Clara, CA). As the PBC pellets were not collected primarily for RNA analyses, we used experimental approaches designed to address issues with storage-associated RNA degradation (described below). We restricted analyses to samples with RNA integrity number (RIN) > 5.

### DNA methylation

Whole genome DNA methylation was determined using commercially available Illumina Infinium MethylationEPIC BeadChip Assay (Illumina, San Diego, CA). 500 ng of DNA is first treated with sodium bisulfite, using the EZ DNA Methylation Kit (Zymo Research) and following the Illumina-specified instructions. Converted DNAs are then processed on the Illumina Infinium Methylation EPIC 8-Sample Beadchip following the Infinium HD Methylation 15019521v01 protocol. DNA from the conversion step is denatured and neutralized to prepare it for amplification. The denatured DNA is amplified overnight at 37 °C. Next, amplified bisulfite-converted DNA is enzymatically fragmented for 60 min at 37 °C, precipitated with isopropanol and air dried. DNA is then re-suspended in hybridization buffer. Eight samples are applied to each beadchip, kept separate with an IntelliHyb seal. The prepared beadchip is incubated overnight in a hybridization oven at 48 °C with rocking. The amplified and fragmented DNA samples anneal to locus-specific 50mers during hybridization. Following hybridization, unhybridized and non-specifically hybridized DNA is washed away, and the chip is prepared for staining and extension. The chip undergoes staining and extension in capillary flow-through chambers. Allele-specific single-base extension of the oligos on the beadchip, using the captured DNA as template, incorporates detectable labels on the beadchip and determines the methylation profile for the sample. After staining, beadchips are scanned using the Illumina iScan + with ICS v3.3.28, and intensity data is extracted with Illumina GenomeStudio software (GenomeStudio v2011.1 with Methylation Analysis Module v1.9.0).

### Gene transcription analyses

Whole transcriptome analyses were performed using the Human Clariom S Array (Affymetrix, ThermoFisher Scientific, Waltham, MA), following the GeneChip Pico protocol, which is optimized for degraded sample types including FFPE samples, frozen tissues and PBCs. This assay can generate robust expression profiles from as little as 100 pg of total RNA. Briefly, amplified double-stranded cDNAs are synthesized and converted to cRNAs via in vitro transcription. After clean-up, double-stranded, antisense cDNAs are synthesized using 20 µg cRNAs. Using 5.5 µg of cDNA samples are then fragmented and labeled, and then hybridized onto the Clariom S Human Arrays for 16 h at 45 °C. Arrays are then washed, stained and scanned using an Affymetrix GeneChip Scanner 3000.

### Bioinformatics and data analysis

For methylation data, raw data from Illumina Human Methylation EPIC arrays (IDAT files) were imported into R (r-project.org) using the Bioconductor minfi package^53^. These arrays have extensive quality control (QC) probes used to determine the quality of each processing step. Any arrays that failed QC were either re-processed or discarded. We also discarded any probes that have binding indistinguishable from background for > 5% of the subjects. We then background adjusted the raw probe intensities using ‘out of bounds’ probes, which are non-complementary probes that are not expected to hybridize and, thus, provide a measure of non-specific probe binding^54^, followed by a functional quantile normalization that uses control probes to eliminate technical differences between arrays^55^. Following normalization, we generated a principal components analysis (PCA) plot to evaluate the relative contribution of clinical variables (e.g. treatment allocation, baseline Epo level, gestational age, sex, severe bronchopulmonary dysplasia, necrotizing enterocolitis Bell’s stage 2b or 3^56^, intraventricular hemorrhage grade 3 or 4^57^, severe retinopathy of prematurity (requiring laser or bevacizumab), culture-proven sepsis, maternal race/ethnicity, CP status^48,49^ and GMFCS^50,51^) and cell components to the variability within each principal component (Supplemental Figures S1-13). Based on these plots, we included sex and race/ethnicity as confounders in our models. In addition, to control for other unobserved variability (for example, differences in cell composition from each sample), we used the Bioconductor sva package to estimate surrogate variables which we included in the models^58^. We made various inter-group comparisons using the Bioconductor limma package^59^ and DMRcate package^29^ to detect DMRs. This software accounts for the fact that individual CpG probes may be less reliable and suffer from both false positives and false negatives. Conversely, CpGs that are closely spaced tend to be highly correlated, with the correlation between CpGs dropping off as the genomic distance increases. Therefore, by grouping CpGs within genomic regions (within < 1000bps) and ‘smoothing’ the statistics used to detect differential methylation from adjacent CpGs, we increased power to detect DMRs. To reduce false positives, we required that any DMR consist of at least 10 CpGs and selected individual CpGs based on a false discovery rate (FDR) < 0.05. The group comparisons performed included: (1) Day 1 CP vs no CP, (2) Day 14 CP vs no CP, (3) the interaction (Day 1 to Day 14 change) CP vs no CP and (4) the Day 1 to 14 change within each diagnosis. Sensitivity analyses were also performed with parallel comparisons between controls and the CP cohort limited to the moderate and severely affected babies (GMFCS level ≥ 1). Given our study design, we fit different models depending on the comparison. For the first two comparisons, we fit a conventional analysis of variance (ANOVA) model, adjusting for sex, race, ethnicity and seven surrogate variables. For the remaining models, since we had repeated measures for each subject, we fit an ANOVA model that included patient-level blocking factors, an interaction between CP status and sampling day, and eight surrogate variables. To ensure goodness of fit for the individual CpG comparisons, we evaluated histograms of the resulting p-values, after which we combined into DMRs and tested for differential methylation.

For transcriptome data, we summarized and normalized transcript expression data using the RMA algorithm^60^, as implemented in the Bioconductor **oligo** package^61^. Analogous to the DNA methylation analyses, we used the Bioconductor **sva** package^62^ to estimate surrogate variables to adjust for known and unknown sources of technical and clinical variability. We then fit a linear mixed model that fits the patient as a random effect and includes these variables as well as the variable of interest (CP or control), and made comparisons using empirical Bayes adjusted contrasts^59^. We performed group comparisons as described above for the DNA methylation analyses. Genes were considered significant based on a FDR < 0.05^63^. We defined genes that may be affected by changes in methylation as those genes that fulfilled our significance criteria (FDR < 0.05) in a given contrast that were also within a 1 Mb region centered on a DMR. We performed several QC plots including normalized unscaled standard errors (NUSE), relative log expression (RLE), and MA plots using the RMA algorithm^52^ to evaluate the quality of the normalization and adequacy of surrogate variables to control for excess technical variability.

Secondarily, we evaluated the profile of genes that demonstrated significant up or downregulation between day 1 and 14 that was unique to CP cases as compared to controls. Impact analyses (iPathwayGuide) were performed to identify relevant pathways that were unique to each diagnosis. Functional information for candidate genes was derived from the GeneCards database (genecards.org).

## Supplementary Information

Supplementary Tables.

Supplementary Figure S1.

Supplementary Figure S2.

Supplementary Figure S3.

Supplementary Figure S4.

Supplementary Figure S5.

Supplementary Figure S6.

Supplementary Figure S7.

Supplementary Figure S8.

Supplementary Figure S9.

Supplementary Figure S10.

Supplementary Figure S11.

Supplementary Figure S12.

Supplementary Figure S13.

## Data Availability

The datasets generated during and/or analyzed during the current study are available from the corresponding author on reasonable request.
