# Supplementary material for: Whole genome methylation and transcriptome analyses to identify risk for cerebral palsy (CP) in extremely low gestational age neonates (ELGAN)
Source: Sci Rep. 2021 Mar 5;11:5305. doi: 10.1038/s41598-021-84214-9 (PMC7935929; doi:10.1038/s41598-021-84214-9)
Supplement: Supplementary file 1 — Supplementary Tables. [file 41598_2021_84214_MOESM1_ESM.docx]

Whole genome methylation and transcriptome analyses to identify risk for cerebral palsy (CP) in extremely low gestational age neonates (ELGAN)

*Supplemental Information*

An N. Massaro,^1*^ Theo K. Bammler,^2^ James W. MacDonald,^2^ Krystle M. Perez, ^3^ Bryan Comstock,^4^ Sandra E. Juul^3^

^1^Pediatrics – Division of Neonatology, Children’s National Health Systems and The George Washington University School of Medicine, Washington, DC; ^2^Department of Environmental & Occupational Health Sciences, University of Washington, Seattle, WA; ^3^ Pediatrics - Division of Neonatology, University of Washington, Seattle, WA; ^4^ Department of Biostatistics, University of Washington, Seattle, WA

**Supplemental Table S1. Differentially Methylated Regions in CP Group between Day 1 and 14**

| **Chr** | **Start** | **End** | **Width** | **Num CpGs** | **p-value** | **Max Prop Change** | **Mean Prop Change** | **Genes** |
| --- | --- | --- | --- | --- | --- | --- | --- | --- |
| chr14 | 1.06E+08 | 1.06E+08 | 2920 | 20 | 1.68e-30 | -0.073 | -0.04 | IGHJ6-001, IGHJ2-001, IGHJ1-001, IGHJ3P-001, IGHD7-27-001, IGHJ1P-001, IGHJ2P-001, IGHJ4-001, IGHJ3-001, IGHJ5-001 |
| chr11 | 2011216 | 2012411 | 1196 | 12 | 6.54e-29 | 0.111 | 0.019 | AC051649.6-001, MRPL23-AS1-001 |
| chr17 | 79004850 | 79007529 | 2680 | 16 | 3.44e-26 | 0.142 | 0.048 | BAIAP2-002, BAIAP2-003, BAIAP2-201, BAIAP2-001, BAIAP2-007, BAIAP2-AS1-201, BAIAP2-019, BAIAP2-011, BAIAP2-AS1-002, BAIAP2-024, BAIAP2-009, BAIAP2-004, BAIAP2-023, BAIAP2-014, BAIAP2-022, BAIAP2-AS1-001 |
| chr17 | 71258045 | 71259281 | 1237 | 11 | 1.57e-24 | 0.109 | 0.059 | CPSF4L-002, CPSF4L-001 |
| chr14 | 1.06E+08 | 1.06E+08 | 7210 | 23 | 2.37e-22 | -0.077 | -0.029 | IGHD6-19-001, IGHD5-18-001, IGHD3-16-001, IGHD2-15-001, AL122127.25-001, AL122127.25-002, IGHD4-17-001, IGHD1-20-001 |
| chr1 | 26644234 | 26646801 | 2568 | 14 | 3.11e-19 | -0.057 | -0.034 | CD52-001, UBXN11-201, UBXN11-001, CD52-002, CD52-003 |
| chr14 | 1.06E+08 | 1.06E+08 | 5085 | 25 | 8.36e-19 | -0.07 | -0.029 | IGHD6-13-001, IGHD5-12-001, IGHD3-10-001, IGHD3-9-001, IGHD2-8-001, IGHD4-11-001, IGHD1-14-001 |
| chr17 | 3704471 | 3708018 | 3548 | 16 | 3.73e-18 | 0.151 | 0.055 | ITGAE-001, CTD-3195I5.5-001 |
| chr2 | 7016509 | 7018885 | 2377 | 12 | 7.50e-14 | -0.083 | -0.041 | RSAD2-001, RSAD2-002, RSAD2-005, RSAD2-201 |
| chr16 | 57672082 | 57673993 | 1912 | 12 | 7.52e-12 | -0.071 | -0.03 | GPR56-009, GPR56-206, GPR56-032, GPR56-076, GPR56-015, GPR56-028, GPR56-016, GPR56-068, GPR56-066, GPR56-008, GPR56-067, GPR56-069, GPR56-045, GPR56-031, GPR56-017, GPR56-029, GPR56-013, GPR56-046 |
| chr10 | 21796152 | 21798698 | 2547 | 11 | 7.58e-12 | 0.09 | 0.036 |  |
| chr2 | 66671478 | 66673985 | 2508 | 15 | 7.79e-12 | -0.065 | -0.03 | MEIS1-012, MEIS1-007, MEIS1-018 |
| chr5 | 58652602 | 58654193 | 1592 | 11 | 1.76e-11 | 0.056 | 0.033 | PDE4D-015 |
| chr1 | 2.49E+08 | 2.49E+08 | 1603 | 11 | 1.83e-10 | 0.105 | 0.056 | LYPD8-001 |
| chr16 | 1494476 | 1496312 | 1837 | 14 | 9.24e-10 | 0.097 | 0.035 | CCDC154-201, CCDC154-001, LA16c-390E6.5-002 |
| chr2 | 33359059 | 33359688 | 630 | 11 | 1.47e-09 | 0.109 | 0.057 | LTBP1-203, LTBP1-008, LTBP1-001, LTBP1-007, LTBP1-204, LTBP1-016 |
| chr5 | 76247647 | 76249776 | 2130 | 13 | 1.52e-09 | -0.092 | -0.031 | CRHBP-001, CRHBP-002, CRHBP-003 |
| chr20 | 11897564 | 11900410 | 2847 | 12 | 1.92e-09 | -0.073 | -0.02 | BTBD3-201, BTBD3-008, RP4-742J24.2-001, BTBD3-007, BTBD3-009, BTBD3-012, BTBD3-011, BTBD3-010 |
| chr11 | 63302736 | 63305749 | 3014 | 11 | 3.77e-09 | -0.066 | -0.026 | RARRES3-001, RARRES3-201, RARRES3-002, RARRES3-003, RARRES3-004 |
| chr7 | 922051 | 923984 | 1934 | 12 | 4.12e-09 | 0.113 | 0.038 | GET4-002, GET4-003 |
| chr16 | 87099124 | 87102691 | 3568 | 19 | 4.60e-09 | 0.171 | 0.04 | RP11-134D3.2-001 |
| chr17 | 80192161 | 80195737 | 3577 | 23 | 6.91e-09 | 0.068 | 0.018 | SLC16A3-008, SLC16A3-017, SLC16A3-009, SLC16A3-001, SLC16A3-010, SLC16A3-018 |
| chr3 | 46718369 | 46719834 | 1466 | 10 | 1.61e-08 | 0.094 | 0.031 | ALS2CL-008, ALS2CL-007 |
| chr2 | 98329337 | 98330493 | 1157 | 10 | 2.17e-08 | -0.07 | -0.022 | ZAP70-001, ZAP70-002 |
| chr17 | 46666926 | 46668733 | 1808 | 10 | 2.96e-08 | -0.047 | -0.025 | HOXB-AS3-007, HOXB3-006, HOXB-AS3-017, HOXB3-003, HOXB-AS3-015, HOXB-AS3-014, HOXB3-004, HOXB-AS3-009, HOXB-AS3-005, HOXB-AS3-010, HOXB3-002 |
| chr15 | 40632399 | 40633816 | 1418 | 12 | 3.35e-08 | 0.05 | 0.019 | C15orf52-003, C15orf52-005, C15orf52-004, C15orf52-002, C15orf52-007 |
| chr17 | 80200634 | 80203277 | 2644 | 13 | 5.26e-08 | 0.239 | 0.061 | CSNK1D-028, CSNK1D-029 |
| chr2 | 1.14E+08 | 1.14E+08 | 2963 | 12 | 1.58e-07 | 0.041 | 0.011 | IL1A-001 |
| chr7 | 27196286 | 27199752 | 3467 | 21 | 1.92e-07 | -0.073 | -0.019 | HOXA7-001, HOXA7-002, HOXA7-003, RP1-170O19.21-001 |
| chr11 | 65190180 | 65192550 | 2371 | 12 | 2.41e-07 | -0.108 | -0.034 | NEAT1-002, NEAT1-001, NEAT1-202 |
| chr22 | 17955274 | 17956641 | 1368 | 10 | 3.96e-07 | 0.09 | 0.033 | CECR2-201, CECR2-001 |
| chr12 | 14996143 | 14997216 | 1074 | 12 | 4.59e-07 | 0.139 | 0.035 | ART4-001, ART4-002, ART4-003, ART4-004 |
| chr11 | 73356316 | 73358107 | 1792 | 15 | 1.33e-06 | 0.052 | 0.018 | PLEKHB1-003, PLEKHB1-001, PLEKHB1-002, PLEKHB1-004, PLEKHB1-007, PLEKHB1-013, PLEKHB1-009, PLEKHB1-016, PLEKHB1-015, PLEKHB1-023, PLEKHB1-019, PLEKHB1-017, PLEKHB1-020, PLEKHB1-010, PLEKHB1-018, PLEKHB1-011, PLEKHB1-012, PLEKHB1-014 |
| chr7 | 1022098 | 1023156 | 1059 | 12 | 1.38e-06 | 0.043 | 0.021 | CYP2W1-001, CYP2W1-002 |
| chr3 | 1.29E+08 | 1.29E+08 | 1027 | 10 | 1.48e-06 | 0.108 | 0.041 | GP9-001 |
| chr1 | 6418521 | 6419906 | 1386 | 10 | 1.91e-06 | -0.044 | -0.023 | ACOT7-005, ACOT7-004, ACOT7-201 |
| chr16 | 88903964 | 88907370 | 3407 | 10 | 2.72e-06 | 0.073 | 0.029 | GALNS-010, GALNS-012, GALNS-011, GALNS-007 |
| chr12 | 1.25E+08 | 1.25E+08 | 1770 | 10 | 2.79e-06 | -0.053 | 0.015 |  |
| chr14 | 24539335 | 24540773 | 1439 | 14 | 5.62e-06 | 0.069 | 0.027 | CPNE6-201, CPNE6-003, CPNE6-002, CPNE6-202, CPNE6-017, CPNE6-014, CPNE6-018, CPNE6-005, CPNE6-011, CPNE6-004, CPNE6-019, CPNE6-013, CPNE6-016, CPNE6-008, CPNE6-015, CPNE6-010, CPNE6-009, CPNE6-012 |
| chr6 | 1.5E+08 | 1.5E+08 | 1368 | 13 | 6.87e-06 | -0.052 | -0.028 | ZC3H12D-201, ZC3H12D-003, ZC3H12D-002, ZC3H12D-202, ZC3H12D-004, ZC3H12D-203 |
| chr8 | 6419438 | 6421171 | 1734 | 13 | 7.25e-06 | 0.063 | 0.034 | ANGPT2-001, ANGPT2-002, ANGPT2-201, ANGPT2-003 |
| chr12 | 10182372 | 10184399 | 2028 | 13 | 1.02e-05 | 0.069 | 0.021 | CLEC9A-001, RP11-133L14.5-001, CLEC9A-002 |
| chr17 | 57915665 | 57918682 | 3018 | 12 | 1.25e-05 | -0.085 | -0.028 | MIR21-201, VMP1-012, VMP1-013, VMP1-016, VMP1-004 |
| chr11 | 1.18E+08 | 1.18E+08 | 1841 | 11 | 1.52e-05 | 0.049 | -0.004 | CD3G-002, CD3D-001, CD3G-201, CD3D-003, CD3D-004, CD3G-004, CD3G-005, CD3D-002, CD3G-006, CD3G-001, CD3G-003, CD3D-005 |
| chr18 | 56296094 | 56296607 | 514 | 10 | 1.95e-05 | 0.081 | 0.031 | ALPK2-001, RPL9P31-001 |
| chr17 | 73719693 | 73720840 | 1148 | 10 | 2.83e-05 | 0.052 | 0.014 | ITGB4-003, ITGB4-005 |
| chr15 | 29212340 | 29213860 | 1521 | 10 | 4.79e-05 | -0.037 | -0.015 | APBA2-201, APBA2-202, APBA2-008 |
| chr16 | 1537775 | 1540144 | 2370 | 13 | 5.35e-05 | 0.066 | 0.025 | PTX4-201, PTX4-002, PTX4-001 |
| chr6 | 25041912 | 25043046 | 1135 | 15 | 5.40e-05 | -0.055 | -0.019 | RP3-425P12.5-001, FAM65B-201, RP11-367G6.3-001 |
| chr7 | 1.35E+08 | 1.35E+08 | 1205 | 14 | 6.14e-05 | -0.056 | -0.023 | TMEM140-001, AC083862.1-201, C7orf49-013, TMEM140-002 |
| chr17 | 25798180 | 25799447 | 1268 | 11 | 6.85e-05 | -0.034 | -0.016 | KSR1-201, KSR1-001, KSR1-008, KSR1-007 |
| chr1 | 1.53E+08 | 1.53E+08 | 1476 | 10 | 8.75e-05 | 0.079 | 0.015 | S100A8-002, S100A8-001, S100A8-003 |
| chr6 | 32907385 | 32909781 | 2397 | 16 | 9.57e-05 | -0.066 | -0.025 | HLA-DMB-006, HLA-DMB-213, HLA-DMB-001, HLA-DMB-003 |
| chr8 | 27468166 | 27470225 | 2060 | 13 | 1.02e-04 | -0.117 | -0.029 | CLU-004, CLU-014, CLU-015, CLU-011, CLU-016, CLU-017, CLU-010, CLU-009, CLU-013, CLU-012, CLU-003, CLU-018, CLU-201, CLU-002 |
| chr16 | 1583391 | 1584516 | 1126 | 13 | 1.45e-04 | -0.059 | -0.043 | IFT140-006, TMEM204-002 |
| chr1 | 1265511 | 1267559 | 2049 | 14 | 1.64e-04 | 0.061 | 0.022 | TAS1R3-001 |
| chr17 | 4673292 | 4675292 | 2001 | 10 | 1.66e-04 | 0.116 | 0.016 | TM4SF5-001, TM4SF5-002 |
| chr15 | 1.02E+08 | 1.02E+08 | 1293 | 10 | 1.69e-04 | 0.081 | 0.024 | CHSY1-002, CHSY1-005, CHSY1-004, CHSY1-003 |
| chr12 | 1.24E+08 | 1.24E+08 | 1797 | 11 | 1.70e-04 | 0.149 | 0.041 | CCDC92-009, CCDC92-008, CCDC92-002 |
| chr1 | 2.06E+08 | 2.06E+08 | 1772 | 11 | 1.82e-04 | 0.097 | 0.022 | CTSE-001, CTSE-002, CTSE-201, CTSE-202 |
| chr17 | 7791630 | 7794516 | 2887 | 10 | 2.23e-04 | 0.067 | 0.007 | CHD3-001, CHD3-002, CHD3-006, CHD3-013, CHD3-012 |
| chr17 | 79798629 | 79801920 | 3292 | 18 | 2.52e-04 | 0.116 | 0.026 | P4HB-012, P4HB-030, RP11-498C9.2-001 |
| chr1 | 1563001 | 1565541 | 2541 | 12 | 2.62e-04 | -0.033 | -0.011 | MMP23B-001, MIB2-012, MIB2-015, MIB2-013, MIB2-014, MIB2-032, MIB2-024 |
| chr19 | 39738664 | 39740501 | 1838 | 10 | 4.23e-04 | 0.069 | 0.022 | IFNL4-001 |
| chr11 | 67809271 | 67811721 | 2451 | 15 | 4.54e-04 | 0.067 | 0.017 | TCIRG1-009, TCIRG1-013, TCIRG1-012, TCIRG1-014, TCIRG1-016, TCIRG1-004 |
| chr17 | 19313391 | 19314618 | 1228 | 10 | 5.31e-04 | 0.039 | 0.016 | RNF112-001, RNF112-002, RNF112-005, CTB-187M2.2-001, RNF112-006 |
| chr22 | 45608023 | 45608713 | 691 | 11 | 5.93e-04 | 0.036 | 0.02 | KIAA0930-004, KIAA0930-009, KIAA0930-012, KIAA0930-201, KIAA0930-003, KIAA0930-011, KIAA0930-010 |
| chr3 | 1.49E+08 | 1.49E+08 | 1377 | 11 | 6.19e-04 | -0.058 | -0.021 | TM4SF1-001, TM4SF1-003, TM4SF1-AS1-002, TM4SF1-005, TM4SF1-004, TM4SF1-AS1-001 |
| chr6 | 31539539 | 31541461 | 1923 | 20 | 6.93e-04 | -0.049 | -0.025 | LTA-001, TNF-001, LTA-002, LTA-004, LTA-003 |
| chr17 | 80539144 | 80542340 | 3197 | 15 | 7.30e-04 | 0.087 | 0.029 | snoU13.58-201, FOXK2-010, FOXK2-011 |
| chr2 | 85660497 | 85664005 | 3509 | 11 | 7.75e-04 | 0.049 | 0.016 | SH2D6-001, Y_RNA.546-201, SH2D6-005, SH2D6-006 |
| chr11 | 64658903 | 64661321 | 2419 | 14 | 7.85e-04 | 0.061 | 0.015 | AP001187.1-201, MIR194-2-201, MIR192-201, MIR194-2-001, RP11-665N17.4-001 |
| chr14 | 24867164 | 24868470 | 1307 | 10 | 8.94e-04 | 0.043 | 0.017 | NYNRIN-001 |
| chr3 | 1.52E+08 | 1.52E+08 | 1519 | 11 | 9.30e-04 | -0.075 | -0.025 | MBNL1-201, MBNL1-202, MBNL1-004, MBNL1-001, MBNL1-AS1-002, MBNL1-016, MBNL1-017, MBNL1-002, MBNL1-003, MBNL1-015, MBNL1-AS1-001 |
| chr6 | 10555114 | 10556523 | 1410 | 13 | 9.58e-04 | 0.056 | 0.028 | GCNT2-001, GCNT2-016 |
| chr3 | 50359363 | 50361180 | 1818 | 12 | 1.03e-03 | 0.069 | 0.021 | HYAL2-001, HYAL2-002, HYAL2-009, HYAL2-008, HYAL2-010, HYAL2-006, HYAL2-004, HYAL2-003, HYAL2-007, HYAL2-005 |
| chr16 | 89363047 | 89364225 | 1179 | 10 | 1.12e-03 | 0.048 | 0.016 | AC137932.5-001, AC137932.5-002 |
| chr20 | 50418952 | 50419348 | 397 | 10 | 1.21e-03 | 0.048 | 0.027 | SALL4-001, SALL4-201, SALL4-002, SALL4-003 |
| chr3 | 1.87E+08 | 1.87E+08 | 2263 | 12 | 1.28e-03 | -0.036 | -0.017 | SNORA63-201, SNORA63.6-201, SNORA81-201, SNORD2-201, EIF4A2-026, EIF4A2-022, EIF4A2-020, EIF4A2-019, EIF4A2-007, EIF4A2-023, EIF4A2-021, EIF4A2-025, EIF4A2-027, EIF4A2-024, EIF4A2-018, RP11-573D15.9-001, SNORA4-201 |
| chr8 | 1.42E+08 | 1.42E+08 | 2819 | 10 | 1.70e-03 | 0.076 | 0.017 |  |
| chr2 | 1.93E+08 | 1.93E+08 | 1800 | 10 | 1.78e-03 | -0.049 | -0.021 | SDPR-001, AC098617.1-003 |
| chr13 | 50701960 | 50703841 | 1882 | 13 | 1.87e-03 | 0.05 | 0.018 |  |
| chr12 | 6745057 | 6746030 | 974 | 14 | 2.48e-03 | 0.04 | 0.013 | LPAR5-001, LPAR5-002, ACRBP-004 |
| chr9 | 1.13E+08 | 1.13E+08 | 3316 | 14 | 2.51e-03 | 0.053 | 0.01 | AKAP2-003, AKAP2-004 |
| chr14 | 23623480 | 23625141 | 1662 | 11 | 2.52e-03 | 0.109 | 0.028 | SLC7A8-006, RNU6-1138P-201, SLC7A8-201, SLC7A8-002, SLC7A8-007, SLC7A8-005 |
| chr16 | 87738623 | 87740757 | 2135 | 11 | 2.59e-03 | 0.038 | 0.011 | FLJ00104-201, KLHDC4-025 |
| chr2 | 27301057 | 27301943 | 887 | 10 | 2.62e-03 | 0.033 | 0.019 | EMILIN1-001 |
| chr6 | 29598695 | 29599836 | 1142 | 11 | 2.72e-03 | 0.04 | 0.018 | GABBR1-201, GABBR1-003, GABBR1-001, GABBR1-015, GABBR1-009, GABBR1-013, GABBR1-016, GABBR1-017, GABBR1-014, GABBR1-005, GABBR1-006 |
| chr6 | 31845879 | 31848099 | 2221 | 15 | 2.77e-03 | 0.037 | 0.014 | SLC44A4-001, SLC44A4-201, SLC44A4-004, SLC44A4-003, SLC44A4-002, SLC44A4-202 |
| chr19 | 46806117 | 46807660 | 1544 | 11 | 3.11e-03 | 0.074 | 0.024 | HIF3A-006, HIF3A-202, HIF3A-013, HIF3A-015, HIF3A-023, HIF3A-024 |
| chr17 | 58499065 | 58500186 | 1122 | 10 | 3.13e-03 | 0.04 | 0.015 | C17orf64-001, C17orf64-003, C17orf64-002, USP32-014, USP32-015 |
| chr7 | 99970074 | 99972461 | 2388 | 10 | 3.17e-03 | 0.033 | 0.014 | PILRA-001, PILRA-006, PILRA-005, PILRA-002, PILRA-007, PILRA-004 |
| chr2 | 85810744 | 85812023 | 1280 | 10 | 3.19e-03 | -0.033 | -0.014 | VAMP5-001 |
| chr15 | 74500175 | 74502788 | 2614 | 13 | 3.23e-03 | -0.066 | 0 | STRA6-002, STRA6-009, STRA6-007, STRA6-003, STRA6-010, STRA6-008, STRA6-011 |
| chr16 | 29830908 | 29833153 | 2246 | 15 | 3.42e-03 | -0.041 | -0.011 | MVP-001, MVP-201, MVP-202, MVP-015, MVP-014, MVP-018, MVP-002, AC009133.12-002, MVP-009, MVP-013, MVP-011, MVP-008, AC009133.20-001, AC009133.12-001, MVP-017, MVP-010 |
| chr19 | 17515486 | 17517762 | 2277 | 13 | 3.68e-03 | -0.048 | -0.017 | BST2-001, CTD-2521M24.9-002, BST2-003, MVB12A-012, MVB12A-009, MVB12A-011, BST2-004, CTD-2521M24.9-001, CTD-2521M24.9-006, CTD-2521M24.9-012, CTD-2521M24.9-003, CTD-2521M24.9-008, CTD-2521M24.9-007, CTD-2521M24.9-004, CTD-2521M24.9-013, CTD-2521M24.9-009, CTD-2521M24.9-005, MVB12A-016, CTD-2521M24.9-011, CTD-2521M24.9-010 |
| chr16 | 85095535 | 85097151 | 1617 | 12 | 4.10e-03 | 0.057 | 0.012 | KIAA0513-001 |
| chr10 | 1.14E+08 | 1.14E+08 | 2025 | 13 | 4.29e-03 | 0.074 | 0.022 | ACSL5-001, ACSL5-002, ACSL5-201, ACSL5-203, ACSL5-204, ACSL5-003 |
| chr19 | 5838729 | 5839901 | 1173 | 10 | 4.60e-03 | 0.035 | 0.012 | FUT6-001, FUT6-201, FUT6-003, FUT6-009, FUT6-002, FUT6-006, FUT6-004, FUT6-005, FUT6-007, FUT6-010 |
| chr6 | 33039396 | 33041583 | 2188 | 18 | 5.12e-03 | -0.055 | -0.027 | HLA-DPA1-205, HLA-DPA1-007, HLA-DPA1-003 |
| chr16 | 87992457 | 87993548 | 1092 | 10 | 5.58e-03 | -0.048 | -0.016 | BANP-012, BANP-203 |
| chr7 | 27162051 | 27163331 | 1281 | 10 | 5.74e-03 | 0.065 | 0.016 | HOXA-AS2-001, HOXA-AS2-005, HOXA-AS2-003, HOXA-AS2-002, HOXA-AS2-004, HOXA-AS2-007 |
| chr20 | 62366755 | 62369445 | 2691 | 18 | 5.83e-03 | -0.033 | -0.015 | SLC2A4RG-004, LIME1-006, LIME1-008, LIME1-010, RP4-583P15.14-002, LIME1-004, RP4-583P15.14-001, LIME1-001, ZGPAT-001, LIME1-011, LIME1-005, LIME1-007, LIME1-002, LIME1-003, LIME1-009 |
| chr6 | 31589926 | 31591345 | 1420 | 15 | 6.06e-03 | 0.047 | 0.015 | SNORA38-201, PRRC2A-001, PRRC2A-002, PRRC2A-013, PRRC2A-003 |
| chr6 | 1.33E+08 | 1.33E+08 | 1319 | 10 | 6.51e-03 | -0.028 | -0.015 | RPS12-001, SNORA33-201, SNORD101-201, SNORD100-201, RPS12-002 |
| chr1 | 1.71E+08 | 1.71E+08 | 1563 | 10 | 6.87e-03 | -0.032 | -0.004 | FMO1-003, FMO1-004, FMO1-002 |
| chr7 | 871436 | 872797 | 1362 | 12 | 7.10e-03 | -0.047 | -0.019 | SUN1-003, SUN1-005, SUN1-007, SUN1-001, SUN1-004, SUN1-019, SUN1-202, SUN1-018, SUN1-022, SUN1-020, SUN1-023 |
| chr5 | 78985425 | 78986160 | 736 | 11 | 7.19e-03 | 0.058 | 0.019 | CMYA5-001 |
| chr8 | 82191575 | 82192606 | 1032 | 10 | 7.39e-03 | 0.043 | 0.014 | FABP5-001, FABP5-002, RP11-363E6.3-002, RP11-363E6.3-001, RP11-363E6.4-001 |
| chr15 | 97320595 | 97322893 | 2299 | 11 | 7.65e-03 | -0.023 | -0.011 | SPATA8-AS1-002 |
| chr7 | 1080836 | 1083209 | 2374 | 11 | 8.72e-03 | 0.061 | 0.001 | GPR146-003, GPR146-004 |
| chr1 | 32040692 | 32041940 | 1249 | 11 | 8.97e-03 | 0.055 | 0.013 | TINAGL1-001, RP11-73M7.1-001, TINAGL1-201, TINAGL1-202, TINAGL1-006, TINAGL1-007, TINAGL1-008, TINAGL1-005, TINAGL1-002, TINAGL1-203 |
| chr6 | 33043220 | 33044510 | 1291 | 10 | 9.62e-03 | -0.063 | -0.02 | HLA-DPB1-001, HLA-DPA1-205, HLA-DPA1-007, HLA-DPB1-004, HLA-DPA1-003, HLA-DPB1-202 |
| chr7 | 27185136 | 27188770 | 3635 | 23 | 1.01e-02 | -0.049 | -0.015 | HOXA5-001, HOXA6-001, HOXA-AS3-001, HOXA-AS3-005, HOXA6-002, HOXA-AS3-002 |
| chr11 | 6425422 | 6427501 | 2080 | 14 | 1.12e-02 | -0.023 | -0.009 | APBB1-010, APBB1-017, APBB1-006, APBB1-009, APBB1-004, APBB1-007, APBB1-003, APBB1-008, APBB1-005, APBB1-025, APBB1-024, APBB1-014, APBB1-018 |
| chr7 | 27207996 | 27212762 | 4767 | 36 | 1.14e-02 | -0.076 | -0.011 | HOXA10-001, MIR196B-201, HOXA9-004, HOXA9-005, HOXA9-002, HOXA10-006, HOXA10-AS-002, HOXA10-AS-003, HOXA10-005, HOXA10-AS-001, HOXA10-004 |
| chr15 | 68497730 | 68499369 | 1640 | 12 | 1.16e-02 | 0.04 | 0.016 | CALML4-005, CALML4-002, CALML4-003, CALML4-001, CALML4-004, CALML4-201 |
| chr19 | 49992293 | 49993758 | 1466 | 10 | 1.17e-02 | -0.022 | -0.012 | SNORD33-201, SNORD35A-201, SNORD32A-201, SNORD34-201, RPL13A-001, RPL13A-009, RPL13A-011, RPL13A-007, RPL13A-010, RPL13A-003, RPL13A-012, RPL13A-008, RPL13A-006, RPL13A-004, RPL13A-005, RPL13A-002 |
| chr6 | 32919700 | 32921233 | 1534 | 11 | 1.20e-02 | -0.045 | -0.011 | HLA-DMA-001, HLA-DMA-002, HLA-DMA-004, XXbac-BPG181M17.5-001, HLA-DMA-006, HLA-DMA-005, HLA-DMA-003 |
| chr15 | 59156878 | 59158587 | 1710 | 10 | 1.24e-02 | 0.081 | 0.019 | RP11-30K9.5-001, ZNF444P1-001, RNF111-007 |
| chr1 | 1.12E+08 | 1.12E+08 | 1590 | 13 | 1.42e-02 | 0.077 | -0.011 | DENND2D-002, CHI3L2-001, DENND2D-003, DENND2D-006, DENND2D-007 |
| chr15 | 63114897 | 63116514 | 1618 | 10 | 1.44e-02 | -0.019 | -0.008 | MIR190A-201 |
| chr1 | 42383900 | 42385941 | 2042 | 17 | 1.56e-02 | -0.058 | -0.018 | HIVEP3-002, HIVEP3-001, HIVEP3-005 |
| chr3 | 1.55E+08 | 1.55E+08 | 1434 | 11 | 1.64e-02 | -0.035 | -0.008 | PLCH1-201 |
| chr6 | 31542556 | 31543686 | 1131 | 15 | 1.64e-02 | 0.071 | 0.014 | TNF-001 |
| chr19 | 4677230 | 4679533 | 2304 | 10 | 1.67e-02 | 0.059 | 0.017 | AC005594.3-001 |
| chr17 | 80349836 | 80351457 | 1622 | 13 | 1.81e-02 | 0.07 | 0.01 | OGFOD3-011, OGFOD3-004, OGFOD3-012 |
| chr1 | 76261799 | 76262984 | 1186 | 10 | 1.88e-02 | -0.055 | -0.013 | MSH4-001 |
| chr7 | 27153212 | 27154387 | 1176 | 12 | 1.97e-02 | 0.056 | 0.025 | HOXA-AS2-006, HOXA-AS2-011, HOXA-AS2-008, HOXA3-004, HOXA-AS2-012 |
| chr6 | 31544694 | 31546067 | 1374 | 10 | 2.03e-02 | -0.023 | -0.012 | TNF-001 |
| chr6 | 32062160 | 32063126 | 967 | 10 | 2.21e-02 | -0.02 | -0.012 |  |
| chr1 | 1.55E+08 | 1.55E+08 | 1298 | 11 | 2.23e-02 | -0.029 | -0.009 | DCST2-201, DCST1-001, DCST1-002, DCST1-003, DCST2-002, DCST1-201, DCST1-006, DCST2-001, DCST1-005 |
| chr12 | 6657744 | 6658945 | 1202 | 10 | 2.33e-02 | 0.066 | 0.017 | IFFO1-010, IFFO1-004, IFFO1-005 |
| chr8 | 1.42E+08 | 1.42E+08 | 1854 | 15 | 2.38e-02 | 0.057 | 0.021 | SLC45A4-201, RP11-10J21.4-001, SLC45A4-003, RP11-10J21.3-001 |
| chr6 | 32020750 | 32026350 | 5601 | 36 | 2.38e-02 | -0.025 | -0.01 |  |
| chr17 | 79030033 | 79031712 | 1680 | 12 | 2.57e-02 | 0.084 | 0.018 | BAIAP2-017, BAIAP2-016, BAIAP2-018, BAIAP2-030, BAIAP2-015 |
| chr13 | 78313933 | 78316150 | 2218 | 12 | 2.58e-02 | 0.065 | 0.019 | SLAIN1-201, SLAIN1-202, SLAIN1-001, SLAIN1-002, SLAIN1-011, SLAIN1-010, SLAIN1-013, SLAIN1-014, SLAIN1-012 |
| chr1 | 25256939 | 25258332 | 1394 | 24 | 2.62e-02 | 0.11 | 0.017 | RUNX3-001, RUNX3-004, RUNX3-202 |
| chr19 | 13055829 | 13056745 | 917 | 12 | 2.69e-02 | -0.056 | -0.011 | RAD23A-001, RAD23A-201, RAD23A-002, CALR-005, CALR-007, RAD23A-006, RAD23A-007, CTC-425F1.4-001, RAD23A-003, RAD23A-005, RAD23A-004 |
| chr16 | 89571489 | 89573955 | 2467 | 11 | 2.72e-02 | 0.037 | 0.013 | SPG7-001, SPG7-002, RP11-104N10.1-001, SPG7-009, SPG7-008, SPG7-022 |
| chr20 | 30406052 | 30407388 | 1337 | 13 | 2.75e-02 | 0.048 | 0.013 | MYLK2-001, MYLK2-002 |
| chr15 | 91427184 | 91428456 | 1273 | 13 | 2.83e-02 | 0.026 | 0.01 | FES-001, FES-005, FES-006, FES-017, FES-007, FES-011, FES-018, FES-201, FES-010, FES-003, FES-019, FES-009, FES-012, FES-020 |
| chr6 | 30848178 | 30851753 | 3576 | 29 | 2.89e-02 | 0.046 | 0.012 | DDR1-005, DDR1-002, DDR1-003, DDR1-204, DDR1-205, DDR1-013, DDR1-031, DDR1-012, DDR1-016, DDR1-014, DDR1-021, DDR1-036, DDR1-206, DDR1-207, DDR1-001, DDR1-010, DDR1-011, DDR1-004, DDR1-025, DDR1-027, DDR1-046, DDR1-042, DDR1-037, DDR1-026, DDR1-047, DDR1-024, DDR1-033, DDR1-034, DDR1-032, DDR1-020, DDR1-028, DDR1-030, DDR1-035, DDR1-048, DDR1-039, DDR1-049, DDR1-038, DDR1-043, DDR1-045, DDR1-044, DDR1-040, DDR1-029, DDR1-041 |
| chr15 | 74494241 | 74496648 | 2408 | 19 | 3.37e-02 | 0.04 | 0.007 | STRA6-001, RP11-60L3.1-004, RP11-60L3.1-001, RP11-60L3.1-003, STRA6-005, STRA6-004, STRA6-015, STRA6-020, STRA6-013, STRA6-016, STRA6-021, STRA6-012, STRA6-014 |
| chr19 | 55549414 | 55550348 | 935 | 11 | 3.41e-02 | 0.062 | 0.023 | GP6-002, GP6-003, GP6-001 |
| chr7 | 1.56E+08 | 1.56E+08 | 1937 | 11 | 3.67e-02 | 0.026 | -0.001 | LINC01006-006, LINC01006-003, LINC01006-004 |
| chr19 | 10735474 | 10736448 | 975 | 11 | 3.71e-02 | 0.048 | 0.011 | SLC44A2-004, SLC44A2-003, SLC44A2-012, SLC44A2-009, SLC44A2-010, SLC44A2-011 |
| chr16 | 2983147 | 2984768 | 1622 | 10 | 3.78e-02 | 0.034 | 0.016 | FLYWCH1-006, FLYWCH1-004, FLYWCH1-011, FLYWCH1-007, FLYWCH1-012 |
| chr7 | 30959709 | 30961331 | 1623 | 11 | 4.13e-02 | -0.036 | -0.006 | AQP1-002, AQP1-003, AQP1-202 |
| chr15 | 77285570 | 77287906 | 2337 | 12 | 4.14e-02 | 0.046 | 0.009 | PSTPIP1-201, PSTPIP1-002, PSTPIP1-001, PSTPIP1-012, PSTPIP1-017, PSTPIP1-009, PSTPIP1-006, PSTPIP1-003, PSTPIP1-005, PSTPIP1-004, PSTPIP1-007, PSTPIP1-018, PSTPIP1-008 |
| chr11 | 33562503 | 33564344 | 1842 | 10 | 4.19e-02 | 0.048 | 0.007 | KIAA1549L-002, KIAA1549L-001, KIAA1549L-201, KIAA1549L-003 |
| chr11 | 312518 | 314493 | 1976 | 17 | 4.28e-02 | -0.049 | -0.015 | IFITM1-003, IFITM1-001, IFITM1-004, RP11-326C3.7-001, IFITM1-002 |
| chr6 | 32017024 | 32019178 | 2155 | 11 | 4.66e-02 | -0.031 | -0.011 |  |
| chr8 | 19459672 | 19460935 | 1264 | 10 | 5.06e-02 | 0.052 | 0.017 | CSGALNACT1-004, CSGALNACT1-003, CSGALNACT1-012, CSGALNACT1-005, CSGALNACT1-014, CSGALNACT1-013 |
| chr1 | 3562370 | 3564676 | 2307 | 11 | 5.34e-02 | 0.109 | 0.016 | WRAP73-001, WRAP73-002, WRAP73-006, WRAP73-005, WRAP73-010 |
| chr19 | 4517498 | 4519155 | 1658 | 12 | 5.50e-02 | 0.062 | 0.005 | PLIN4-001 |
| chr6 | 30130109 | 30132226 | 2118 | 32 | 5.59e-02 | -0.102 | -0.015 | TRIM15-203, TRIM15-001, TRIM10-001, TRIM10-002 |
| chr17 | 62777648 | 62778413 | 766 | 13 | 5.87e-02 | 0.045 | 0.014 | hsa-mir-6080.1-201, hsa-mir-6080.1-001, hsa-mir-6080.1-002 |
| chr4 | 24795830 | 24797176 | 1347 | 11 | 5.98e-02 | 0.044 | 0.017 | SOD3-001 |
| chr22 | 19512228 | 19513971 | 1744 | 13 | 6.28e-02 | 0.038 | 0.014 | CLDN5-003, CLDN5-001, CLDN5-002 |
| chr1 | 1.5E+08 | 1.5E+08 | 1121 | 11 | 6.43e-02 | 0.023 | 0.004 | MTMR11-201, MTMR11-001, MTMR11-202, MTMR11-203, MTMR11-003, MTMR11-007, MTMR11-005, MTMR11-002, MTMR11-004 |
| chr6 | 32904061 | 32905320 | 1260 | 14 | 6.58e-02 | 0.073 | 0.018 | AL645941.1-201, HLA-DMB-006, HLA-DMB-007, HLA-DMB-004, HLA-DMB-005 |
| chr11 | 62620843 | 62622234 | 1392 | 11 | 6.73e-02 | -0.04 | -0.011 | SNHG1-201, SNHG1-202, SNHG1-203, SLC3A2-002, SLC3A2-001, SLC3A2-003, SLC3A2-201, SNHG1-204, SNHG1-205, SNHG1-206, SNHG1-207, SNHG1-208, SNHG1-209, SNHG1-022, SLC3A2-026, SNHG1-016, SNHG1-013, SNHG1-025, SNHG1-004, SNHG1-002, SNHG1-011, SNHG1-019, SNHG1-003, SNHG1-023, SNHG1-006, SNHG1-005, SNHG1-001, SNHG1-017, SNHG1-010, SNHG1-008, SNHG1-021, SNHG1-020, SNHG1-007, SNHG1-018, SNHG1-009, SNHG1-012, SNHG1-014, SNHG1-015, SNHG1-024 |
| chr6 | 32941825 | 32943025 | 1201 | 12 | 6.94e-02 | -0.03 | -0.017 | BRD2-005, BRD2-006, BRD2-203, BRD2-011, BRD2-012, XXbac-BPG181M17.6-001, BRD2-026, BRD2-023 |
| chr11 | 1.19E+08 | 1.19E+08 | 1396 | 10 | 7.01e-02 | -0.027 | -0.013 | BCL9L-001, BCL9L-010, MIR4492-201 |
| chr11 | 1773018 | 1775244 | 2227 | 10 | 7.88e-02 | -0.085 | -0.017 | IFITM10-001, IFITM10-004, CTSD-004, CTSD-003 |
| chr2 | 66663813 | 66665027 | 1215 | 12 | 8.07e-02 | -0.034 | -0.008 | MEIS1-001, MEIS1-003, MEIS1-201, MEIS1-202, MEIS1-002, MEIS1-005, MEIS1-008, MEIS1-016, MEIS1-017 |
| chr17 | 1943561 | 1945509 | 1949 | 14 | 8.11e-02 | 0.124 | 0.018 | DPH1-019, DPH1-005, DPH1-009, OVCA2-001, DPH1-016, RP11-667K14.4-001, DPH1-006, DPH1-017, DPH1-012, DPH1-018 |
| chr10 | 72362292 | 72363272 | 981 | 11 | 8.54e-02 | -0.026 | -0.009 | PRF1-001, PRF1-201 |
| chr6 | 90271152 | 90272492 | 1341 | 13 | 8.66e-02 | 0.062 | 0.015 | ANKRD6-004, ANKRD6-006, ANKRD6-018, ANKRD6-017 |
| chr21 | 47878552 | 47878993 | 442 | 10 | 8.75e-02 | -0.013 | -0.004 | DIP2A-201, DIP2A-001, DIP2A-012, DIP2A-202, DIP2A-003, DIP2A-002, DIP2A-004 |
| chr19 | 47287778 | 47289611 | 1834 | 12 | 9.41e-02 | -0.035 | -0.01 | SLC1A5-005, SLC1A5-004, SLC1A5-007, SLC1A5-003, SLC1A5-006 |
| chr19 | 46525566 | 46527546 | 1981 | 18 | 9.66e-02 | 0.047 | 0.012 | PGLYRP1-001 |
| chr19 | 35981224 | 35982442 | 1219 | 10 | 9.96e-02 | 0.028 | 0.004 | KRTDAP-001, KRTDAP-003 |
| chr5 | 59064235 | 59064682 | 448 | 11 | 1.02e-01 | -0.044 | -0.012 | PDE4D-013, PDE4D-011, PDE4D-012 |
| chr17 | 74523424 | 74525066 | 1643 | 12 | 1.07e-01 | -0.039 | -0.014 | PRCD-001, CYGB-004, PRCD-005 |
| chr21 | 45749343 | 45751350 | 2008 | 12 | 1.10e-01 | 0.038 | 0.003 | AP001062.1-201, AP001062.8-002, AP001062.8-001, AP001062.7-001, C21orf2-006, C21orf2-007 |
| chr1 | 45272261 | 45273680 | 1420 | 11 | 1.11e-01 | -0.061 | -0.013 | TCTEX1D4-001, TCTEX1D4-201, BTBD19-008, BTBD19-009, BTBD19-201, BTBD19-202, BTBD19-006, BTBD19-001, PLK3-004 |
| chr2 | 66667039 | 66668012 | 974 | 12 | 1.11e-01 | -0.05 | -0.014 | MEIS1-010, MEIS1-006, MEIS1-AS2-001, AC092669.1-002, MEIS1-011, MEIS1-016, MEIS1-007 |
| chr12 | 96389066 | 96390366 | 1301 | 11 | 1.16e-01 | 0.075 | 0.018 | HAL-001, HAL-002, HAL-201, HAL-003, HAL-010, HAL-005, RP11-256L6.3-001, HAL-006 |
| chr14 | 24777708 | 24779959 | 2252 | 12 | 1.17e-01 | 0.047 | 0.011 | CIDEB-001, CIDEB-002, LTB4R-001, LTB4R2-001, LTB4R2-003, LTB4R2-002, LTB4R2-201, LTB4R-004, CIDEB-003, CIDEB-005, CIDEB-006 |
| chr18 | 44562019 | 44562918 | 900 | 11 | 1.18e-01 | -0.014 | -0.005 | TCEB3B-001 |
| chr6 | 33141152 | 33142317 | 1166 | 11 | 1.19e-01 | 0.051 | 0.003 |  |
| chr6 | 26225246 | 26226256 | 1011 | 13 | 1.19e-01 | 0.035 | 0.02 | HIST1H3E-001 |
| chr22 | 46449430 | 46450251 | 822 | 12 | 1.23e-01 | -0.038 | -0.018 | C22orf26-201, FLJ27365-003, C22orf26-001, RP6-109B7.3-001, RP6-109B7.3-003, RP6-109B7.3-002, RP6-109B7.5-001 |
| chr17 | 77924582 | 77925938 | 1357 | 10 | 1.23e-01 | 0.03 | 0.012 | TBC1D16-003, TBC1D16-008, TBC1D16-004, TBC1D16-006, TBC1D16-009, TBC1D16-007, TBC1D16-002 |
| chr13 | 31505976 | 31507578 | 1603 | 14 | 1.24e-01 | 0.024 | 0.009 | TEX26-001, TEX26-AS1-001, TEX26-AS1-003, TEX26-AS1-013, TEX26-002, TEX26-005, TEX26-AS1-006, TEX26-AS1-002, TEX26-AS1-014, TEX26-AS1-012, TEX26-AS1-009, TEX26-AS1-004, TEX26-AS1-010, TEX26-AS1-005, TEX26-AS1-017, TEX26-AS1-016, TEX26-AS1-008, TEX26-AS1-007, TEX26-AS1-011, TEX26-AS1-015 |
| chr6 | 30610592 | 30612330 | 1739 | 12 | 1.28e-01 | 0.088 | 0.014 | ATAT1-009 |
| chr11 | 47470768 | 47471789 | 1022 | 10 | 1.29e-01 | 0.074 | 0.013 | RAPSN-001, RAPSN-002, RAPSN-004, RAPSN-003 |
| chr4 | 683046 | 684653 | 1608 | 12 | 1.29e-01 | 0.054 | 0.006 | MFSD7-001, MFSD7-003, MFSD7-004, MFSD7-005, MFSD7-007, MFSD7-009, MFSD7-008, MFSD7-002 |
| chr13 | 97873877 | 97874638 | 762 | 10 | 1.30e-01 | -0.054 | -0.012 | MBNL2-201, MBNL2-001, MBNL2-202, MBNL2-004, MBNL2-002 |
| chr1 | 1.47E+08 | 1.47E+08 | 553 | 10 | 1.30e-01 | -0.036 | -0.01 | BCL9-001 |
| chr3 | 1.96E+08 | 1.96E+08 | 583 | 11 | 1.34e-01 | -0.039 | -0.013 | TM4SF19-001, TM4SF19-001, TM4SF19-002, TM4SF19-004 |
| chr6 | 30652688 | 30653799 | 1112 | 15 | 1.36e-01 | -0.054 | -0.013 | PPP1R18-001, PPP1R18-002, PPP1R18-004, PPP1R18-003 |
| chr6 | 1.71E+08 | 1.71E+08 | 3606 | 21 | 1.37e-01 | 0.128 | 0.018 |  |
| chr7 | 1.23E+08 | 1.23E+08 | 1145 | 10 | 1.39e-01 | -0.03 | -0.013 | RP11-390E23.3-002, RP11-390E23.3-005, RP11-390E23.3-003, RP11-390E23.3-001, RP11-390E23.3-004 |
| chr12 | 12222913 | 12224457 | 1545 | 10 | 1.41e-01 | -0.044 | -0.014 | BCL2L14-003, BCL2L14-001, BCL2L14-012, BCL2L14-201, BCL2L14-006, BCL2L14-007 |
| chr14 | 1.01E+08 | 1.01E+08 | 1731 | 15 | 1.46e-01 | -0.023 | -0.009 | MIR380-201, MIR323A-201, MIR411-201, MIR299-201, MIR329-1-201, MIR329-2-201, MIR758-201, MIR1197-201 |
| chr2 | 2.42E+08 | 2.42E+08 | 1219 | 11 | 1.47e-01 | 0.034 | -0.002 | AGXT-001, AGXT-003, AGXT-004 |
| chr4 | 16085367 | 16086292 | 926 | 13 | 1.50e-01 | -0.066 | -0.013 | PROM1-008, PROM1-001, PROM1-003, PROM1-010, PROM1-009, PROM1-004, PROM1-011, PROM1-204 |
| chr3 | 1.11E+08 | 1.11E+08 | 1102 | 10 | 1.51e-01 | -0.047 | -0.013 | CD96-001, CD96-002, CD96-003, CD96-005, CD96-004 |
| chr12 | 95945082 | 95945927 | 846 | 11 | 1.53e-01 | 0.028 | 0.015 | USP44-001 |
| chr12 | 7032793 | 7033911 | 1119 | 12 | 1.60e-01 | 0.056 | 0.007 | ATN1-002, ENO2-015 |
| chr10 | 1.04E+08 | 1.04E+08 | 1070 | 11 | 1.60e-01 | 0.033 | 0.011 | C10orf95-002, RP11-18I14.10-001, RP11-18I14.10-002, RP11-18I14.10-005, RP11-18I14.10-007, RP11-18I14.10-006, RP11-18I14.10-008, RP11-18I14.10-004, RP11-18I14.10-003 |
| chr22 | 38713874 | 38714771 | 898 | 11 | 1.65e-01 | 0.022 | 0.006 | CSNK1E-003, CSNK1E-201, CSNK1E-002, CSNK1E-005, CSNK1E-004, CSNK1E-008 |
| chr1 | 64058180 | 64058941 | 762 | 10 | 1.69e-01 | 0.054 | 0.015 | PGM1-001, PGM1-003, ITGB3BP-009, PGM1-201 |
| chr16 | 57405979 | 57406955 | 977 | 11 | 1.78e-01 | 0.034 | 0.007 | CX3CL1-001, CX3CL1-002, CX3CL1-004 |
| chr12 | 27395963 | 27397422 | 1460 | 14 | 1.83e-01 | 0.066 | 0.009 | STK38L-001, STK38L-010, STK38L-201, STK38L-011, STK38L-007, STK38L-004, STK38L-005, STK38L-009, STK38L-012, STK38L-008 |
| chr1 | 1.78E+08 | 1.78E+08 | 522 | 10 | 1.87e-01 | -0.019 | -0.006 | TEX35-201, TEX35-001, TEX35-004, TEX35-002, TEX35-003, TEX35-005, TEX35-007 |
| chr11 | 72928926 | 72929844 | 919 | 13 | 1.91e-01 | 0.058 | 0.006 | P2RY2-001, P2RY2-003, P2RY2-002, RP11-800A3.2-001 |
| chr12 | 66275865 | 66276974 | 1110 | 11 | 2.08e-01 | 0.062 | 0.01 | RP11-366L20.2-001, RP11-366L20.2-004, RP11-366L20.2-003, RP11-366L20.2-002 |
| chr15 | 78555966 | 78556945 | 980 | 12 | 2.13e-01 | 0.035 | 0.009 | DNAJA4-003, DNAJA4-002, DNAJA4-001, DNAJA4-004, DNAJA4-012, DNAJA4-006, DNAJA4-011, DNAJA4-005, DNAJA4-007, RP11-762H8.3-001, RP11-762H8.3-002 |
| chr11 | 68919873 | 68921264 | 1392 | 10 | 2.17e-01 | 0.044 | 0.016 |  |
| chr17 | 75470567 | 75472168 | 1602 | 12 | 2.18e-01 | -0.028 | -0.01 | SEPT9-015, RP11-75C10.9-001 |
| chr17 | 40345445 | 40346798 | 1354 | 12 | 2.21e-01 | 0.027 | -0.003 | GHDC-006, GHDC-002, GHDC-201, GHDC-202, GHDC-010, GHDC-011, GHDC-001, GHDC-008, GHDC-003, GHDC-007, GHDC-009, GHDC-005 |
| chr16 | 89167018 | 89169289 | 2272 | 11 | 2.29e-01 | 0.081 | 0.02 | ACSF3-015, ACSF3-006, ACSF3-014, ACSF3-012 |
| chr10 | 86087566 | 86088270 | 705 | 10 | 2.30e-01 | 0.053 | 0.004 | CCSER2-002, CCSER2-001, CCSER2-201 |
| chr6 | 32013699 | 32014381 | 683 | 12 | 2.30e-01 | 0.057 | 0.003 | TNXB-006, TNXB-005 |
| chr9 | 1.24E+08 | 1.24E+08 | 1071 | 11 | 2.32e-01 | 0.086 | 0.013 | GSN-201, GSN-202, GSN-203, GSN-204, GSN-002, GSN-205, GSN-206, GSN-003, GSN-007 |
| chr3 | 99978949 | 99979976 | 1028 | 18 | 2.32e-01 | 0.041 | 0.008 | TBC1D23-001, TBC1D23-002, TBC1D23-008, TBC1D23-005 |
| chr8 | 1.24E+08 | 1.24E+08 | 1619 | 19 | 2.39e-01 | -0.033 | -0.012 | FAM83A-002, FAM83A-201, U3.48-201, FAM83A-004, RP11-539E17.5-001, FAM83A-003, FAM83A-202, FAM83A-203 |
| chr11 | 2170779 | 2172168 | 1390 | 12 | 2.53e-01 | -0.042 | -0.013 | IGF2-201 |
| chr6 | 30623775 | 30624769 | 995 | 14 | 2.56e-01 | 0.039 | 0.009 |  |
| chr17 | 6898315 | 6900799 | 2485 | 23 | 2.61e-01 | 0.049 | 0.009 | ALOX12-001, ALOX12-003, RP11-589P10.5-001 |
| chr6 | 31744339 | 31745181 | 843 | 13 | 2.70e-01 | -0.03 | -0.01 | Y_RNA.294-201, VWA7-201, VWA7-001, VWA7-202, VWA7-002, VARS-014, VWA7-003 |
| chr4 | 39481691 | 39483194 | 1504 | 13 | 2.77e-01 | 0.088 | 0.02 | RP11-472B18.1-001, RP11-472B18.1-003, RP11-472B18.1-004 |
| chr6 | 30297174 | 30297941 | 768 | 10 | 3.00e-01 | 0.065 | 0.023 | TRIM39-002, TRIM39-009, TRIM39-008, TRIM39-RPP21-001 |
| chr1 | 1.55E+08 | 1.55E+08 | 1878 | 11 | 3.07e-01 | -0.053 | -0.01 | MUC1-007, MUC1-201, MUC1-009, MUC1-008, MUC1-005, MUC1-014, MUC1-002, MUC1-020, MUC1-001, MUC1-006, MUC1-013, MUC1-003, MUC1-202, MUC1-004, MUC1-017, MUC1-015, MUC1-011, MUC1-019, MUC1-012, MUC1-018, MUC1-016, MUC1-010, MIR92B-201 |
| chr13 | 1.14E+08 | 1.14E+08 | 1602 | 15 | 3.11e-01 | -0.066 | -0.017 | MCF2L-202, MCF2L-002, MCF2L-AS1-001, MCF2L-005, RP11-120K24.3-001 |
| chr10 | 63807168 | 63809170 | 2003 | 17 | 3.11e-01 | -0.047 | -0.011 | ARID5B-002 |
| chr20 | 36156919 | 36157675 | 757 | 10 | 3.18e-01 | 0.08 | 0.014 | BLCAP-001, BLCAP-007, BLCAP-003, BLCAP-006, BLCAP-201, BLCAP-004, PPIAP3-001, BLCAP-002 |
| chr17 | 17721180 | 17722624 | 1445 | 12 | 3.22e-01 | 0.022 | -0.003 | SREBF1-018, SREBF1-005, SREBF1-004, SREBF1-012, SREBF1-010, SREBF1-007, SREBF1-011, SREBF1-009, SREBF1-013, SREBF1-017, SREBF1-019, SREBF1-025, SREBF1-023 |
| chr19 | 46999224 | 46999840 | 617 | 10 | 3.24e-01 | 0.043 | 0.01 | AC011484.1-201, PNMAL2-201, PNMAL2-004, PNMAL2-001 |
| chr6 | 30458048 | 30459867 | 1820 | 24 | 3.25e-01 | -0.043 | -0.011 | HLA-E-001, HLA-E-002, HLA-E-003 |
| chr9 | 91604473 | 91606223 | 1751 | 16 | 3.37e-01 | 0.05 | 0.012 | C9orf47-003, S1PR3-002, C9orf47-002, C9orf47-001 |
| chr6 | 33218604 | 33220041 | 1438 | 13 | 3.39e-01 | -0.044 | -0.01 | HCG25-003, HCG25-002, HCG25-004, HCG25-001 |
| chr16 | 68056688 | 68057225 | 538 | 15 | 3.40e-01 | 0.014 | 0.001 | DDX28-001, DUS2-003, DUS2-002, DUS2-007, DUS2-009, DUS2-001, DUS2-005, DUS2-012, DUS2-006 |
| chr3 | 42699933 | 42701042 | 1110 | 11 | 3.40e-01 | 0.064 | 0.016 | ZBTB47-003 |
| chr19 | 56056468 | 56057386 | 919 | 12 | 3.43e-01 | -0.016 | -0.007 | SBK3-001, SBK3-003 |
| chr11 | 57364100 | 57365701 | 1602 | 10 | 3.46e-01 | 0.038 | 0.01 | SERPING1-001, SERPING1-006, SERPING1-005, SERPING1-007, SERPING1-002, SERPING1-004, SERPING1-003, SERPING1-008, SERPING1-009 |
| chr8 | 1.34E+08 | 1.34E+08 | 828 | 11 | 3.51e-01 | -0.044 | -0.007 | SLA-003, SLA-001, SLA-004, SLA-014, SLA-002, SLA-015, SLA-018, SLA-016, SLA-017, SLA-019, SLA-013, SLA-012, SLA-011, SLA-005 |
| chr14 | 1.02E+08 | 1.02E+08 | 929 | 12 | 3.52e-01 | -0.028 | -0.01 | MIR654-201, MIR300-201, AL132709.2-201, MIR376A1-201, MIR376C-201 |
| chr19 | 4909175 | 4909600 | 426 | 11 | 3.70e-01 | 0.015 | 0.003 | UHRF1-201, UHRF1-202, UHRF1-203, UHRF1-005, UHRF1-002, UHRF1-003, UHRF1-001, UHRF1-004 |
| chr7 | 50799978 | 50801000 | 1023 | 10 | 3.70e-01 | 0.016 | 0.006 | GRB10-005, GRB10-203 |
| chr7 | 1.42E+08 | 1.42E+08 | 1100 | 15 | 3.80e-01 | -0.03 | -0.012 | TRBJ2-1-001, TRBJ2-2-001, TRBJ2-2P-001, TRBJ2-3-001, TRBJ2-4-001, TRBJ2-5-001, TRBJ2-6-001, TRBJ2-7-001 |
| chr12 | 1.17E+08 | 1.17E+08 | 827 | 11 | 3.82e-01 | 0.027 | -0.002 | MAP1LC3B2-201 |
| chr1 | 45243032 | 45243960 | 929 | 10 | 3.85e-01 | 0.084 | 0.01 | SNORD46-201, SNORD38A-201, RPS8-003, SNORD38B-201, RPS8-001, RP11-269F19.2-002, RPS8-006, RPS8-005, RPS8-008, RPS8-007, RPS8-004, SNORD55-201 |
| chr11 | 69259247 | 69261044 | 1798 | 10 | 3.90e-01 | 0.038 | 0.009 | AP000439.2-001 |
| chr13 | 1.13E+08 | 1.13E+08 | 1107 | 11 | 3.94e-01 | 0.035 | 0.008 |  |
| chr20 | 33145456 | 33146543 | 1088 | 11 | 4.01e-01 | -0.041 | -0.01 | MAP1LC3A-002, MAP1LC3A-003, MAP1LC3A-004 |
| chr17 | 80545020 | 80545869 | 850 | 12 | 4.07e-01 | 0.063 | 0.012 | RP13-638C3.3-001, FOXK2-011 |
| chr12 | 57587422 | 57588350 | 929 | 11 | 4.14e-01 | 0.033 | -0.004 | MIR1228-201 |
| chr6 | 31645405 | 31646834 | 1430 | 12 | 4.15e-01 | 0.037 | 0.006 | LY6G5C-202, LY6G5C-003, LY6G5C-005, LY6G5C-004 |
| chr2 | 25141696 | 25142523 | 828 | 12 | 4.18e-01 | -0.048 | -0.012 | ADCY3-001, ADCY3-002, ADCY3-004 |
| chr19 | 35628294 | 35630651 | 2358 | 20 | 4.29e-01 | 0.057 | 0.009 | FXYD1-001, AC020907.2-201, FXYD1-006, CTD-2527I21.4-002, FXYD1-004, FXYD1-007, FXYD1-003, FXYD1-002, FXYD1-009, FXYD1-005, FXYD1-010, CTD-2527I21.4-001, FXYD1-008 |
| chr7 | 1.51E+08 | 1.51E+08 | 1324 | 17 | 4.63e-01 | 0.06 | 0.007 | ASB10-002, ASB10-003, ASB10-004, ASB10-001, ASB10-201, ASB10-202 |
| chr6 | 42988445 | 42989367 | 923 | 10 | 4.71e-01 | 0.032 | 0.006 | RRP36-001 |
| chr3 | 71179796 | 71180818 | 1023 | 12 | 4.72e-01 | -0.04 | -0.01 | FOXP1-011, FOXP1-009 |
| chr8 | 1.45E+08 | 1.45E+08 | 1146 | 12 | 4.86e-01 | 0.038 | 0.009 | PLEC-008 |
| chr11 | 1891295 | 1892307 | 1013 | 14 | 4.98e-01 | -0.042 | -0.012 | LSP1-005, LSP1-002, LSP1-008, LSP1-016, LSP1-015, LSP1-006, LSP1-007, LSP1-009 |
| chr2 | 1.57E+08 | 1.57E+08 | 953 | 10 | 5.03e-01 | 0.049 | 0.009 | GPD2-001, GPD2-201, GPD2-008, GPD2-002, GPD2-007, GPD2-202 |
| chr13 | 20532610 | 20533018 | 409 | 11 | 5.16e-01 | -0.007 | -0.001 | ZMYM2-201, ZMYM2-005, ZMYM2-003, ZMYM2-004 |
| chr1 | 2.01E+08 | 2.01E+08 | 758 | 17 | 5.18e-01 | -0.026 | -0.008 | CSRP1-201, CSRP1-001, RP11-134G8.7-001, CSRP1-005, CSRP1-003, CSRP1-002, CSRP1-012, CSRP1-015, CSRP1-014, CSRP1-013, CSRP1-017, CSRP1-016, CSRP1-011, CSRP1-018, CSRP1-010, CSRP1-006 |
| chr16 | 1629409 | 1630880 | 1472 | 10 | 5.25e-01 | 0.074 | 0.013 | LA16c-395F10.2-001, LA16c-425C2.1-001 |
| chr10 | 1.02E+08 | 1.02E+08 | 1222 | 15 | 5.33e-01 | 0.024 | 0.005 | SEC31B-201, SEC31B-003, SEC31B-202, SEC31B-004, SEC31B-009, SEC31B-001, SEC31B-010 |
| chr7 | 2556531 | 2557466 | 936 | 10 | 5.47e-01 | 0.042 | 0.005 | LFNG-201, LFNG-004 |
| chr1 | 1.55E+08 | 1.55E+08 | 1405 | 14 | 5.55e-01 | 0.044 | 0.006 | SLC50A1-003, SLC50A1-002, SLC50A1-001, SLC50A1-008, SLC50A1-010, SLC50A1-009, SLC50A1-012, SLC50A1-005, SLC50A1-006, SLC50A1-004, SLC50A1-007, SLC50A1-013 |
| chr1 | 1.61E+08 | 1.61E+08 | 1502 | 12 | 5.60e-01 | 0.063 | 0.011 | NDUFS2-001, ADAMTS4-002, ADAMTS4-001, NDUFS2-002, NDUFS2-003 |
| chr17 | 46622012 | 46622899 | 888 | 13 | 5.62e-01 | -0.053 | -0.016 | HOXB2-001, HOXB-AS1-001, HOXB-AS1-004, HOXB2-003, HOXB-AS1-002, HOXB-AS1-003, HOXB2-002, HOXB2-004 |
| chr20 | 57582213 | 57583709 | 1497 | 25 | 5.72e-01 | 0.028 | 0.002 | CTSZ-001, CTSZ-003, CTSZ-002, CTSZ-005 |
| chrX | 1.54E+08 | 1.54E+08 | 901 | 16 | 5.99e-01 | 0.066 | 0.019 | TKTL1-201, TEX28-002, TKTL1-002, TEX28-001, TEX28-003, TKTL1-009 |
| chr7 | 1.39E+08 | 1.39E+08 | 2121 | 13 | 6.04e-01 | 0.107 | 0.009 | KIAA1549-201 |
| chr6 | 30038254 | 30038975 | 722 | 14 | 6.16e-01 | 0.039 | 0.004 |  |
| chr10 | 77541834 | 77542585 | 752 | 10 | 6.28e-01 | 0.054 | 0.001 | C10orf11-001 |
| chr13 | 1.15E+08 | 1.15E+08 | 1321 | 12 | 6.30e-01 | 0.057 | 0.014 |  |
| chr6 | 32119874 | 32121611 | 1738 | 42 | 6.32e-01 | 0.053 | 0.016 | PRRT1-002, PPT2-207, PPT2-001, PPT2-003, PPT2-004, PRRT1-001, PRRT1-201, PPT2-002, PPT2-007, PPT2-EGFL8-002, PPT2-009, PPT2-EGFL8-001, PRRT1-005, PPT2-012, PPT2-208, PPT2-209, PPT2-EGFL8-006, PPT2-013, PRRT1-006, PRRT1-009, PPT2-008, PRRT1-012, PRRT1-010, PPT2-010, PRRT1-008, PRRT1-003, PRRT1-011, PRRT1-004, PPT2-EGFL8-005, PPT2-EGFL8-004 |
| chr14 | 1.02E+08 | 1.02E+08 | 1332 | 10 | 6.40e-01 | -0.015 | -0.006 | MIR487A-201, MIR382-201, MIR134-201, MIR323B-201, MIR485-201, AL132709.3-201 |
| chr6 | 31777609 | 31778942 | 1334 | 21 | 6.53e-01 | -0.024 | -0.006 |  |
| chr5 | 1498266 | 1499797 | 1532 | 13 | 6.79e-01 | 0.068 | 0.013 |  |
| chr20 | 3051493 | 3052483 | 991 | 12 | 6.86e-01 | -0.029 | -0.009 | OXT-001 |
| chr21 | 46340810 | 46341918 | 1109 | 11 | 7.18e-01 | 0.066 | 0.014 | ITGB2-201, ITGB2-008, ITGB2-003, ITGB2-AS1-002, ITGB2-AS1-001, ITGB2-007, ITGB2-016, ITGB2-019, ITGB2-020, ITGB2-018, ITGB2-017, ITGB2-013, ITGB2-021, ITGB2-AS1-004, ITGB2-AS1-005, ITGB2-AS1-003, ITGB2-AS1-006 |
| chr14 | 35514911 | 35515417 | 507 | 11 | 7.21e-01 | 0.029 | 0.002 | FAM177A1-001, FAM177A1-010, FAM177A1-201, FAM177A1-004, FAM177A1-005, FAM177A1-003, FAM177A1-002, FAM177A1-006 |
| chr22 | 50585161 | 50586318 | 1158 | 12 | 7.21e-01 | 0.026 | 0.006 | MOV10L1-006, MOV10L1-005 |
| chr6 | 33288599 | 33289719 | 1121 | 28 | 7.61e-01 | 0.037 | 0.005 | DAXX-002, DAXX-001, DAXX-201, DAXX-007, DAXX-011, DAXX-009, DAXX-010, DAXX-004, DAXX-008, DAXX-003 |
| chr1 | 2.06E+08 | 2.06E+08 | 1453 | 10 | 7.66e-01 | -0.044 | -0.006 | SLC41A1-001, SLC41A1-004 |
| chr6 | 31831681 | 31833747 | 2067 | 27 | 7.85e-01 | 0.034 | -0.001 | NEU1-001, NEU1-004, SLC44A4-007, NEU1-002, NEU1-005 |
| chr11 | 13983705 | 13984077 | 373 | 12 | 7.98e-01 | 0.032 | 0.013 | SPON1-201, SPON1-001 |
| chr6 | 32078127 | 32078624 | 498 | 10 | 8.02e-01 | -0.021 | -0.005 | TNXB-001, TNXB-201, TNXB-007, TNXB-002 |
| chr13 | 1.14E+08 | 1.14E+08 | 1261 | 14 | 8.16e-01 | -0.056 | -0.008 | MCF2L-003, MCF2L-018, MCF2L-030, MCF2L-203, MCF2L-031 |
| chr6 | 30690213 | 30691305 | 1093 | 15 | 8.19e-01 | -0.044 | -0.009 | TUBB-002, TUBB-004, TUBB-003, XXbac-BPG252P9.9-001 |
| chr22 | 46480891 | 46482023 | 1133 | 14 | 8.47e-01 | -0.041 | -0.008 | FLJ27365-001, FLJ27365-002 |
| chr12 | 53730072 | 53730421 | 350 | 10 | 8.48e-01 | 0.046 | 0.012 | SP7-002, SP7-001, SP7-003 |
| chr3 | 48471218 | 48471995 | 778 | 10 | 8.65e-01 | -0.046 | -0.006 | PLXNB1-001, PLXNB1-201, PLXNB1-003, PLXNB1-202, PLXNB1-019 |
| chr6 | 31867698 | 31868847 | 1150 | 34 | 8.67e-01 | 0.072 | 0.015 | ZBTB12-001, C2-015, C2-014 |
| chr6 | 1.61E+08 | 1.61E+08 | 481 | 11 | 8.72e-01 | -0.025 | -0.009 | SLC22A2-001, SLC22A2-005, SLC22A2-004 |
| chr17 | 19648846 | 19649293 | 448 | 10 | 9.09e-01 | 0.052 | 0.004 | ALDH3A1-018, ALDH3A1-009, ALDH3A1-201, ALDH3A1-010, ALDH3A1-016, ALDH3A1-015, ALDH3A1-017 |
| chr11 | 2397977 | 2398533 | 557 | 14 | 9.29e-01 | -0.008 | -0.002 | CD81-001, CD81-AS1-002, CD81-AS1-001, CD81-002, CD81-012, CD81-013 |
| chr3 | 15374148 | 15375047 | 900 | 15 | 9.55e-01 | 0.036 | 0.003 | SH3BP5-201, SH3BP5-001, SH3BP5-003, SH3BP5-002, SH3BP5-005, SH3BP5-006 |
| chr11 | 2890389 | 2891118 | 730 | 26 | 9.55e-01 | -0.027 | -0.006 | KCNQ1DN-001 |
| chr6 | 31860013 | 31860832 | 820 | 16 | 9.86e-01 | 0.051 | 0.003 | EHMT2-010 |
| chr6 | 32812518 | 32813715 | 1198 | 25 | 9.94e-01 | -0.075 | -0.005 | PSMB8-001, PSMB8-002, PSMB9-005, PSMB8-004, TAPSAR1-005, TAPSAR1-004, PSMB9-006, TAPSAR1-006, TAPSAR1-003, TAPSAR1-001, TAPSAR1-002, PSMB8-003 |
| chr6 | 32819858 | 32823116 | 3259 | 68 | 9.96e-01 | 0.057 | -0.005 | TAP1-001, PSMB9-001, TAP1-201, PSMB9-202, PSMB9-002, PSMB9-004, TAP1-002 |

**Supplemental Table S2. Differentially Methylated Regions in Control Group between Day 1 and 14**

| **Chr** | **Start** | **End** | **Width** | **Num CpGs** | **p-value** | **Max Prop Change** | **Mean Prop Change** | **Genes** |
| --- | --- | --- | --- | --- | --- | --- | --- | --- |
| chr17 | 71258045 | 71259281 | 1237 | 11 | 1.84e-23 | 0.121 | 0.065 | CPSF4L-002, CPSF4L-001 |
| chr14 | 1.06E+08 | 1.06E+08 | 2920 | 20 | 1.51e-22 | -0.062 | -0.038 | IGHJ6-001, IGHJ2-001, IGHJ1-001, IGHJ3P-001, IGHD7-27-001, IGHJ1P-001, IGHJ2P-001, IGHJ4-001, IGHJ3-001, IGHJ5-001 |
| chr11 | 2011216 | 2012411 | 1196 | 12 | 7.70e-21 | 0.119 | 0.025 | AC051649.6-001, MRPL23-AS1-001 |
| chr17 | 79004850 | 79007529 | 2680 | 16 | 1.11e-19 | 0.132 | 0.049 | BAIAP2-002, BAIAP2-003, BAIAP2-201, BAIAP2-001, BAIAP2-007, BAIAP2-AS1-201, BAIAP2-019, BAIAP2-011, BAIAP2-AS1-002, BAIAP2-024, BAIAP2-009, BAIAP2-004, BAIAP2-023, BAIAP2-014, BAIAP2-022, BAIAP2-AS1-001 |
| chr1 | 26644234 | 26646309 | 2076 | 13 | 7.47e-16 | -0.055 | -0.036 | CD52-001, UBXN11-201, UBXN11-001, CD52-002, CD52-003 |
| chr1 | 2.49E+08 | 2.49E+08 | 1603 | 11 | 7.23e-13 | 0.13 | 0.066 | LYPD8-001 |
| chr17 | 80192161 | 80197360 | 5200 | 27 | 2.48e-12 | 0.084 | 0.022 | SLC16A3-008, SLC16A3-017, SLC16A3-009, SLC16A3-001, SLC16A3-010, SLC16A3-018 |
| chr16 | 1493859 | 1496312 | 2454 | 15 | 4.19e-12 | 0.107 | 0.038 | CCDC154-201, CCDC154-001, LA16c-390E6.5-002 |
| chr2 | 7016367 | 7018885 | 2519 | 13 | 1.80e-11 | -0.092 | -0.037 | RSAD2-001, RSAD2-002, RSAD2-005, RSAD2-201 |
| chr2 | 33359059 | 33359688 | 630 | 11 | 3.84e-11 | 0.12 | 0.061 | LTBP1-203, LTBP1-008, LTBP1-001, LTBP1-007, LTBP1-204, LTBP1-016 |
| chr16 | 87099124 | 87102691 | 3568 | 19 | 9.82e-11 | 0.209 | 0.045 | RP11-134D3.2-001 |
| chr11 | 73356316 | 73358107 | 1792 | 15 | 1.74e-09 | 0.051 | 0.024 | PLEKHB1-003, PLEKHB1-001, PLEKHB1-002, PLEKHB1-004, PLEKHB1-007, PLEKHB1-013, PLEKHB1-009, PLEKHB1-016, PLEKHB1-015, PLEKHB1-023, PLEKHB1-019, PLEKHB1-017, PLEKHB1-020, PLEKHB1-010, PLEKHB1-018, PLEKHB1-011, PLEKHB1-012, PLEKHB1-014 |
| chr7 | 1022098 | 1023156 | 1059 | 12 | 4.20e-09 | 0.05 | 0.02 | CYP2W1-001, CYP2W1-002 |
| chr17 | 80200634 | 80203277 | 2644 | 13 | 9.88e-09 | 0.236 | 0.061 | CSNK1D-028, CSNK1D-029 |
| chr5 | 78985425 | 78986160 | 736 | 11 | 1.28e-08 | 0.061 | 0.027 | CMYA5-001 |
| chr22 | 17955274 | 17956641 | 1368 | 10 | 1.50e-08 | 0.081 | 0.031 | CECR2-201, CECR2-001 |
| chr5 | 58652602 | 58654193 | 1592 | 11 | 1.64e-08 | 0.062 | 0.032 | PDE4D-015 |
| chr17 | 79798629 | 79801920 | 3292 | 18 | 1.91e-08 | 0.135 | 0.034 | P4HB-012, P4HB-030, RP11-498C9.2-001 |
| chr16 | 1537064 | 1539318 | 2255 | 13 | 2.34e-08 | 0.067 | 0.031 | PTX4-201, PTX4-002, PTX4-001 |
| chr14 | 1.06E+08 | 1.06E+08 | 3953 | 14 | 2.35e-08 | -0.069 | -0.028 | IGHD5-18-001, IGHD3-16-001, IGHD2-15-001, AL122127.25-002, IGHD4-17-001 |
| chr10 | 21796152 | 21799143 | 2992 | 13 | 2.39e-08 | 0.085 | 0.035 |  |
| chr6 | 31542556 | 31543686 | 1131 | 15 | 2.42e-08 | 0.082 | 0.023 | TNF-001 |
| chr15 | 68497730 | 68499369 | 1640 | 12 | 1.75e-07 | 0.056 | 0.022 | CALML4-005, CALML4-002, CALML4-003, CALML4-001, CALML4-004, CALML4-201 |
| chr15 | 59156878 | 59158587 | 1710 | 10 | 2.35e-07 | 0.096 | 0.031 | RP11-30K9.5-001, ZNF444P1-001, RNF111-007 |
| chr7 | 921845 | 925664 | 3820 | 17 | 2.48e-07 | 0.102 | 0.029 | GET4-002, GET4-004, GET4-003, GET4-007, RP11-449P15.2-001 |
| chr17 | 57915665 | 57918682 | 3018 | 12 | 2.80e-07 | -0.086 | -0.031 | MIR21-201, VMP1-012, VMP1-013, VMP1-016, VMP1-004 |
| chr11 | 63302736 | 63304963 | 2228 | 10 | 4.41e-07 | -0.073 | -0.027 | RARRES3-001, RARRES3-201, RARRES3-002, RARRES3-003, RARRES3-004 |
| chr1 | 1266178 | 1267559 | 1382 | 13 | 4.86e-07 | 0.073 | 0.027 | TAS1R3-001 |
| chr5 | 76247647 | 76249776 | 2130 | 13 | 6.62e-07 | -0.102 | -0.034 | CRHBP-001, CRHBP-002, CRHBP-003 |
| chr8 | 1.45E+08 | 1.45E+08 | 2367 | 10 | 9.60e-07 | 0.087 | 0.03 | SCRIB-001, SCRIB-002, SCRIB-003, MIR937-201 |
| chr3 | 46718369 | 46719834 | 1466 | 10 | 1.09e-06 | 0.088 | 0.028 | ALS2CL-008, ALS2CL-007 |
| chr12 | 14996143 | 14997216 | 1074 | 12 | 1.53e-06 | 0.12 | 0.025 | ART4-001, ART4-002, ART4-003, ART4-004 |
| chr6 | 1.33E+08 | 1.33E+08 | 1319 | 10 | 1.73e-06 | -0.04 | -0.022 | RPS12-001, SNORA33-201, SNORD101-201, SNORD100-201, RPS12-002 |
| chr18 | 56296094 | 56296607 | 514 | 10 | 2.01e-06 | 0.111 | 0.036 | ALPK2-001, RPL9P31-001 |
| chr6 | 10555114 | 10556523 | 1410 | 13 | 2.20e-06 | 0.081 | 0.036 | GCNT2-001, GCNT2-016 |
| chr20 | 30406052 | 30407388 | 1337 | 13 | 9.11e-06 | 0.052 | 0.015 | MYLK2-001, MYLK2-002 |
| chr8 | 1893977 | 1897697 | 3721 | 13 | 1.23e-05 | 0.049 | 0.021 |  |
| chr9 | 1.13E+08 | 1.13E+08 | 3199 | 13 | 1.82e-05 | 0.056 | 0.016 | AKAP2-003, AKAP2-004 |
| chr8 | 1.29E+08 | 1.29E+08 | 3141 | 16 | 4.62e-05 | 0.064 | -0.007 | PVT1-201, PVT1-005, PVT1-006, PVT1-007, PVT1-009, PVT1-002, PVT1-008, PVT1-010, PVT1-003, PVT1-011, PVT1-001 |
| chr3 | 1.29E+08 | 1.29E+08 | 1027 | 10 | 4.83e-05 | 0.132 | 0.043 | GP9-001 |
| chr6 | 1.5E+08 | 1.5E+08 | 1064 | 12 | 4.92e-05 | -0.054 | -0.03 | ZC3H12D-201, ZC3H12D-003, ZC3H12D-002, ZC3H12D-202, ZC3H12D-004, ZC3H12D-203 |
| chr2 | 66671478 | 66673985 | 2508 | 15 | 5.85e-05 | -0.052 | -0.026 | MEIS1-012, MEIS1-007, MEIS1-018 |
| chr2 | 1.14E+08 | 1.14E+08 | 2963 | 12 | 8.42e-05 | 0.051 | 0.009 | IL1A-001 |
| chr16 | 87736832 | 87740757 | 3926 | 16 | 1.05e-04 | 0.047 | 0.022 | FLJ00104-201, KLHDC4-026, KLHDC4-025 |
| chr13 | 78313933 | 78316492 | 2560 | 13 | 1.06e-04 | 0.088 | 0.026 | SLAIN1-201, SLAIN1-202, SLAIN1-001, SLAIN1-002, SLAIN1-011, SLAIN1-010, SLAIN1-013, SLAIN1-014, SLAIN1-012 |
| chr20 | 11897921 | 11900410 | 2490 | 11 | 1.13e-04 | -0.056 | -0.017 | BTBD3-201, BTBD3-008, RP4-742J24.2-001, BTBD3-007, BTBD3-009, BTBD3-012, BTBD3-011, BTBD3-010 |
| chr3 | 50359583 | 50361180 | 1598 | 11 | 1.19e-04 | 0.048 | 0.024 | HYAL2-001, HYAL2-002, HYAL2-009, HYAL2-008, HYAL2-010, HYAL2-006, HYAL2-004, HYAL2-003, HYAL2-007, HYAL2-005 |
| chr2 | 98329337 | 98330493 | 1157 | 10 | 1.34e-04 | -0.047 | -0.021 | ZAP70-001, ZAP70-002 |
| chr11 | 62620843 | 62622991 | 2149 | 20 | 1.45e-04 | -0.064 | -0.016 | SNHG1-201, SNHG1-202, SNHG1-203, SLC3A2-002, SLC3A2-001, SLC3A2-003, SLC3A2-201, SNHG1-204, SNHG1-205, SNHG1-206, SNHG1-207, SNHG1-208, SNHG1-209, SNHG1-022, SLC3A2-026, SNHG1-016, SNHG1-013, SNHG1-025, SNHG1-004, SNHG1-002, SNHG1-011, SNHG1-019, SNHG1-003, SNHG1-023, SNHG1-006, SNHG1-005, SNHG1-001, SNHG1-017, SNHG1-010, SNHG1-008, SNHG1-021, SNHG1-020, SNHG1-007, SNHG1-018, SNHG1-009, SNHG1-012, SNHG1-014, SNHG1-015, SNHG1-024 |
| chr2 | 85660497 | 85664005 | 3509 | 11 | 1.78e-04 | 0.05 | 0.02 | SH2D6-001, Y_RNA.546-201, SH2D6-005, SH2D6-006 |
| chr11 | 1296469 | 1298256 | 1788 | 10 | 1.88e-04 | 0.077 | 0.005 | AC136297.1-201 |
| chr16 | 57672219 | 57673993 | 1775 | 11 | 2.02e-04 | -0.046 | -0.021 | GPR56-009, GPR56-206, GPR56-032, GPR56-076, GPR56-015, GPR56-028, GPR56-016, GPR56-068, GPR56-066, GPR56-008, GPR56-067, GPR56-069, GPR56-045, GPR56-031, GPR56-017, GPR56-029, GPR56-013, GPR56-046 |
| chr6 | 32908239 | 32909523 | 1285 | 14 | 2.30e-04 | -0.06 | -0.025 | HLA-DMB-006, HLA-DMB-213, HLA-DMB-001, HLA-DMB-003 |
| chr7 | 1.35E+08 | 1.35E+08 | 1205 | 14 | 2.52e-04 | -0.06 | -0.023 | TMEM140-001, AC083862.1-201, C7orf49-013, TMEM140-002 |
| chr17 | 79030033 | 79031712 | 1680 | 12 | 2.62e-04 | 0.071 | 0.019 | BAIAP2-017, BAIAP2-016, BAIAP2-018, BAIAP2-030, BAIAP2-015 |
| chr11 | 64659044 | 64661321 | 2278 | 10 | 4.75e-04 | 0.046 | 0.017 | AP001187.1-201, MIR194-2-201, MIR192-201, MIR194-2-001, RP11-665N17.4-001 |
| chr11 | 94277826 | 94279068 | 1243 | 10 | 5.56e-04 | 0.052 | 0.026 | FUT4-001, PIWIL4-003, PIWIL4-002 |
| chr1 | 2.06E+08 | 2.06E+08 | 1772 | 11 | 5.64e-04 | 0.1 | 0.014 | CTSE-001, CTSE-002, CTSE-201, CTSE-202 |
| chr12 | 1.24E+08 | 1.24E+08 | 1922 | 12 | 5.85e-04 | 0.174 | 0.039 | CCDC92-009, CCDC92-008, CCDC92-002 |
| chr7 | 2756996 | 2759063 | 2068 | 10 | 6.36e-04 | -0.048 | -0.014 |  |
| chr2 | 1.57E+08 | 1.57E+08 | 1224 | 10 | 8.45e-04 | 0.028 | 0.02 | NR4A2-007 |
| chr12 | 6745057 | 6746030 | 974 | 14 | 9.49e-04 | 0.043 | 0.016 | LPAR5-001, LPAR5-002, ACRBP-004 |
| chr7 | 27208577 | 27211741 | 3165 | 23 | 1.04e-03 | -0.063 | -0.016 | MIR196B-201, HOXA9-004, HOXA9-005, HOXA9-002, HOXA10-006, HOXA10-AS-002, HOXA10-AS-003, HOXA10-005, HOXA10-AS-001, HOXA10-004 |
| chr16 | 30959315 | 30960328 | 1014 | 12 | 1.09e-03 | 0.063 | 0.019 | ORAI3-001, AC135048.13-001, ORAI3-005, ORAI3-003, AC135048.13-002, ORAI3-002 |
| chr17 | 80539144 | 80542340 | 3197 | 15 | 1.16e-03 | 0.112 | 0.043 | snoU13.58-201, FOXK2-010, FOXK2-011 |
| chr1 | 6418521 | 6419906 | 1386 | 10 | 1.20e-03 | -0.037 | -0.021 | ACOT7-005, ACOT7-004, ACOT7-201 |
| chr21 | 45704089 | 45706101 | 2013 | 17 | 1.48e-03 | 0.063 | 0.02 | AIRE-001, AIRE-004, AIRE-005 |
| chr16 | 2983147 | 2984768 | 1622 | 10 | 1.80e-03 | 0.05 | 0.016 | FLYWCH1-006, FLYWCH1-004, FLYWCH1-011, FLYWCH1-007, FLYWCH1-012 |
| chr20 | 748992 | 750226 | 1235 | 10 | 1.89e-03 | 0.036 | 0.006 | SLC52A3-001, SLC52A3-004, SLC52A3-003 |
| chr2 | 1.93E+08 | 1.93E+08 | 2573 | 13 | 1.92e-03 | -0.048 | -0.017 | SDPR-001, AC098617.1-003 |
| chr1 | 25256705 | 25258679 | 1975 | 26 | 2.25e-03 | 0.111 | 0.021 | RUNX3-001, RUNX3-004, RUNX3-202 |
| chr7 | 1.56E+08 | 1.56E+08 | 1937 | 11 | 2.31e-03 | 0.023 | 0 | LINC01006-006, LINC01006-003, LINC01006-004 |
| chr17 | 58498871 | 58500186 | 1316 | 13 | 2.55e-03 | 0.043 | 0.017 | C17orf64-001, C17orf64-003, C17orf64-002, USP32-014, USP32-015 |
| chr12 | 10182437 | 10184399 | 1963 | 12 | 2.56e-03 | 0.065 | 0.023 | CLEC9A-001, RP11-133L14.5-001, CLEC9A-002 |
| chr11 | 67809271 | 67811721 | 2451 | 15 | 2.70e-03 | 0.075 | 0.017 | TCIRG1-009, TCIRG1-013, TCIRG1-012, TCIRG1-014, TCIRG1-016, TCIRG1-004 |
| chr7 | 27196790 | 27198896 | 2107 | 12 | 3.07e-03 | -0.059 | -0.021 | HOXA7-001, HOXA7-002, HOXA7-003, RP1-170O19.21-001 |
| chr8 | 1.42E+08 | 1.42E+08 | 2805 | 10 | 3.76e-03 | 0.08 | 0.023 |  |
| chr6 | 30847733 | 30851940 | 4208 | 33 | 4.25e-03 | 0.049 | 0.016 | DDR1-005, DDR1-002, DDR1-003, DDR1-204, DDR1-205, DDR1-013, DDR1-031, DDR1-012, DDR1-016, DDR1-014, DDR1-021, DDR1-036, DDR1-206, DDR1-207, DDR1-001, DDR1-010, DDR1-011, DDR1-004, DDR1-025, DDR1-027, DDR1-046, DDR1-042, DDR1-037, DDR1-026, DDR1-047, DDR1-024, DDR1-033, DDR1-034, DDR1-032, DDR1-020, DDR1-028, DDR1-030, DDR1-035, DDR1-048, DDR1-039, DDR1-049, DDR1-038, DDR1-043, DDR1-045, DDR1-044, DDR1-040, DDR1-029, DDR1-041 |
| chr11 | 914329 | 916207 | 1879 | 14 | 4.74e-03 | -0.025 | 0 | CHID1-201, CHID1-203, CHID1-008, CHID1-007, CHID1-005, CHID1-006 |
| chr14 | 24539335 | 24540773 | 1439 | 14 | 4.90e-03 | 0.066 | 0.024 | CPNE6-201, CPNE6-003, CPNE6-002, CPNE6-202, CPNE6-017, CPNE6-014, CPNE6-018, CPNE6-005, CPNE6-011, CPNE6-004, CPNE6-019, CPNE6-013, CPNE6-016, CPNE6-008, CPNE6-015, CPNE6-010, CPNE6-009, CPNE6-012 |
| chr1 | 1.54E+08 | 1.54E+08 | 1476 | 12 | 5.73e-03 | 0.061 | 0.021 | SLC27A3-001, SLC27A3-201, SLC27A3-009, SLC27A3-008, SLC27A3-002, SLC27A3-010, SLC27A3-007 |
| chr19 | 46525566 | 46527546 | 1981 | 18 | 6.06e-03 | 0.07 | 0.016 | PGLYRP1-001 |
| chr8 | 27468684 | 27469673 | 990 | 10 | 6.10e-03 | -0.118 | -0.03 | CLU-004, CLU-011, CLU-016, CLU-017, CLU-003, CLU-201, CLU-002 |
| chr15 | 29212340 | 29213860 | 1521 | 10 | 6.30e-03 | -0.039 | -0.013 | APBA2-201, APBA2-202, APBA2-008 |
| chr13 | 43565399 | 43567153 | 1755 | 14 | 6.32e-03 | -0.051 | -0.012 | EPSTI1-001, EPSTI1-003, EPSTI1-010, EPSTI1-002, EPSTI1-005, EPSTI1-008, EPSTI1-006, EPSTI1-004, EPSTI1-007 |
| chr15 | 77285359 | 77287906 | 2548 | 13 | 6.45e-03 | 0.064 | 0.013 | PSTPIP1-201, PSTPIP1-002, PSTPIP1-001, PSTPIP1-012, PSTPIP1-017, PSTPIP1-009, PSTPIP1-006, PSTPIP1-003, PSTPIP1-005, PSTPIP1-004, PSTPIP1-007, PSTPIP1-018, PSTPIP1-008 |
| chr17 | 62777648 | 62778413 | 766 | 13 | 6.71e-03 | 0.051 | 0.016 | hsa-mir-6080.1-201, hsa-mir-6080.1-001, hsa-mir-6080.1-002 |
| chr19 | 39738664 | 39740501 | 1838 | 10 | 6.89e-03 | 0.073 | 0.018 | IFNL4-001 |
| chr4 | 681086 | 684552 | 3467 | 20 | 7.24e-03 | 0.055 | 0.012 | MFSD7-001, MFSD7-003, MFSD7-004, MFSD7-005, MFSD7-007, MFSD7-009, MFSD7-008, MFSD7-002 |
| chr13 | 1.15E+08 | 1.15E+08 | 1675 | 13 | 7.26e-03 | 0.076 | 0.014 |  |
| chr16 | 89363047 | 89364225 | 1179 | 10 | 7.39e-03 | 0.053 | 0.018 | AC137932.5-001, AC137932.5-002 |
| chr22 | 19512228 | 19513971 | 1744 | 13 | 7.56e-03 | 0.035 | 0.013 | CLDN5-003, CLDN5-001, CLDN5-002 |
| chr11 | 71258392 | 71260323 | 1932 | 10 | 7.85e-03 | 0.059 | 0.013 | KRTAP5-9-001 |
| chr8 | 54935016 | 54936189 | 1174 | 11 | 8.51e-03 | 0.115 | 0.023 | TCEA1-013, TCEA1-004, TCEA1-009, TCEA1-010, TCEA1-008, TCEA1-011, TCEA1-001, TCEA1-002, TCEA1-005, TCEA1-006, TCEA1-003 |
| chr6 | 33141152 | 33142317 | 1166 | 11 | 8.97e-03 | 0.055 | 0.007 |  |
| chr6 | 32904061 | 32905320 | 1260 | 14 | 9.02e-03 | 0.077 | 0.025 | AL645941.1-201, HLA-DMB-006, HLA-DMB-007, HLA-DMB-004, HLA-DMB-005 |
| chr6 | 29598695 | 29599836 | 1142 | 11 | 9.99e-03 | 0.037 | 0.021 | GABBR1-201, GABBR1-003, GABBR1-001, GABBR1-015, GABBR1-009, GABBR1-013, GABBR1-016, GABBR1-017, GABBR1-014, GABBR1-005, GABBR1-006 |
| chr7 | 1584479 | 1586256 | 1778 | 10 | 1.07e-02 | 0.06 | 0.017 | TMEM184A-012, TMEM184A-010 |
| chr17 | 77924371 | 77925938 | 1568 | 11 | 1.12e-02 | 0.038 | 0.015 | TBC1D16-003, TBC1D16-008, TBC1D16-004, TBC1D16-006, TBC1D16-009, TBC1D16-007, TBC1D16-002 |
| chr8 | 1.42E+08 | 1.42E+08 | 1655 | 14 | 1.15e-02 | 0.083 | 0.023 | SLC45A4-201, RP11-10J21.4-001, SLC45A4-003, RP11-10J21.3-001 |
| chr8 | 49647579 | 49648519 | 941 | 13 | 1.15e-02 | 0.067 | 0.011 | EFCAB1-001, EFCAB1-201, EFCAB1-002, EFCAB1-007, EFCAB1-009, EFCAB1-008 |
| chr17 | 25798180 | 25799447 | 1268 | 11 | 1.20e-02 | -0.039 | -0.015 | KSR1-201, KSR1-001, KSR1-008, KSR1-007 |
| chr22 | 45608023 | 45608713 | 691 | 11 | 1.22e-02 | 0.039 | 0.019 | KIAA0930-004, KIAA0930-009, KIAA0930-012, KIAA0930-201, KIAA0930-003, KIAA0930-011, KIAA0930-010 |
| chr17 | 80349836 | 80351457 | 1622 | 13 | 1.23e-02 | 0.091 | 0.015 | OGFOD3-011, OGFOD3-004, OGFOD3-012 |
| chr11 | 69259247 | 69261044 | 1798 | 10 | 1.44e-02 | 0.054 | 0.02 | AP000439.2-001 |
| chr19 | 46806404 | 46807660 | 1257 | 10 | 1.59e-02 | 0.094 | 0.026 | HIF3A-006, HIF3A-202, HIF3A-013, HIF3A-015, HIF3A-023, HIF3A-024 |
| chr13 | 50701960 | 50703841 | 1882 | 13 | 1.72e-02 | 0.041 | 0.017 |  |
| chr12 | 6657744 | 6658945 | 1202 | 10 | 1.86e-02 | 0.078 | 0.02 | IFFO1-010, IFFO1-004, IFFO1-005 |
| chr6 | 25041912 | 25043046 | 1135 | 15 | 1.98e-02 | -0.044 | -0.015 | RP3-425P12.5-001, FAM65B-201, RP11-367G6.3-001 |
| chr17 | 19313024 | 19314408 | 1385 | 10 | 1.98e-02 | 0.03 | 0.009 | RNF112-001, RNF112-002, RNF112-005, RNF112-006 |
| chr6 | 31846769 | 31848534 | 1766 | 17 | 2.30e-02 | 0.05 | 0.009 | SLC44A4-001, SLC44A4-201, SLC44A4-004, SLC44A4-003, SLC44A4-002, SLC44A4-202 |
| chr15 | 40632399 | 40633816 | 1418 | 12 | 2.64e-02 | 0.08 | 0.023 | C15orf52-003, C15orf52-005, C15orf52-004, C15orf52-002, C15orf52-007 |
| chr1 | 76261602 | 76262984 | 1383 | 11 | 2.65e-02 | -0.048 | -0.007 | MSH4-001 |
| chr19 | 49992293 | 49993915 | 1623 | 13 | 2.75e-02 | -0.041 | -0.012 | SNORD33-201, SNORD35A-201, SNORD32A-201, SNORD34-201, RPL13A-001, RPL13A-009, RPL13A-011, RPL13A-007, RPL13A-010, RPL13A-003, RPL13A-012, RPL13A-008, RPL13A-006, RPL13A-004, RPL13A-005, RPL13A-002 |
| chr7 | 27162051 | 27163331 | 1281 | 10 | 3.02e-02 | 0.063 | 0.014 | HOXA-AS2-001, HOXA-AS2-005, HOXA-AS2-003, HOXA-AS2-002, HOXA-AS2-004, HOXA-AS2-007 |
| chr6 | 1.07E+08 | 1.07E+08 | 1294 | 14 | 3.09e-02 | -0.037 | -0.018 | PRDM1-002, PRDM1-007, PRDM1-003, RP1-134E15.3-001 |
| chr12 | 1.24E+08 | 1.24E+08 | 1444 | 12 | 3.32e-02 | 0.024 | 0.006 | CDK2AP1-006, CDK2AP1-002, CDK2AP1-003, CDK2AP1-005 |
| chr10 | 1.04E+08 | 1.04E+08 | 1070 | 11 | 3.41e-02 | 0.027 | 0.013 | C10orf95-002, RP11-18I14.10-001, RP11-18I14.10-002, RP11-18I14.10-005, RP11-18I14.10-007, RP11-18I14.10-006, RP11-18I14.10-008, RP11-18I14.10-004, RP11-18I14.10-003 |
| chr13 | 31505976 | 31507578 | 1603 | 14 | 3.45e-02 | 0.034 | 0.012 | TEX26-001, TEX26-AS1-001, TEX26-AS1-003, TEX26-AS1-013, TEX26-002, TEX26-005, TEX26-AS1-006, TEX26-AS1-002, TEX26-AS1-014, TEX26-AS1-012, TEX26-AS1-009, TEX26-AS1-004, TEX26-AS1-010, TEX26-AS1-005, TEX26-AS1-017, TEX26-AS1-016, TEX26-AS1-008, TEX26-AS1-007, TEX26-AS1-011, TEX26-AS1-015 |
| chr4 | 39481691 | 39483194 | 1504 | 13 | 3.53e-02 | 0.077 | 0.025 | RP11-472B18.1-001, RP11-472B18.1-003, RP11-472B18.1-004 |
| chr14 | 57735545 | 57736365 | 821 | 14 | 3.78e-02 | 0.016 | 0.005 | AP5M1-001, EXOC5-002, EXOC5-001, AP5M1-003, AP5M1-006, EXOC5-008, AP5M1-002, EXOC5-003, AP5M1-005, EXOC5-005, EXOC5-006, AP5M1-004 |
| chr15 | 78555890 | 78557094 | 1205 | 15 | 3.81e-02 | 0.027 | 0.005 | DNAJA4-003, DNAJA4-002, DNAJA4-001, DNAJA4-004, DNAJA4-012, DNAJA4-006, DNAJA4-011, DNAJA4-005, DNAJA4-007, RP11-762H8.3-001, RP11-762H8.3-002 |
| chr12 | 57586714 | 57588350 | 1637 | 12 | 4.35e-02 | 0.043 | -0.002 | MIR1228-201 |
| chr12 | 96388589 | 96390366 | 1778 | 12 | 4.37e-02 | 0.086 | 0.018 | HAL-001, HAL-002, HAL-201, HAL-003, HAL-010, HAL-005, RP11-256L6.3-001, HAL-006 |
| chr17 | 6898315 | 6900799 | 2485 | 23 | 4.38e-02 | 0.064 | 0.017 | ALOX12-001, ALOX12-003, RP11-589P10.5-001 |
| chr6 | 30610592 | 30612330 | 1739 | 12 | 4.52e-02 | 0.093 | 0.014 | ATAT1-009 |
| chr15 | 40861240 | 40862047 | 808 | 13 | 4.71e-02 | -0.025 | -0.006 | RPUSD2-001, RPUSD2-004 |
| chr15 | 74494241 | 74495657 | 1417 | 16 | 4.83e-02 | 0.039 | 0.012 | STRA6-001, RP11-60L3.1-004, RP11-60L3.1-001, RP11-60L3.1-003, STRA6-005, STRA6-004, STRA6-015, STRA6-020, STRA6-013, STRA6-016, STRA6-021, STRA6-012, STRA6-014 |
| chr10 | 63807920 | 63809170 | 1251 | 16 | 5.27e-02 | -0.04 | -0.012 | ARID5B-002 |
| chr6 | 32019966 | 32021154 | 1189 | 11 | 5.33e-02 | -0.036 | -0.013 |  |
| chr3 | 1.38E+08 | 1.38E+08 | 1461 | 16 | 5.74e-02 | 0.06 | 0.013 | DZIP1L-001, DZIP1L-004, DZIP1L-002, DZIP1L-003 |
| chr16 | 85095535 | 85096813 | 1279 | 11 | 5.78e-02 | 0.058 | 0.013 | KIAA0513-001 |
| chr12 | 6959471 | 6960807 | 1337 | 13 | 5.80e-02 | 0.034 | 0.005 | CDCA3-006, USP5-001, USP5-002, CDCA3-011, CDCA3-003, CDCA3-004, CDCA3-008, CDCA3-002, CDCA3-010, CDCA3-007, USP5-003, CDCA3-009, CDCA3-005, CDCA3-012, CDCA3-013 |
| chr10 | 3822379 | 3825031 | 2653 | 14 | 6.05e-02 | -0.053 | -0.008 | KLF6-004, KLF6-005, KLF6-006 |
| chr4 | 1.24E+08 | 1.24E+08 | 603 | 10 | 6.74e-02 | 0.038 | 0.012 | FGF2-001, AC021205.1-201, FGF2-002 |
| chr5 | 1.35E+08 | 1.35E+08 | 1339 | 14 | 6.86e-02 | 0.038 | 0.011 | H2AFY-004, H2AFY-002, H2AFY-008, H2AFY-003, H2AFY-014, H2AFY-019, H2AFY-006, H2AFY-017, H2AFY-021, H2AFY-015, H2AFY-012, H2AFY-005, H2AFY-001, CTC-203F4.2-001 |
| chr20 | 50418952 | 50419348 | 397 | 10 | 6.89e-02 | 0.049 | 0.024 | SALL4-001, SALL4-201, SALL4-002, SALL4-003 |
| chr4 | 24795830 | 24797140 | 1311 | 10 | 6.96e-02 | 0.044 | 0.017 | SOD3-001 |
| chr6 | 33039804 | 33041583 | 1780 | 13 | 7.14e-02 | -0.038 | -0.022 | HLA-DPA1-205, HLA-DPA1-007, HLA-DPA1-003 |
| chr17 | 1943561 | 1945160 | 1600 | 13 | 7.16e-02 | 0.123 | 0.02 | DPH1-019, DPH1-005, DPH1-009, OVCA2-001, DPH1-016, RP11-667K14.4-001, DPH1-006, DPH1-017, DPH1-012, DPH1-018 |
| chr10 | 1.15E+08 | 1.15E+08 | 980 | 10 | 7.18e-02 | 0.028 | 0.013 | TCF7L2-006, TCF7L2-011, TCF7L2-013, TCF7L2-008 |
| chr6 | 32119874 | 32121843 | 1970 | 46 | 7.37e-02 | 0.072 | 0.018 | PRRT1-002, PPT2-207, PPT2-001, PPT2-003, PPT2-004, PRRT1-001, PRRT1-201, PPT2-002, PPT2-007, PPT2-EGFL8-002, PPT2-009, PPT2-EGFL8-001, PRRT1-005, PPT2-012, PPT2-208, PPT2-209, PPT2-EGFL8-006, PPT2-013, PRRT1-006, PRRT1-009, PPT2-008, PRRT1-012, PRRT1-010, PPT2-010, PRRT1-008, PRRT1-003, PRRT1-011, PRRT1-004, PPT2-EGFL8-005, PPT2-EGFL8-004 |
| chr15 | 63114897 | 63116514 | 1618 | 10 | 7.65e-02 | -0.017 | -0.008 | MIR190A-201 |
| chr5 | 1498266 | 1499797 | 1532 | 13 | 7.96e-02 | 0.068 | 0.015 |  |
| chr16 | 68027075 | 68028395 | 1321 | 10 | 8.07e-02 | 0.028 | 0.008 | DPEP2-002, DPEP2-009, DUS2-015 |
| chr16 | 89167018 | 89169289 | 2272 | 11 | 8.08e-02 | 0.078 | 0.023 | ACSF3-015, ACSF3-006, ACSF3-014, ACSF3-012 |
| chr3 | 1.52E+08 | 1.52E+08 | 1519 | 11 | 8.32e-02 | -0.057 | -0.021 | MBNL1-201, MBNL1-202, MBNL1-004, MBNL1-001, MBNL1-AS1-002, MBNL1-016, MBNL1-017, MBNL1-002, MBNL1-003, MBNL1-015, MBNL1-AS1-001 |
| chr19 | 47287778 | 47289611 | 1834 | 12 | 8.43e-02 | -0.046 | -0.013 | SLC1A5-005, SLC1A5-004, SLC1A5-007, SLC1A5-003, SLC1A5-006 |
| chr6 | 32171786 | 32172871 | 1086 | 12 | 8.72e-02 | 0.036 | -0.003 | NOTCH4-003 |
| chr1 | 42384437 | 42385941 | 1505 | 11 | 8.88e-02 | -0.05 | -0.021 | HIVEP3-002, HIVEP3-001, HIVEP3-005 |
| chr5 | 1.77E+08 | 1.77E+08 | 2268 | 13 | 9.15e-02 | 0.04 | 0.017 | PFN3-001, GRK6-009, F12-005, F12-006 |
| chr1 | 1.5E+08 | 1.5E+08 | 1121 | 11 | 9.18e-02 | 0.022 | 0.002 | MTMR11-201, MTMR11-001, MTMR11-202, MTMR11-203, MTMR11-003, MTMR11-007, MTMR11-005, MTMR11-002, MTMR11-004 |
| chr14 | 24835104 | 24837184 | 2081 | 19 | 9.56e-02 | -0.043 | 0.001 | NFATC4-001, NFATC4-002, NFATC4-021, NFATC4-006, NFATC4-003, NFATC4-009, NFATC4-011, NFATC4-016, NFATC4-017, NFATC4-013, NFATC4-019, NFATC4-004, NFATC4-012, NFATC4-020, NFATC4-034, NFATC4-031, NFATC4-008, NFATC4-023, NFATC4-007, NFATC4-022, NFATC4-010, NFATC4-024, NFATC4-005, NFATC4-014, NFATC4-018, NFATC4-032 |
| chr1 | 32040692 | 32042157 | 1466 | 14 | 9.78e-02 | 0.045 | 0.009 | TINAGL1-001, RP11-73M7.1-001, TINAGL1-201, TINAGL1-202, TINAGL1-006, TINAGL1-007, TINAGL1-008, TINAGL1-005, TINAGL1-002, TINAGL1-203 |
| chr14 | 21491045 | 21492472 | 1428 | 14 | 1.08e-01 | 0.047 | 0.006 | NDRG2-004, NDRG2-008, NDRG2-003, NDRG2-002, NDRG2-070, NDRG2-001, NDRG2-007, NDRG2-010, NDRG2-005, NDRG2-201, NDRG2-202, NDRG2-203, NDRG2-006, AL161668.5-002, TPPP2-013, AL161668.5-001, NDRG2-050, NDRG2-011, NDRG2-058, NDRG2-071, NDRG2-069, NDRG2-053, NDRG2-057, NDRG2-065, NDRG2-052, NDRG2-033, NDRG2-056, NDRG2-017, NDRG2-009, NDRG2-077, NDRG2-061, NDRG2-051, NDRG2-023, NDRG2-020, NDRG2-046, NDRG2-035, NDRG2-043, NDRG2-064, NDRG2-045, NDRG2-040, NDRG2-037, NDRG2-029, NDRG2-012, NDRG2-039, NDRG2-042, NDRG2-054, NDRG2-036, NDRG2-025, NDRG2-026, NDRG2-034, NDRG2-013, NDRG2-030, NDRG2-066, NDRG2-038, NDRG2-024, NDRG2-044, NDRG2-032, NDRG2-027, NDRG2-021, NDRG2-055, NDRG2-048, NDRG2-049, NDRG2-059, NDRG2-063, NDRG2-062, NDRG2-047, NDRG2-041, NDRG2-068, NDRG2-060, NDRG2-014, NDRG2-031, NDRG2-076, NDRG2-022, NDRG2-028, NDRG2-067 |
| chr2 | 27301057 | 27301943 | 887 | 10 | 1.09e-01 | 0.028 | 0.014 | EMILIN1-001 |
| chr12 | 66275542 | 66276974 | 1433 | 12 | 1.12e-01 | 0.062 | 0.012 | RP11-366L20.2-001, RP11-366L20.2-004, RP11-366L20.2-003, RP11-366L20.2-002 |
| chr10 | 1.32E+08 | 1.32E+08 | 1101 | 10 | 1.15e-01 | 0.046 | 0.014 | RP11-109A6.3-001 |
| chr9 | 1.4E+08 | 1.4E+08 | 2168 | 12 | 1.17e-01 | 0.047 | 0.006 | PHPT1-001, MAMDC4-005, MAMDC4-201, PHPT1-005, PHPT1-004, MAMDC4-001, PHPT1-002, PHPT1-006 |
| chr1 | 1.55E+08 | 1.55E+08 | 1453 | 14 | 1.24e-01 | 0.044 | 0.009 | SLC50A1-003, SLC50A1-002, SLC50A1-001, SLC50A1-008, SLC50A1-010, SLC50A1-009, SLC50A1-012, SLC50A1-005, SLC50A1-006, SLC50A1-004, SLC50A1-007, SLC50A1-013 |
| chr6 | 1.71E+08 | 1.71E+08 | 3077 | 18 | 1.26e-01 | 0.116 | 0.02 |  |
| chr17 | 71308194 | 71308806 | 613 | 11 | 1.30e-01 | 0.045 | 0.006 | CDC42EP4-001, CDC42EP4-003, CDC42EP4-005, CDC42EP4-004, CDC42EP4-006, CDC42EP4-009, CDC42EP4-007 |
| chr12 | 51784414 | 51785378 | 965 | 13 | 1.31e-01 | 0.067 | 0.009 | GALNT6-001, SLC4A8-016, SLC4A8-002, GALNT6-012, GALNT6-018, GALNT6-021, GALNT6-015, SLC4A8-017, GALNT6-008, GALNT6-005, GALNT6-017, GALNT6-011, GALNT6-009, GALNT6-020, GALNT6-016, GALNT6-013, GALNT6-007, GALNT6-010, GALNT6-006 |
| chr1 | 1.5E+08 | 1.5E+08 | 2039 | 16 | 1.39e-01 | -0.025 | 0 | C1orf51-001, C1orf51-003, C1orf51-002, C1orf51-004, C1orf51-005, C1orf51-007, C1orf51-006 |
| chr12 | 53661219 | 53662405 | 1187 | 10 | 1.39e-01 | 0.02 | 0 | ESPL1-201, ESPL1-002, ESPL1-001, ESPL1-007 |
| chr10 | 13748597 | 13749953 | 1357 | 11 | 1.41e-01 | 0.02 | 0.008 | AL157392.1-201, FRMD4A-008, FRMD4A-007 |
| chr12 | 12764255 | 12764762 | 508 | 10 | 1.45e-01 | -0.025 | -0.003 | CREBL2-001, CREBL2-003 |
| chr14 | 23623480 | 23624788 | 1309 | 10 | 1.51e-01 | 0.065 | 0.025 | SLC7A8-006, RNU6-1138P-201, SLC7A8-201, SLC7A8-002, SLC7A8-007, SLC7A8-005 |
| chr1 | 6453910 | 6454339 | 430 | 10 | 1.52e-01 | -0.017 | -0.002 | ACOT7-006, ACOT7-002, ACOT7-003, ACOT7-202, RP1-202O8.3-001, ACOT7-009 |
| chr3 | 50303757 | 50304463 | 707 | 10 | 1.53e-01 | -0.02 | -0.002 | SEMA3B-201, SEMA3B-001, SEMA3B-010, SEMA3B-AS1-001, SEMA3B-002 |
| chr14 | 21269744 | 21271485 | 1742 | 14 | 1.58e-01 | -0.035 | -0.009 | RNASE1-002, RNASE1-001, RNASE1-003, RNASE1-004, RNASE1-005 |
| chr3 | 42699933 | 42701042 | 1110 | 11 | 1.60e-01 | 0.061 | 0.015 | ZBTB47-003 |
| chr16 | 30366665 | 30367660 | 996 | 12 | 1.61e-01 | 0.036 | 0.007 | CD2BP2-001, RP11-347C12.10-001, CD2BP2-003, CD2BP2-002 |
| chr19 | 13055829 | 13056485 | 657 | 10 | 1.63e-01 | -0.058 | -0.013 | RAD23A-001, RAD23A-201, RAD23A-002, CALR-005, CALR-007, RAD23A-006, RAD23A-007, CTC-425F1.4-001, RAD23A-003, RAD23A-005, RAD23A-004 |
| chr4 | 16085367 | 16086292 | 926 | 13 | 1.66e-01 | -0.061 | -0.014 | PROM1-008, PROM1-001, PROM1-003, PROM1-010, PROM1-009, PROM1-004, PROM1-011, PROM1-204 |
| chr17 | 7340375 | 7341616 | 1242 | 10 | 1.72e-01 | 0.02 | 0.008 | FGF11-001, TMEM102-001, TMEM102-002, RP11-104H15.9-001, FGF11-004, FGF11-002, RP11-104H15.10-001 |
| chr7 | 4049531 | 4051198 | 1668 | 11 | 1.74e-01 | 0.066 | -0.006 |  |
| chr2 | 74728623 | 74730047 | 1425 | 10 | 1.74e-01 | 0.031 | 0.01 | LBX2-003, LBX2-002, LBX2-001, LBX2-AS1-001, LBX2-005, LBX2-AS1-002, RP11-523H20.3-001 |
| chr6 | 31539539 | 31540750 | 1212 | 18 | 1.77e-01 | -0.061 | -0.025 | LTA-001, LTA-002, LTA-004, LTA-003 |
| chr12 | 1.07E+08 | 1.07E+08 | 2250 | 18 | 1.82e-01 | 0.048 | 0.005 | RIC8B-002, RIC8B-001, RIC8B-006, RIC8B-003, RIC8B-005, RIC8B-012, RP11-144F15.1-002, RIC8B-008, RIC8B-007, RP11-144F15.1-001, RIC8B-009 |
| chr3 | 1.24E+08 | 1.24E+08 | 507 | 11 | 1.85e-01 | -0.022 | -0.009 | KALRN-005, KALRN-201, KALRN-013, KALRN-007, KALRN-012 |
| chr2 | 2.34E+08 | 2.34E+08 | 1607 | 17 | 1.92e-01 | 0.06 | 0.01 | INPP5D-201, INPP5D-002, INPP5D-005, INPP5D-204 |
| chr5 | 1.41E+08 | 1.41E+08 | 1206 | 12 | 1.95e-01 | 0.054 | 0.014 | ARAP3-001, ARAP3-005, ARAP3-002 |
| chr1 | 36038775 | 36039885 | 1111 | 11 | 1.96e-01 | 0.048 | 0.012 | TFAP2E-001, RP4-728D4.2-002 |
| chr1 | 2.15E+08 | 2.15E+08 | 917 | 10 | 2.05e-01 | -0.038 | -0.005 | CENPF-001, CENPF-003, CENPF-004 |
| chr1 | 65432346 | 65433360 | 1015 | 10 | 2.19e-01 | -0.026 | -0.001 | JAK1-001 |
| chr16 | 29831566 | 29833153 | 1588 | 11 | 2.19e-01 | -0.036 | -0.011 | MVP-001, MVP-201, MVP-202, MVP-015, MVP-014, MVP-018, MVP-002, AC009133.12-002, MVP-009, MVP-013, MVP-011, MVP-008, AC009133.20-001, AC009133.12-001, MVP-017, MVP-010 |
| chr4 | 54457645 | 54459009 | 1365 | 12 | 2.20e-01 | 0.081 | 0.013 | LNX1-001, LNX1-AS2-002, LNX1-AS2-001, LNX1-003 |
| chr17 | 19648157 | 19649293 | 1137 | 14 | 2.21e-01 | 0.052 | 0.006 | ALDH3A1-018, ALDH3A1-009, ALDH3A1-201, ALDH3A1-010, ALDH3A1-019, ALDH3A1-016, ALDH3A1-015, ALDH3A1-017 |
| chr3 | 99978949 | 99979976 | 1028 | 18 | 2.23e-01 | 0.053 | 0.012 | TBC1D23-001, TBC1D23-002, TBC1D23-008, TBC1D23-005 |
| chr16 | 55542330 | 55543166 | 837 | 12 | 2.25e-01 | -0.055 | -0.007 | LPCAT2-001, LPCAT2-003 |
| chr1 | 1.54E+08 | 1.54E+08 | 614 | 10 | 2.26e-01 | -0.013 | -0.001 | RAB13-001, RAB13-002, RAB13-004, RAB13-003 |
| chr6 | 30623964 | 30624769 | 806 | 12 | 2.28e-01 | 0.032 | 0.012 |  |
| chr6 | 30301427 | 30303126 | 1700 | 10 | 2.29e-01 | 0.047 | 0.015 |  |
| chr1 | 45272633 | 45273680 | 1048 | 10 | 2.33e-01 | -0.048 | -0.008 | TCTEX1D4-001, TCTEX1D4-201, BTBD19-008, BTBD19-009, BTBD19-201, BTBD19-202, BTBD19-006, BTBD19-001 |
| chr4 | 46391259 | 46391737 | 479 | 10 | 2.35e-01 | 0.017 | 0.006 | GABRA2-002, GABRA2-001, RP11-436F23.1-001, GABRA2-008, GABRA2-007, GABRA2-013, GABRA2-003, GABRA2-009, GABRA2-010, GABRA2-014, GABRA2-011, GABRA2-006, GABRA2-004, GABRA2-201 |
| chr19 | 55549414 | 55550348 | 935 | 11 | 2.35e-01 | 0.062 | 0.02 | GP6-002, GP6-003, GP6-001 |
| chr11 | 1.18E+08 | 1.18E+08 | 1352 | 11 | 2.38e-01 | 0.021 | 0.003 | IL10RA-001, IL10RA-003, IL10RA-006, IL10RA-002, IL10RA-007, IL10RA-005, IL10RA-004, IL10RA-201, IL10RA-202 |
| chr6 | 32805142 | 32806022 | 881 | 12 | 2.38e-01 | -0.033 | -0.012 | TAP2-001, TAP2-002, TAP2-001 |
| chr12 | 27395963 | 27397422 | 1460 | 14 | 2.41e-01 | 0.071 | 0.012 | STK38L-001, STK38L-010, STK38L-201, STK38L-011, STK38L-007, STK38L-004, STK38L-005, STK38L-009, STK38L-012, STK38L-008 |
| chr6 | 30130420 | 30132226 | 1807 | 28 | 2.42e-01 | -0.101 | -0.014 | TRIM15-203, TRIM15-001, TRIM10-001, TRIM10-002 |
| chr4 | 1.46E+08 | 1.46E+08 | 818 | 15 | 2.43e-01 | -0.031 | -0.002 | ABCE1-001, ANAPC10-001, ANAPC10-002, ANAPC10-007, ABCE1-004, ANAPC10-006, ABCE1-006, ANAPC10-014, ANAPC10-015, ABCE1-003, ANAPC10-010, ANAPC10-011, ABCE1-010, ANAPC10-003, ANAPC10-008, ANAPC10-009, ANAPC10-005, ANAPC10-016, ANAPC10-013, ANAPC10-004, ANAPC10-012 |
| chr7 | 75114950 | 75115859 | 910 | 11 | 2.44e-01 | -0.012 | 0.003 | POM121C-002, AC006014.7-001, POM121C-001 |
| chr6 | 31867698 | 31869304 | 1607 | 42 | 2.46e-01 | 0.1 | 0.014 | ZBTB12-001, C2-015, C2-014 |
| chr1 | 39339004 | 39339553 | 550 | 10 | 2.49e-01 | 0.014 | 0.002 | MYCBP-001, RP5-864K19.4-002, MYCBP-002, MYCBP-007, MYCBP-004, MYCBP-003 |
| chr10 | 1.35E+08 | 1.35E+08 | 856 | 12 | 2.51e-01 | 0.055 | 0.013 | VENTX-001 |
| chr3 | 1.02E+08 | 1.02E+08 | 777 | 11 | 2.52e-01 | -0.058 | -0.012 | NFKBIZ-001, NFKBIZ-005, NFKBIZ-004 |
| chr12 | 6418690 | 6419784 | 1095 | 12 | 2.54e-01 | 0.027 | 0.009 | PLEKHG6-001, PLEKHG6-003, PLEKHG6-002, PLEKHG6-007 |
| chr9 | 1.24E+08 | 1.24E+08 | 1071 | 11 | 2.62e-01 | 0.096 | 0.016 | GSN-201, GSN-202, GSN-203, GSN-204, GSN-002, GSN-205, GSN-206, GSN-003, GSN-007 |
| chr7 | 1.51E+08 | 1.51E+08 | 704 | 10 | 2.69e-01 | 0.033 | 0.011 | WDR86-003, WDR86-AS1-005, WDR86-AS1-004, WDR86-004, WDR86-001, WDR86-AS1-002, WDR86-007, WDR86-AS1-003, WDR86-006 |
| chr6 | 26225246 | 26225767 | 522 | 10 | 2.75e-01 | 0.058 | 0.022 | HIST1H3E-001 |
| chr11 | 66232921 | 66234348 | 1428 | 18 | 2.77e-01 | 0.027 | 0.008 | PELI3-001, PELI3-002, PELI3-004, MRPL11-003, PELI3-007, PELI3-006, PELI3-008, PELI3-003 |
| chr15 | 40331067 | 40332663 | 1597 | 14 | 2.82e-01 | 0.023 | 0.002 | SRP14-001, SRP14-AS1-001, SRP14-003, SRP14-002, SRP14-005, SRP14-007, SRP14-006, SRP14-008, SRP14-AS1-002, SRP14-004 |
| chr7 | 27184667 | 27188770 | 4104 | 28 | 2.84e-01 | -0.052 | -0.012 | HOXA5-001, HOXA6-001, HOXA-AS3-001, HOXA-AS3-005, HOXA6-002, HOXA-AS3-002 |
| chr1 | 36789388 | 36790444 | 1057 | 11 | 2.84e-01 | 0.013 | 0.006 | EVA1B-001, RP11-268J15.5-001, EVA1B-002 |
| chr8 | 1.45E+08 | 1.45E+08 | 1661 | 13 | 2.88e-01 | 0.037 | 0.009 | PLEC-008 |
| chr22 | 19938096 | 19939028 | 933 | 10 | 2.89e-01 | 0.056 | 0.014 | COMT-007, COMT-001, COMT-005 |
| chr2 | 2.19E+08 | 2.19E+08 | 986 | 11 | 2.97e-01 | 0.042 | 0.005 | SLC11A1-001, SLC11A1-003, SLC11A1-004, SLC11A1-009, SLC11A1-011, SLC11A1-016, SLC11A1-015, SLC11A1-013, SLC11A1-002, SLC11A1-014, SLC11A1-017, SLC11A1-007, SLC11A1-006, SLC11A1-010, SLC11A1-201 |
| chr1 | 54665230 | 54665948 | 719 | 13 | 3.00e-01 | 0.01 | 0.003 | RP11-446E24.4-001, MRPL37-201, MRPL37-001, CYB5RL-007, MRPL37-002, CYB5RL-003, CYB5RL-009, CYB5RL-203, CYB5RL-204, MRPL37-005 |
| chr17 | 38464679 | 38465510 | 832 | 11 | 3.04e-01 | -0.037 | -0.001 | RARA-001, RARA-008 |
| chr6 | 30297174 | 30297941 | 768 | 10 | 3.05e-01 | 0.056 | 0.023 | TRIM39-002, TRIM39-009, TRIM39-008, TRIM39-RPP21-001 |
| chr14 | 75593685 | 75594775 | 1091 | 15 | 3.12e-01 | 0.023 | 0.004 | NEK9-001, NEK9-005, NEK9-011, NEK9-007, NEK9-002, RP11-950C14.7-001, NEK9-006 |
| chr11 | 71639257 | 71640085 | 829 | 14 | 3.13e-01 | 0.019 | 0.006 | RNF121-001, RNF121-003, RNF121-002, RNF121-004, RNF121-011, RNF121-005, RP11-849H4.2-004, RP11-849H4.2-003, RNF121-010, RP11-849H4.2-006, RP11-849H4.2-001, RNF121-007, RNF121-006, RNF121-008, RP11-849H4.2-005, RP11-849H4.2-002, RNF121-009, RP11-849H4.2-008, RNF121-201 |
| chr5 | 76114114 | 76116535 | 2422 | 21 | 3.14e-01 | -0.059 | -0.01 | F2RL1-001, F2RL1-002 |
| chr6 | 30113074 | 30114039 | 966 | 10 | 3.16e-01 | 0.039 | 0.013 |  |
| chr2 | 97405651 | 97406466 | 816 | 11 | 3.20e-01 | -0.029 | -0.002 | LMAN2L-001, LMAN2L-007, LMAN2L-201, LMAN2L-006, LMAN2L-005, LMAN2L-003, LMAN2L-004, LMAN2L-002, LMAN2L-202, LMAN2L-203 |
| chr1 | 59042745 | 59043576 | 832 | 11 | 3.23e-01 | 0.061 | 0.017 | TACSTD2-001 |
| chr6 | 90271339 | 90272492 | 1154 | 12 | 3.25e-01 | 0.044 | 0.014 | ANKRD6-004, ANKRD6-006, ANKRD6-018, ANKRD6-017 |
| chr19 | 45926585 | 45927696 | 1112 | 19 | 3.31e-01 | 0.015 | 0.005 | ERCC1-003, ERCC1-001, ERCC1-002, ERCC1-007, ERCC1-006, ERCC1-010, ERCC1-013, ERCC1-009, ERCC1-008 |
| chr5 | 1201033 | 1201978 | 946 | 12 | 3.37e-01 | 0.031 | 0.006 | SLC6A19-001, SLC6A19-002 |
| chr14 | 24583056 | 24583924 | 869 | 11 | 3.47e-01 | 0.027 | 0.006 | DCAF11-008, DCAF11-002, DCAF11-003, DCAF11-001, DCAF11-004, DCAF11-029, DCAF11-031, DCAF11-037, DCAF11-017, DCAF11-027, NRL-005, DCAF11-036, DCAF11-034, DCAF11-016, DCAF11-007, DCAF11-028, DCAF11-005, DCAF11-020, DCAF11-021, DCAF11-025, DCAF11-033, DCAF11-015, DCAF11-030, DCAF11-035, DCAF11-013, DCAF11-032, DCAF11-023, DCAF11-014, DCAF11-024, DCAF11-018, DCAF11-019, DCAF11-039, NRL-004, DCAF11-038, DCAF11-026, DCAF11-022 |
| chr1 | 45243032 | 45243960 | 929 | 10 | 3.51e-01 | 0.081 | 0.011 | SNORD46-201, SNORD38A-201, RPS8-003, SNORD38B-201, RPS8-001, RP11-269F19.2-002, RPS8-006, RPS8-005, RPS8-008, RPS8-007, RPS8-004, SNORD55-201 |
| chr6 | 32022470 | 32023650 | 1181 | 14 | 3.52e-01 | -0.036 | -0.014 |  |
| chr3 | 1.96E+08 | 1.96E+08 | 583 | 11 | 3.53e-01 | -0.036 | -0.011 | TM4SF19-001, TM4SF19-001, TM4SF19-002, TM4SF19-004 |
| chr16 | 66612774 | 66613970 | 1197 | 13 | 3.54e-01 | -0.037 | -0.006 | CMTM2-001, CMTM2-002, RP11-403P17.2-001, CMTM2-004 |
| chr6 | 31645405 | 31646834 | 1430 | 12 | 3.56e-01 | 0.035 | 0.004 | LY6G5C-202, LY6G5C-003, LY6G5C-005, LY6G5C-004 |
| chr2 | 1.91E+08 | 1.91E+08 | 1453 | 14 | 3.57e-01 | 0.037 | 0.009 | INPP1-001, INPP1-201, HIBCH-002, INPP1-006, INPP1-004, INPP1-008, INPP1-009, INPP1-005, INPP1-007, INPP1-002, INPP1-010, INPP1-003, INPP1-202 |
| chr8 | 1.46E+08 | 1.46E+08 | 466 | 10 | 3.68e-01 | 0.034 | 0.002 | SLC39A4-002, SLC39A4-001, SLC39A4-010 |
| chr1 | 1.61E+08 | 1.61E+08 | 4219 | 31 | 3.68e-01 | 0.072 | 0.011 | NDUFS2-001, ADAMTS4-002, ADAMTS4-001, NDUFS2-201, NDUFS2-005, NDUFS2-007, NDUFS2-202, NDUFS2-008, NDUFS2-002, NDUFS2-003, NDUFS2-004 |
| chr8 | 33370637 | 33371237 | 601 | 14 | 3.77e-01 | -0.037 | -0.009 | TTI2-002, TTI2-001, SNORD13-201, TTI2-004, TTI2-003 |
| chr19 | 35629022 | 35630651 | 1630 | 19 | 3.78e-01 | 0.077 | 0.014 | FXYD1-001, AC020907.2-201, FXYD1-006, CTD-2527I21.4-002, FXYD1-004, FXYD1-007, FXYD1-003, FXYD1-002, FXYD1-009, FXYD1-005, FXYD1-010, CTD-2527I21.4-001, FXYD1-008 |
| chr12 | 75784144 | 75785295 | 1152 | 12 | 3.86e-01 | -0.044 | 0.001 | GLIPR1L2-003, CAPS2-004, CAPS2-003, GLIPR1L2-201, GLIPR1L2-002, GLIPR1L2-202, CAPS2-006, GLIPR1L2-203, CAPS2-201, CAPS2-007, GLIPR1L2-004, CAPS2-014, CAPS2-011, CAPS2-015, GLIPR1L2-001, CAPS2-012, CAPS2-013 |
| chr15 | 75315579 | 75316240 | 662 | 13 | 3.87e-01 | 0.024 | 0.001 | PPCDC-001, PPCDC-010, PPCDC-005, RP11-151H2.1-001, PPCDC-009, PPCDC-007, PPCDC-003 |
| chr11 | 63439065 | 63439548 | 484 | 13 | 3.87e-01 | -0.016 | 0 | ATL3-201, ATL3-001, ATL3-005, ATL3-002, ATL3-004 |
| chr6 | 32025453 | 32027023 | 1571 | 18 | 3.93e-01 | -0.031 | -0.008 |  |
| chr5 | 1.49E+08 | 1.49E+08 | 826 | 10 | 3.94e-01 | -0.021 | -0.009 | CSNK1A1-001, CSNK1A1-201, CSNK1A1-002, ARHGEF37-002, CSNK1A1-004, CSNK1A1-005, CSNK1A1-003, CSNK1A1-007 |
| chr12 | 53730072 | 53730421 | 350 | 10 | 3.98e-01 | 0.045 | 0.011 | SP7-002, SP7-001, SP7-003 |
| chr11 | 2919689 | 2921176 | 1488 | 20 | 4.01e-01 | 0.057 | 0.003 | SLC22A18-002 |
| chr14 | 88459216 | 88460351 | 1136 | 15 | 4.02e-01 | -0.024 | -0.002 | GALC-001, GALC-005, GALC-002, GALC-003, GALC-007, GALC-009, GALC-010, GALC-006 |
| chr1 | 1.8E+08 | 1.8E+08 | 326 | 10 | 4.11e-01 | -0.046 | -0.002 | TOR1AIP2-001, TOR1AIP2-003, TOR1AIP2-002, TOR1AIP2-004, TOR1AIP2-005, TOR1AIP2-006 |
| chr3 | 71179796 | 71180415 | 620 | 11 | 4.12e-01 | -0.032 | -0.011 | FOXP1-011, FOXP1-009 |
| chr3 | 1.87E+08 | 1.87E+08 | 1337 | 14 | 4.15e-01 | -0.048 | -0.011 | BCL6-001, RP11-211G3.2-001, BCL6-005, BCL6-009, BCL6-006 |
| chr1 | 1.15E+08 | 1.15E+08 | 588 | 11 | 4.20e-01 | -0.016 | 0 | CSDE1-003, CSDE1-001, CSDE1-002, CSDE1-004, CSDE1-011, CSDE1-015, CSDE1-010, CSDE1-013, CSDE1-014, CSDE1-007, CSDE1-012, CSDE1-009 |
| chr14 | 74352920 | 74353709 | 790 | 11 | 4.24e-01 | -0.014 | -0.002 | ZNF410-003, ZNF410-013, ZNF410-014, ZNF410-012, ZNF410-002, ZNF410-009, ZNF410-015, ZNF410-001, ZNF410-005, ZNF410-019, ZNF410-017, ZNF410-004, ZNF410-018, ZNF410-020, ZNF410-021 |
| chr12 | 56660326 | 56660988 | 663 | 11 | 4.26e-01 | 0.021 | 0.003 | COQ10A-001, COQ10A-002, COQ10A-003, COQ10A-005, COQ10A-007, COQ10A-008, COQ10A-004, COQ10A-009, COQ10A-010 |
| chr7 | 95025905 | 95026193 | 289 | 11 | 4.33e-01 | 0.015 | 0.009 | PON3-001, PON3-005, PON3-008, PON3-006, PON3-004, PON3-007, PON3-009, PON3-003, PON1-201 |
| chr3 | 1.92E+08 | 1.92E+08 | 668 | 12 | 4.37e-01 | -0.021 | 0.001 | FGF12-201, RNU1-20P-201, FGF12-005, FGF12-006, FGF12-002, FGF12-007, FGF12-004, FGF12-003 |
| chr11 | 2321770 | 2323459 | 1690 | 31 | 4.53e-01 | 0.017 | 0.006 | TSPAN32-001, TSPAN32-004, TSPAN32-014, TSPAN32-005, C11orf21-001, TSPAN32-007, TSPAN32-002, TSPAN32-003, C11orf21-004, TSPAN32-006, C11orf21-002, TSPAN32-012, TSPAN32-019, TSPAN32-011, TSPAN32-008, TSPAN32-010, C11orf21-005, TSPAN32-009 |
| chr16 | 31213883 | 31214880 | 998 | 13 | 4.54e-01 | 0.02 | 0.006 | PYCARD-001, PYCARD-002, PYCARD-003, C16orf98-001, PYCARD-004 |
| chr4 | 83955829 | 83956393 | 565 | 11 | 4.54e-01 | 0.015 | 0.007 | COPS4-001, COPS4-003, COPS4-006, COPS4-007, COPS4-002, COPS4-011, COPS4-010, COPS4-004, COPS4-008, COPS4-005 |
| chr13 | 1.11E+08 | 1.11E+08 | 1359 | 14 | 4.56e-01 | 0.025 | 0.007 | ING1-001, ING1-004, ING1-002, ING1-005, CARS2-008, CARS2-014, CARS2-015, CARS2-017, CARS2-016 |
| chr2 | 2.07E+08 | 2.07E+08 | 406 | 12 | 4.56e-01 | 0.016 | 0.004 | ZDBF2-001 |
| chr6 | 26183508 | 26184037 | 530 | 11 | 4.58e-01 | -0.032 | -0.006 | HIST1H2BE-001 |
| chr13 | 24844108 | 24844896 | 789 | 10 | 4.62e-01 | 0.078 | 0.008 | SPATA13-201, SPATA13-006, SPATA13-011, SPATA13-005, SPATA13-004 |
| chr1 | 44456739 | 44457394 | 656 | 13 | 4.66e-01 | 0.034 | 0.008 | CCDC24-001, CCDC24-011, CCDC24-003, CCDC24-008, CCDC24-009, CCDC24-007, CCDC24-002, CCDC24-006, CCDC24-005, CCDC24-004 |
| chr12 | 96336246 | 96338038 | 1793 | 17 | 4.68e-01 | -0.05 | 0 | AMDHD1-001, CCDC38-001, CCDC38-002, CCDC38-004, CCDC38-006, AMDHD1-002, CCDC38-005 |
| chr17 | 46622012 | 46622899 | 888 | 13 | 4.69e-01 | -0.062 | -0.016 | HOXB2-001, HOXB-AS1-001, HOXB-AS1-004, HOXB2-003, HOXB-AS1-002, HOXB-AS1-003, HOXB2-002, HOXB2-004 |
| chr6 | 33218604 | 33220041 | 1438 | 13 | 4.71e-01 | -0.045 | -0.01 | HCG25-003, HCG25-002, HCG25-004, HCG25-001 |
| chr12 | 7032793 | 7033911 | 1119 | 12 | 4.72e-01 | 0.056 | 0.008 | ATN1-002, ENO2-015 |
| chr3 | 15374148 | 15374785 | 638 | 11 | 4.78e-01 | 0.035 | 0.006 | SH3BP5-201, SH3BP5-001, SH3BP5-003, SH3BP5-002, SH3BP5-005, SH3BP5-006 |
| chr6 | 31126599 | 31127271 | 673 | 16 | 4.88e-01 | 0.035 | 0.007 | TCF19-002, TCF19-001, CCHCR1-001, CCHCR1-003, CCHCR1-002, CCHCR1-007, CCHCR1-004, CCHCR1-019, CCHCR1-005, CCHCR1-011, CCHCR1-009, CCHCR1-010, CCHCR1-016, CCHCR1-008, TCF19-003, CCHCR1-027, CCHCR1-026, CCHCR1-021, CCHCR1-031, CCHCR1-029, CCHCR1-024, CCHCR1-033, CCHCR1-028, CCHCR1-018, CCHCR1-025, CCHCR1-020, CCHCR1-032, CCHCR1-017, CCHCR1-030, CCHCR1-023, TCF19-004 |
| chr12 | 1.11E+08 | 1.11E+08 | 1846 | 19 | 4.90e-01 | 0.027 | 0.006 | RAD9B-004, VPS29-002, RAD9B-003, RAD9B-001, RAD9B-002, RAD9B-005, RAD9B-006, RAD9B-009, RAD9B-008, RAD9B-007, VPS29-201, VPS29-005, VPS29-007, VPS29-001, VPS29-006, VPS29-008, VPS29-010, VPS29-003, VPS29-009 |
| chr21 | 46340517 | 46341918 | 1402 | 13 | 4.94e-01 | 0.087 | 0.017 | ITGB2-201, ITGB2-008, ITGB2-003, ITGB2-AS1-002, ITGB2-AS1-001, ITGB2-007, ITGB2-016, ITGB2-019, ITGB2-020, ITGB2-018, ITGB2-017, ITGB2-013, ITGB2-021, ITGB2-AS1-004, ITGB2-AS1-005, ITGB2-AS1-003, ITGB2-AS1-006 |
| chr16 | 57405979 | 57406955 | 977 | 11 | 4.95e-01 | 0.026 | 0.006 | CX3CL1-001, CX3CL1-002, CX3CL1-004 |
| chr18 | 11688350 | 11689284 | 935 | 12 | 4.99e-01 | -0.021 | -0.002 | GNAL-001, GNAL-007 |
| chr16 | 57831745 | 57832309 | 565 | 10 | 5.05e-01 | 0.037 | 0.015 | KIFC3-010, KIFC3-002, KIFC3-201, KIFC3-034, KIFC3-033, CTD-2600O9.1-002, KIFC3-032, CTD-2600O9.1-003 |
| chr9 | 91605035 | 91606164 | 1130 | 13 | 5.18e-01 | 0.039 | 0.012 | C9orf47-003, S1PR3-002, C9orf47-002, C9orf47-001 |
| chr11 | 31530551 | 31531500 | 950 | 14 | 5.22e-01 | -0.019 | -0.003 | IMMP1L-001, ELP4-001, ELP4-004, ELP4-003, ELP4-002, IMMP1L-012, IMMP1L-013, IMMP1L-004, IMMP1L-011, IMMP1L-006, IMMP1L-008, IMMP1L-002, IMMP1L-003, IMMP1L-010, IMMP1L-005, IMMP1L-007, IMMP1L-009 |
| chr5 | 1.08E+08 | 1.08E+08 | 1259 | 11 | 5.27e-01 | 0.11 | 0.005 | FER-001, FER-004, FER-008, FER-005, FER-003, FER-202 |
| chr6 | 30522620 | 30523426 | 807 | 11 | 5.33e-01 | 0.029 | 0.006 | PRR3-002, PRR3-001, GNL1-001, GNL1-002, PRR3-004, GNL1-003, PRR3-006, PRR3-005 |
| chr22 | 50585161 | 50585538 | 378 | 10 | 5.38e-01 | 0.047 | 0.015 | MOV10L1-005 |
| chr10 | 1.35E+08 | 1.35E+08 | 1141 | 13 | 5.43e-01 | 0.058 | -0.002 | MIR202HG-001, MIR202HG-201, MIR202HG-002 |
| chr22 | 42474656 | 42476080 | 1425 | 15 | 5.47e-01 | 0.03 | 0.003 | SMDT1-001, SMDT1-003, SMDT1-002 |
| chr1 | 11795409 | 11796573 | 1165 | 14 | 5.48e-01 | 0.046 | 0.006 | AGTRAP-001, AGTRAP-005, AGTRAP-002, AGTRAP-003, AGTRAP-010, AGTRAP-011, AGTRAP-008, AGTRAP-007, AGTRAP-004, AGTRAP-012 |
| chr12 | 56360065 | 56360681 | 617 | 11 | 5.50e-01 | 0.026 | 0.003 | CDK2-001, CDK2-002, PMEL-201, CDK2-003, PMEL-005, PMEL-202, PMEL-203, PMEL-013, PMEL-012, PMEL-002, PMEL-010, PMEL-001, PMEL-016, PMEL-014, PMEL-008, PMEL-009, PMEL-007, PMEL-004, PMEL-011, PMEL-003, CDK2-007, RP11-973D8.4-001, CDK2-005, CDK2-004, CDK2-006, CDK2-010, CDK2-011, CDK2-009, CDK2-012 |
| chr7 | 39605529 | 39606138 | 610 | 14 | 5.55e-01 | -0.029 | -0.007 | YAE1D1-001, YAE1D1-006, AC011290.4-001, YAE1D1-003, YAE1D1-004, YAE1D1-002 |
| chr3 | 1.72E+08 | 1.72E+08 | 582 | 13 | 5.59e-01 | 0.018 | 0.001 | NCEH1-201, NCEH1-003, NCEH1-002, NCEH1-001, NCEH1-202, NCEH1-203 |
| chr6 | 32013699 | 32015773 | 2075 | 33 | 5.75e-01 | 0.04 | -0.004 | TNXB-006, TNXB-005 |
| chr6 | 32135195 | 32136431 | 1237 | 17 | 5.75e-01 | 0.015 | 0.001 | EGFL8-005, EGFL8-006 |
| chr6 | 32097116 | 32098346 | 1231 | 32 | 5.76e-01 | -0.033 | 0.002 | FKBPL-001, ATF6B-001, ATF6B-002, ATF6B-004, ATF6B-005, ATF6B-003 |
| chr4 | 86395989 | 86396765 | 777 | 14 | 5.91e-01 | 0.015 | 0.003 | ARHGAP24-001, ARHGAP24-003, ARHGAP24-007, ARHGAP24-008 |
| chr6 | 42988445 | 42989600 | 1156 | 11 | 5.94e-01 | 0.044 | 0.009 | RRP36-001 |
| chr7 | 2556531 | 2557466 | 936 | 10 | 5.98e-01 | 0.042 | 0.006 | LFNG-201, LFNG-004 |
| chr12 | 6798753 | 6799378 | 626 | 10 | 6.02e-01 | 0.017 | 0.003 | ZNF384-201, ZNF384-003, ZNF384-004, ZNF384-202, ZNF384-002, ZNF384-001, ZNF384-018, ZNF384-011, ZNF384-017, ZNF384-012, ZNF384-007, ZNF384-016, ZNF384-013, ZNF384-006, ZNF384-014 |
| chr4 | 56502392 | 56503521 | 1130 | 16 | 6.11e-01 | 0.02 | 0.002 | NMU-001, NMU-005, NMU-003, NMU-002, NMU-006 |
| chr6 | 31589926 | 31590870 | 945 | 13 | 6.16e-01 | 0.048 | 0.016 | SNORA38-201, PRRC2A-001, PRRC2A-002, PRRC2A-013, PRRC2A-003 |
| chr11 | 78285742 | 78286346 | 605 | 12 | 6.22e-01 | 0.016 | 0.004 | NARS2-001, NARS2-002, NARS2-004, NARS2-005 |
| chr14 | 1.02E+08 | 1.02E+08 | 902 | 11 | 6.26e-01 | -0.022 | -0.01 | MIR654-201, MIR300-201, AL132709.2-201, MIR376A1-201, MIR376C-201 |
| chr10 | 1.35E+08 | 1.35E+08 | 1331 | 10 | 6.26e-01 | 0.03 | 0.005 | RPL5P28-001 |
| chr7 | 1.51E+08 | 1.51E+08 | 1030 | 14 | 6.31e-01 | 0.037 | 0.012 | ASB10-002, ASB10-003, ASB10-004, ASB10-001, ASB10-201, ASB10-202 |
| chr19 | 4790990 | 4791861 | 872 | 12 | 6.31e-01 | 0.023 | 0.007 | FEM1A-001, AC005523.3-001 |
| chr17 | 7323110 | 7323730 | 621 | 11 | 6.34e-01 | 0.04 | 0 | SPEM1-001, RP11-104H15.7-001 |
| chr7 | 1.02E+08 | 1.02E+08 | 309 | 10 | 6.34e-01 | 0.017 | 0.005 | FAM185A-003, FAM185A-009, FAM185A-004, FAM185A-001, FAM185A-002, FAM185A-006, FAM185A-007, FAM185A-005 |
| chr6 | 32819858 | 32820893 | 1036 | 22 | 6.35e-01 | -0.031 | -0.01 | TAP1-001, PSMB9-001, TAP1-201, PSMB9-202, PSMB9-002, TAP1-002 |
| chr20 | 36156471 | 36157675 | 1205 | 32 | 6.37e-01 | 0.086 | 0.007 | BLCAP-001, BLCAP-007, BLCAP-003, BLCAP-006, BLCAP-201, BLCAP-004, PPIAP3-001, BLCAP-002 |
| chr14 | 91720173 | 91720818 | 646 | 11 | 6.40e-01 | 0.02 | 0.009 | GPR68-201, GPR68-003, GPR68-001 |
| chr11 | 44972293 | 44973218 | 926 | 15 | 6.41e-01 | 0.031 | 0.005 | TP53I11-201, TP53I11-005, TP53I11-006, TP53I11-012, TP53I11-001, TP53I11-019, TP53I11-015, TP53I11-021, TP53I11-008, TP53I11-017, TP53I11-011, TP53I11-003, TP53I11-013, TP53I11-007, TP53I11-004, TP53I11-014 |
| chr6 | 33287504 | 33289719 | 2216 | 39 | 6.45e-01 | 0.046 | 0.005 | DAXX-002, DAXX-001, DAXX-201, ZBTB22-204, ZBTB22-001, ZBTB22-002, DAXX-007, DAXX-011, DAXX-009, DAXX-010, DAXX-004, DAXX-008, DAXX-003 |
| chr6 | 30458078 | 30459595 | 1518 | 21 | 6.56e-01 | -0.034 | -0.009 | HLA-E-001, HLA-E-002, HLA-E-003 |
| chr17 | 80545020 | 80545869 | 850 | 12 | 6.60e-01 | 0.084 | 0.019 | RP13-638C3.3-001, FOXK2-011 |
| chr8 | 22959776 | 22960619 | 844 | 11 | 6.65e-01 | 0.013 | 0.005 | TNFRSF10C-001, TNFRSF10C-004, TNFRSF10C-201 |
| chr3 | 46448496 | 46449636 | 1141 | 14 | 6.78e-01 | -0.027 | -0.003 | CCRL2-001, CCRL2-002, CCRL2-004, CCRL2-003, CCRL2-005, CCRL2-007, RP11-24F11.2-001, CCRL2-006 |
| chr1 | 63988814 | 63989705 | 892 | 14 | 6.79e-01 | -0.022 | 0.001 | ITGB3BP-017, ITGB3BP-201, EFCAB7-001, ITGB3BP-008, ITGB3BP-015, ITGB3BP-011, ITGB3BP-010, ITGB3BP-007, EFCAB7-003, ITGB3BP-014, ITGB3BP-006 |
| chr18 | 11850907 | 11851989 | 1083 | 13 | 6.99e-01 | -0.019 | 0.001 | CHMP1B-001, RP11-78A19.3-001, CHMP1B-002 |
| chr13 | 76123086 | 76123904 | 819 | 13 | 7.17e-01 | 0.01 | 0.002 | UCHL3-001, COMMD6-011, COMMD6-012, UCHL3-002, RP11-29G8.3-001 |
| chr17 | 1553202 | 1554549 | 1348 | 10 | 7.21e-01 | 0.022 | 0.007 | RILP-001, RILP-003, PRPF8-017, PRPF8-016, RILP-002, RILP-004 |
| chr3 | 1.01E+08 | 1.01E+08 | 1039 | 15 | 7.40e-01 | -0.018 | 0.004 | CEP97-201, CEP97-001, CEP97-004, CEP97-002, CEP97-003 |
| chr3 | 37034997 | 37035625 | 629 | 15 | 7.57e-01 | -0.026 | -0.002 | MLH1-001, EPM2AIP1-001, MLH1-016, MLH1-002, MLH1-006, MLH1-020, MLH1-013, MLH1-018, MLH1-005, MLH1-003, MLH1-012, MLH1-004, MLH1-019, MLH1-014, MLH1-017, MLH1-015, MLH1-201, MLH1-202 |
| chr10 | 93557741 | 93558457 | 717 | 11 | 7.78e-01 | -0.028 | -0.004 | TNKS2-001, TNKS2-AS1-002, TNKS2-AS1-001 |
| chr6 | 32051857 | 32052736 | 880 | 14 | 7.82e-01 | -0.018 | -0.008 |  |
| chr7 | 1.17E+08 | 1.17E+08 | 817 | 15 | 8.00e-01 | 0.041 | 0.007 | ST7-002, ST7-001, ST7-013, ST7-014, ST7-007, ST7-OT4-001, ST7-OT4-004, ST7-027, ST7-028, ST7-015, ST7-021, ST7-026, ST7-AS1-001, ST7-OT4-002, ST7-OT4-003, ST7-OT4-005 |
| chr3 | 25830573 | 25831643 | 1071 | 12 | 8.00e-01 | -0.034 | -0.006 | OXSM-001, NGLY1-008, OXSM-003, OXSM-007, OXSM-002, OXSM-008 |
| chr1 | 64058180 | 64059385 | 1206 | 19 | 8.07e-01 | 0.05 | 0.006 | PGM1-001, PGM1-003, ITGB3BP-009, PGM1-201 |
| chr6 | 33265133 | 33266407 | 1275 | 19 | 8.20e-01 | 0.019 | 0.006 | RGL2-009, RGL2-004, RGL2-204, RGL2-011, RGL2-007, RGL2-010, RGL2-002, RGL2-006, RGL2-005, RGL2-003, RGL2-001 |
| chr19 | 53897900 | 53898513 | 614 | 11 | 8.27e-01 | 0.018 | 0.004 | ZNF765-001, ZNF765-004, ZNF765-002, ZNF765-005, ZNF765-009 |
| chr17 | 79303434 | 79304649 | 1216 | 12 | 8.33e-01 | 0.05 | 0.006 | TMEM105-001, TMEM105-004, TMEM105-002, TMEM105-003, TMEM105-005 |
| chr6 | 33239487 | 33241026 | 1540 | 29 | 8.43e-01 | -0.037 | -0.002 | VPS52-201, RPS18-001, VPS52-001, VPS52-005, VPS52-004, RPS18-006, RPS18-002, RPS18-003, VPS52-002, VPS52-007, RPS18-005, RPS18-007 |
| chr10 | 77541764 | 77542585 | 822 | 11 | 8.48e-01 | 0.067 | 0.011 | C10orf11-001 |
| chr7 | 1.39E+08 | 1.39E+08 | 1762 | 11 | 8.72e-01 | 0.108 | 0.013 | KIAA1549-201 |
| chr13 | 36920644 | 36921174 | 531 | 13 | 9.09e-01 | -0.016 | 0 | SPG20OS-002, SPG20-002, SPG20-202, SPG20-009, SPG20OS-003, SPG20OS-001, SPG20-010, SPG20-011, SPG20-003 |
| chr14 | 24700477 | 24702094 | 1618 | 20 | 9.09e-01 | -0.037 | 0.003 | NEDD8-001, GMPR2-201, GMPR2-003, NEDD8-002, GMPR2-004, GMPR2-001, GMPR2-011, NEDD8-004, NEDD8-007, NEDD8-005, NEDD8-MDP1-004, NEDD8-006, NEDD8-003, NEDD8-MDP1-001, GMPR2-009, GMPR2-024, GMPR2-030, GMPR2-025, GMPR2-023, GMPR2-028, GMPR2-010, GMPR2-006, GMPR2-029, GMPR2-013, GMPR2-002, GMPR2-007, GMPR2-012, GMPR2-026, GMPR2-008, GMPR2-027, NEDD8-MDP1-003, NEDD8-MDP1-002 |
| chr3 | 28390637 | 28391118 | 482 | 11 | 9.10e-01 | 0.038 | 0.004 | AZI2-003, AZI2-014, ZCWPW2-002, AZI2-011, AZI2-013, ZCWPW2-001, AZI2-005, AZI2-009, AZI2-004, AZI2-001 |
| chr6 | 31619762 | 31620861 | 1100 | 31 | 9.33e-01 | -0.037 | 0.002 | BAG6-001, BAG6-202, APOM-003, APOM-002, BAG6-203, BAG6-003, BAG6-204, BAG6-011, BAG6-006, BAG6-014, BAG6-017, BAG6-018, BAG6-005, BAG6-004, BAG6-205, BAG6-019, BAG6-007, BAG6-013, BAG6-015, BAG6-016, BAG6-010 |
| chr8 | 74884292 | 74884910 | 619 | 18 | 9.37e-01 | 0.02 | 0.003 | TCEB1-007, TCEB1-004, TCEB1-009, TCEB1-006, TMEM70-007, TCEB1-003, TCEB1-001, TCEB1-010, TMEM70-006, TCEB1-008, TCEB1-005 |
| chr6 | 31831599 | 31833162 | 1564 | 24 | 9.47e-01 | 0.044 | 0.001 | NEU1-001, NEU1-004, SLC44A4-007, NEU1-002, NEU1-005 |
| chr6 | 32810551 | 32813715 | 3165 | 62 | 9.53e-01 | -0.065 | -0.001 | PSMB8-001, PSMB8-002, PSMB9-005, PSMB8-004, TAPSAR1-005, TAPSAR1-004, PSMB9-006, TAPSAR1-006, TAPSAR1-003, TAPSAR1-001, TAPSAR1-002, PSMB8-003, PSMB8-005 |
| chr11 | 72928926 | 72929382 | 457 | 11 | 9.66e-01 | 0.05 | 0.009 | P2RY2-001, P2RY2-003, P2RY2-002, RP11-800A3.2-001 |
| chr6 | 31860013 | 31860832 | 820 | 16 | 9.70e-01 | 0.052 | 0.003 | EHMT2-010 |
| chr6 | 31632477 | 31634890 | 2414 | 55 | 9.99e-01 | 0.024 | 0.004 | Y_RNA.248-201, CSNK2B-008, CSNK2B-009, CSNK2B-LY6G5B-1181-001, CSNK2B-001, CSNK2B-004, GPANK1-004, GPANK1-002, GPANK1-005, GPANK1-001, GPANK1-003, GPANK1-009, GPANK1-010, GPANK1-008, CSNK2B-005, CSNK2B-003, CSNK2B-010, CSNK2B-006 |

**Supplemental Table S3. Differentially Expressed Genes in CP Group between Day 1 and 14**

| **ENTREZID** | **SYMBOL** | **GENENAME** | **Log fold change** | **t-stat** | **p-value** | **adj p-value** |
| --- | --- | --- | --- | --- | --- | --- |
| 55204 | GOLPH3L | golgi phosphoprotein 3 like | -1.12783 | -9.13622 | 9.85E-14 | 2.22E-09 |
|  |  |  | -0.83628 | -7.87342 | 2.35E-11 | 1.7E-07 |
| 3792 | KEL | Kell metallo-endopeptidase (Kell blood group) | -0.81075 | -7.9473 | 1.71E-11 | 1.7E-07 |
| 84932 | RAB2B | RAB2B, member RAS oncogene family | -0.83356 | -7.81651 | 3.01E-11 | 1.7E-07 |
| 26127 | FGFR1OP2 | FGFR1 oncogene partner 2 | -0.79556 | -7.61438 | 7.22E-11 | 3.26E-07 |
| 317671 | RFESD | Rieske Fe-S domain containing | -1.12105 | -7.51068 | 1.13E-10 | 4.26E-07 |
| 130540 | FLACC1 | flagellum associated containing coiled-coil domains 1 | -0.6525 | -7.21938 | 3.97E-10 | 8.96E-07 |
| 26268 | FBXO9 | F-box protein 9 | -0.87382 | -7.25249 | 3.44E-10 | 8.96E-07 |
| 57515 | SERINC1 | serine incorporator 1 | -0.89461 | -7.23945 | 3.64E-10 | 8.96E-07 |
| 6451 | SH3BGRL | SH3 domain binding glutamate rich protein like | -0.63137 | -7.30067 | 2.8E-10 | 8.96E-07 |
| 129607 | CMPK2 | cytidine/uridine monophosphate kinase 2 | -0.77529 | -6.88938 | 1.63E-09 | 3.35E-06 |
| 10826 | FAXDC2 | fatty acid hydroxylase domain containing 2 | -0.78817 | -6.81619 | 2.23E-09 | 4.19E-06 |
| 3159 | HMGA1 | high mobility group AT-hook 1 | 0.571477 | 6.75841 | 2.85E-09 | 4.6E-06 |
| 9532 | BAG2 | BCL2 associated athanogene 2 | -0.64907 | -6.77415 | 2.66E-09 | 4.6E-06 |
| 10910 | SUGT1 | SGT1 homolog, MIS12 kinetochore complex assembly cochaperone | -0.70476 | -6.69409 | 3.74E-09 | 5.64E-06 |
| 10085 | EDIL3 | EGF like repeats and discoidin domains 3 | -0.86272 | -6.6576 | 4.37E-09 | 5.81E-06 |
| 23613 | ZMYND8 | zinc finger MYND-type containing 8 | -0.5127 | -6.66174 | 4.3E-09 | 5.81E-06 |
| 51706 | CYB5R1 | cytochrome b5 reductase 1 | -0.70463 | -6.52573 | 7.64E-09 | 8.71E-06 |
| 7504 | XK | X-linked Kx blood group | -0.86809 | -6.54125 | 7.15E-09 | 8.71E-06 |
| 92745 | SLC38A5 | solute carrier family 38 member 5 | -0.89169 | -6.52334 | 7.71E-09 | 8.71E-06 |
| 7029 | TFDP2 | transcription factor Dp-2 | -0.69474 | -6.42429 | 1.17E-08 | 1.26E-05 |
| 55553 | SOX6 | SRY-box 6 | -0.72363 | -6.28939 | 2.06E-08 | 2.02E-05 |
| 23762 | OSBP2 | oxysterol binding protein 2 | -0.75459 | -6.29171 | 2.04E-08 | 2.02E-05 |
| 8945 | BTRC | beta-transducin repeat containing E3 ubiquitin protein ligase | -0.63791 | -6.19172 | 3.1E-08 | 2.92E-05 |
| 10284 | SAP18 | Sin3A associated protein 18 | -0.58434 | -6.13412 | 3.94E-08 | 3.55E-05 |
| 1039 | CDR2 | cerebellar degeneration related protein 2 | 0.648297 | 6.125295 | 4.09E-08 | 3.55E-05 |
| 7381 | UQCRB | ubiquinol-cytochrome c reductase binding protein | -0.57196 | -6.07563 | 5.02E-08 | 4.11E-05 |
| 92521 | SPECC1 | sperm antigen with calponin homology and coiled-coil domains 1 | -0.66194 | -6.07209 | 5.1E-08 | 4.11E-05 |
| 93621 | MRFAP1 | Morf4 family associated protein 1 | -0.74219 | -6.03184 | 6.02E-08 | 4.69E-05 |
| 1340 | COX6B1 | cytochrome c oxidase subunit 6B1 | -0.39792 | -5.98724 | 7.24E-08 | 5.45E-05 |
| 5052 | PRDX1 | peroxiredoxin 1 | -0.67258 | -5.96276 | 8.01E-08 | 5.83E-05 |
| 7111 | TMOD1 | tropomodulin 1 | -0.65953 | -5.94366 | 8.66E-08 | 6.11E-05 |
| 11235 | PDCD10 | programmed cell death 10 | -0.50419 | -5.92445 | 9.38E-08 | 6.31E-05 |
| 23240 | TMEM131L | transmembrane 131 like | -0.47829 | -5.91767 | 9.64E-08 | 6.31E-05 |
| 84993 | UBL7 | ubiquitin like 7 | -0.44277 | -5.91424 | 9.78E-08 | 6.31E-05 |
| 4121 | MAN1A1 | mannosidase alpha class 1A member 1 | -0.60036 | -5.90207 | 1.03E-07 | 6.32E-05 |
| 10159 | ATP6AP2 | ATPase H+ transporting accessory protein 2 | -0.49866 | -5.90032 | 1.04E-07 | 6.32E-05 |
| 84232 | MAF1 | MAF1 homolog, negative regulator of RNA polymerase III | -0.41787 | -5.87256 | 1.16E-07 | 6.9E-05 |
| 117177 | RAB3IP | RAB3A interacting protein | -0.61806 | -5.82743 | 1.4E-07 | 8.09E-05 |
| 80896 | NPL | N-acetylneuraminate pyruvate lyase | -0.66179 | -5.80577 | 1.53E-07 | 8.4E-05 |
| 1510 | CTSE | cathepsin E | -1.31971 | -5.8059 | 1.52E-07 | 8.4E-05 |
| 966 | CD59 | CD59 molecule (CD59 blood group) | -0.76012 | -5.77142 | 1.76E-07 | 9.44E-05 |
| 8444 | DYRK3 | dual specificity tyrosine phosphorylation regulated kinase 3 | -0.94814 | -5.76017 | 1.84E-07 | 9.65E-05 |
| 83658 | DYNLRB1 | dynein light chain roadblock-type 1 | -0.45053 | -5.73415 | 2.04E-07 | 0.000105 |
| 84337 | ELOF1 | elongation factor 1 homolog | -0.69127 | -5.69042 | 2.44E-07 | 0.000123 |
| 79048 | SECISBP2 | SECIS binding protein 2 | -0.41551 | -5.65352 | 2.83E-07 | 0.000139 |
| 1173 | AP2M1 | adaptor related protein complex 2 subunit mu 1 | -0.40487 | -5.64679 | 2.91E-07 | 0.00014 |
| 1870 | E2F2 | E2F transcription factor 2 | -0.75478 | -5.62348 | 3.2E-07 | 0.000151 |
| 284904 | SEC14L4 | SEC14 like lipid binding 4 | -0.68246 | -5.61131 | 3.36E-07 | 0.000155 |
| 25996 | REXO2 | RNA exonuclease 2 | -0.75448 | -5.57985 | 3.82E-07 | 0.000172 |
| 653659 | TMEM183B | transmembrane protein 183B | -0.42313 | -5.57331 | 3.92E-07 | 0.000174 |
| 1656 | DDX6 | DEAD-box helicase 6 | -0.47326 | -5.56535 | 4.05E-07 | 0.000176 |
| 5532 | PPP3CB | protein phosphatase 3 catalytic subunit beta | -0.49934 | -5.52249 | 4.81E-07 | 0.000205 |
| 51021 | MRPS16 | mitochondrial ribosomal protein S16 | -0.43774 | -5.51812 | 4.9E-07 | 0.000205 |
| 27095 | TRAPPC3 | trafficking protein particle complex 3 | -0.50244 | -5.48758 | 5.53E-07 | 0.000227 |
| 760 | CA2 | carbonic anhydrase 2 | 0.591062 | 5.4739 | 5.85E-07 | 0.000236 |
| 6248 | RSC1A1 | regulator of solute carriers 1 | -0.44317 | -5.46052 | 6.17E-07 | 0.00024 |
| 51257 | MARCH2 | membrane associated ring-CH-type finger 2 | -0.59215 | -5.46346 | 6.1E-07 | 0.00024 |
| 55766 | H2AFJ | H2A histone family member J | -0.56653 | -5.43681 | 6.78E-07 | 0.00026 |
| 128989 | TANGO2 | transport and golgi organization 2 homolog | -0.49788 | -5.42743 | 7.04E-07 | 0.000265 |
| 29058 | TMEM230 | transmembrane protein 230 | -0.47337 | -5.40041 | 7.84E-07 | 0.00029 |
| 60313 | GPBP1L1 | GC-rich promoter binding protein 1 like 1 | -0.42663 | -5.36022 | 9.2E-07 | 0.000335 |
| 55363 | HEMGN | hemogen | -0.41506 | -5.32104 | 1.08E-06 | 0.000386 |
| 79071 | ELOVL6 | ELOVL fatty acid elongase 6 | -0.90112 | -5.30456 | 1.15E-06 | 0.000405 |
| 9604 | RNF14 | ring finger protein 14 | -0.65713 | -5.28892 | 1.22E-06 | 0.000424 |
| 2996 | GYPE | glycophorin E (MNS blood group) | -0.60387 | -5.28369 | 1.25E-06 | 0.000424 |
| 3934 | LCN2 | lipocalin 2 | 1.225071 | 5.281194 | 1.26E-06 | 0.000424 |
| 8317 | CDC7 | cell division cycle 7 | -0.63852 | -5.24207 | 1.47E-06 | 0.000488 |
| 5906 | RAP1A | RAP1A, member of RAS oncogene family | -0.51236 | -5.23743 | 1.5E-06 | 0.00049 |
| 55907 | CMAS | cytidine monophosphate N-acetylneuraminic acid synthetase | -0.96541 | -5.21569 | 1.63E-06 | 0.000526 |
| 90736 | FAM104B | family with sequence similarity 104 member B | -0.38743 | -5.20727 | 1.68E-06 | 0.000536 |
| 9240 | PNMA1 | PNMA family member 1 | 0.753109 | 5.166284 | 1.98E-06 | 0.000621 |
| 4750 | NEK1 | NIMA related kinase 1 | -0.3641 | -5.15126 | 2.1E-06 | 0.000649 |
| 162466 | PHOSPHO1 | phosphoethanolamine/phosphocholine phosphatase | -0.67863 | -5.13415 | 2.24E-06 | 0.000685 |
| 6117 | RPA1 | replication protein A1 | 0.512569 | 5.129978 | 2.28E-06 | 0.000687 |
| 1642 | DDB1 | damage specific DNA binding protein 1 | -0.73641 | -5.09463 | 2.62E-06 | 0.000778 |
| 1069 | CETN2 | centrin 2 | -0.58889 | -5.08856 | 2.68E-06 | 0.000786 |
| 10928 | RALBP1 | ralA binding protein 1 | -0.32027 | -5.06605 | 2.93E-06 | 0.000847 |
| 4893 | NRAS | NRAS proto-oncogene, GTPase | -0.43365 | -5.0416 | 3.22E-06 | 0.000914 |
| 6048 | RNF5 | ring finger protein 5 | -0.60915 | -5.0369 | 3.28E-06 | 0.000914 |
| 5609 | MAP2K7 | mitogen-activated protein kinase kinase 7 | -0.52153 | -5.03804 | 3.26E-06 | 0.000914 |
|  |  |  | 0.469452 | 5.032941 | 3.33E-06 | 0.000917 |
| 163 | AP2B1 | adaptor related protein complex 2 subunit beta 1 | -0.50305 | -5.02499 | 3.43E-06 | 0.000934 |
| 25780 | RASGRP3 | RAS guanyl releasing protein 3 | -1.22666 | -5.01266 | 3.6E-06 | 0.000957 |
| 55088 | CCDC186 | coiled-coil domain containing 186 | -0.51096 | -5.01516 | 3.57E-06 | 0.000957 |
|  |  |  | 0.314239 | 4.994689 | 3.86E-06 | 0.000958 |
|  |  |  | 0.314239 | 4.994689 | 3.86E-06 | 0.000958 |
|  |  |  | 0.314239 | 4.994689 | 3.86E-06 | 0.000958 |
|  |  |  | -0.55657 | -5.00254 | 3.74E-06 | 0.000958 |
| 23161 | SNX13 | sorting nexin 13 | -0.37689 | -5.00923 | 3.65E-06 | 0.000958 |
| 29068 | ZBTB44 | zinc finger and BTB domain containing 44 | -0.36224 | -4.9947 | 3.86E-06 | 0.000958 |
| 66008 | TRAK2 | trafficking kinesin protein 2 | -0.72508 | -4.98403 | 4.02E-06 | 0.000977 |
| 54977 | SLC25A38 | solute carrier family 25 member 38 | -0.77195 | -4.98601 | 3.99E-06 | 0.000977 |
| 4154 | MBNL1 | muscleblind like splicing regulator 1 | 0.308573 | 4.975357 | 4.16E-06 | 0.000993 |
| 219899 | TBCEL | tubulin folding cofactor E like | -0.4376 | -4.97154 | 4.22E-06 | 0.000993 |
| 10661 | KLF1 | Kruppel like factor 1 | -0.48228 | -4.97262 | 4.2E-06 | 0.000993 |
| 26100 | WIPI2 | WD repeat domain, phosphoinositide interacting 2 | -0.39682 | -4.9687 | 4.27E-06 | 0.000993 |
| 114625 | ERMAP | erythroblast membrane associated protein (Scianna blood group) | -0.65235 | -4.96587 | 4.31E-06 | 0.000994 |
| 23476 | BRD4 | bromodomain containing 4 | -0.31312 | -4.96054 | 4.4E-06 | 0.000994 |
| 10955 | SERINC3 | serine incorporator 3 | -0.40799 | -4.96085 | 4.4E-06 | 0.000994 |
| 3093 | UBE2K | ubiquitin conjugating enzyme E2 K | -0.33349 | -4.94221 | 4.72E-06 | 0.001046 |
| 1603 | DAD1 | defender against cell death 1 | -0.39854 | -4.94342 | 4.7E-06 | 0.001046 |
| 79018 | GID4 | GID complex subunit 4 homolog | -0.61678 | -4.9244 | 5.06E-06 | 0.001109 |
| 26036 | ZNF451 | zinc finger protein 451 | -0.4112 | -4.92067 | 5.13E-06 | 0.001115 |
|  |  |  | -0.60244 | -4.88986 | 5.78E-06 | 0.001144 |
|  |  |  | -0.60244 | -4.88986 | 5.78E-06 | 0.001144 |
|  |  |  | -0.60244 | -4.88986 | 5.78E-06 | 0.001144 |
|  |  |  | -0.60244 | -4.88986 | 5.78E-06 | 0.001144 |
|  |  |  | -0.60244 | -4.88986 | 5.78E-06 | 0.001144 |
|  |  |  | -0.60244 | -4.88986 | 5.78E-06 | 0.001144 |
| 25911 | DPCD | deleted in primary ciliary dyskinesia homolog (mouse) | -0.63061 | -4.90069 | 5.54E-06 | 0.001144 |
| 283450 | HECTD4 | HECT domain E3 ubiquitin protein ligase 4 | -0.61371 | -4.89853 | 5.59E-06 | 0.001144 |
| 1984 | EIF5A | eukaryotic translation initiation factor 5A | -0.50278 | -4.90389 | 5.47E-06 | 0.001144 |
| 9253 | NUMBL | NUMB like endocytic adaptor protein | 0.430526 | 4.908736 | 5.37E-06 | 0.001144 |
| 342618 | SLFN14 | schlafen family member 14 | -0.88254 | -4.88366 | 5.91E-06 | 0.001162 |
| 10580 | SORBS1 | sorbin and SH3 domain containing 1 | -0.42888 | -4.87606 | 6.09E-06 | 0.001186 |
| 286451 | YIPF6 | Yip1 domain family member 6 | -0.38367 | -4.87295 | 6.16E-06 | 0.00119 |
| 30968 | STOML2 | stomatin like 2 | -0.45479 | -4.87031 | 6.22E-06 | 0.001191 |
| 1508 | CTSB | cathepsin B | -0.64957 | -4.8642 | 6.37E-06 | 0.001209 |
| 9188 | DDX21 | DExD-box helicase 21 | 0.377834 | 4.856357 | 6.56E-06 | 0.001236 |
| 54585 | LZTFL1 | leucine zipper transcription factor like 1 | -0.80478 | -4.85095 | 6.7E-06 | 0.001251 |
| 153527 | ZMAT2 | zinc finger matrin-type 2 | -0.53162 | -4.84802 | 6.78E-06 | 0.001255 |
| 57095 | PITHD1 | PITH domain containing 1 | -0.74105 | -4.82673 | 7.35E-06 | 0.00135 |
|  |  |  | -0.83629 | -4.81578 | 7.66E-06 | 0.001396 |
| 210 | ALAD | aminolevulinate dehydratase | -0.46746 | -4.81216 | 7.77E-06 | 0.001404 |
| 22848 | AAK1 | AP2 associated kinase 1 | 0.566608 | 4.796786 | 8.24E-06 | 0.001476 |
| 80127 | BBOF1 | basal body orientation factor 1 | -0.69449 | -4.7895 | 8.47E-06 | 0.001506 |
| 759 | CA1 | carbonic anhydrase 1 | 2.481068 | 4.774649 | 8.96E-06 | 0.001581 |
| 6004 | RGS16 | regulator of G protein signaling 16 | -0.50445 | -4.77038 | 9.1E-06 | 0.001582 |
| 5274 | SERPINI1 | serpin family I member 1 | -1.1296 | -4.77116 | 9.08E-06 | 0.001582 |
| 23039 | XPO7 | exportin 7 | -0.41365 | -4.76172 | 9.41E-06 | 0.001622 |
| 221477 | C6orf89 | chromosome 6 open reading frame 89 | -0.28193 | -4.75 | 9.83E-06 | 0.001682 |
| 167153 | TENT2 | terminal nucleotidyltransferase 2 | -0.34591 | -4.74169 | 1.01E-05 | 0.001723 |
| 124540 | MSI2 | musashi RNA binding protein 2 | -0.46682 | -4.73377 | 1.05E-05 | 0.001762 |
|  |  |  | 0.465858 | 4.721079 | 1.1E-05 | 0.001808 |
|  |  |  | 0.465858 | 4.721079 | 1.1E-05 | 0.001808 |
|  |  |  | 0.465858 | 4.721079 | 1.1E-05 | 0.001808 |
| 7486 | WRN | WRN RecQ like helicase | -0.47303 | -4.71179 | 1.14E-05 | 0.001859 |
| 5868 | RAB5A | RAB5A, member RAS oncogene family | -0.57168 | -4.69731 | 1.2E-05 | 0.001938 |
| 100141515 | C17orf99 | chromosome 17 open reading frame 99 | -0.54469 | -4.69682 | 1.2E-05 | 0.001938 |
|  |  |  | -0.81164 | -4.68837 | 1.24E-05 | 0.001983 |
| 4676 | NAP1L4 | nucleosome assembly protein 1 like 4 | -0.35985 | -4.68703 | 1.25E-05 | 0.001983 |
| 25950 | RWDD3 | RWD domain containing 3 | -0.47062 | -4.67739 | 1.29E-05 | 0.002041 |
| 11227 | GALNT5 | polypeptide N-acetylgalactosaminyltransferase 5 | -0.90218 | -4.67336 | 1.31E-05 | 0.002058 |
| 7389 | UROD | uroporphyrinogen decarboxylase | -0.54391 | -4.66502 | 1.35E-05 | 0.002109 |
|  |  |  | -0.73065 | -4.65764 | 1.39E-05 | 0.002153 |
| 9232 | PTTG1 | PTTG1 regulator of sister chromatid separation, securin | -0.65365 | -4.64089 | 1.48E-05 | 0.002261 |
| 9070 | ASH2L | ASH2 like, histone lysine methyltransferase complex subunit | -0.35701 | -4.64208 | 1.48E-05 | 0.002261 |
| 8925 | HERC1 | HECT and RLD domain containing E3 ubiquitin protein ligase family member 1 | -0.34419 | -4.6347 | 1.52E-05 | 0.002299 |
|  |  |  | -0.21893 | -4.62189 | 1.59E-05 | 0.002303 |
| 5792 | PTPRF | protein tyrosine phosphatase receptor type F | -0.3716 | -4.62233 | 1.59E-05 | 0.002303 |
| 132660 | LIN54 | lin-54 DREAM MuvB core complex component | -0.44138 | -4.62838 | 1.55E-05 | 0.002303 |
| 7430 | EZR | ezrin | 0.536431 | 4.626824 | 1.56E-05 | 0.002303 |
| 26224 | FBXL3 | F-box and leucine rich repeat protein 3 | -0.5673 | -4.62826 | 1.55E-05 | 0.002303 |
| 7453 | WARS | tryptophanyl-tRNA synthetase | 0.801453 | 4.623266 | 1.58E-05 | 0.002303 |
| 63893 | UBE2O | ubiquitin conjugating enzyme E2 O | -0.76999 | -4.62268 | 1.59E-05 | 0.002303 |
| 7037 | TFRC | transferrin receptor | -0.5749 | -4.61402 | 1.64E-05 | 0.002357 |
| 222642 | TSPO2 | translocator protein 2 | -0.97609 | -4.60379 | 1.7E-05 | 0.002433 |
| 7358 | UGDH | UDP-glucose 6-dehydrogenase | 1.349794 | 4.598353 | 1.74E-05 | 0.002467 |
| 155 | ADRB3 | adrenoceptor beta 3 | -0.27388 | -4.58106 | 1.85E-05 | 0.002614 |
| 9911 | TMCC2 | transmembrane and coiled-coil domain family 2 | -0.72625 | -4.56935 | 1.93E-05 | 0.002714 |
|  |  |  | 0.437702 | 4.56483 | 1.97E-05 | 0.002726 |
|  |  |  | 0.437702 | 4.56483 | 1.97E-05 | 0.002726 |
| 10767 | HBS1L | HBS1 like translational GTPase | -0.41849 | -4.55845 | 2.01E-05 | 0.002774 |
| 4303 | FOXO4 | forkhead box O4 | -0.54509 | -4.54491 | 2.12E-05 | 0.002899 |
| 7328 | UBE2H | ubiquitin conjugating enzyme E2 H | -0.36475 | -4.53958 | 2.16E-05 | 0.002939 |
|  |  |  | -0.36698 | -4.52901 | 2.25E-05 | 0.003038 |
| 9320 | TRIP12 | thyroid hormone receptor interactor 12 | -0.28121 | -4.51314 | 2.38E-05 | 0.003202 |
| 37 | ACADVL | acyl-CoA dehydrogenase very long chain | 0.424818 | 4.509989 | 2.41E-05 | 0.00322 |
| 6917 | TCEA1 | transcription elongation factor A1 | 0.553705 | 4.497046 | 2.53E-05 | 0.003338 |
| 10519 | CIB1 | calcium and integrin binding 1 | 0.413073 | 4.498113 | 2.52E-05 | 0.003338 |
| 10048 | RANBP9 | RAN binding protein 9 | -0.47987 | -4.49453 | 2.55E-05 | 0.003349 |
| 57466 | SCAF4 | SR-related CTD associated factor 4 | -0.33784 | -4.47894 | 2.7E-05 | 0.003526 |
| 387521 | TMEM189 | transmembrane protein 189 | 0.468304 | 4.47342 | 2.76E-05 | 0.003578 |
| 8140 | SLC7A5 | solute carrier family 7 member 5 | -0.62588 | -4.47028 | 2.79E-05 | 0.003599 |
| 81627 | TRMT1L | tRNA methyltransferase 1 like | -0.48095 | -4.46812 | 2.81E-05 | 0.003607 |
| 254428 | SLC41A1 | solute carrier family 41 member 1 | -0.41988 | -4.463 | 2.86E-05 | 0.003634 |
| 16 | AARS | alanyl-tRNA synthetase | 0.805362 | 4.463388 | 2.86E-05 | 0.003634 |
| 2038 | EPB42 | erythrocyte membrane protein band 4.2 | -0.5138 | -4.45708 | 2.93E-05 | 0.003652 |
| 23450 | SF3B3 | splicing factor 3b subunit 3 | -0.54268 | -4.46 | 2.9E-05 | 0.003652 |
| 9663 | LPIN2 | lipin 2 | -0.466 | -4.45753 | 2.92E-05 | 0.003652 |
| 1981 | EIF4G1 | eukaryotic translation initiation factor 4 gamma 1 | 0.351707 | 4.448906 | 3.02E-05 | 0.003743 |
| 10899 | JTB | jumping translocation breakpoint | -0.30073 | -4.44299 | 3.08E-05 | 0.003804 |
| 53339 | BTBD1 | BTB domain containing 1 | 0.363987 | 4.441274 | 3.1E-05 | 0.003807 |
|  |  |  | -0.39747 | -4.43905 | 3.13E-05 | 0.003817 |
| 3050 | HBZ | hemoglobin subunit zeta | -1.263 | -4.43461 | 3.18E-05 | 0.003859 |
|  |  |  | -0.62477 | -4.42813 | 3.25E-05 | 0.003905 |
| 127733 | UBXN10 | UBX domain protein 10 | -0.50545 | -4.42651 | 3.27E-05 | 0.003905 |
| 55219 | MACO1 | macoilin 1 | -0.55245 | -4.42925 | 3.24E-05 | 0.003905 |
| 81688 | C6orf62 | chromosome 6 open reading frame 62 | -0.4241 | -4.42413 | 3.3E-05 | 0.003905 |
| 220972 | MARCH8 | membrane associated ring-CH-type finger 8 | -0.645 | -4.42552 | 3.29E-05 | 0.003905 |
| 25874 | MPC2 | mitochondrial pyruvate carrier 2 | -0.46459 | -4.4092 | 3.49E-05 | 0.004102 |
|  |  |  | -0.67941 | -4.4029 | 3.57E-05 | 0.004133 |
| 5695 | PSMB7 | proteasome subunit beta 7 | -0.37367 | -4.40369 | 3.56E-05 | 0.004133 |
| 29086 | BABAM1 | BRISC and BRCA1 A complex member 1 | -0.42544 | -4.40301 | 3.57E-05 | 0.004133 |
| 84191 | CIAO2A | cytosolic iron-sulfur assembly component 2A | -0.35574 | -4.39994 | 3.61E-05 | 0.004156 |
| 6923 | ELOB | elongin B | -0.33906 | -4.39744 | 3.64E-05 | 0.004165 |
| 1088 | CEACAM8 | carcinoembryonic antigen related cell adhesion molecule 8 | 0.502134 | 4.396557 | 3.65E-05 | 0.004165 |
|  |  |  | -0.68499 | -4.38952 | 3.75E-05 | 0.004252 |
| 83483 | PLVAP | plasmalemma vesicle associated protein | -0.99848 | -4.38447 | 3.82E-05 | 0.004309 |
|  |  |  | 0.291616 | 4.38171 | 3.85E-05 | 0.004331 |
| 820 | CAMP | cathelicidin antimicrobial peptide | 0.99335 | 4.37129 | 4E-05 | 0.004476 |
| 6223 | RPS19 | ribosomal protein S19 | 0.328861 | 4.360103 | 4.17E-05 | 0.004638 |
| 54809 | SAMD9 | sterile alpha motif domain containing 9 | -0.88158 | -4.34531 | 4.4E-05 | 0.004869 |
| 9377 | COX5A | cytochrome c oxidase subunit 5A | 0.338426 | 4.342243 | 4.45E-05 | 0.0049 |
| 29796 | UQCR10 | ubiquinol-cytochrome c reductase, complex III subunit X | -0.36777 | -4.33515 | 4.56E-05 | 0.005003 |
| 162427 | RETREG3 | reticulophagy regulator family member 3 | -0.41065 | -4.33257 | 4.61E-05 | 0.005025 |
| 23608 | MKRN1 | makorin ring finger protein 1 | -0.55464 | -4.32159 | 4.79E-05 | 0.005181 |
| 5870 | RAB6A | RAB6A, member RAS oncogene family | -0.51929 | -4.32143 | 4.79E-05 | 0.005181 |
| 6827 | SUPT4H1 | SPT4 homolog, DSIF elongation factor subunit | -0.34295 | -4.3182 | 4.85E-05 | 0.005217 |
| 10565 | ARFGEF1 | ADP ribosylation factor guanine nucleotide exchange factor 1 | 0.361097 | 4.305269 | 5.08E-05 | 0.005415 |
| 6535 | SLC6A8 | solute carrier family 6 member 8 | -0.46128 | -4.30625 | 5.06E-05 | 0.005415 |
| 9419 | CRIPT | CXXC repeat containing interactor of PDZ3 domain | -0.40788 | -4.30025 | 5.17E-05 | 0.005436 |
| 55691 | FRMD4A | FERM domain containing 4A | -0.57214 | -4.30149 | 5.15E-05 | 0.005436 |
| 64131 | XYLT1 | xylosyltransferase 1 | 0.318279 | 4.301236 | 5.16E-05 | 0.005436 |
| 10107 | TRIM10 | tripartite motif containing 10 | -0.61463 | -4.29678 | 5.24E-05 | 0.005457 |
| 5303 | PIN4 | peptidylprolyl cis/trans isomerase, NIMA-interacting 4 | -0.47137 | -4.29662 | 5.24E-05 | 0.005457 |
| 7114 | TMSB4X | thymosin beta 4 X-linked | 0.324282 | 4.284445 | 5.48E-05 | 0.005656 |
| 682 | BSG | basigin (Ok blood group) | -0.35178 | -4.28411 | 5.48E-05 | 0.005656 |
|  |  |  | -0.58249 | -4.27162 | 5.74E-05 | 0.005862 |
| 5203 | PFDN4 | prefoldin subunit 4 | -0.56167 | -4.27163 | 5.74E-05 | 0.005862 |
| 11236 | RNF139 | ring finger protein 139 | -0.45207 | -4.26972 | 5.78E-05 | 0.005876 |
| 1983 | EIF5 | eukaryotic translation initiation factor 5 | -0.43186 | -4.26136 | 5.95E-05 | 0.006027 |
| 10695 | CNPY3 | canopy FGF signaling regulator 3 | 0.264122 | 4.257624 | 6.03E-05 | 0.006081 |
| 10540 | DCTN2 | dynactin subunit 2 | 0.405346 | 4.254173 | 6.11E-05 | 0.00613 |
|  |  |  | 0.261431 | 4.251408 | 6.17E-05 | 0.006163 |
| 669 | BPGM | bisphosphoglycerate mutase | -0.28055 | -4.24559 | 6.3E-05 | 0.006249 |
| 6647 | SOD1 | superoxide dismutase 1 | -0.5097 | -4.24507 | 6.31E-05 | 0.006249 |
| 6006 | RHCE | Rh blood group CcEe antigens | -0.62428 | -4.2375 | 6.48E-05 | 0.006367 |
| 1347 | COX7A2 | cytochrome c oxidase subunit 7A2 | -0.44052 | -4.2374 | 6.48E-05 | 0.006367 |
| 522 | ATP5PF | ATP synthase peripheral stalk subunit F6 | -0.35052 | -4.22645 | 6.74E-05 | 0.006592 |
| 149840 | SHLD1 | shieldin complex subunit 1 | -0.587 | -4.22405 | 6.8E-05 | 0.00662 |
| 7700 | ZNF141 | zinc finger protein 141 | 0.330213 | 4.221095 | 6.87E-05 | 0.006646 |
|  |  |  | -0.50771 | -4.22059 | 6.88E-05 | 0.006646 |
| 4635 | MYL4 | myosin light chain 4 | -0.40117 | -4.21099 | 7.12E-05 | 0.006848 |
| 2549 | GAB1 | GRB2 associated binding protein 1 | -0.36753 | -4.20601 | 7.25E-05 | 0.006911 |
| 221545 | C6orf136 | chromosome 6 open reading frame 136 | 0.335017 | 4.206651 | 7.24E-05 | 0.006911 |
|  |  |  | -0.6519 | -4.19845 | 7.45E-05 | 0.00704 |
| 55108 | BSDC1 | BSD domain containing 1 | -0.35878 | -4.19882 | 7.44E-05 | 0.00704 |
| 7390 | UROS | uroporphyrinogen III synthase | -0.60553 | -4.18714 | 7.75E-05 | 0.007298 |
| 4057 | LTF | lactotransferrin | 1.325055 | 4.185311 | 7.81E-05 | 0.007316 |
| 4680 | CEACAM6 | carcinoembryonic antigen related cell adhesion molecule 6 | 0.381192 | 4.177387 | 8.03E-05 | 0.007493 |
| 8775 | NAPA | NSF attachment protein alpha | -0.44959 | -4.17533 | 8.09E-05 | 0.007517 |
| 4673 | NAP1L1 | nucleosome assembly protein 1 like 1 | 0.448993 | 4.169673 | 8.25E-05 | 0.007638 |
| 51506 | UFC1 | ubiquitin-fold modifier conjugating enzyme 1 | -0.31585 | -4.16505 | 8.39E-05 | 0.007722 |
| 22990 | PCNX1 | pecanex 1 | -0.35685 | -4.16429 | 8.41E-05 | 0.007722 |
| 84164 | ASCC2 | activating signal cointegrator 1 complex subunit 2 | -0.45166 | -4.15586 | 8.66E-05 | 0.007923 |
| 100130933 | SMIM6 | small integral membrane protein 6 | -0.20215 | -4.14716 | 8.93E-05 | 0.008138 |
| 9491 | PSMF1 | proteasome inhibitor subunit 1 | -0.42349 | -4.14597 | 8.97E-05 | 0.008139 |
| 9318 | COPS2 | COP9 signalosome subunit 2 | -0.41864 | -4.13887 | 9.2E-05 | 0.008312 |
| 54497 | HEATR5B | HEAT repeat containing 5B | -0.30855 | -4.12832 | 9.55E-05 | 0.008593 |
| 6001 | RGS10 | regulator of G protein signaling 10 | -0.29439 | -4.12229 | 9.75E-05 | 0.008708 |
| 51573 | GDE1 | glycerophosphodiester phosphodiesterase 1 | -0.52359 | -4.12259 | 9.74E-05 | 0.008708 |
| 352954 | CASTOR3 | CASTOR family member 3 | -0.49464 | -4.11872 | 9.88E-05 | 0.008784 |
| 55633 | TBC1D22B | TBC1 domain family member 22B | -0.45413 | -4.11169 | 0.000101 | 0.008969 |
| 3145 | HMBS | hydroxymethylbilane synthase | -0.70689 | -4.10787 | 0.000103 | 0.009054 |
| 8498 | RANBP3 | RAN binding protein 3 | 0.220229 | 4.106108 | 0.000103 | 0.009075 |
| 400569 | MED11 | mediator complex subunit 11 | 0.260874 | 4.104049 | 0.000104 | 0.009084 |
| 5688 | PSMA7 | proteasome subunit alpha 7 | 0.310298 | 4.103636 | 0.000104 | 0.009084 |
| 10975 | UQCR11 | ubiquinol-cytochrome c reductase, complex III subunit XI | -0.26962 | -4.10194 | 0.000105 | 0.009103 |
| 23633 | KPNA6 | karyopherin subunit alpha 6 | -0.31877 | -4.09395 | 0.000108 | 0.009326 |
| 23091 | ZC3H13 | zinc finger CCCH-type containing 13 | -0.26042 | -4.0836 | 0.000112 | 0.009634 |
|  |  |  | 0.236154 | 4.081237 | 0.000113 | 0.00964 |
| 25853 | DCAF12 | DDB1 and CUL4 associated factor 12 | -0.35692 | -4.08127 | 0.000113 | 0.00964 |
| 116988 | AGAP3 | ArfGAP with GTPase domain, ankyrin repeat and PH domain 3 | 0.26547 | 4.077021 | 0.000114 | 0.009747 |
| 389860 | PAGE2B | PAGE family member 2B | -0.47695 | -4.07062 | 0.000117 | 0.00993 |
| 55544 | RBM38 | RNA binding motif protein 38 | -0.45174 | -4.06849 | 0.000118 | 0.009967 |
| 51629 | SLC25A39 | solute carrier family 25 member 39 | -0.33624 | -4.06214 | 0.00012 | 0.010153 |
| 996 | CDC27 | cell division cycle 27 | -0.32352 | -4.0553 | 0.000123 | 0.010283 |
| 9352 | TXNL1 | thioredoxin like 1 | -0.39027 | -4.05719 | 0.000123 | 0.010283 |
| 598 | BCL2L1 | BCL2 like 1 | -0.40638 | -4.05558 | 0.000123 | 0.010283 |
| 6947 | TCN1 | transcobalamin 1 | 0.802209 | 4.05381 | 0.000124 | 0.010299 |
| 25844 | YIPF3 | Yip1 domain family member 3 | -0.39446 | -4.05031 | 0.000126 | 0.01032 |
| 57410 | SCYL1 | SCY1 like pseudokinase 1 | 0.28717 | 4.051783 | 0.000125 | 0.01032 |
| 23204 | ARL6IP1 | ADP ribosylation factor like GTPase 6 interacting protein 1 | -0.45427 | -4.05007 | 0.000126 | 0.01032 |
| 22818 | COPZ1 | coatomer protein complex subunit zeta 1 | -0.37283 | -4.04378 | 0.000128 | 0.010511 |
| 1778 | DYNC1H1 | dynein cytoplasmic 1 heavy chain 1 | 0.379223 | 4.037823 | 0.000131 | 0.010692 |
| 5649 | RELN | reelin | -0.65908 | -4.02769 | 0.000136 | 0.011036 |
| 80323 | CCDC68 | coiled-coil domain containing 68 | -0.55516 | -4.02353 | 0.000138 | 0.011157 |
| 2993 | GYPA | glycophorin A (MNS blood group) | -0.28741 | -4.01982 | 0.00014 | 0.011261 |
| 54926 | UBE2R2 | ubiquitin conjugating enzyme E2 R2 | 0.311972 | 4.014748 | 0.000142 | 0.011421 |
|  |  |  | -0.42713 | -4.01032 | 0.000144 | 0.011557 |
| 116228 | COX20 | cytochrome c oxidase assembly factor COX20 | -0.41357 | -4.0024 | 0.000148 | 0.011759 |
| 203569 | PAGE2 | PAGE family member 2 | -0.47021 | -4.00157 | 0.000149 | 0.011759 |
| 6356 | CCL11 | C-C motif chemokine ligand 11 | 0.287919 | 4.00126 | 0.000149 | 0.011759 |
| 94103 | ORMDL3 | ORMDL sphingolipid biosynthesis regulator 3 | -0.51116 | -4.00419 | 0.000147 | 0.011759 |
| 932 | MS4A3 | membrane spanning 4-domains A3 | 0.735714 | 3.996822 | 0.000151 | 0.011899 |
| 10467 | ZNHIT1 | zinc finger HIT-type containing 1 | -0.40051 | -3.99409 | 0.000153 | 0.011971 |
| 3927 | LASP1 | LIM and SH3 protein 1 | 0.313706 | 3.987032 | 0.000156 | 0.012224 |
|  |  |  | 0.510294 | 3.983496 | 0.000158 | 0.012332 |
| 115123 | MARCH3 | membrane associated ring-CH-type finger 3 | -0.40564 | -3.9821 | 0.000159 | 0.012349 |
| 204851 | HIPK1 | homeodomain interacting protein kinase 1 | -0.2871 | -3.98094 | 0.00016 | 0.012357 |
| 10487 | CAP1 | cyclase associated actin cytoskeleton regulatory protein 1 | 0.241593 | 3.961928 | 0.000171 | 0.01315 |
| 54970 | TTC12 | tetratricopeptide repeat domain 12 | 0.3134 | 3.955281 | 0.000175 | 0.013408 |
| 9829 | DNAJC6 | DnaJ heat shock protein family (Hsp40) member C6 | -0.35921 | -3.95099 | 0.000177 | 0.013562 |
| 1349 | COX7B | cytochrome c oxidase subunit 7B | -0.4743 | -3.9495 | 0.000178 | 0.013585 |
| 51646 | YPEL5 | yippee like 5 | -0.34998 | -3.94589 | 0.00018 | 0.013709 |
| 6500 | SKP1 | S-phase kinase associated protein 1 | -0.48996 | -3.93933 | 0.000184 | 0.013846 |
| 4860 | PNP | purine nucleoside phosphorylase | -0.53332 | -3.94182 | 0.000183 | 0.013846 |
| 23765 | IL17RA | interleukin 17 receptor A | -0.25356 | -3.9391 | 0.000185 | 0.013846 |
| 642757 | TBC1D22A-AS1 | TBC1D22A antisense RNA 1 | 0.27525 | 3.940483 | 0.000184 | 0.013846 |
| 10124 | ARL4A | ADP ribosylation factor like GTPase 4A | -0.51149 | -3.93106 | 0.00019 | 0.014047 |
|  |  |  | -0.49015 | -3.93237 | 0.000189 | 0.014047 |
|  |  |  | 0.404159 | 3.931075 | 0.00019 | 0.014047 |
|  |  |  | -0.61596 | -3.93359 | 0.000188 | 0.014047 |
| 1608 | DGKG | diacylglycerol kinase gamma | 0.421257 | 3.926047 | 0.000193 | 0.014244 |
| 54823 | SWT1 | SWT1 RNA endoribonuclease homolog | -0.41171 | -3.92139 | 0.000196 | 0.01438 |
|  |  |  | -0.577 | -3.9219 | 0.000196 | 0.01438 |
| 1436 | CSF1R | colony stimulating factor 1 receptor | 0.438001 | 3.919991 | 0.000197 | 0.014402 |
| 1465 | CSRP1 | cysteine and glycine rich protein 1 | 0.270348 | 3.910005 | 0.000204 | 0.014675 |
| 100129094 | BTNL10 | butyrophilin like 10 | -0.39244 | -3.90996 | 0.000204 | 0.014675 |
| 51652 | CHMP3 | charged multivesicular body protein 3 | -0.30202 | -3.91068 | 0.000203 | 0.014675 |
| 89910 | UBE3B | ubiquitin protein ligase E3B | -0.41342 | -3.91017 | 0.000204 | 0.014675 |
| 26258 | BLOC1S6 | biogenesis of lysosomal organelles complex 1 subunit 6 | -0.40881 | -3.90983 | 0.000204 | 0.014675 |
| 1727 | CYB5R3 | cytochrome b5 reductase 3 | -0.47862 | -3.90779 | 0.000205 | 0.014731 |
|  |  |  | -0.50721 | -3.90337 | 0.000209 | 0.014907 |
| 2950 | GSTP1 | glutathione S-transferase pi 1 | 0.466608 | 3.900304 | 0.000211 | 0.015017 |
| 7036 | TFR2 | transferrin receptor 2 | -0.31767 | -3.89898 | 0.000212 | 0.015038 |
| 8724 | SNX3 | sorting nexin 3 | -0.31216 | -3.89001 | 0.000218 | 0.015408 |
| 3190 | HNRNPK | heterogeneous nuclear ribonucleoprotein K | -0.30302 | -3.89017 | 0.000218 | 0.015408 |
| 6158 | RPL28 | ribosomal protein L28 | 0.346833 | 3.887405 | 0.00022 | 0.015498 |
| 10277 | UBE4B | ubiquitination factor E4B | -0.31172 | -3.87618 | 0.000229 | 0.015963 |
| 9782 | MATR3 | matrin 3 | 0.246699 | 3.875989 | 0.000229 | 0.015963 |
|  |  |  | -0.57923 | -3.87688 | 0.000228 | 0.015963 |
| 9334 | B4GALT5 | beta-1,4-galactosyltransferase 5 | 0.351221 | 3.8745 | 0.00023 | 0.015995 |
| 149041 | RC3H1 | ring finger and CCCH-type domains 1 | -0.31632 | -3.87241 | 0.000232 | 0.01606 |
| 1350 | COX7C | cytochrome c oxidase subunit 7C | -0.28035 | -3.86842 | 0.000235 | 0.016168 |
| 146722 | CD300LF | CD300 molecule like family member f | 0.259754 | 3.868697 | 0.000235 | 0.016168 |
| 25941 | TPGS2 | tubulin polyglutamylase complex subunit 2 | -0.3878 | -3.86775 | 0.000236 | 0.016168 |
| 23274 | CLEC16A | C-type lectin domain containing 16A | -0.39616 | -3.86467 | 0.000238 | 0.016289 |
| 27257 | LSM1 | LSM1 homolog, mRNA degradation associated | -0.32718 | -3.85785 | 0.000244 | 0.01662 |
| 27109 | DMAC2L | distal membrane arm assembly complex 2 like | -0.45923 | -3.85559 | 0.000245 | 0.016698 |
| 5884 | RAD17 | RAD17 checkpoint clamp loader component | -0.40583 | -3.85336 | 0.000247 | 0.016724 |
| 81689 | ISCA1 | iron-sulfur cluster assembly 1 | -0.45856 | -3.85339 | 0.000247 | 0.016724 |
| 8818 | DPM2 | dolichyl-phosphate mannosyltransferase subunit 2, regulatory | -0.39053 | -3.84423 | 0.000255 | 0.017199 |
| 57545 | CC2D2A | coiled-coil and C2 domain containing 2A | 0.31208 | 3.8421 | 0.000257 | 0.017272 |
| 54855 | TENT5C | terminal nucleotidyltransferase 5C | -0.31299 | -3.8378 | 0.000261 | 0.017402 |
| 689 | BTF3 | basic transcription factor 3 | -0.27235 | -3.83647 | 0.000262 | 0.017402 |
| 26130 | GAPVD1 | GTPase activating protein and VPS9 domains 1 | -0.45474 | -3.83674 | 0.000262 | 0.017402 |
| 50616 | IL22 | interleukin 22 | 0.333052 | 3.836393 | 0.000262 | 0.017402 |
| 220594 | USP32P2 | ubiquitin specific peptidase 32 pseudogene 2 | -0.27462 | -3.83478 | 0.000263 | 0.017446 |
| 54439 | RBM27 | RNA binding motif protein 27 | -0.21585 | -3.83031 | 0.000267 | 0.017636 |
| 25897 | RNF19A | ring finger protein 19A, RBR E3 ubiquitin protein ligase | -0.45564 | -3.82985 | 0.000268 | 0.017636 |
|  |  |  | -0.49571 | -3.8284 | 0.000269 | 0.017639 |
| 11007 | CCDC85B | coiled-coil domain containing 85B | 0.229965 | 3.828069 | 0.000269 | 0.017639 |
| 164091 | PAQR7 | progestin and adipoQ receptor family member 7 | 0.328394 | 3.813324 | 0.000283 | 0.01843 |
| 55437 | STRADB | STE20 related adaptor beta | -0.327 | -3.81256 | 0.000284 | 0.01843 |
| 27102 | EIF2AK1 | eukaryotic translation initiation factor 2 alpha kinase 1 | -0.31023 | -3.81252 | 0.000284 | 0.01843 |
| 10150 | MBNL2 | muscleblind like splicing regulator 2 | 0.587529 | 3.811456 | 0.000285 | 0.018444 |
| 1054 | CEBPG | CCAAT enhancer binding protein gamma | 0.587353 | 3.801273 | 0.000295 | 0.019033 |
| 2879 | GPX4 | glutathione peroxidase 4 | 0.715203 | 3.79929 | 0.000297 | 0.019106 |
| 6794 | STK11 | serine/threonine kinase 11 | -0.3206 | -3.79704 | 0.000299 | 0.019197 |
| 23186 | RCOR1 | REST corepressor 1 | 0.478221 | 3.794776 | 0.000301 | 0.019234 |
| 10347 | ABCA7 | ATP binding cassette subfamily A member 7 | -0.40568 | -3.79481 | 0.000301 | 0.019234 |
| 8061 | FOSL1 | FOS like 1, AP-1 transcription factor subunit | 0.313832 | 3.792495 | 0.000304 | 0.019328 |
| 432369 | ATP5F1EP2 | ATP synthase F1 subunit epsilon pseudogene 2 | -0.27988 | -3.78504 | 0.000311 | 0.019763 |
| 51082 | POLR1D | RNA polymerase I and III subunit D | -0.4159 | -3.78127 | 0.000315 | 0.019959 |
| 84967 | LSM10 | LSM10, U7 small nuclear RNA associated | 0.322659 | 3.776748 | 0.00032 | 0.020095 |
| 785 | CACNB4 | calcium voltage-gated channel auxiliary subunit beta 4 | 0.307391 | 3.777181 | 0.00032 | 0.020095 |
| 64770 | CCDC14 | coiled-coil domain containing 14 | -0.26989 | -3.77681 | 0.00032 | 0.020095 |
| 3611 | ILK | integrin linked kinase | 0.289213 | 3.771726 | 0.000326 | 0.02038 |
| 9103 | FCGR2C | Fc fragment of IgG receptor IIc (gene/pseudogene) | 0.503079 | 3.767152 | 0.000331 | 0.020468 |
| 253012 | HEPACAM2 | HEPACAM family member 2 | -0.95627 | -3.76751 | 0.00033 | 0.020468 |
| 28977 | MRPL42 | mitochondrial ribosomal protein L42 | -0.47754 | -3.76926 | 0.000328 | 0.020468 |
| 57591 | MRTFA | myocardin related transcription factor A | 0.232305 | 3.767763 | 0.00033 | 0.020468 |
| 57577 | CCDC191 | coiled-coil domain containing 191 | 0.320248 | 3.764589 | 0.000334 | 0.020588 |
|  |  |  | 0.463925 | 3.759009 | 0.00034 | 0.020919 |
| 6622 | SNCA | synuclein alpha | -0.28737 | -3.75657 | 0.000343 | 0.021034 |
| 63877 | FAM204A | family with sequence similarity 204 member A | -0.2638 | -3.75398 | 0.000346 | 0.021128 |
| 4793 | NFKBIB | NFKB inhibitor beta | 0.299231 | 3.75361 | 0.000346 | 0.021128 |
| 10930 | APOBEC2 | apolipoprotein B mRNA editing enzyme catalytic subunit 2 | -0.27159 | -3.75064 | 0.00035 | 0.021282 |
| 59286 | UBL5 | ubiquitin like 5 | -0.39858 | -3.74558 | 0.000356 | 0.021586 |
| 92017 | SNX29 | sorting nexin 29 | 0.297356 | 3.743747 | 0.000358 | 0.02166 |
| 57553 | MICAL3 | microtubule associated monooxygenase, calponin and LIM domain containing 3 | -0.42295 | -3.7418 | 0.00036 | 0.021743 |
| 7265 | TTC1 | tetratricopeptide repeat domain 1 | -0.40138 | -3.74021 | 0.000362 | 0.021801 |
| 10576 | CCT2 | chaperonin containing TCP1 subunit 2 | -0.32991 | -3.73906 | 0.000363 | 0.021827 |
| 56829 | ZC3HAV1 | zinc finger CCCH-type containing, antiviral 1 | -0.33142 | -3.73749 | 0.000365 | 0.021883 |
| 10438 | C1D | C1D nuclear receptor corepressor | -0.58155 | -3.7348 | 0.000369 | 0.022021 |
| 728358 | DEFA1B | defensin alpha 1B | 1.531893 | 3.7286 | 0.000376 | 0.022363 |
| 1667 | DEFA1 | defensin alpha 1 | 1.531893 | 3.7286 | 0.000376 | 0.022363 |
| 51043 | ZBTB7B | zinc finger and BTB domain containing 7B | 0.35546 | 3.725948 | 0.00038 | 0.022502 |
| 100132074 | FOXO6 | forkhead box O6 | -0.22279 | -3.72443 | 0.000381 | 0.022557 |
| 8642 | DCHS1 | dachsous cadherin-related 1 | -0.35542 | -3.72354 | 0.000383 | 0.022565 |
| 3386 | ICAM4 | intercellular adhesion molecule 4 (Landsteiner-Wiener blood group) | -0.43179 | -3.72069 | 0.000386 | 0.022705 |
| 6510 | SLC1A5 | solute carrier family 1 member 5 | -0.37471 | -3.72012 | 0.000387 | 0.022705 |
| 6161 | RPL32 | ribosomal protein L32 | 0.22215 | 3.717407 | 0.000391 | 0.022818 |
| 2109 | ETFB | electron transfer flavoprotein subunit beta | 0.340316 | 3.717063 | 0.000391 | 0.022818 |
| 63914 | LINC01590 | long intergenic non-protein coding RNA 1590 | 0.294235 | 3.716171 | 0.000392 | 0.022827 |
| 1848 | DUSP6 | dual specificity phosphatase 6 | 0.562666 | 3.71136 | 0.000398 | 0.023064 |
| 23191 | CYFIP1 | cytoplasmic FMR1 interacting protein 1 | 0.275967 | 3.711125 | 0.000399 | 0.023064 |
|  |  |  | -0.47257 | -3.71075 | 0.000399 | 0.023064 |
| 137886 | UBXN2B | UBX domain protein 2B | -0.30285 | -3.70773 | 0.000403 | 0.023237 |
| 23142 | DCUN1D4 | defective in cullin neddylation 1 domain containing 4 | -0.31714 | -3.70621 | 0.000405 | 0.023244 |
| 9221 | NOLC1 | nucleolar and coiled-body phosphoprotein 1 | 0.283515 | 3.706111 | 0.000405 | 0.023244 |
| 57552 | NCEH1 | neutral cholesterol ester hydrolase 1 | -0.52089 | -3.70051 | 0.000413 | 0.02362 |
| 5082 | PDCL | phosducin like | -0.37932 | -3.69933 | 0.000415 | 0.023653 |
| 9588 | PRDX6 | peroxiredoxin 6 | -0.35628 | -3.69834 | 0.000416 | 0.02367 |
| 2115 | ETV1 | ETS variant 1 | 0.302592 | 3.695918 | 0.000419 | 0.023802 |
| 286 | ANK1 | ankyrin 1 | -0.40841 | -3.6941 | 0.000422 | 0.023886 |
| 157773 | C8orf48 | chromosome 8 open reading frame 48 | -0.2193 | -3.68916 | 0.000429 | 0.024142 |
| 10247 | RIDA | reactive intermediate imine deaminase A homolog | -0.48952 | -3.68861 | 0.00043 | 0.024142 |
| 84333 | PCGF5 | polycomb group ring finger 5 | -0.38097 | -3.69008 | 0.000428 | 0.024142 |
| 1611 | DAP | death associated protein | 0.304379 | 3.68378 | 0.000437 | 0.02447 |
| 3590 | IL11RA | interleukin 11 receptor subunit alpha | -0.18162 | -3.68208 | 0.000439 | 0.024547 |
| 19 | ABCA1 | ATP binding cassette subfamily A member 1 | 0.556248 | 3.680263 | 0.000442 | 0.024634 |
| 6563 | SLC14A1 | solute carrier family 14 member 1 (Kidd blood group) | -0.67758 | -3.67934 | 0.000443 | 0.024649 |
| 51271 | UBAP1 | ubiquitin associated protein 1 | -0.39333 | -3.67715 | 0.000446 | 0.024767 |
| 5634 | PRPS2 | phosphoribosyl pyrophosphate synthetase 2 | 0.254409 | 3.676351 | 0.000447 | 0.024772 |
| 644100 | ARL14EPL | ADP ribosylation factor like GTPase 14 effector protein like | 0.241876 | 3.674192 | 0.000451 | 0.024888 |
| 2876 | GPX1 | glutathione peroxidase 1 | -0.85068 | -3.66863 | 0.000459 | 0.025246 |
| 7314 | UBB | ubiquitin B | -0.15263 | -3.66839 | 0.000459 | 0.025246 |
| 4218 | RAB8A | RAB8A, member RAS oncogene family | 0.288039 | 3.666442 | 0.000462 | 0.025347 |
| 23390 | ZDHHC17 | zinc finger DHHC-type containing 17 | -0.42154 | -3.66546 | 0.000464 | 0.025367 |
| 8565 | YARS | tyrosyl-tRNA synthetase | 0.469243 | 3.664046 | 0.000466 | 0.025425 |
|  |  |  | -0.35049 | -3.66128 | 0.00047 | 0.02557 |
| 11282 | MGAT4B | alpha-1,3-mannosyl-glycoprotein 4-beta-N-acetylglucosaminyltransferase B | 0.286635 | 3.66013 | 0.000472 | 0.02557 |
| 90737 | PAGE5 | PAGE family member 5 | -0.33913 | -3.66054 | 0.000471 | 0.02557 |
| 2553 | GABPB1 | GA binding protein transcription factor subunit beta 1 | -0.28451 | -3.65863 | 0.000474 | 0.025635 |
| 1371 | CPOX | coproporphyrinogen oxidase | -0.43683 | -3.65556 | 0.000479 | 0.025759 |
| 8861 | LDB1 | LIM domain binding 1 | 0.207062 | 3.654475 | 0.000481 | 0.025759 |
| 8714 | ABCC3 | ATP binding cassette subfamily C member 3 | 0.418759 | 3.654279 | 0.000481 | 0.025759 |
| 84923 | FAM104A | family with sequence similarity 104 member A | -0.41377 | -3.65642 | 0.000478 | 0.025759 |
| 9902 | MRC2 | mannose receptor C type 2 | -0.38278 | -3.64962 | 0.000489 | 0.026095 |
| 55222 | LRRC20 | leucine rich repeat containing 20 | -0.20922 | -3.64773 | 0.000492 | 0.026134 |
| 85019 | TMEM241 | transmembrane protein 241 | 0.515468 | 3.648358 | 0.000491 | 0.026134 |
| 1488 | CTBP2 | C-terminal binding protein 2 | -0.36863 | -3.64642 | 0.000494 | 0.026186 |
| 2874 | GPS2 | G protein pathway suppressor 2 | -0.30136 | -3.64158 | 0.000502 | 0.026543 |
| 6708 | SPTA1 | spectrin alpha, erythrocytic 1 | -0.95633 | -3.64022 | 0.000504 | 0.0266 |
| 5498 | PPOX | protoporphyrinogen oxidase | -0.35771 | -3.63638 | 0.00051 | 0.026829 |
| 1536 | CYBB | cytochrome b-245 beta chain | 0.37907 | 3.635483 | 0.000512 | 0.026829 |
| 4778 | NFE2 | nuclear factor, erythroid 2 | -0.37256 | -3.63618 | 0.000511 | 0.026829 |
| 1612 | DAPK1 | death associated protein kinase 1 | 0.333262 | 3.634077 | 0.000514 | 0.02689 |
|  |  |  | 0.58228 | 3.632898 | 0.000516 | 0.026932 |
| 6633 | SNRPD2 | small nuclear ribonucleoprotein D2 polypeptide | 0.252414 | 3.628847 | 0.000523 | 0.02723 |
| 10569 | SLU7 | SLU7 homolog, splicing factor | -0.30684 | -3.62726 | 0.000526 | 0.027246 |
| 1965 | EIF2S1 | eukaryotic translation initiation factor 2 subunit alpha | 0.350672 | 3.62738 | 0.000526 | 0.027246 |
| 1317 | SLC31A1 | solute carrier family 31 member 1 | -0.38663 | -3.62035 | 0.000538 | 0.027807 |
| 80232 | WDR26 | WD repeat domain 26 | -0.33245 | -3.61638 | 0.000545 | 0.028106 |
| 4317 | MMP8 | matrix metallopeptidase 8 | 1.256637 | 3.611701 | 0.000553 | 0.028475 |
| 3344 | FOXN2 | forkhead box N2 | 0.244698 | 3.608976 | 0.000558 | 0.028664 |
| 4891 | SLC11A2 | solute carrier family 11 member 2 | -0.4354 | -3.60757 | 0.000561 | 0.028731 |
| 64755 | C16orf58 | chromosome 16 open reading frame 58 | -0.19473 | -3.60661 | 0.000563 | 0.028756 |
| 3021 | H3F3B | H3 histone family member 3B | 0.325062 | 3.60358 | 0.000568 | 0.028977 |
| 439 | ASNA1 | arsA arsenite transporter, ATP-binding, homolog 1 (bacterial) | -0.35526 | -3.59915 | 0.000577 | 0.029333 |
| 81565 | NDEL1 | nudE neurodevelopment protein 1 like 1 | -0.52438 | -3.59797 | 0.000579 | 0.02938 |
| 23410 | SIRT3 | sirtuin 3 | 0.325673 | 3.596681 | 0.000581 | 0.029437 |
| 391114 | OR6K3 | olfactory receptor family 6 subfamily K member 3 | 0.345934 | 3.593593 | 0.000587 | 0.029603 |
| 23328 | SASH1 | SAM and SH3 domain containing 1 | 0.295557 | 3.593748 | 0.000587 | 0.029603 |
| 8897 | MTMR3 | myotubularin related protein 3 | -0.35095 | -3.59215 | 0.00059 | 0.029676 |
| 54919 | DNAAF5 | dynein axonemal assembly factor 5 | 0.215525 | 3.587411 | 0.000599 | 0.030071 |
| 7417 | VDAC2 | voltage dependent anion channel 2 | 0.404946 | 3.586337 | 0.000601 | 0.030109 |
|  |  |  | -0.23611 | -3.58051 | 0.000613 | 0.030439 |
| 64795 | RMND5A | required for meiotic nuclear division 5 homolog A | -0.41404 | -3.58121 | 0.000611 | 0.030439 |
|  |  |  | 0.432639 | 3.579605 | 0.000615 | 0.030439 |
|  |  |  | 0.436114 | 3.58027 | 0.000613 | 0.030439 |
| 79735 | TBC1D17 | TBC1 domain family member 17 | -0.25747 | -3.57988 | 0.000614 | 0.030439 |
| 8110 | DPF3 | double PHD fingers 3 | -0.31978 | -3.57748 | 0.000619 | 0.030583 |
| 3046 | HBE1 | hemoglobin subunit epsilon 1 | -0.64106 | -3.57435 | 0.000625 | 0.030828 |
|  |  |  | -0.31474 | -3.57338 | 0.000627 | 0.030858 |
| 23367 | LARP1 | La ribonucleoprotein domain family member 1 | -0.372 | -3.57159 | 0.000631 | 0.030971 |
| 2778 | GNAS | GNAS complex locus | -0.27485 | -3.56413 | 0.000646 | 0.031661 |
| 26512 | INTS6 | integrator complex subunit 6 | -0.29583 | -3.56115 | 0.000652 | 0.0319 |
| 1380 | CR2 | complement C3d receptor 2 | 0.425307 | 3.5565 | 0.000662 | 0.032211 |
| 7634 | ZNF80 | zinc finger protein 80 | 0.266293 | 3.556312 | 0.000663 | 0.032211 |
| 55520 | ELAC1 | elaC ribonuclease Z 1 | 0.248627 | 3.556164 | 0.000663 | 0.032211 |
| 5166 | PDK4 | pyruvate dehydrogenase kinase 4 | 0.364362 | 3.554352 | 0.000667 | 0.032331 |
| 647087 | STMP1 | short transmembrane mitochondrial protein 1 | 0.301302 | 3.552414 | 0.000671 | 0.032465 |
|  |  |  | 0.309288 | 3.546487 | 0.000684 | 0.032468 |
|  |  |  | 0.309288 | 3.546487 | 0.000684 | 0.032468 |
|  |  |  | 0.309288 | 3.546487 | 0.000684 | 0.032468 |
|  |  |  | 0.309288 | 3.546487 | 0.000684 | 0.032468 |
|  |  |  | 0.309288 | 3.546487 | 0.000684 | 0.032468 |
| 375387 | NRROS | negative regulator of reactive oxygen species | 0.255702 | 3.546785 | 0.000684 | 0.032468 |
|  |  |  | -0.2743 | -3.54701 | 0.000683 | 0.032468 |
| 988 | CDC5L | cell division cycle 5 like | 0.242178 | 3.546649 | 0.000684 | 0.032468 |
| 2729 | GCLC | glutamate-cysteine ligase catalytic subunit | -0.40217 | -3.54924 | 0.000678 | 0.032468 |
| 6790 | AURKA | aurora kinase A | -0.30962 | -3.54511 | 0.000687 | 0.032545 |
| 113 | ADCY7 | adenylate cyclase 7 | 0.295587 | 3.543967 | 0.00069 | 0.032597 |
| 221302 | ZUP1 | zinc finger containing ubiquitin peptidase 1 | -0.30924 | -3.54202 | 0.000694 | 0.032734 |
| 80256 | FAM214B | family with sequence similarity 214 member B | -0.34949 | -3.54052 | 0.000698 | 0.032757 |
| 7776 | ZNF236 | zinc finger protein 236 | -0.25937 | -3.54094 | 0.000697 | 0.032757 |
| 6938 | TCF12 | transcription factor 12 | 0.218826 | 3.539516 | 0.0007 | 0.032795 |
| 143689 | PIWIL4 | piwi like RNA-mediated gene silencing 4 | 0.35238 | 3.538272 | 0.000703 | 0.032859 |
|  |  |  | -0.27116 | -3.53678 | 0.000706 | 0.032881 |
| 389075 | RESP18 | regulated endocrine specific protein 18 | 0.26112 | 3.53697 | 0.000706 | 0.032881 |
| 2316 | FLNA | filamin A | 0.265173 | 3.532452 | 0.000716 | 0.033276 |
| 115201 | ATG4A | autophagy related 4A cysteine peptidase | -0.50257 | -3.53133 | 0.000719 | 0.033328 |
| 8761 | PABPC4 | poly(A) binding protein cytoplasmic 4 | 0.21409 | 3.529932 | 0.000722 | 0.03341 |
| 5341 | PLEK | pleckstrin | 0.356584 | 3.526848 | 0.000729 | 0.033675 |
| 374969 | SVBP | small vasohibin binding protein | -0.4571 | -3.51922 | 0.000747 | 0.034442 |
| 55827 | DCAF6 | DDB1 and CUL4 associated factor 6 | -0.37976 | -3.51621 | 0.000754 | 0.034637 |
| 1668 | DEFA3 | defensin alpha 3 | 1.345157 | 3.516235 | 0.000754 | 0.034637 |
| 7009 | TMBIM6 | transmembrane BAX inhibitor motif containing 6 | -0.22685 | -3.51332 | 0.000762 | 0.034889 |
| 83849 | SYT15 | synaptotagmin 15 | 0.253479 | 3.510247 | 0.000769 | 0.035165 |
| 2995 | GYPC | glycophorin C (Gerbich blood group) | -0.27073 | -3.50912 | 0.000772 | 0.035222 |
| 8036 | SHOC2 | SHOC2 leucine rich repeat scaffold protein | -0.20542 | -3.5062 | 0.000779 | 0.035482 |
| 1808 | DPYSL2 | dihydropyrimidinase like 2 | 0.308738 | 3.505347 | 0.000781 | 0.035508 |
| 91543 | RSAD2 | radical S-adenosyl methionine domain containing 2 | -0.62283 | -3.50307 | 0.000787 | 0.035696 |
| 84101 | USP44 | ubiquitin specific peptidase 44 | 0.26096 | 3.501184 | 0.000792 | 0.035842 |
|  |  |  | -0.56435 | -3.49777 | 0.000801 | 0.036163 |
| 8328 | GFI1B | growth factor independent 1B transcriptional repressor | -0.35599 | -3.49485 | 0.000808 | 0.036369 |
| 51079 | NDUFA13 | NADH:ubiquinone oxidoreductase subunit A13 | -0.3679 | -3.49477 | 0.000808 | 0.036369 |
| 84519 | ACRBP | acrosin binding protein | 0.233901 | 3.493668 | 0.000811 | 0.036425 |
| 5586 | PKN2 | protein kinase N2 | -0.24311 | -3.49008 | 0.000821 | 0.036773 |
| 5775 | PTPN4 | protein tyrosine phosphatase non-receptor type 4 | -0.24629 | -3.48763 | 0.000827 | 0.036989 |
| 767 | CA8 | carbonic anhydrase 8 | -0.52036 | -3.48619 | 0.000831 | 0.037087 |
| 6141 | RPL18 | ribosomal protein L18 | 0.199802 | 3.485333 | 0.000833 | 0.037115 |
| 84236 | RHBDD1 | rhomboid domain containing 1 | -0.35983 | -3.48445 | 0.000835 | 0.037126 |
| 226 | ALDOA | aldolase, fructose-bisphosphate A | 0.186641 | 3.484016 | 0.000837 | 0.037126 |
| 84869 | CBR4 | carbonyl reductase 4 | -0.3746 | -3.48238 | 0.000841 | 0.037203 |
| 51092 | SIDT2 | SID1 transmembrane family member 2 | 0.303141 | 3.482143 | 0.000842 | 0.037203 |
| 8916 | HERC3 | HECT and RLD domain containing E3 ubiquitin protein ligase 3 | -0.21935 | -3.48023 | 0.000847 | 0.037286 |
| 10263 | CDK2AP2 | cyclin dependent kinase 2 associated protein 2 | 0.219331 | 3.480288 | 0.000847 | 0.037286 |
| 4815 | NINJ2 | ninjurin 2 | -0.59608 | -3.47268 | 0.000868 | 0.038048 |
| 84669 | USP32 | ubiquitin specific peptidase 32 | -0.24394 | -3.47313 | 0.000866 | 0.038048 |
| 57691 | KIAA1586 | KIAA1586 | -0.31488 | -3.47196 | 0.00087 | 0.038062 |
| 114796 | PSMG3-AS1 | PSMG3 antisense RNA 1 (head to head) | 0.254551 | 3.468026 | 0.000881 | 0.038352 |
| 387680 | WASHC2A | WASH complex subunit 2A | 0.336995 | 3.467914 | 0.000881 | 0.038352 |
| 7296 | TXNRD1 | thioredoxin reductase 1 | -0.30121 | -3.46777 | 0.000881 | 0.038352 |
| 55831 | EMC3 | ER membrane protein complex subunit 3 | -0.34305 | -3.46637 | 0.000885 | 0.038393 |
| 51635 | DHRS7 | dehydrogenase/reductase 7 | 0.342903 | 3.466231 | 0.000886 | 0.038393 |
| 345757 | FAM174A | family with sequence similarity 174 member A | -0.24339 | -3.46472 | 0.00089 | 0.038431 |
| 6175 | RPLP0 | ribosomal protein lateral stalk subunit P0 | 0.250351 | 3.464909 | 0.000889 | 0.038431 |
|  |  |  | -0.24408 | -3.46119 | 0.0009 | 0.038792 |
| 6809 | STX3 | syntaxin 3 | -0.33069 | -3.45826 | 0.000908 | 0.039081 |
| 6612 | SUMO3 | small ubiquitin like modifier 3 | 0.300302 | 3.455787 | 0.000916 | 0.039316 |
| 1024 | CDK8 | cyclin dependent kinase 8 | -0.45271 | -3.45437 | 0.00092 | 0.039419 |
| 3640 | INSL3 | insulin like 3 | -0.21446 | -3.45271 | 0.000925 | 0.039553 |
| 3006 | HIST1H1C | histone cluster 1 H1 family member c | 0.521964 | 3.451739 | 0.000927 | 0.0396 |
| 127933 | UHMK1 | U2AF homology motif kinase 1 | -0.30904 | -3.45094 | 0.00093 | 0.039627 |
| 3587 | IL10RA | interleukin 10 receptor subunit alpha | 0.395834 | 3.449651 | 0.000934 | 0.039714 |
| 2029 | ENSA | endosulfine alpha | -0.25551 | -3.44621 | 0.000944 | 0.040076 |
| 54765 | TRIM44 | tripartite motif containing 44 | 0.248711 | 3.441826 | 0.000957 | 0.040486 |
| 162966 | ZNF600 | zinc finger protein 600 | 0.205865 | 3.441941 | 0.000957 | 0.040486 |
| 100462981 | MTRNR2L2 | MT-RNR2 like 2 | 0.217225 | 3.435652 | 0.000976 | 0.041211 |
| 4709 | NDUFB3 | NADH:ubiquinone oxidoreductase subunit B3 | -0.38226 | -3.43059 | 0.000992 | 0.041722 |
| 2054 | STX2 | syntaxin 2 | 0.33599 | 3.431124 | 0.00099 | 0.041722 |
| 10098 | TSPAN5 | tetraspanin 5 | -0.39991 | -3.42928 | 0.000996 | 0.041818 |
| 6882 | TAF11 | TATA-box binding protein associated factor 11 | -0.23863 | -3.42727 | 0.001002 | 0.042008 |
| 116211 | TM4SF19 | transmembrane 4 L six family member 19 | -0.21961 | -3.42664 | 0.001004 | 0.042014 |
| 333 | APLP1 | amyloid beta precursor like protein 1 | 0.157762 | 3.423479 | 0.001015 | 0.042358 |
| 51335 | NGRN | neugrin, neurite outgrowth associated | -0.28399 | -3.42274 | 0.001017 | 0.04238 |
| 4209 | MEF2D | myocyte enhancer factor 2D | 0.257066 | 3.422026 | 0.001019 | 0.042397 |
| 57198 | ATP8B2 | ATPase phospholipid transporting 8B2 | 0.252735 | 3.420637 | 0.001024 | 0.042428 |
| 192286 | HIGD2A | HIG1 hypoxia inducible domain family member 2A | 0.270212 | 3.420658 | 0.001024 | 0.042428 |
| 54165 | DCUN1D1 | defective in cullin neddylation 1 domain containing 1 | -0.34237 | -3.4158 | 0.00104 | 0.042846 |
|  |  |  | 0.366077 | 3.415967 | 0.001039 | 0.042846 |
| 4946 | OAZ1 | ornithine decarboxylase antizyme 1 | -0.26903 | -3.41682 | 0.001036 | 0.042846 |
| 10974 | ADIRF | adipogenesis regulatory factor | 0.289988 | 3.41231 | 0.001051 | 0.043243 |
| 22821 | RASA3 | RAS p21 protein activator 3 | 0.208223 | 3.407346 | 0.001068 | 0.043847 |
| 7913 | DEK | DEK proto-oncogene | 0.219723 | 3.404433 | 0.001078 | 0.04402 |
| 58475 | MS4A7 | membrane spanning 4-domains A7 | 0.568552 | 3.404373 | 0.001078 | 0.04402 |
| 140735 | DYNLL2 | dynein light chain LC8-type 2 | 0.282734 | 3.404677 | 0.001077 | 0.04402 |
|  |  |  | -0.41451 | -3.40248 | 0.001084 | 0.044204 |
|  |  |  | 0.250996 | 3.399629 | 0.001094 | 0.044524 |
| 5501 | PPP1CC | protein phosphatase 1 catalytic subunit gamma | -0.35183 | -3.39713 | 0.001103 | 0.044795 |
| 571 | BACH1 | BTB domain and CNC homolog 1 | -0.28452 | -3.39654 | 0.001105 | 0.044798 |
| 55144 | LRRC8D | leucine rich repeat containing 8 VRAC subunit D | 0.489746 | 3.392571 | 0.001119 | 0.045281 |
| 3313 | HSPA9 | heat shock protein family A (Hsp70) member 9 | 0.406049 | 3.391947 | 0.001121 | 0.045288 |
| 27069 | GHITM | growth hormone inducible transmembrane protein | -0.42144 | -3.39139 | 0.001123 | 0.045288 |
| 131669 | UROC1 | urocanate hydratase 1 | -0.20635 | -3.38896 | 0.001131 | 0.045545 |
| 2074 | ERCC6 | ERCC excision repair 6, chromatin remodeling factor | -0.25965 | -3.38845 | 0.001133 | 0.045545 |
| 84071 | ARMC2 | armadillo repeat containing 2 | 0.297125 | 3.386144 | 0.001142 | 0.045796 |
| 29889 | GNL2 | G protein nucleolar 2 | 0.300031 | 3.385162 | 0.001145 | 0.045856 |
| 60681 | FKBP10 | FKBP prolyl isomerase 10 | 0.187746 | 3.383638 | 0.001151 | 0.045995 |
| 6722 | SRF | serum response factor | 0.264357 | 3.382591 | 0.001154 | 0.046 |
| 6878 | TAF6 | TATA-box binding protein associated factor 6 | 0.322384 | 3.38248 | 0.001155 | 0.046 |
| 7052 | TGM2 | transglutaminase 2 | -0.3517 | -3.38134 | 0.001159 | 0.046084 |
| 129787 | TMEM18 | transmembrane protein 18 | 0.203597 | 3.379884 | 0.001164 | 0.046214 |
| 7073 | TIAL1 | TIA1 cytotoxic granule associated RNA binding protein like 1 | -0.22493 | -3.3789 | 0.001168 | 0.046276 |
| 57621 | ZBTB2 | zinc finger and BTB domain containing 2 | 0.304541 | 3.377565 | 0.001173 | 0.046389 |
| 359948 | IRF2BP2 | interferon regulatory factor 2 binding protein 2 | 0.35605 | 3.372805 | 0.00119 | 0.047006 |
| 384 | ARG2 | arginase 2 | 0.880194 | 3.36339 | 0.001226 | 0.048331 |
| 11041 | B4GAT1 | beta-1,4-glucuronyltransferase 1 | -0.24201 | -3.36166 | 0.001233 | 0.048509 |
| 8559 | PRPF18 | pre-mRNA processing factor 18 | -0.38436 | -3.3551 | 0.001258 | 0.049429 |
| 9612 | NCOR2 | nuclear receptor corepressor 2 | 0.184542 | 3.353989 | 0.001263 | 0.049515 |
| 5434 | POLR2E | RNA polymerase II subunit E | 0.272655 | 3.352712 | 0.001268 | 0.049628 |
| 747 | DAGLA | diacylglycerol lipase alpha | 0.228526 | 3.351383 | 0.001273 | 0.049748 |
| 140461 | ASB8 | ankyrin repeat and SOCS box containing 8 | -0.27116 | -3.34989 | 0.001279 | 0.049894 |

**Supplemental Table S4. Differentially Expressed Genes in Control Group between Day 1 and 14**

| **ENTREZID** | **SYMBOL** | **GENENAME** | **Log fold change** | **t-stat** | **p-value** | **adj p-value** |
| --- | --- | --- | --- | --- | --- | --- |
| 55204 | GOLPH3L | golgi phosphoprotein 3 like | -1.16255 | -8.86298 | 3.22E-13 | 7.26E-09 |
| 57515 | SERINC1 | serine incorporator 1 | -1.13057 | -8.61028 | 9.62E-13 | 1.09E-08 |
| 26268 | FBXO9 | F-box protein 9 | -1.05086 | -8.20833 | 5.51E-12 | 4.15E-08 |
| 84932 | RAB2B | RAB2B, member RAS oncogene family | -0.86652 | -7.64718 | 6.27E-11 | 1.77E-07 |
| 130540 | FLACC1 | flagellum associated containing coiled-coil domains 1 | -0.73666 | -7.67065 | 5.66E-11 | 1.77E-07 |
| 10085 | EDIL3 | EGF like repeats and discoidin domains 3 | -1.05417 | -7.65602 | 6.03E-11 | 1.77E-07 |
| 51706 | CYB5R1 | cytochrome b5 reductase 1 | -0.88804 | -7.74014 | 4.19E-11 | 1.77E-07 |
| 84337 | ELOF1 | elongation factor 1 homolog | -0.99683 | -7.72262 | 4.52E-11 | 1.77E-07 |
| 10910 | SUGT1 | SGT1 homolog, MIS12 kinetochore complex assembly cochaperone | -0.83296 | -7.44593 | 1.5E-10 | 3.75E-07 |
| 51021 | MRPS16 | mitochondrial ribosomal protein S16 | -0.6198 | -7.35317 | 2.23E-10 | 5.04E-07 |
| 117177 | RAB3IP | RAB3A interacting protein | -0.82356 | -7.30782 | 2.71E-10 | 5.57E-07 |
| 55553 | SOX6 | SRY-box 6 | -0.88569 | -7.24462 | 3.56E-10 | 6.21E-07 |
| 5052 | PRDX1 | peroxiredoxin 1 | -0.86817 | -7.24359 | 3.58E-10 | 6.21E-07 |
| 153527 | ZMAT2 | zinc finger matrin-type 2 | -0.83447 | -7.16182 | 5.08E-10 | 8.2E-07 |
| 8945 | BTRC | beta-transducin repeat containing E3 ubiquitin protein ligase | -0.76236 | -6.96397 | 1.19E-09 | 1.79E-06 |
| 6451 | SH3BGRL | SH3 domain binding glutamate rich protein like | -0.63101 | -6.86684 | 1.8E-09 | 2.25E-06 |
| 9604 | RNF14 | ring finger protein 14 | -0.90843 | -6.88101 | 1.69E-09 | 2.25E-06 |
| 4893 | NRAS | NRAS proto-oncogene, GTPase | -0.62876 | -6.8796 | 1.7E-09 | 2.25E-06 |
| 1510 | CTSE | cathepsin E | -1.64533 | -6.81227 | 2.27E-09 | 2.69E-06 |
| 1870 | E2F2 | E2F transcription factor 2 | -0.96165 | -6.74289 | 3.04E-09 | 3.44E-06 |
| 5868 | RAB5A | RAB5A, member RAS oncogene family | -0.87031 | -6.73008 | 3.21E-09 | 3.46E-06 |
| 60313 | GPBP1L1 | GC-rich promoter binding protein 1 like 1 | -0.56586 | -6.69083 | 3.8E-09 | 3.86E-06 |
| 79018 | GID4 | GID complex subunit 4 homolog | -0.88937 | -6.68269 | 3.93E-09 | 3.86E-06 |
| 9532 | BAG2 | BCL2 associated athanogene 2 | -0.67472 | -6.62717 | 4.97E-09 | 4.68E-06 |
| 3792 | KEL | Kell metallo-endopeptidase (Kell blood group) | -0.71479 | -6.59414 | 5.72E-09 | 5.17E-06 |
|  |  |  | -0.73921 | -6.54977 | 6.9E-09 | 5.63E-06 |
| 26127 | FGFR1OP2 | FGFR1 oncogene partner 2 | -0.72875 | -6.5642 | 6.49E-09 | 5.63E-06 |
| 11235 | PDCD10 | programmed cell death 10 | -0.59203 | -6.54697 | 6.98E-09 | 5.63E-06 |
| 7504 | XK | X-linked Kx blood group | -0.92135 | -6.53383 | 7.38E-09 | 5.71E-06 |
| 92521 | SPECC1 | sperm antigen with calponin homology and coiled-coil domains 1 | -0.7561 | -6.52748 | 7.58E-09 | 5.71E-06 |
| 51257 | MARCH2 | membrane associated ring-CH-type finger 2 | -0.74471 | -6.46657 | 9.8E-09 | 6.77E-06 |
| 219899 | TBCEL | tubulin folding cofactor E like | -0.6046 | -6.46436 | 9.89E-09 | 6.77E-06 |
| 221477 | C6orf89 | chromosome 6 open reading frame 89 | -0.40853 | -6.47766 | 9.35E-09 | 6.77E-06 |
| 317671 | RFESD | Rieske Fe-S domain containing | -1.01902 | -6.42518 | 1.17E-08 | 7.75E-06 |
| 10826 | FAXDC2 | fatty acid hydroxylase domain containing 2 | -0.78617 | -6.39854 | 1.3E-08 | 8.19E-06 |
| 29058 | TMEM230 | transmembrane protein 230 | -0.59612 | -6.40031 | 1.29E-08 | 8.19E-06 |
| 129607 | CMPK2 | cytidine/uridine monophosphate kinase 2 | -0.76106 | -6.36474 | 1.5E-08 | 8.91E-06 |
| 54823 | SWT1 | SWT1 RNA endoribonuclease homolog | -0.70959 | -6.36064 | 1.53E-08 | 8.91E-06 |
| 9588 | PRDX6 | peroxiredoxin 6 | -0.65095 | -6.35929 | 1.54E-08 | 8.91E-06 |
| 210 | ALAD | aminolevulinate dehydratase | -0.63856 | -6.18641 | 3.17E-08 | 1.79E-05 |
| 284904 | SEC14L4 | SEC14 like lipid binding 4 | -0.79535 | -6.15451 | 3.62E-08 | 1.99E-05 |
| 23613 | ZMYND8 | zinc finger MYND-type containing 8 | -0.49839 | -6.09449 | 4.64E-08 | 2.5E-05 |
|  |  |  | -0.52195 | -6.06225 | 5.31E-08 | 2.79E-05 |
| 63893 | UBE2O | ubiquitin conjugating enzyme E2 O | -1.07049 | -6.04836 | 5.62E-08 | 2.89E-05 |
| 4303 | FOXO4 | forkhead box O4 | -0.76972 | -6.03998 | 5.82E-08 | 2.92E-05 |
| 10284 | SAP18 | Sin3A associated protein 18 | -0.60792 | -6.00593 | 6.7E-08 | 3.29E-05 |
| 84993 | UBL7 | ubiquitin like 7 | -0.47698 | -5.99602 | 6.98E-08 | 3.35E-05 |
| 5609 | MAP2K7 | mitogen-activated protein kinase kinase 7 | -0.65787 | -5.98093 | 7.43E-08 | 3.36E-05 |
| 54926 | UBE2R2 | ubiquitin conjugating enzyme E2 R2 | 0.493784 | 5.98033 | 7.45E-08 | 3.36E-05 |
| 51646 | YPEL5 | yippee like 5 | -0.56366 | -5.98088 | 7.43E-08 | 3.36E-05 |
| 220972 | MARCH8 | membrane associated ring-CH-type finger 8 | -0.9248 | -5.9717 | 7.72E-08 | 3.42E-05 |
| 7029 | TFDP2 | transcription factor Dp-2 | -0.68479 | -5.95942 | 8.12E-08 | 3.47E-05 |
| 653659 | TMEM183B | transmembrane protein 183B | -0.48067 | -5.95841 | 8.15E-08 | 3.47E-05 |
| 7037 | TFRC | transferrin receptor | -0.78187 | -5.90567 | 1.01E-07 | 4.24E-05 |
| 1069 | CETN2 | centrin 2 | -0.7237 | -5.88528 | 1.1E-07 | 4.44E-05 |
| 4676 | NAP1L4 | nucleosome assembly protein 1 like 4 | -0.48027 | -5.8872 | 1.09E-07 | 4.44E-05 |
| 27109 | DMAC2L | distal membrane arm assembly complex 2 like | -0.74361 | -5.87558 | 1.15E-07 | 4.54E-05 |
| 128989 | TANGO2 | transport and golgi organization 2 homolog | -0.5717 | -5.86526 | 1.2E-07 | 4.66E-05 |
| 1642 | DDB1 | damage specific DNA binding protein 1 | -0.89148 | -5.8043 | 1.53E-07 | 5.6E-05 |
| 25911 | DPCD | deleted in primary ciliary dyskinesia homolog (mouse) | -0.79353 | -5.80367 | 1.54E-07 | 5.6E-05 |
| 9320 | TRIP12 | thyroid hormone receptor interactor 12 | -0.3845 | -5.80746 | 1.51E-07 | 5.6E-05 |
| 359948 | IRF2BP2 | interferon regulatory factor 2 binding protein 2 | 0.651569 | 5.808806 | 1.51E-07 | 5.6E-05 |
| 4121 | MAN1A1 | mannosidase alpha class 1A member 1 | -0.62395 | -5.77276 | 1.75E-07 | 6.16E-05 |
| 6923 | ELOB | elongin B | -0.47305 | -5.77402 | 1.74E-07 | 6.16E-05 |
| 7111 | TMOD1 | tropomodulin 1 | -0.68011 | -5.7682 | 1.78E-07 | 6.18E-05 |
| 6780 | STAU1 | staufen double-stranded RNA binding protein 1 | -0.45264 | -5.73128 | 2.07E-07 | 7.07E-05 |
| 4154 | MBNL1 | muscleblind like splicing regulator 1 | 0.377244 | 5.724466 | 2.13E-07 | 7.16E-05 |
|  |  |  | -0.95107 | -5.70576 | 2.29E-07 | 7.62E-05 |
| 23762 | OSBP2 | oxysterol binding protein 2 | -0.7216 | -5.66243 | 2.73E-07 | 8.24E-05 |
| 93621 | MRFAP1 | Morf4 family associated protein 1 | -0.74107 | -5.66812 | 2.67E-07 | 8.24E-05 |
|  |  |  | -0.74081 | -5.65889 | 2.77E-07 | 8.24E-05 |
|  |  |  | -0.74081 | -5.65889 | 2.77E-07 | 8.24E-05 |
|  |  |  | -0.74081 | -5.65889 | 2.77E-07 | 8.24E-05 |
|  |  |  | -0.74081 | -5.65889 | 2.77E-07 | 8.24E-05 |
|  |  |  | -0.74081 | -5.65889 | 2.77E-07 | 8.24E-05 |
|  |  |  | -0.74081 | -5.65889 | 2.77E-07 | 8.24E-05 |
| 9070 | ASH2L | ASH2 like, histone lysine methyltransferase complex subunit | -0.4608 | -5.6389 | 3.01E-07 | 8.74E-05 |
| 23204 | ARL6IP1 | ADP ribosylation factor like GTPase 6 interacting protein 1 | -0.67196 | -5.63818 | 3.02E-07 | 8.74E-05 |
| 4750 | NEK1 | NIMA related kinase 1 | -0.42315 | -5.63423 | 3.07E-07 | 8.76E-05 |
| 26036 | ZNF451 | zinc finger protein 451 | -0.49938 | -5.62412 | 3.19E-07 | 9.02E-05 |
|  |  |  | -0.83954 | -5.59996 | 3.52E-07 | 9.82E-05 |
|  |  |  | -0.91622 | -5.58797 | 3.7E-07 | 0.000102 |
| 5532 | PPP3CB | protein phosphatase 3 catalytic subunit beta | -0.53552 | -5.5739 | 3.91E-07 | 0.000103 |
| 55363 | HEMGN | hemogen | -0.4616 | -5.56919 | 3.99E-07 | 0.000103 |
| 2996 | GYPE | glycophorin E (MNS blood group) | -0.67744 | -5.57835 | 3.84E-07 | 0.000103 |
| 6048 | RNF5 | ring finger protein 5 | -0.71551 | -5.56802 | 4.01E-07 | 0.000103 |
|  |  |  | -1.02802 | -5.57132 | 3.95E-07 | 0.000103 |
| 149840 | SHLD1 | shieldin complex subunit 1 | -0.82282 | -5.57243 | 3.93E-07 | 0.000103 |
| 7381 | UQCRB | ubiquinol-cytochrome c reductase binding protein | -0.55645 | -5.56288 | 4.09E-07 | 0.000104 |
| 689 | BTF3 | basic transcription factor 3 | -0.41866 | -5.55034 | 4.3E-07 | 0.000108 |
| 966 | CD59 | CD59 molecule (CD59 blood group) | -0.77136 | -5.51191 | 5.02E-07 | 0.000122 |
| 10928 | RALBP1 | ralA binding protein 1 | -0.37022 | -5.51139 | 5.03E-07 | 0.000122 |
| 1983 | EIF5 | eukaryotic translation initiation factor 5 | -0.59403 | -5.51638 | 4.93E-07 | 0.000122 |
| 92745 | SLC38A5 | solute carrier family 38 member 5 | -0.79905 | -5.50143 | 5.24E-07 | 0.000126 |
| 10107 | TRIM10 | tripartite motif containing 10 | -0.83283 | -5.4794 | 5.72E-07 | 0.000135 |
|  |  |  | -0.79398 | -5.47972 | 5.71E-07 | 0.000135 |
| 55766 | H2AFJ | H2A histone family member J | -0.60622 | -5.47513 | 5.82E-07 | 0.000135 |
|  |  |  | -0.9072 | -5.47116 | 5.91E-07 | 0.000135 |
| 378 | ARF4 | ADP ribosylation factor 4 | -0.59228 | -5.47115 | 5.91E-07 | 0.000135 |
| 252839 | TMEM9 | transmembrane protein 9 | -0.47707 | -5.46756 | 6E-07 | 0.000135 |
| 6647 | SOD1 | superoxide dismutase 1 | -0.69375 | -5.43776 | 6.76E-07 | 0.000151 |
| 90736 | FAM104B | family with sequence similarity 104 member B | -0.42956 | -5.4335 | 6.87E-07 | 0.000152 |
|  |  |  | -0.99518 | -5.41011 | 7.55E-07 | 0.000165 |
|  |  |  | -0.89133 | -5.40246 | 7.78E-07 | 0.000167 |
| 2874 | GPS2 | G protein pathway suppressor 2 | -0.47513 | -5.40336 | 7.75E-07 | 0.000167 |
| 8444 | DYRK3 | dual specificity tyrosine phosphorylation regulated kinase 3 | -0.94218 | -5.3869 | 8.28E-07 | 0.000175 |
| 84299 | MIEN1 | migration and invasion enhancer 1 | -0.53543 | -5.38606 | 8.3E-07 | 0.000175 |
| 5600 | MAPK11 | mitogen-activated protein kinase 11 | 0.336151 | 5.373894 | 8.72E-07 | 0.000182 |
| 79048 | SECISBP2 | SECIS binding protein 2 | -0.41904 | -5.3658 | 9E-07 | 0.000183 |
| 81688 | C6orf62 | chromosome 6 open reading frame 62 | -0.54657 | -5.36595 | 9E-07 | 0.000183 |
| 23274 | CLEC16A | C-type lectin domain containing 16A | -0.58478 | -5.36893 | 8.89E-07 | 0.000183 |
| 23039 | XPO7 | exportin 7 | -0.4936 | -5.34753 | 9.68E-07 | 0.000195 |
| 25996 | REXO2 | RNA exonuclease 2 | -0.76714 | -5.33945 | 1E-06 | 0.0002 |
| 1656 | DDX6 | DEAD-box helicase 6 | -0.48199 | -5.33423 | 1.02E-06 | 0.000202 |
| 23161 | SNX13 | sorting nexin 13 | -0.42426 | -5.30674 | 1.14E-06 | 0.000223 |
| 80127 | BBOF1 | basal body orientation factor 1 | -0.81708 | -5.30313 | 1.15E-06 | 0.000225 |
| 55527 | FEM1A | fem-1 homolog A | -0.50379 | -5.28399 | 1.25E-06 | 0.00024 |
| 25950 | RWDD3 | RWD domain containing 3 | -0.56347 | -5.27045 | 1.31E-06 | 0.000251 |
| 1603 | DAD1 | defender against cell death 1 | -0.45108 | -5.2656 | 1.34E-06 | 0.000252 |
| 598 | BCL2L1 | BCL2 like 1 | -0.56079 | -5.26694 | 1.33E-06 | 0.000252 |
| 3159 | HMGA1 | high mobility group AT-hook 1 | 0.471863 | 5.251794 | 1.41E-06 | 0.000262 |
| 11080 | DNAJB4 | DnaJ heat shock protein family (Hsp40) member B4 | -0.75158 | -5.25225 | 1.41E-06 | 0.000262 |
| 94103 | ORMDL3 | ORMDL sphingolipid biosynthesis regulator 3 | -0.71096 | -5.24136 | 1.47E-06 | 0.000268 |
| 1349 | COX7B | cytochrome c oxidase subunit 7B | -0.66897 | -5.24257 | 1.47E-06 | 0.000268 |
| 81689 | ISCA1 | iron-sulfur cluster assembly 1 | -0.66119 | -5.22899 | 1.55E-06 | 0.00028 |
| 6248 | RSC1A1 | regulator of solute carriers 1 | -0.44977 | -5.21555 | 1.63E-06 | 0.00029 |
| 26224 | FBXL3 | F-box and leucine rich repeat protein 3 | -0.67931 | -5.21583 | 1.63E-06 | 0.00029 |
| 116228 | COX20 | cytochrome c oxidase assembly factor COX20 | -0.57206 | -5.21021 | 1.67E-06 | 0.000294 |
| 81627 | TRMT1L | tRNA methyltransferase 1 like | -0.59417 | -5.19498 | 1.77E-06 | 0.00031 |
| 84232 | MAF1 | MAF1 homolog, negative regulator of RNA polymerase III | -0.39141 | -5.1768 | 1.9E-06 | 0.00033 |
| 4189 | DNAJB9 | DnaJ heat shock protein family (Hsp40) member B9 | -0.7688 | -5.14651 | 2.14E-06 | 0.000369 |
| 9240 | PNMA1 | PNMA family member 1 | 0.794572 | 5.129795 | 2.28E-06 | 0.000391 |
| 222642 | TSPO2 | translocator protein 2 | -1.15082 | -5.10833 | 2.48E-06 | 0.000414 |
| 23633 | KPNA6 | karyopherin subunit alpha 6 | -0.42289 | -5.1113 | 2.45E-06 | 0.000414 |
| 9829 | DNAJC6 | DnaJ heat shock protein family (Hsp40) member C6 | -0.49336 | -5.10704 | 2.49E-06 | 0.000414 |
| 5498 | PPOX | protoporphyrinogen oxidase | -0.53389 | -5.10783 | 2.49E-06 | 0.000414 |
| 80323 | CCDC68 | coiled-coil domain containing 68 | -0.74796 | -5.1016 | 2.55E-06 | 0.00042 |
| 100141515 | C17orf99 | chromosome 17 open reading frame 99 | -0.62724 | -5.09022 | 2.66E-06 | 0.000436 |
| 9663 | LPIN2 | lipin 2 | -0.56353 | -5.07308 | 2.85E-06 | 0.000463 |
| 23240 | TMEM131L | transmembrane 131 like | -0.43551 | -5.07111 | 2.87E-06 | 0.000463 |
| 253012 | HEPACAM2 | HEPACAM family member 2 | -1.3645 | -5.05932 | 3E-06 | 0.000481 |
| 53349 | ZFYVE1 | zinc finger FYVE-type containing 1 | -0.41816 | -5.04848 | 3.13E-06 | 0.000498 |
| 7389 | UROD | uroporphyrinogen decarboxylase | -0.62489 | -5.04407 | 3.19E-06 | 0.000503 |
| 9419 | CRIPT | CXXC repeat containing interactor of PDZ3 domain | -0.50668 | -5.02741 | 3.4E-06 | 0.000533 |
| 54977 | SLC25A38 | solute carrier family 25 member 38 | -0.82626 | -5.0226 | 3.46E-06 | 0.00054 |
| 2995 | GYPC | glycophorin C (Gerbich blood group) | -0.41041 | -5.00631 | 3.69E-06 | 0.000571 |
| 1173 | AP2M1 | adaptor related protein complex 2 subunit mu 1 | -0.38118 | -5.00333 | 3.73E-06 | 0.000573 |
| 10661 | KLF1 | Kruppel like factor 1 | -0.5146 | -4.99346 | 3.88E-06 | 0.000592 |
| 3093 | UBE2K | ubiquitin conjugating enzyme E2 K | -0.35707 | -4.98013 | 4.08E-06 | 0.000619 |
| 9318 | COPS2 | COP9 signalosome subunit 2 | -0.53473 | -4.97531 | 4.16E-06 | 0.000626 |
| 1317 | SLC31A1 | solute carrier family 31 member 1 | -0.56357 | -4.96656 | 4.3E-06 | 0.000643 |
| 6917 | TCEA1 | transcription elongation factor A1 | 0.648628 | 4.957811 | 4.45E-06 | 0.000661 |
| 80896 | NPL | N-acetylneuraminate pyruvate lyase | -0.59889 | -4.94456 | 4.68E-06 | 0.000691 |
| 11227 | GALNT5 | polypeptide N-acetylgalactosaminyltransferase 5 | -1.01331 | -4.93998 | 4.77E-06 | 0.000699 |
| 51629 | SLC25A39 | solute carrier family 25 member 39 | -0.43394 | -4.93381 | 4.88E-06 | 0.000711 |
| 29068 | ZBTB44 | zinc finger and BTB domain containing 44 | -0.37935 | -4.92259 | 5.09E-06 | 0.000738 |
| 6563 | SLC14A1 | solute carrier family 14 member 1 (Kidd blood group) | -0.96113 | -4.91178 | 5.31E-06 | 0.000764 |
| 8775 | NAPA | NSF attachment protein alpha | -0.56136 | -4.90642 | 5.42E-06 | 0.000775 |
|  |  |  | -0.5794 | -4.90115 | 5.53E-06 | 0.000786 |
| 7430 | EZR | ezrin | 0.602831 | 4.893404 | 5.7E-06 | 0.00079 |
| 51506 | UFC1 | ubiquitin-fold modifier conjugating enzyme 1 | -0.39456 | -4.89664 | 5.63E-06 | 0.00079 |
| 8560 | DEGS1 | delta 4-desaturase, sphingolipid 1 | 0.540308 | 4.893535 | 5.69E-06 | 0.00079 |
| 83931 | STK40 | serine/threonine kinase 40 | 0.440726 | 4.896381 | 5.63E-06 | 0.00079 |
| 3934 | LCN2 | lipocalin 2 | 1.199696 | 4.8673 | 6.3E-06 | 0.000862 |
| 286451 | YIPF6 | Yip1 domain family member 6 | -0.40732 | -4.8688 | 6.26E-06 | 0.000862 |
| 6158 | RPL28 | ribosomal protein L28 | 0.461037 | 4.863187 | 6.4E-06 | 0.00087 |
| 10159 | ATP6AP2 | ATPase H+ transporting accessory protein 2 | -0.43645 | -4.86023 | 6.47E-06 | 0.000875 |
| 2752 | GLUL | glutamate-ammonia ligase | -0.41817 | -4.84845 | 6.77E-06 | 0.000904 |
| 637 | BID | BH3 interacting domain death agonist | 0.524331 | 4.84997 | 6.73E-06 | 0.000904 |
| 80344 | DCAF11 | DDB1 and CUL4 associated factor 11 | -0.46866 | -4.83882 | 7.02E-06 | 0.000933 |
| 55907 | CMAS | cytidine monophosphate N-acetylneuraminic acid synthetase | -0.94928 | -4.82659 | 7.35E-06 | 0.000971 |
| 127933 | UHMK1 | U2AF homology motif kinase 1 | -0.45895 | -4.82313 | 7.45E-06 | 0.000978 |
|  |  |  | 0.476996 | 4.812729 | 7.75E-06 | 0.001005 |
| 132660 | LIN54 | lin-54 DREAM MuvB core complex component | -0.48761 | -4.81211 | 7.77E-06 | 0.001005 |
| 23608 | MKRN1 | makorin ring finger protein 1 | -0.65576 | -4.80866 | 7.87E-06 | 0.001005 |
| 80256 | FAM214B | family with sequence similarity 214 member B | -0.50424 | -4.80743 | 7.91E-06 | 0.001005 |
| 79366 | HMGN5 | high mobility group nucleosome binding domain 5 | -0.41826 | -4.80997 | 7.83E-06 | 0.001005 |
| 2639 | GCDH | glutaryl-CoA dehydrogenase | -0.32274 | -4.80712 | 7.92E-06 | 0.001005 |
| 221302 | ZUP1 | zinc finger containing ubiquitin peptidase 1 | -0.44512 | -4.79821 | 8.19E-06 | 0.001034 |
| 8724 | SNX3 | sorting nexin 3 | -0.40883 | -4.79466 | 8.3E-06 | 0.001042 |
| 23418 | CRB1 | crumbs cell polarity complex component 1 | -0.48387 | -4.78741 | 8.53E-06 | 0.001065 |
| 84967 | LSM10 | LSM10, U7 small nuclear RNA associated | 0.434112 | 4.782141 | 8.71E-06 | 0.001077 |
| 9612 | NCOR2 | nuclear receptor corepressor 2 | 0.279548 | 4.781543 | 8.73E-06 | 0.001077 |
| 85021 | REPS1 | RALBP1 associated Eps domain containing 1 | 0.330729 | 4.779012 | 8.81E-06 | 0.001081 |
| 192666 | KRT24 | keratin 24 | -0.39278 | -4.77635 | 8.9E-06 | 0.001087 |
| 54855 | TENT5C | terminal nucleotidyltransferase 5C | -0.4136 | -4.77276 | 9.02E-06 | 0.001095 |
| 9352 | TXNL1 | thioredoxin like 1 | -0.48695 | -4.76419 | 9.32E-06 | 0.001126 |
| 55633 | TBC1D22B | TBC1 domain family member 22B | -0.5583 | -4.75722 | 9.57E-06 | 0.001149 |
| 283450 | HECTD4 | HECT domain E3 ubiquitin protein ligase 4 | -0.63225 | -4.74938 | 9.86E-06 | 0.001168 |
|  |  |  | -0.60698 | -4.74876 | 9.88E-06 | 0.001168 |
| 55827 | DCAF6 | DDB1 and CUL4 associated factor 6 | -0.54526 | -4.75136 | 9.78E-06 | 0.001168 |
| 5792 | PTPRF | protein tyrosine phosphatase receptor type F | -0.4051 | -4.74234 | 1.01E-05 | 0.001191 |
| 1340 | COX6B1 | cytochrome c oxidase subunit 6B1 | -0.33453 | -4.7371 | 1.03E-05 | 0.001208 |
| 10955 | SERINC3 | serine incorporator 3 | -0.41347 | -4.73141 | 1.05E-05 | 0.001222 |
| 645 | BLVRB | biliverdin reductase B | -0.45668 | -4.73137 | 1.05E-05 | 0.001222 |
| 127733 | UBXN10 | UBX domain protein 10 | -0.57314 | -4.72379 | 1.09E-05 | 0.001251 |
| 83658 | DYNLRB1 | dynein light chain roadblock-type 1 | -0.39369 | -4.7157 | 1.12E-05 | 0.001278 |
| 114625 | ERMAP | erythroblast membrane associated protein (Scianna blood group) | -0.65813 | -4.71489 | 1.12E-05 | 0.001278 |
| 204851 | HIPK1 | homeodomain interacting protein kinase 1 | -0.36124 | -4.71399 | 1.13E-05 | 0.001278 |
| 27095 | TRAPPC3 | trafficking protein particle complex 3 | -0.45829 | -4.71067 | 1.14E-05 | 0.001288 |
| 84923 | FAM104A | family with sequence similarity 104 member A | -0.56512 | -4.69981 | 1.19E-05 | 0.00133 |
| 2029 | ENSA | endosulfine alpha | -0.37023 | -4.69951 | 1.19E-05 | 0.00133 |
| 6117 | RPA1 | replication protein A1 | 0.4976 | 4.686943 | 1.25E-05 | 0.001378 |
| 79735 | TBC1D17 | TBC1 domain family member 17 | -0.35811 | -4.6861 | 1.25E-05 | 0.001378 |
| 2316 | FLNA | filamin A | 0.373838 | 4.686795 | 1.25E-05 | 0.001378 |
| 2052 | EPHX1 | epoxide hydrolase 1 | 0.475585 | 4.682328 | 1.27E-05 | 0.001391 |
| 7265 | TTC1 | tetratricopeptide repeat domain 1 | -0.53374 | -4.68078 | 1.28E-05 | 0.001392 |
| 84164 | ASCC2 | activating signal cointegrator 1 complex subunit 2 | -0.53998 | -4.67599 | 1.3E-05 | 0.001411 |
| 28977 | MRPL42 | mitochondrial ribosomal protein L42 | -0.62893 | -4.67194 | 1.32E-05 | 0.001412 |
| 8402 | SLC25A11 | solute carrier family 25 member 11 | 0.293974 | 4.67203 | 1.32E-05 | 0.001412 |
| 84678 | KDM2B | lysine demethylase 2B | 0.374372 | 4.673317 | 1.31E-05 | 0.001412 |
| 8818 | DPM2 | dolichyl-phosphate mannosyltransferase subunit 2, regulatory | -0.5041 | -4.66997 | 1.33E-05 | 0.001416 |
| 57095 | PITHD1 | PITH domain containing 1 | -0.76134 | -4.66693 | 1.34E-05 | 0.001425 |
| 54165 | DCUN1D1 | defective in cullin neddylation 1 domain containing 1 | -0.49539 | -4.65145 | 1.42E-05 | 0.001503 |
| 115123 | MARCH3 | membrane associated ring-CH-type finger 3 | -0.50279 | -4.64521 | 1.46E-05 | 0.001518 |
|  |  |  | -0.64112 | -4.64346 | 1.47E-05 | 0.001518 |
| 10098 | TSPAN5 | tetraspanin 5 | -0.57552 | -4.64456 | 1.46E-05 | 0.001518 |
| 9679 | FAM53B | family with sequence similarity 53 member B | 0.433078 | 4.643628 | 1.47E-05 | 0.001518 |
| 5196 | PF4 | platelet factor 4 | 0.408788 | 4.642732 | 1.47E-05 | 0.001518 |
| 163 | AP2B1 | adaptor related protein complex 2 subunit beta 1 | -0.4934 | -4.63845 | 1.5E-05 | 0.001535 |
| 642757 | TBC1D22A-AS1 | TBC1D22A antisense RNA 1 | 0.343639 | 4.629876 | 1.54E-05 | 0.001576 |
| 56922 | MCCC1 | methylcrotonoyl-CoA carboxylase 1 | 0.35451 | 4.628937 | 1.55E-05 | 0.001576 |
| 51327 | AHSP | alpha hemoglobin stabilizing protein | -0.65701 | -4.60758 | 1.68E-05 | 0.0017 |
| 3998 | LMAN1 | lectin, mannose binding 1 | 0.823331 | 4.602303 | 1.71E-05 | 0.001726 |
| 2495 | FTH1 | ferritin heavy chain 1 | 0.304982 | 4.599031 | 1.73E-05 | 0.001739 |
| 1508 | CTSB | cathepsin B | -0.652 | -4.59495 | 1.76E-05 | 0.001758 |
| 162466 | PHOSPHO1 | phosphoethanolamine/phosphocholine phosphatase | -0.64359 | -4.5824 | 1.84E-05 | 0.001834 |
| 387036 | GUSBP2 | GUSB pseudogene 2 | 0.516172 | 4.580401 | 1.86E-05 | 0.001839 |
| 4860 | PNP | purine nucleoside phosphorylase | -0.65668 | -4.56783 | 1.95E-05 | 0.001919 |
| 6708 | SPTA1 | spectrin alpha, erythrocytic 1 | -1.27464 | -4.56616 | 1.96E-05 | 0.001922 |
| 25780 | RASGRP3 | RAS guanyl releasing protein 3 | -1.18535 | -4.55864 | 2.01E-05 | 0.001968 |
| 26100 | WIPI2 | WD repeat domain, phosphoinositide interacting 2 | -0.38512 | -4.53826 | 2.17E-05 | 0.002113 |
| 57552 | NCEH1 | neutral cholesterol ester hydrolase 1 | -0.67721 | -4.52775 | 2.26E-05 | 0.002187 |
| 1984 | EIF5A | eukaryotic translation initiation factor 5A | -0.49262 | -4.52196 | 2.31E-05 | 0.002216 |
| 342618 | SLFN14 | schlafen family member 14 | -0.86836 | -4.52229 | 2.3E-05 | 0.002216 |
| 92703 | TMEM183A | transmembrane protein 183A | -0.42378 | -4.51667 | 2.35E-05 | 0.00225 |
| 7486 | WRN | WRN RecQ like helicase | -0.48121 | -4.511 | 2.4E-05 | 0.002284 |
| 92597 | MOB1B | MOB kinase activator 1B | -0.48128 | -4.51025 | 2.41E-05 | 0.002284 |
| 37 | ACADVL | acyl-CoA dehydrogenase very long chain | 0.450854 | 4.504579 | 2.46E-05 | 0.002323 |
| 66008 | TRAK2 | trafficking kinesin protein 2 | -0.6955 | -4.4992 | 2.51E-05 | 0.002359 |
| 25941 | TPGS2 | tubulin polyglutamylase complex subunit 2 | -0.47902 | -4.4962 | 2.53E-05 | 0.002376 |
| 146722 | CD300LF | CD300 molecule like family member f | 0.320261 | 4.489026 | 2.6E-05 | 0.002429 |
| 22821 | RASA3 | RAS p21 protein activator 3 | 0.291271 | 4.485717 | 2.63E-05 | 0.002446 |
| 22924 | MAPRE3 | microtubule associated protein RP/EB family member 3 | 0.674451 | 4.484887 | 2.64E-05 | 0.002446 |
| 8315 | BRAP | BRCA1 associated protein | -0.42066 | -4.48007 | 2.69E-05 | 0.00248 |
| 115950 | ZNF653 | zinc finger protein 653 | -0.43223 | -4.47406 | 2.75E-05 | 0.002525 |
| 220594 | USP32P2 | ubiquitin specific peptidase 32 pseudogene 2 | -0.33987 | -4.46653 | 2.83E-05 | 0.002585 |
| 5906 | RAP1A | RAP1A, member of RAS oncogene family | -0.46393 | -4.46319 | 2.86E-05 | 0.002599 |
| 2038 | EPB42 | erythrocyte membrane protein band 4.2 | -0.54665 | -4.46285 | 2.87E-05 | 0.002599 |
| 996 | CDC27 | cell division cycle 27 | -0.3782 | -4.46157 | 2.88E-05 | 0.002601 |
| 1727 | CYB5R3 | cytochrome b5 reductase 3 | -0.58041 | -4.45992 | 2.9E-05 | 0.002606 |
| 5303 | PIN4 | peptidylprolyl cis/trans isomerase, NIMA-interacting 4 | -0.51858 | -4.44865 | 3.02E-05 | 0.002696 |
| 51400 | PPME1 | protein phosphatase methylesterase 1 | -0.66209 | -4.4485 | 3.02E-05 | 0.002696 |
| 10121 | ACTR1A | actin related protein 1A | -0.47847 | -4.44643 | 3.04E-05 | 0.002706 |
| 22809 | ATF5 | activating transcription factor 5 | 0.603988 | 4.444718 | 3.06E-05 | 0.002709 |
| 25875 | LETMD1 | LETM1 domain containing 1 | 0.448429 | 4.444 | 3.07E-05 | 0.002709 |
| 10971 | YWHAQ | tyrosine 3-monooxygenase/tryptophan 5-monooxygenase activation protein theta | 0.316399 | 4.440873 | 3.11E-05 | 0.00273 |
| 432369 | ATP5F1EP2 | ATP synthase F1 subunit epsilon pseudogene 2 | -0.34819 | -4.43172 | 3.21E-05 | 0.002812 |
|  |  |  | -0.59952 | -4.43039 | 3.23E-05 | 0.002814 |
| 51335 | NGRN | neugrin, neurite outgrowth associated | -0.39 | -4.42375 | 3.31E-05 | 0.002872 |
| 16 | AARS | alanyl-tRNA synthetase | 0.846562 | 4.415485 | 3.41E-05 | 0.002949 |
|  |  |  | 0.462624 | 4.412273 | 3.45E-05 | 0.00295 |
|  |  |  | 0.462624 | 4.412273 | 3.45E-05 | 0.00295 |
|  |  |  | 0.462624 | 4.412273 | 3.45E-05 | 0.00295 |
| 25874 | MPC2 | mitochondrial pyruvate carrier 2 | -0.49379 | -4.41039 | 3.47E-05 | 0.002959 |
| 6790 | AURKA | aurora kinase A | -0.40799 | -4.39635 | 3.65E-05 | 0.003103 |
| 55108 | BSDC1 | BSD domain containing 1 | -0.39842 | -4.38822 | 3.76E-05 | 0.003184 |
| 118429 | ANTXR2 | ANTXR cell adhesion molecule 2 | 0.365411 | 4.383351 | 3.83E-05 | 0.003229 |
| 54809 | SAMD9 | sterile alpha motif domain containing 9 | -0.94441 | -4.38095 | 3.86E-05 | 0.003245 |
| 52 | ACP1 | acid phosphatase 1 | -0.60839 | -4.37519 | 3.95E-05 | 0.003301 |
| 23142 | DCUN1D4 | defective in cullin neddylation 1 domain containing 4 | -0.39764 | -4.37327 | 3.97E-05 | 0.003304 |
| 373 | TRIM23 | tripartite motif containing 23 | -0.47572 | -4.37296 | 3.98E-05 | 0.003304 |
| 8027 | STAM | signal transducing adaptor molecule | -0.36291 | -4.36923 | 4.03E-05 | 0.00333 |
| 3674 | ITGA2B | integrin subunit alpha 2b | 0.427376 | 4.368789 | 4.04E-05 | 0.00333 |
| 6633 | SNRPD2 | small nuclear ribonucleoprotein D2 polypeptide | 0.322537 | 4.36396 | 4.11E-05 | 0.003376 |
| 6006 | RHCE | Rh blood group CcEe antigens | -0.68038 | -4.34639 | 4.38E-05 | 0.003572 |
| 5292 | PIM1 | Pim-1 proto-oncogene, serine/threonine kinase | -0.57749 | -4.34652 | 4.38E-05 | 0.003572 |
| 10569 | SLU7 | SLU7 homolog, splicing factor | -0.38839 | -4.32099 | 4.8E-05 | 0.003902 |
| 7444 | VRK2 | VRK serine/threonine kinase 2 | -0.41803 | -4.31872 | 4.84E-05 | 0.00392 |
| 9293 | GPR52 | G protein-coupled receptor 52 | -0.44922 | -4.30919 | 5.01E-05 | 0.004042 |
| 226 | ALDOA | aldolase, fructose-bisphosphate A | 0.24522 | 4.308001 | 5.03E-05 | 0.004045 |
| 6767 | ST13 | ST13 Hsp70 interacting protein | -0.46913 | -4.30222 | 5.14E-05 | 0.004115 |
|  |  |  | -0.67199 | -4.29861 | 5.21E-05 | 0.004155 |
| 820 | CAMP | cathelicidin antimicrobial peptide | 1.035967 | 4.290414 | 5.36E-05 | 0.004261 |
| 6947 | TCN1 | transcobalamin 1 | 0.90196 | 4.289526 | 5.38E-05 | 0.004261 |
| 11017 | SNRNP27 | small nuclear ribonucleoprotein U4/U6.U5 subunit 27 | -0.51728 | -4.28769 | 5.41E-05 | 0.004261 |
| 832 | CAPZB | capping actin protein of muscle Z-line subunit beta | 0.304607 | 4.288149 | 5.41E-05 | 0.004261 |
| 91543 | RSAD2 | radical S-adenosyl methionine domain containing 2 | -0.80783 | -4.27607 | 5.65E-05 | 0.004427 |
| 7001 | PRDX2 | peroxiredoxin 2 | -0.56414 | -4.2743 | 5.68E-05 | 0.00444 |
| 1039 | CDR2 | cerebellar degeneration related protein 2 | 0.480225 | 4.270161 | 5.77E-05 | 0.004475 |
| 1612 | DAPK1 | death associated protein kinase 1 | 0.416103 | 4.270265 | 5.76E-05 | 0.004475 |
| 2115 | ETV1 | ETS variant 1 | 0.370712 | 4.26135 | 5.95E-05 | 0.004591 |
| 25840 | METTL7A | methyltransferase like 7A | -0.82473 | -4.26113 | 5.96E-05 | 0.004591 |
| 1116 | CHI3L1 | chitinase 3 like 1 | 0.780679 | 4.26007 | 5.98E-05 | 0.004593 |
| 9911 | TMCC2 | transmembrane and coiled-coil domain family 2 | -0.71916 | -4.25834 | 6.02E-05 | 0.004606 |
| 55144 | LRRC8D | leucine rich repeat containing 8 VRAC subunit D | 0.652409 | 4.253277 | 6.13E-05 | 0.004674 |
| 5884 | RAD17 | RAD17 checkpoint clamp loader component | -0.47565 | -4.2504 | 6.19E-05 | 0.004707 |
| 6130 | RPL7A | ribosomal protein L7a | 0.280093 | 4.248446 | 6.23E-05 | 0.004724 |
| 513 | ATP5F1D | ATP synthase F1 subunit delta | 0.358279 | 4.244509 | 6.32E-05 | 0.004749 |
| 9124 | PDLIM1 | PDZ and LIM domain 1 | 0.366155 | 4.245903 | 6.29E-05 | 0.004749 |
| 57161 | PELI2 | pellino E3 ubiquitin protein ligase family member 2 | 0.443355 | 4.244141 | 6.33E-05 | 0.004749 |
|  |  |  | -0.26586 | -4.23877 | 6.45E-05 | 0.004826 |
| 60685 | ZFAND3 | zinc finger AN1-type containing 3 | 0.39344 | 4.237129 | 6.49E-05 | 0.004838 |
| 5775 | PTPN4 | protein tyrosine phosphatase non-receptor type 4 | -0.31778 | -4.23491 | 6.54E-05 | 0.00486 |
| 3300 | DNAJB2 | DnaJ heat shock protein family (Hsp40) member B2 | -0.53121 | -4.22862 | 6.69E-05 | 0.004955 |
| 51573 | GDE1 | glycerophosphodiester phosphodiesterase 1 | -0.57023 | -4.22541 | 6.77E-05 | 0.004995 |
| 1981 | EIF4G1 | eukaryotic translation initiation factor 4 gamma 1 | 0.354634 | 4.221816 | 6.85E-05 | 0.005013 |
| 2950 | GSTP1 | glutathione S-transferase pi 1 | 0.536654 | 4.22169 | 6.86E-05 | 0.005013 |
| 90378 | SAMD1 | sterile alpha motif domain containing 1 | 0.24261 | 4.221841 | 6.85E-05 | 0.005013 |
| 728318 | KRTAP9-1 | keratin associated protein 9-1 | 0.338561 | 4.21346 | 7.06E-05 | 0.005129 |
| 5520 | PPP2R2A | protein phosphatase 2 regulatory subunit Balpha | -0.42175 | -4.21417 | 7.04E-05 | 0.005129 |
| 26015 | RPAP1 | RNA polymerase II associated protein 1 | -0.25785 | -4.21214 | 7.1E-05 | 0.005137 |
| 6223 | RPS19 | ribosomal protein S19 | 0.337211 | 4.207568 | 7.21E-05 | 0.005188 |
| 7322 | UBE2D2 | ubiquitin conjugating enzyme E2 D2 | -0.39315 | -4.20758 | 7.21E-05 | 0.005188 |
| 9103 | FCGR2C | Fc fragment of IgG receptor IIc (gene/pseudogene) | 0.595951 | 4.199847 | 7.41E-05 | 0.005294 |
| 23478 | SEC11A | SEC11 homolog A, signal peptidase complex subunit | 0.333843 | 4.200343 | 7.4E-05 | 0.005294 |
| 25974 | MMACHC | metabolism of cobalamin associated C | 0.305942 | 4.199168 | 7.43E-05 | 0.005294 |
| 10133 | OPTN | optineurin | -0.49245 | -4.19445 | 7.56E-05 | 0.005367 |
| 5203 | PFDN4 | prefoldin subunit 4 | -0.58551 | -4.19083 | 7.65E-05 | 0.005403 |
| 9665 | MARF1 | meiosis regulator and mRNA stability factor 1 | -0.49043 | -4.19108 | 7.65E-05 | 0.005403 |
| 759 | CA1 | carbonic anhydrase 1 | 2.310341 | 4.184319 | 7.83E-05 | 0.005512 |
|  |  |  | 0.27818 | 4.161206 | 8.5E-05 | 0.005877 |
|  |  |  | 0.27818 | 4.161206 | 8.5E-05 | 0.005877 |
|  |  |  | 0.27818 | 4.161206 | 8.5E-05 | 0.005877 |
| 27257 | LSM1 | LSM1 homolog, mRNA degradation associated | -0.37496 | -4.1609 | 8.51E-05 | 0.005877 |
| 8669 | EIF3J | eukaryotic translation initiation factor 3 subunit J | -0.44781 | -4.16455 | 8.4E-05 | 0.005877 |
| 9218 | VAPA | VAMP associated protein A | -0.35804 | -4.16013 | 8.53E-05 | 0.005877 |
| 3572 | IL6ST | interleukin 6 signal transducer | 0.423934 | 4.160412 | 8.53E-05 | 0.005877 |
| 6827 | SUPT4H1 | SPT4 homolog, DSIF elongation factor subunit | -0.35078 | -4.1568 | 8.64E-05 | 0.005929 |
| 4798 | NFRKB | nuclear factor related to kappaB binding protein | 0.347757 | 4.155814 | 8.67E-05 | 0.005931 |
| 23091 | ZC3H13 | zinc finger CCCH-type containing 13 | -0.28153 | -4.15468 | 8.7E-05 | 0.005937 |
| 10767 | HBS1L | HBS1 like translational GTPase | -0.40411 | -4.14263 | 9.08E-05 | 0.006158 |
| 81565 | NDEL1 | nudE neurodevelopment protein 1 like 1 | -0.64159 | -4.14297 | 9.07E-05 | 0.006158 |
| 7328 | UBE2H | ubiquitin conjugating enzyme E2 H | -0.35322 | -4.13721 | 9.25E-05 | 0.006253 |
| 64770 | CCDC14 | coiled-coil domain containing 14 | -0.31403 | -4.13576 | 9.3E-05 | 0.006253 |
| 9611 | NCOR1 | nuclear receptor corepressor 1 | 0.269799 | 4.136217 | 9.29E-05 | 0.006253 |
|  |  |  | -0.45016 | -4.12308 | 9.73E-05 | 0.00652 |
| 1454 | CSNK1E | casein kinase 1 epsilon | 0.203477 | 4.116868 | 9.94E-05 | 0.006644 |
| 59286 | UBL5 | ubiquitin like 5 | -0.46529 | -4.11499 | 0.0001 | 0.006668 |
| 22818 | COPZ1 | coatomer protein complex subunit zeta 1 | -0.40267 | -4.11024 | 0.000102 | 0.006761 |
| 2806 | GOT2 | glutamic-oxaloacetic transaminase 2 | 0.369364 | 4.109152 | 0.000102 | 0.006767 |
| 389860 | PAGE2B | PAGE family member 2B | -0.5113 | -4.10678 | 0.000103 | 0.006803 |
| 10467 | ZNHIT1 | zinc finger HIT-type containing 1 | -0.43717 | -4.10302 | 0.000104 | 0.006874 |
| 6167 | RPL37 | ribosomal protein L37 | 0.31624 | 4.101294 | 0.000105 | 0.006896 |
| 79071 | ELOVL6 | ELOVL fatty acid elongase 6 | -0.74006 | -4.09994 | 0.000106 | 0.006908 |
| 4778 | NFE2 | nuclear factor, erythroid 2 | -0.44604 | -4.09702 | 0.000107 | 0.006959 |
| 162427 | RETREG3 | reticulophagy regulator family member 3 | -0.41236 | -4.09442 | 0.000108 | 0.007003 |
| 6777 | STAT5B | signal transducer and activator of transcription 5B | 0.331983 | 4.090672 | 0.000109 | 0.007075 |
| 4635 | MYL4 | myosin light chain 4 | -0.41281 | -4.07803 | 0.000114 | 0.007347 |
| 10975 | UQCR11 | ubiquinol-cytochrome c reductase, complex III subunit XI | -0.28481 | -4.07785 | 0.000114 | 0.007347 |
| 203569 | PAGE2 | PAGE family member 2 | -0.50902 | -4.07676 | 0.000114 | 0.007347 |
| 7763 | ZFAND5 | zinc finger AN1-type containing 5 | -0.38194 | -4.07666 | 0.000114 | 0.007347 |
|  |  |  | -0.50226 | -4.07232 | 0.000116 | 0.007438 |
| 80205 | CHD9 | chromodomain helicase DNA binding protein 9 | -0.46026 | -4.07069 | 0.000117 | 0.00746 |
| 29086 | BABAM1 | BRISC and BRCA1 A complex member 1 | -0.41767 | -4.06812 | 0.000118 | 0.007506 |
| 54386 | TERF2IP | TERF2 interacting protein | -0.35647 | -4.06624 | 0.000119 | 0.007534 |
| 5695 | PSMB7 | proteasome subunit beta 7 | -0.36609 | -4.06041 | 0.000121 | 0.007604 |
| 85019 | TMEM241 | transmembrane protein 241 | 0.609652 | 4.060912 | 0.000121 | 0.007604 |
|  |  |  | -0.52579 | -4.06179 | 0.000121 | 0.007604 |
| 54504 | CPVL | carboxypeptidase vitellogenic like | 0.939952 | 4.061981 | 0.000121 | 0.007604 |
| 535 | ATP6V0A1 | ATPase H+ transporting V0 subunit a1 | -0.28971 | -4.05918 | 0.000122 | 0.007615 |
| 3927 | LASP1 | LIM and SH3 protein 1 | 0.339293 | 4.058343 | 0.000122 | 0.007617 |
| 4057 | LTF | lactotransferrin | 1.36376 | 4.053942 | 0.000124 | 0.007713 |
| 7114 | TMSB4X | thymosin beta 4 X-linked | 0.325533 | 4.047734 | 0.000127 | 0.00786 |
| 1488 | CTBP2 | C-terminal binding protein 2 | -0.43425 | -4.04257 | 0.000129 | 0.007981 |
| 4128 | MAOA | monoamine oxidase A | -0.89412 | -4.03585 | 0.000132 | 0.008148 |
| 6143 | RPL19 | ribosomal protein L19 | 0.281323 | 4.033383 | 0.000133 | 0.008196 |
| 26258 | BLOC1S6 | biogenesis of lysosomal organelles complex 1 subunit 6 | -0.44755 | -4.02833 | 0.000136 | 0.008319 |
| 55219 | MACO1 | macoilin 1 | -0.53339 | -4.02462 | 0.000137 | 0.008404 |
| 9902 | MRC2 | mannose receptor C type 2 | -0.4476 | -4.01639 | 0.000141 | 0.008624 |
| 9519 | TBPL1 | TATA-box binding protein like 1 | -0.41503 | -4.01378 | 0.000143 | 0.008679 |
|  |  |  | -0.63682 | -4.01136 | 0.000144 | 0.008729 |
|  |  |  | 0.408305 | 4.007528 | 0.000146 | 0.00875 |
|  |  |  | 0.408305 | 4.007528 | 0.000146 | 0.00875 |
| 7555 | CNBP | CCHC-type zinc finger nucleic acid binding protein | -0.33625 | -4.00827 | 0.000145 | 0.00875 |
| 140460 | ASB7 | ankyrin repeat and SOCS box containing 7 | -0.4336 | -4.00979 | 0.000145 | 0.00875 |
| 23420 | NOMO1 | NODAL modulator 1 | -0.33054 | -4.00683 | 0.000146 | 0.00875 |
|  |  |  | 0.5449 | 4.003191 | 0.000148 | 0.008772 |
| 2934 | GSN | gelsolin | 0.457383 | 4.004518 | 0.000147 | 0.008772 |
|  |  |  | -0.39033 | -4.00401 | 0.000147 | 0.008772 |
| 133690 | CAPSL | calcyphosine like | 0.276755 | 4.003065 | 0.000148 | 0.008772 |
|  |  |  | -0.20133 | -4.0002 | 0.000149 | 0.008836 |
| 4217 | MAP3K5 | mitogen-activated protein kinase kinase kinase 5 | 0.345556 | 3.999405 | 0.00015 | 0.008837 |
| 7314 | UBB | ubiquitin B | -0.17667 | -3.99622 | 0.000152 | 0.008912 |
| 23365 | ARHGEF12 | Rho guanine nucleotide exchange factor 12 | -0.53651 | -3.99492 | 0.000152 | 0.008929 |
| 10580 | SORBS1 | sorbin and SH3 domain containing 1 | -0.37228 | -3.98339 | 0.000158 | 0.009269 |
| 55214 | P3H2 | prolyl 3-hydroxylase 2 | -0.3753 | -3.97591 | 0.000163 | 0.009487 |
| 23582 | CCNDBP1 | cyclin D1 binding protein 1 | -0.50565 | -3.97425 | 0.000163 | 0.009506 |
| 81570 | CLPB | ClpB homolog, mitochondrial AAA ATPase chaperonin | -0.3341 | -3.97382 | 0.000164 | 0.009506 |
| 23186 | RCOR1 | REST corepressor 1 | 0.531749 | 3.971094 | 0.000165 | 0.009572 |
| 55052 | MRPL20 | mitochondrial ribosomal protein L20 | 0.357441 | 3.965342 | 0.000169 | 0.009716 |
| 27343 | POLL | DNA polymerase lambda | -0.30924 | -3.96528 | 0.000169 | 0.009716 |
| 54469 | ZFAND6 | zinc finger AN1-type containing 6 | -0.32178 | -3.96302 | 0.00017 | 0.009767 |
| 1088 | CEACAM8 | carcinoembryonic antigen related cell adhesion molecule 8 | 0.479163 | 3.948415 | 0.000179 | 0.010245 |
| 83483 | PLVAP | plasmalemma vesicle associated protein | -0.95318 | -3.93913 | 0.000185 | 0.010468 |
| 7251 | TSG101 | tumor susceptibility 101 | -0.3246 | -3.9407 | 0.000184 | 0.010468 |
| 55791 | LRIF1 | ligand dependent nuclear receptor interacting factor 1 | 0.381239 | 3.938484 | 0.000185 | 0.010468 |
| 1389 | CREBL2 | cAMP responsive element binding protein like 2 | -0.30174 | -3.93866 | 0.000185 | 0.010468 |
|  |  |  | -0.25333 | -3.94068 | 0.000184 | 0.010468 |
| 3050 | HBZ | hemoglobin subunit zeta | -1.18986 | -3.93183 | 0.000189 | 0.010683 |
| 51246 | SHISA5 | shisa family member 5 | -0.30217 | -3.92924 | 0.000191 | 0.010751 |
| 64478 | CSMD1 | CUB and Sushi multiple domains 1 | -0.31414 | -3.92492 | 0.000194 | 0.010885 |
| 55831 | EMC3 | ER membrane protein complex subunit 3 | -0.41263 | -3.92387 | 0.000194 | 0.010897 |
| 51181 | DCXR | dicarbonyl and L-xylulose reductase | -0.2765 | -3.91861 | 0.000198 | 0.011068 |
| 10540 | DCTN2 | dynactin subunit 2 | 0.396584 | 3.917147 | 0.000199 | 0.011096 |
| 10124 | ARL4A | ADP ribosylation factor like GTPase 4A | -0.54143 | -3.91616 | 0.0002 | 0.011106 |
| 54439 | RBM27 | RNA binding motif protein 27 | -0.23437 | -3.91407 | 0.000201 | 0.011158 |
| 2618 | GART | phosphoribosylglycinamide formyltransferase, phosphoribosylglycinamide synthetase, phosphoribosylaminoimidazole synthetase | 0.346545 | 3.909562 | 0.000204 | 0.011304 |
| 23390 | ZDHHC17 | zinc finger DHHC-type containing 17 | -0.47763 | -3.90871 | 0.000205 | 0.011309 |
| 57410 | SCYL1 | SCY1 like pseudokinase 1 | 0.294198 | 3.906553 | 0.000206 | 0.011365 |
| 3587 | IL10RA | interleukin 10 receptor subunit alpha | 0.475375 | 3.898926 | 0.000212 | 0.011609 |
| 56654 | NPDC1 | neural proliferation, differentiation and control 1 | 0.213691 | 3.899002 | 0.000212 | 0.011609 |
| 10618 | TGOLN2 | trans-golgi network protein 2 | 0.209242 | 3.896306 | 0.000214 | 0.011685 |
| 10973 | ASCC3 | activating signal cointegrator 1 complex subunit 3 | 0.417632 | 3.894734 | 0.000215 | 0.011719 |
| 6521 | SLC4A1 | solute carrier family 4 member 1 (Diego blood group) | -0.38618 | -3.89141 | 0.000217 | 0.011768 |
| 402055 | SRRD | SRR1 domain containing | -0.48261 | -3.89244 | 0.000216 | 0.011768 |
| 782 | CACNB1 | calcium voltage-gated channel auxiliary subunit beta 1 | 0.316136 | 3.891823 | 0.000217 | 0.011768 |
| 6161 | RPL32 | ribosomal protein L32 | 0.246588 | 3.883402 | 0.000223 | 0.012044 |
| 151903 | CCDC12 | coiled-coil domain containing 12 | 0.242184 | 3.883199 | 0.000223 | 0.012044 |
| 4815 | NINJ2 | ninjurin 2 | -0.7071 | -3.87689 | 0.000228 | 0.012277 |
|  |  |  | 0.535585 | 3.870075 | 0.000234 | 0.012535 |
| 387521 | TMEM189 | transmembrane protein 189 | 0.430245 | 3.867882 | 0.000235 | 0.012599 |
| 9491 | PSMF1 | proteasome inhibitor subunit 1 | -0.41885 | -3.85909 | 0.000243 | 0.012951 |
|  |  |  | -0.25774 | -3.85381 | 0.000247 | 0.013154 |
| 55691 | FRMD4A | FERM domain containing 4A | -0.54398 | -3.84901 | 0.000251 | 0.013339 |
| 57687 | VAT1L | vesicle amine transport 1 like | -0.29677 | -3.84729 | 0.000252 | 0.013385 |
| 5197 | PF4V1 | platelet factor 4 variant 1 | 0.395925 | 3.845905 | 0.000254 | 0.013417 |
| 254428 | SLC41A1 | solute carrier family 41 member 1 | -0.38421 | -3.84338 | 0.000256 | 0.013437 |
| 6878 | TAF6 | TATA-box binding protein associated factor 6 | 0.389324 | 3.844314 | 0.000255 | 0.013437 |
| 8609 | KLF7 | Kruppel like factor 7 | 0.234157 | 3.843811 | 0.000255 | 0.013437 |
| 5876 | RABGGTB | Rab geranylgeranyltransferase subunit beta | -0.39986 | -3.83944 | 0.000259 | 0.0135 |
| 8896 | BUD31 | BUD31 homolog | -0.54175 | -3.8401 | 0.000259 | 0.0135 |
| 338596 | ST8SIA6 | ST8 alpha-N-acetyl-neuraminide alpha-2,8-sialyltransferase 6 | 0.294329 | 3.840937 | 0.000258 | 0.0135 |
| 6156 | RPL30 | ribosomal protein L30 | 0.183759 | 3.839287 | 0.000259 | 0.0135 |
| 64748 | PLPPR2 | phospholipid phosphatase related 2 | 0.356656 | 3.838293 | 0.00026 | 0.013514 |
| 7866 | IFRD2 | interferon related developmental regulator 2 | -0.28674 | -3.83687 | 0.000262 | 0.013548 |
| 2993 | GYPA | glycophorin A (MNS blood group) | -0.29115 | -3.83232 | 0.000266 | 0.013727 |
| 27246 | RNF115 | ring finger protein 115 | -0.31408 | -3.83015 | 0.000268 | 0.013765 |
| 80198 | MUS81 | MUS81 structure-specific endonuclease subunit | 0.25365 | 3.830599 | 0.000267 | 0.013765 |
|  |  |  | -0.33726 | -3.82251 | 0.000275 | 0.014093 |
|  |  |  | 0.494526 | 3.820773 | 0.000276 | 0.014144 |
| 23367 | LARP1 | La ribonucleoprotein domain family member 1 | -0.42242 | -3.8169 | 0.00028 | 0.014298 |
| 6622 | SNCA | synuclein alpha | -0.31006 | -3.81451 | 0.000282 | 0.014349 |
| 55122 | AKIRIN2 | akirin 2 | -0.38105 | -3.81504 | 0.000282 | 0.014349 |
| 5341 | PLEK | pleckstrin | 0.409186 | 3.808827 | 0.000288 | 0.014561 |
| 84071 | ARMC2 | armadillo repeat containing 2 | 0.355144 | 3.809055 | 0.000287 | 0.014561 |
| 22936 | ELL2 | elongation factor for RNA polymerase II 2 | -0.4126 | -3.8068 | 0.000289 | 0.014595 |
| 644054 | FAM25C | family with sequence similarity 25 member C | -0.18074 | -3.80727 | 0.000289 | 0.014595 |
| 26146 | TRAF3IP1 | TRAF3 interacting protein 1 | 0.276954 | 3.803779 | 0.000292 | 0.014712 |
| 439 | ASNA1 | arsA arsenite transporter, ATP-binding, homolog 1 (bacterial) | -0.39885 | -3.80292 | 0.000293 | 0.014722 |
| 8897 | MTMR3 | myotubularin related protein 3 | -0.39421 | -3.79736 | 0.000299 | 0.014967 |
| 10048 | RANBP9 | RAN binding protein 9 | -0.43065 | -3.79606 | 0.0003 | 0.014986 |
| 9628 | RGS6 | regulator of G protein signaling 6 | -0.6749 | -3.79566 | 0.000301 | 0.014986 |
| 22826 | DNAJC8 | DnaJ heat shock protein family (Hsp40) member C8 | 0.282736 | 3.793236 | 0.000303 | 0.015042 |
| 5920 | PLAAT4 | phospholipase A and acyltransferase 4 | 0.322543 | 3.792594 | 0.000304 | 0.015042 |
| 55851 | PSENEN | presenilin enhancer, gamma-secretase subunit | -0.40639 | -3.79317 | 0.000303 | 0.015042 |
| 6004 | RGS16 | regulator of G protein signaling 16 | -0.42497 | -3.78213 | 0.000315 | 0.015546 |
| 57596 | BEGAIN | brain enriched guanylate kinase associated | 0.327311 | 3.779448 | 0.000317 | 0.015653 |
| 167153 | TENT2 | terminal nucleotidyltransferase 2 | -0.29273 | -3.77643 | 0.000321 | 0.015772 |
| 10565 | ARFGEF1 | ADP ribosylation factor guanine nucleotide exchange factor 1 | 0.336279 | 3.773305 | 0.000324 | 0.015772 |
| 6794 | STK11 | serine/threonine kinase 11 | -0.33853 | -3.77334 | 0.000324 | 0.015772 |
| 5694 | PSMB6 | proteasome subunit beta 6 | -0.52899 | -3.77463 | 0.000323 | 0.015772 |
| 54820 | NDE1 | nudE neurodevelopment protein 1 | 0.262938 | 3.774998 | 0.000322 | 0.015772 |
| 23670 | CEMIP2 | cell migration inducing hyaluronidase 2 | -0.36026 | -3.77377 | 0.000323 | 0.015772 |
| 6641 | SNTB1 | syntrophin beta 1 | 0.370069 | 3.770641 | 0.000327 | 0.01588 |
| 1347 | COX7A2 | cytochrome c oxidase subunit 7A2 | -0.4159 | -3.76507 | 0.000333 | 0.01611 |
| 5055 | SERPINB2 | serpin family B member 2 | -0.2721 | -3.76517 | 0.000333 | 0.01611 |
| 54108 | CHRAC1 | chromatin accessibility complex subunit 1 | 0.345792 | 3.763113 | 0.000335 | 0.016181 |
| 53339 | BTBD1 | BTB domain containing 1 | 0.32734 | 3.758947 | 0.00034 | 0.016338 |
| 124801 | LSM12 | LSM12 homolog | -0.2723 | -3.75939 | 0.000339 | 0.016338 |
| 7305 | TYROBP | TYRO protein tyrosine kinase binding protein | 0.267913 | 3.755071 | 0.000344 | 0.016516 |
| 5436 | POLR2G | RNA polymerase II subunit G | 0.332354 | 3.753902 | 0.000346 | 0.016546 |
| 11275 | KLHL2 | kelch like family member 2 | -0.3276 | -3.75184 | 0.000348 | 0.016625 |
| 57465 | TBC1D24 | TBC1 domain family member 24 | 0.297273 | 3.749382 | 0.000351 | 0.016727 |
| 6169 | RPL38 | ribosomal protein L38 | 0.206373 | 3.746896 | 0.000354 | 0.016831 |
| 89910 | UBE3B | ubiquitin protein ligase E3B | -0.4208 | -3.74565 | 0.000355 | 0.016866 |
| 5082 | PDCL | phosducin like | -0.40787 | -3.74352 | 0.000358 | 0.016951 |
| 394 | ARHGAP5 | Rho GTPase activating protein 5 | -0.39947 | -3.73968 | 0.000363 | 0.017133 |
| 56829 | ZC3HAV1 | zinc finger CCCH-type containing, antiviral 1 | -0.35198 | -3.73565 | 0.000367 | 0.017329 |
| 5971 | RELB | RELB proto-oncogene, NF-kB subunit | 0.4114 | 3.73208 | 0.000372 | 0.0175 |
|  |  |  | 0.356783 | 3.721416 | 0.000385 | 0.018075 |
|  |  |  | -0.39118 | -3.72113 | 0.000386 | 0.018075 |
| 522 | ATP5PF | ATP synthase peripheral stalk subunit F6 | -0.32748 | -3.71616 | 0.000392 | 0.018298 |
| 55544 | RBM38 | RNA binding motif protein 38 | -0.4385 | -3.71677 | 0.000391 | 0.018298 |
| 80306 | MED28 | mediator complex subunit 28 | -0.25922 | -3.71558 | 0.000393 | 0.018298 |
| 91298 | C12orf29 | chromosome 12 open reading frame 29 | -0.72216 | -3.71465 | 0.000394 | 0.018311 |
| 64850 | ETNPPL | ethanolamine-phosphate phospho-lyase | 0.299126 | 3.714119 | 0.000395 | 0.018311 |
| 1054 | CEBPG | CCAAT enhancer binding protein gamma | 0.609397 | 3.711732 | 0.000398 | 0.018419 |
| 9943 | OXSR1 | oxidative stress responsive kinase 1 | -0.34817 | -3.71 | 0.0004 | 0.018488 |
| 6535 | SLC6A8 | solute carrier family 6 member 8 | -0.42187 | -3.70645 | 0.000405 | 0.018669 |
| 7358 | UGDH | UDP-glucose 6-dehydrogenase | 1.155738 | 3.705441 | 0.000406 | 0.018693 |
| 25897 | RNF19A | ring finger protein 19A, RBR E3 ubiquitin protein ligase | -0.46817 | -3.70342 | 0.000409 | 0.018743 |
| 90737 | PAGE5 | PAGE family member 5 | -0.36457 | -3.70346 | 0.000409 | 0.018743 |
| 30968 | STOML2 | stomatin like 2 | -0.36721 | -3.70093 | 0.000412 | 0.018824 |
| 8678 | BECN1 | beclin 1 | -0.35557 | -3.7009 | 0.000413 | 0.018824 |
| 6001 | RGS10 | regulator of G protein signaling 10 | -0.28052 | -3.69687 | 0.000418 | 0.018952 |
| 23191 | CYFIP1 | cytoplasmic FMR1 interacting protein 1 | 0.292204 | 3.698121 | 0.000416 | 0.018952 |
| 200014 | CC2D1B | coiled-coil and C2 domain containing 1B | 0.294488 | 3.697182 | 0.000418 | 0.018952 |
| 28973 | MRPS18B | mitochondrial ribosomal protein S18B | 0.299129 | 3.696427 | 0.000419 | 0.018952 |
| 8425 | LTBP4 | latent transforming growth factor beta binding protein 4 | 0.292987 | 3.695377 | 0.00042 | 0.01898 |
| 151790 | WDR49 | WD repeat domain 49 | 0.441638 | 3.693829 | 0.000422 | 0.01904 |
|  |  |  | 0.346721 | 3.691119 | 0.000426 | 0.019173 |
| 3145 | HMBS | hydroxymethylbilane synthase | -0.67473 | -3.69009 | 0.000428 | 0.0192 |
| 4637 | MYL6 | myosin light chain 6 | -0.21018 | -3.6874 | 0.000431 | 0.019333 |
| 116988 | AGAP3 | ArfGAP with GTPase domain, ankyrin repeat and PH domain 3 | 0.255004 | 3.685702 | 0.000434 | 0.019404 |
| 9046 | DOK2 | docking protein 2 | 0.445432 | 3.685032 | 0.000435 | 0.019409 |
| 6482 | ST3GAL1 | ST3 beta-galactoside alpha-2,3-sialyltransferase 1 | -0.58283 | -3.68298 | 0.000438 | 0.019502 |
| 3046 | HBE1 | hemoglobin subunit epsilon 1 | -0.70167 | -3.68198 | 0.000439 | 0.019509 |
| 54943 | DNAJC28 | DnaJ heat shock protein family (Hsp40) member C28 | -0.46794 | -3.68169 | 0.00044 | 0.019509 |
|  |  |  | 0.40213 | 3.681052 | 0.000441 | 0.019512 |
| 1286 | COL4A4 | collagen type IV alpha 4 chain | -0.28005 | -3.68006 | 0.000442 | 0.019537 |
| 54542 | RC3H2 | ring finger and CCCH-type domains 2 | -0.27607 | -3.67849 | 0.000444 | 0.019601 |
| 4973 | OLR1 | oxidized low density lipoprotein receptor 1 | 0.440404 | 3.676147 | 0.000448 | 0.019715 |
| 4259 | MGST3 | microsomal glutathione S-transferase 3 | -0.57241 | -3.66989 | 0.000457 | 0.020041 |
| 80315 | CPEB4 | cytoplasmic polyadenylation element binding protein 4 | -0.44964 | -3.66942 | 0.000458 | 0.020041 |
| 51761 | ATP8A2 | ATPase phospholipid transporting 8A2 | 0.240367 | 3.669846 | 0.000457 | 0.020041 |
| 1622 | DBI | diazepam binding inhibitor, acyl-CoA binding protein | 0.295817 | 3.667532 | 0.000461 | 0.020127 |
| 8498 | RANBP3 | RAN binding protein 3 | 0.208972 | 3.666818 | 0.000462 | 0.020135 |
| 51251 | NT5C3A | 5'-nucleotidase, cytosolic IIIA | -0.51055 | -3.66521 | 0.000464 | 0.020203 |
|  |  |  | -0.41451 | -3.66264 | 0.000468 | 0.020336 |
| 118 | ADD1 | adducin 1 | -0.31927 | -3.66146 | 0.00047 | 0.020337 |
| 6036 | RNASE2 | ribonuclease A family member 2 | -0.39489 | -3.66152 | 0.00047 | 0.020337 |
| 100507217 | LINC01578 | long intergenic non-protein coding RNA 1578 | -0.3134 | -3.65424 | 0.000481 | 0.020782 |
| 6135 | RPL11 | ribosomal protein L11 | 0.223224 | 3.653738 | 0.000482 | 0.020782 |
| 55796 | MBNL3 | muscleblind like splicing regulator 3 | -0.29483 | -3.64942 | 0.000489 | 0.021039 |
| 5663 | PSEN1 | presenilin 1 | 0.274565 | 3.647406 | 0.000492 | 0.021139 |
| 55088 | CCDC186 | coiled-coil domain containing 186 | -0.39454 | -3.6445 | 0.000497 | 0.021301 |
| 9253 | NUMBL | NUMB like endocytic adaptor protein | 0.338937 | 3.636937 | 0.00051 | 0.021746 |
| 79180 | EFHD2 | EF-hand domain family member D2 | 0.292603 | 3.63648 | 0.00051 | 0.021746 |
| 573 | BAG1 | BCL2 associated athanogene 1 | -0.56557 | -3.63753 | 0.000509 | 0.021746 |
| 79992 | AGPAT4-IT1 | AGPAT4 intronic transcript 1 | -0.37124 | -3.63498 | 0.000513 | 0.021771 |
| 23129 | PLXND1 | plexin D1 | 0.517334 | 3.63499 | 0.000513 | 0.021771 |
|  |  |  | 0.322354 | 3.63313 | 0.000516 | 0.021781 |
|  |  |  | 0.322354 | 3.63313 | 0.000516 | 0.021781 |
|  |  |  | 0.322354 | 3.63313 | 0.000516 | 0.021781 |
| 54206 | ERRFI1 | ERBB receptor feedback inhibitor 1 | -0.34561 | -3.63189 | 0.000518 | 0.021829 |
| 23476 | BRD4 | bromodomain containing 4 | -0.2434 | -3.62904 | 0.000523 | 0.021941 |
| 57691 | KIAA1586 | KIAA1586 | -0.34978 | -3.62964 | 0.000522 | 0.021941 |
| 7073 | TIAL1 | TIA1 cytotoxic granule associated RNA binding protein like 1 | -0.25667 | -3.62863 | 0.000524 | 0.021941 |
| 84444 | DOT1L | DOT1 like histone lysine methyltransferase | 0.275536 | 3.62747 | 0.000526 | 0.021984 |
| 10614 | HEXIM1 | HEXIM P-TEFb complex subunit 1 | -0.56609 | -3.62525 | 0.000529 | 0.022104 |
| 23352 | UBR4 | ubiquitin protein ligase E3 component n-recognin 4 | -0.22856 | -3.62391 | 0.000532 | 0.022159 |
| 54487 | DGCR8 | DGCR8 microprocessor complex subunit | -0.45576 | -3.62063 | 0.000538 | 0.022352 |
| 119692 | OR51S1 | olfactory receptor family 51 subfamily S member 1 | -0.21618 | -3.62015 | 0.000538 | 0.022352 |
| 8061 | FOSL1 | FOS like 1, AP-1 transcription factor subunit | 0.318234 | 3.619263 | 0.00054 | 0.022376 |
| 51547 | SIRT7 | sirtuin 7 | 0.254901 | 3.617776 | 0.000543 | 0.022444 |
| 56895 | AGPAT4 | 1-acylglycerol-3-phosphate O-acyltransferase 4 | -0.41011 | -3.61493 | 0.000548 | 0.022613 |
| 8991 | SELENBP1 | selenium binding protein 1 | -0.63208 | -3.61379 | 0.00055 | 0.022656 |
| 7468 | NSD2 | nuclear receptor binding SET domain protein 2 | -0.38125 | -3.61269 | 0.000552 | 0.022696 |
| 767558 | LUZP6 | leucine zipper protein 6 | -0.36921 | -3.61128 | 0.000554 | 0.022759 |
| 11236 | RNF139 | ring finger protein 139 | -0.40574 | -3.60654 | 0.000563 | 0.022987 |
| 6235 | RPS29 | ribosomal protein S29 | 0.282674 | 3.605836 | 0.000564 | 0.022987 |
| 10587 | TXNRD2 | thioredoxin reductase 2 | -0.42629 | -3.60724 | 0.000562 | 0.022987 |
|  |  |  | 0.247448 | 3.607056 | 0.000562 | 0.022987 |
| 63899 | NSUN3 | NOP2/Sun RNA methyltransferase 3 | -0.40249 | -3.60547 | 0.000565 | 0.022987 |
| 79005 | SCNM1 | sodium channel modifier 1 | -0.27288 | -3.60466 | 0.000566 | 0.023006 |
| 51247 | PAIP2 | poly(A) binding protein interacting protein 2 | -0.21788 | -3.602 | 0.000571 | 0.023166 |
| 11267 | SNF8 | SNF8 subunit of ESCRT-II | -0.30834 | -3.59685 | 0.000581 | 0.023516 |
| 6722 | SRF | serum response factor | 0.2986 | 3.595796 | 0.000583 | 0.023555 |
| 8925 | HERC1 | HECT and RLD domain containing E3 ubiquitin protein ligase family member 1 | -0.28334 | -3.5906 | 0.000593 | 0.023819 |
| 10150 | MBNL2 | muscleblind like splicing regulator 2 | 0.587953 | 3.589637 | 0.000595 | 0.023819 |
| 671 | BPI | bactericidal permeability increasing protein | 0.427512 | 3.589855 | 0.000594 | 0.023819 |
| 2539 | G6PD | glucose-6-phosphate dehydrogenase | 0.333637 | 3.59007 | 0.000594 | 0.023819 |
| 51024 | FIS1 | fission, mitochondrial 1 | -0.43013 | -3.58981 | 0.000594 | 0.023819 |
| 10766 | TOB2 | transducer of ERBB2, 2 | 0.248987 | 3.584188 | 0.000605 | 0.024203 |
| 23635 | SSBP2 | single stranded DNA binding protein 2 | 0.294834 | 3.583284 | 0.000607 | 0.024231 |
| 64795 | RMND5A | required for meiotic nuclear division 5 homolog A | -0.43988 | -3.58067 | 0.000612 | 0.024353 |
| 55014 | STX17 | syntaxin 17 | -0.36807 | -3.58073 | 0.000612 | 0.024353 |
| 352954 | CASTOR3 | CASTOR family member 3 | -0.45656 | -3.57779 | 0.000618 | 0.024538 |
| 6205 | RPS11 | ribosomal protein S11 | 0.239796 | 3.573999 | 0.000626 | 0.024799 |
| 4946 | OAZ1 | ornithine decarboxylase antizyme 1 | -0.29884 | -3.57193 | 0.00063 | 0.024836 |
| 9056 | SLC7A7 | solute carrier family 7 member 7 | 0.360601 | 3.572145 | 0.00063 | 0.024836 |
| 3692 | EIF6 | eukaryotic translation initiation factor 6 | 0.238114 | 3.572129 | 0.00063 | 0.024836 |
| 1396 | CRIP1 | cysteine rich protein 1 | 0.437367 | 3.568102 | 0.000638 | 0.025103 |
| 116151 | FAM210B | family with sequence similarity 210 member B | -0.35632 | -3.56743 | 0.000639 | 0.025114 |
| 7320 | UBE2B | ubiquitin conjugating enzyme E2 B | -0.2388 | -3.56459 | 0.000645 | 0.025302 |
| 92714 | ARRDC1 | arrestin domain containing 1 | 0.283373 | 3.563815 | 0.000647 | 0.025322 |
| 694 | BTG1 | BTG anti-proliferation factor 1 | -0.25434 | -3.55996 | 0.000655 | 0.025596 |
| 5018 | OXA1L | OXA1L mitochondrial inner membrane protein | 0.260217 | 3.55837 | 0.000658 | 0.025684 |
| 81631 | MAP1LC3B | microtubule associated protein 1 light chain 3 beta | -0.28226 | -3.555 | 0.000666 | 0.025922 |
| 549 | AUH | AU RNA binding methylglutaconyl-CoA hydratase | -0.28487 | -3.55126 | 0.000674 | 0.026172 |
| 6603 | SMARCD2 | SWI/SNF related, matrix associated, actin dependent regulator of chromatin, subfamily d, member 2 | 0.218535 | 3.550438 | 0.000676 | 0.026172 |
| 219333 | USP12 | ubiquitin specific peptidase 12 | -0.40684 | -3.55094 | 0.000674 | 0.026172 |
| 51043 | ZBTB7B | zinc finger and BTB domain containing 7B | 0.359335 | 3.544797 | 0.000688 | 0.026609 |
| 374969 | SVBP | small vasohibin binding protein | -0.48912 | -3.54397 | 0.00069 | 0.026635 |
| 23108 | RAP1GAP2 | RAP1 GTPase activating protein 2 | 0.2858 | 3.542539 | 0.000693 | 0.026712 |
| 1833 | EPYC | epiphycan | -0.27146 | -3.53891 | 0.000701 | 0.026982 |
| 3959 | LGALS3BP | galectin 3 binding protein | -0.17437 | -3.53657 | 0.000707 | 0.027141 |
| 8634 | RTCA | RNA 3'-terminal phosphate cyclase | -0.34841 | -3.53377 | 0.000713 | 0.02734 |
| 23261 | CAMTA1 | calmodulin binding transcription activator 1 | 0.26588 | 3.533243 | 0.000714 | 0.02734 |
| 64114 | TMBIM1 | transmembrane BAX inhibitor motif containing 1 | -0.17999 | -3.53241 | 0.000716 | 0.027368 |
| 57553 | MICAL3 | microtubule associated monooxygenase, calponin and LIM domain containing 3 | -0.4239 | -3.52938 | 0.000723 | 0.027497 |
|  |  |  | -0.33211 | -3.53033 | 0.000721 | 0.027497 |
| 23158 | TBC1D9 | TBC1 domain family member 9 | 0.473195 | 3.529868 | 0.000722 | 0.027497 |
| 22834 | ZNF652 | zinc finger protein 652 | 0.284332 | 3.527851 | 0.000727 | 0.027545 |
| 2588 | GALNS | galactosamine (N-acetyl)-6-sulfatase | 0.211097 | 3.527797 | 0.000727 | 0.027545 |
| 6175 | RPLP0 | ribosomal protein lateral stalk subunit P0 | 0.270356 | 3.521466 | 0.000742 | 0.027972 |
| 143244 | EIF5AL1 | eukaryotic translation initiation factor 5A like 1 | -0.27715 | -3.5224 | 0.00074 | 0.027972 |
| 3029 | HAGH | hydroxyacylglutathione hydrolase | -0.49384 | -3.52188 | 0.000741 | 0.027972 |
| 4149 | MAX | MYC associated factor X | -0.23267 | -3.52028 | 0.000745 | 0.028033 |
| 57179 | KIAA1191 | KIAA1191 | -0.34392 | -3.51841 | 0.000749 | 0.028108 |
| 51060 | TXNDC12 | thioredoxin domain containing 12 | 0.311194 | 3.518537 | 0.000749 | 0.028108 |
| 9590 | AKAP12 | A-kinase anchoring protein 12 | 0.284021 | 3.516147 | 0.000755 | 0.028267 |
|  |  |  | -0.32588 | -3.51544 | 0.000756 | 0.028284 |
| 27020 | NPTN | neuroplastin | 0.298294 | 3.513209 | 0.000762 | 0.028394 |
| 145873 | MESP2 | mesoderm posterior bHLH transcription factor 2 | -0.24269 | -3.51362 | 0.000761 | 0.028394 |
| 384 | ARG2 | arginase 2 | 0.976499 | 3.511695 | 0.000766 | 0.028486 |
| 203100 | HTRA4 | HtrA serine peptidase 4 | -0.29987 | -3.50942 | 0.000771 | 0.028648 |
| 26168 | SENP3 | SUMO specific peptidase 3 | 0.296068 | 3.508856 | 0.000773 | 0.028653 |
| 6566 | SLC16A1 | solute carrier family 16 member 1 | -0.7172 | -3.50494 | 0.000782 | 0.028881 |
| 80264 | ZNF430 | zinc finger protein 430 | 0.332441 | 3.504853 | 0.000783 | 0.028881 |
| 5293 | PIK3CD | phosphatidylinositol-4,5-bisphosphate 3-kinase catalytic subunit delta | 0.221876 | 3.5058 | 0.00078 | 0.028881 |
| 2035 | EPB41 | erythrocyte membrane protein band 4.1 | -0.1834 | -3.49867 | 0.000798 | 0.029174 |
| 246184 | CDC26 | cell division cycle 26 | -0.4786 | -3.4991 | 0.000797 | 0.029174 |
|  |  |  | 0.324698 | 3.498844 | 0.000798 | 0.029174 |
| 25852 | ARMC8 | armadillo repeat containing 8 | -0.23499 | -3.50086 | 0.000793 | 0.029174 |
| 100463486 | MTRNR2L8 | MT-RNR2 like 8 | 0.146426 | 3.49868 | 0.000798 | 0.029174 |
| 7514 | XPO1 | exportin 1 | -0.34448 | -3.50062 | 0.000793 | 0.029174 |
| 682 | BSG | basigin (Ok blood group) | -0.30516 | -3.49747 | 0.000801 | 0.029204 |
| 83849 | SYT15 | synaptotagmin 15 | 0.21665 | 3.497351 | 0.000802 | 0.029204 |
| 25844 | YIPF3 | Yip1 domain family member 3 | -0.36158 | -3.49413 | 0.00081 | 0.02946 |
| 2119 | ETV5 | ETS variant 5 | 0.48029 | 3.489186 | 0.000823 | 0.029752 |
| 409 | ARRB2 | arrestin beta 2 | 0.29707 | 3.488477 | 0.000825 | 0.029752 |
| 4299 | AFF1 | AF4/FMR2 family member 1 | -0.22529 | -3.488 | 0.000826 | 0.029752 |
| 339745 | SPOPL | speckle type BTB/POZ protein like | 0.345634 | 3.486563 | 0.00083 | 0.029752 |
| 55716 | LMBR1L | limb development membrane protein 1 like | 0.277689 | 3.488966 | 0.000823 | 0.029752 |
| 135152 | B3GAT2 | beta-1,3-glucuronyltransferase 2 | 0.302241 | 3.49024 | 0.00082 | 0.029752 |
| 5172 | SLC26A4 | solute carrier family 26 member 4 | -0.29161 | -3.48698 | 0.000829 | 0.029752 |
| 65008 | MRPL1 | mitochondrial ribosomal protein L1 | -0.33396 | -3.48871 | 0.000824 | 0.029752 |
| 101060376 | TBC1D3L | TBC1 domain family member 3L | 0.246474 | 3.486751 | 0.000829 | 0.029752 |
| 4953 | ODC1 | ornithine decarboxylase 1 | -0.41189 | -3.48599 | 0.000831 | 0.029759 |
| 2909 | ARHGAP35 | Rho GTPase activating protein 35 | 0.28583 | 3.484239 | 0.000836 | 0.029879 |
| 55177 | RMDN3 | regulator of microtubule dynamics 3 | -0.41011 | -3.48138 | 0.000844 | 0.030106 |
| 6210 | RPS15A | ribosomal protein S15a | 0.213617 | 3.479974 | 0.000848 | 0.030194 |
|  |  |  | 0.286919 | 3.478801 | 0.000851 | 0.03026 |
| 1471 | CST3 | cystatin C | 0.468924 | 3.476897 | 0.000856 | 0.030397 |
| 613212 | CTXN3 | cortexin 3 | -0.21577 | -3.47313 | 0.000866 | 0.030717 |
| 10260 | DENND4A | DENN domain containing 4A | -0.31204 | -3.46994 | 0.000875 | 0.030983 |
| 5440 | POLR2K | RNA polymerase II subunit K | -0.47996 | -3.46918 | 0.000877 | 0.031009 |
| 123688 | HYKK | hydroxylysine kinase | 0.274583 | 3.468413 | 0.000879 | 0.031037 |
| 25853 | DCAF12 | DDB1 and CUL4 associated factor 12 | -0.32225 | -3.46788 | 0.000881 | 0.031041 |
| 6863 | TAC1 | tachykinin precursor 1 | 0.279513 | 3.465854 | 0.000887 | 0.031193 |
| 11015 | KDELR3 | KDEL endoplasmic reticulum protein retention receptor 3 | 0.280445 | 3.465378 | 0.000888 | 0.031193 |
| 28964 | GIT1 | GIT ArfGAP 1 | 0.289908 | 3.464686 | 0.00089 | 0.031213 |
| 118924 | FRA10AC1 | FRA10A associated CGG repeat 1 | -0.28971 | -3.46343 | 0.000894 | 0.03129 |
| 123803 | NTAN1 | N-terminal asparagine amidase | -0.43321 | -3.46208 | 0.000897 | 0.031376 |
| 9334 | B4GALT5 | beta-1,4-galactosyltransferase 5 | 0.333281 | 3.460126 | 0.000903 | 0.031442 |
| 84333 | PCGF5 | polycomb group ring finger 5 | -0.3796 | -3.46029 | 0.000903 | 0.031442 |
| 401207 | C5orf63 | chromosome 5 open reading frame 63 | -0.45956 | -3.45998 | 0.000903 | 0.031442 |
| 147007 | TMEM199 | transmembrane protein 199 | -0.21755 | -3.45741 | 0.000911 | 0.031651 |
|  |  |  | 0.238536 | 3.45499 | 0.000918 | 0.031799 |
| 51510 | CHMP5 | charged multivesicular body protein 5 | -0.30642 | -3.4554 | 0.000917 | 0.031799 |
| 2879 | GPX4 | glutathione peroxidase 4 | 0.690971 | 3.45445 | 0.000919 | 0.031805 |
| 9551 | ATP5MF | ATP synthase membrane subunit f | -0.41945 | -3.45383 | 0.000921 | 0.031819 |
| 23480 | SEC61G | SEC61 translocon gamma subunit | -0.36424 | -3.45237 | 0.000926 | 0.031918 |
| 414060 | TBC1D3C | TBC1 domain family member 3C | 0.270551 | 3.451505 | 0.000928 | 0.031956 |
| 55209 | SETD5 | SET domain containing 5 | 0.222537 | 3.451039 | 0.00093 | 0.031956 |
|  |  |  | 0.225394 | 3.449576 | 0.000934 | 0.032057 |
|  |  |  | -2.239 | -3.4488 | 0.000936 | 0.032087 |
| 23067 | SETD1B | SET domain containing 1B, histone lysine methyltransferase | 0.301576 | 3.443562 | 0.000952 | 0.032577 |
| 6141 | RPL18 | ribosomal protein L18 | 0.20949 | 3.439171 | 0.000965 | 0.032985 |
| 5269 | SERPINB6 | serpin family B member 6 | 0.318193 | 3.438328 | 0.000968 | 0.033023 |
| 6500 | SKP1 | S-phase kinase associated protein 1 | -0.45422 | -3.4369 | 0.000972 | 0.033123 |
| 4985 | OPRD1 | opioid receptor delta 1 | 0.313789 | 3.435718 | 0.000976 | 0.033198 |
| 4100 | MAGEA1 | MAGE family member A1 | -0.20545 | -3.43479 | 0.000979 | 0.033246 |
| 51082 | POLR1D | RNA polymerase I and III subunit D | -0.40092 | -3.43047 | 0.000992 | 0.033553 |
| 140461 | ASB8 | ankyrin repeat and SOCS box containing 8 | -0.29506 | -3.43054 | 0.000992 | 0.033553 |
| 51192 | CKLF | chemokine like factor | 0.232569 | 3.431284 | 0.00099 | 0.033553 |
|  |  |  | 0.265064 | 3.428899 | 0.000997 | 0.03367 |
| 23305 | ACSL6 | acyl-CoA synthetase long chain family member 6 | -0.44691 | -3.4284 | 0.000999 | 0.033673 |
| 728358 | DEFA1B | defensin alpha 1B | 1.49545 | 3.425588 | 0.001008 | 0.033723 |
| 1667 | DEFA1 | defensin alpha 1 | 1.49545 | 3.425588 | 0.001008 | 0.033723 |
| 115201 | ATG4A | autophagy related 4A cysteine peptidase | -0.51805 | -3.4258 | 0.001007 | 0.033723 |
| 25932 | CLIC4 | chloride intracellular channel 4 | 0.448009 | 3.426432 | 0.001005 | 0.033723 |
| 7515 | XRCC1 | X-ray repair cross complementing 1 | 0.163159 | 3.426634 | 0.001004 | 0.033723 |
| 137886 | UBXN2B | UBX domain protein 2B | -0.29706 | -3.42273 | 0.001017 | 0.033966 |
| 6193 | RPS5 | ribosomal protein S5 | 0.247926 | 3.42239 | 0.001018 | 0.033966 |
| 4317 | MMP8 | matrix metallopeptidase 8 | 1.264845 | 3.421252 | 0.001022 | 0.033988 |
| 7296 | TXNRD1 | thioredoxin reductase 1 | -0.31579 | -3.42152 | 0.001021 | 0.033988 |
| 7311 | UBA52 | ubiquitin A-52 residue ribosomal protein fusion product 1 | -0.179 | -3.42035 | 0.001025 | 0.034036 |
| 467 | ATF3 | activating transcription factor 3 | 0.416798 | 3.419121 | 0.001029 | 0.034118 |
| 57610 | RANBP10 | RAN binding protein 10 | -0.50458 | -3.41688 | 0.001036 | 0.03431 |
| 9748 | SLK | STE20 like kinase | 0.215416 | 3.413371 | 0.001048 | 0.034643 |
| 6202 | RPS8 | ribosomal protein S8 | 0.225012 | 3.41233 | 0.001051 | 0.034706 |
| 60492 | CCDC90B | coiled-coil domain containing 90B | -0.38933 | -3.40959 | 0.00106 | 0.034957 |
| 114885 | OSBPL11 | oxysterol binding protein like 11 | 0.355842 | 3.408937 | 0.001062 | 0.034978 |
| 57724 | EPG5 | ectopic P-granules autophagy protein 5 homolog | -0.35952 | -3.40711 | 0.001068 | 0.035129 |
| 58528 | RRAGD | Ras related GTP binding D | 0.325139 | 3.405526 | 0.001074 | 0.035254 |
| 55437 | STRADB | STE20 related adaptor beta | -0.31027 | -3.40458 | 0.001077 | 0.035309 |
| 5742 | PTGS1 | prostaglandin-endoperoxide synthase 1 | 0.305407 | 3.403429 | 0.001081 | 0.035334 |
| 642 | BLMH | bleomycin hydrolase | 0.322669 | 3.403812 | 0.00108 | 0.035334 |
| 9440 | MED17 | mediator complex subunit 17 | 0.340765 | 3.402595 | 0.001084 | 0.035376 |
| 11260 | XPOT | exportin for tRNA | 0.280048 | 3.397948 | 0.0011 | 0.035695 |
| 26061 | HACL1 | 2-hydroxyacyl-CoA lyase 1 | -0.30646 | -3.39792 | 0.0011 | 0.035695 |
| 4851 | NOTCH1 | notch receptor 1 | 0.224403 | 3.39823 | 0.001099 | 0.035695 |
| 6712 | SPTBN2 | spectrin beta, non-erythrocytic 2 | 0.21119 | 3.39903 | 0.001096 | 0.035695 |
| 11337 | GABARAP | GABA type A receptor-associated protein | -0.20823 | -3.39743 | 0.001102 | 0.035699 |
| 3190 | HNRNPK | heterogeneous nuclear ribonucleoprotein K | -0.28098 | -3.3949 | 0.00111 | 0.035842 |
| 11270 | NRM | nurim | 0.249155 | 3.394355 | 0.001112 | 0.035842 |
| 2673 | GFPT1 | glutamine--fructose-6-phosphate transaminase 1 | 0.252379 | 3.39445 | 0.001112 | 0.035842 |
| 7185 | TRAF1 | TNF receptor associated factor 1 | 0.243145 | 3.394873 | 0.001111 | 0.035842 |
| 7390 | UROS | uroporphyrinogen III synthase | -0.521 | -3.39049 | 0.001126 | 0.036229 |
| 10519 | CIB1 | calcium and integrin binding 1 | 0.330537 | 3.38742 | 0.001137 | 0.036506 |
| 390792 | KRT39 | keratin 39 | 0.229417 | 3.387164 | 0.001138 | 0.036506 |
| 129563 | DIS3L2 | DIS3 like 3'-5' exoribonuclease 2 | 0.235427 | 3.382911 | 0.001153 | 0.036946 |
| 6660 | SOX5 | SRY-box 5 | 0.243432 | 3.380871 | 0.001161 | 0.037131 |
| 10930 | APOBEC2 | apolipoprotein B mRNA editing enzyme catalytic subunit 2 | -0.25996 | -3.37863 | 0.001169 | 0.037341 |
| 7171 | TPM4 | tropomyosin 4 | 0.209496 | 3.3758 | 0.001179 | 0.037621 |
|  |  |  | 0.307726 | 3.375279 | 0.001181 | 0.037629 |
| 143686 | SESN3 | sestrin 3 | -0.45633 | -3.37216 | 0.001193 | 0.037914 |
| 57175 | CORO1B | coronin 1B | 0.264617 | 3.371983 | 0.001193 | 0.037914 |
| 1668 | DEFA3 | defensin alpha 3 | 1.369764 | 3.369741 | 0.001202 | 0.038128 |
| 5274 | SERPINI1 | serpin family I member 1 | -0.84737 | -3.36837 | 0.001207 | 0.038239 |
| 284194 | LGALS9B | galectin 9B | 0.248612 | 3.367563 | 0.00121 | 0.038282 |
| 1436 | CSF1R | colony stimulating factor 1 receptor | 0.39969 | 3.366501 | 0.001214 | 0.038356 |
| 55789 | DEPDC1B | DEP domain containing 1B | 0.283552 | 3.365483 | 0.001218 | 0.038425 |
|  |  |  | 0.281665 | 3.361775 | 0.001232 | 0.03882 |
| 3357 | HTR2B | 5-hydroxytryptamine receptor 2B | -0.31502 | -3.36045 | 0.001237 | 0.038927 |
| 6782 | HSPA13 | heat shock protein family A (Hsp70) member 13 | -0.54525 | -3.35849 | 0.001245 | 0.039055 |
| 4242 | MFNG | MFNG O-fucosylpeptide 3-beta-N-acetylglucosaminyltransferase | 0.217124 | 3.358814 | 0.001244 | 0.039055 |
| 167227 | DCP2 | decapping mRNA 2 | -0.19657 | -3.35807 | 0.001247 | 0.039055 |
|  |  |  | 0.297914 | 3.355256 | 0.001258 | 0.039184 |
|  |  |  | 0.297914 | 3.355256 | 0.001258 | 0.039184 |
|  |  |  | 0.297914 | 3.355256 | 0.001258 | 0.039184 |
|  |  |  | -0.40431 | -3.35591 | 0.001255 | 0.039184 |
| 460 | ASTN1 | astrotactin 1 | 0.206245 | 3.352975 | 0.001267 | 0.03941 |
| 767 | CA8 | carbonic anhydrase 8 | -0.53164 | -3.35203 | 0.00127 | 0.039472 |
| 23450 | SF3B3 | splicing factor 3b subunit 3 | -0.433 | -3.3491 | 0.001282 | 0.039781 |
|  |  |  | 0.263002 | 3.346398 | 0.001293 | 0.039954 |
| 55776 | SAYSD1 | SAYSVFN motif domain containing 1 | -0.20543 | -3.34671 | 0.001292 | 0.039954 |
| 79969 | ATAT1 | alpha tubulin acetyltransferase 1 | 0.234709 | 3.346402 | 0.001293 | 0.039954 |
| 1891 | ECH1 | enoyl-CoA hydratase 1 | 0.275221 | 3.345813 | 0.001295 | 0.039972 |
| 7102 | TSPAN7 | tetraspanin 7 | -0.62442 | -3.34437 | 0.001301 | 0.040098 |
| 760 | CA2 | carbonic anhydrase 2 | 0.382541 | 3.334174 | 0.001343 | 0.040528 |
| 343171 | OR2W3 | olfactory receptor family 2 subfamily W member 3 | -0.46345 | -3.3355 | 0.001338 | 0.040528 |
| 85403 | EAF1 | ELL associated factor 1 | -0.26996 | -3.33485 | 0.00134 | 0.040528 |
| 5054 | SERPINE1 | serpin family E member 1 | 0.348007 | 3.335559 | 0.001338 | 0.040528 |
| 5590 | PRKCZ | protein kinase C zeta | 0.243513 | 3.338861 | 0.001324 | 0.040528 |
| 6224 | RPS20 | ribosomal protein S20 | 0.17849 | 3.33467 | 0.001341 | 0.040528 |
| 2519 | FUCA2 | alpha-L-fucosidase 2 | 0.283956 | 3.334424 | 0.001342 | 0.040528 |
| 347148 | QRFP | pyroglutamylated RFamide peptide | 0.197877 | 3.33737 | 0.00133 | 0.040528 |
|  |  |  | -0.2103 | -3.33442 | 0.001342 | 0.040528 |
|  |  |  | -0.2103 | -3.33442 | 0.001342 | 0.040528 |
|  |  |  | -0.2103 | -3.33442 | 0.001342 | 0.040528 |
|  |  |  | -0.2103 | -3.33442 | 0.001342 | 0.040528 |
|  |  |  | -0.2103 | -3.33442 | 0.001342 | 0.040528 |
|  |  |  | -0.2103 | -3.33442 | 0.001342 | 0.040528 |
|  |  |  | -0.2103 | -3.33442 | 0.001342 | 0.040528 |
| 403 | ARL3 | ADP ribosylation factor like GTPase 3 | 0.304005 | 3.334031 | 0.001344 | 0.040528 |
| 100129094 | BTNL10 | butyrophilin like 10 | -0.35538 | -3.33227 | 0.001351 | 0.040675 |
| 55701 | ARHGEF40 | Rho guanine nucleotide exchange factor 40 | -0.3444 | -3.33201 | 0.001352 | 0.040675 |
| 79641 | ROGDI | rogdi atypical leucine zipper | -0.37725 | -3.33004 | 0.001361 | 0.040796 |
| 23649 | POLA2 | DNA polymerase alpha 2, accessory subunit | 0.277847 | 3.329786 | 0.001362 | 0.040796 |
| 65056 | GPBP1 | GC-rich promoter binding protein 1 | -0.21263 | -3.3304 | 0.001359 | 0.040796 |
| 2863 | GPR39 | G protein-coupled receptor 39 | -0.29027 | -3.32821 | 0.001369 | 0.040942 |
| 9875 | URB1 | URB1 ribosome biogenesis homolog | -0.239 | -3.32148 | 0.001398 | 0.041754 |
| 4673 | NAP1L1 | nucleosome assembly protein 1 like 1 | 0.379983 | 3.321025 | 0.001399 | 0.041757 |
| 55501 | CHST12 | carbohydrate sulfotransferase 12 | 0.221782 | 3.320383 | 0.001402 | 0.041785 |
| 9087 | TMSB4Y | thymosin beta 4 Y-linked | 0.252107 | 3.318763 | 0.001409 | 0.041941 |
| 389336 | C5orf46 | chromosome 5 open reading frame 46 | 0.238149 | 3.317861 | 0.001413 | 0.042004 |
| 79724 | ZNF768 | zinc finger protein 768 | 0.270368 | 3.316109 | 0.001421 | 0.042122 |
| 29799 | YPEL1 | yippee like 1 | 0.254652 | 3.316144 | 0.001421 | 0.042122 |
| 221545 | C6orf136 | chromosome 6 open reading frame 136 | 0.280428 | 3.313885 | 0.001431 | 0.042264 |
|  |  |  | 0.268909 | 3.31376 | 0.001431 | 0.042264 |
|  |  |  | 0.268909 | 3.31376 | 0.001431 | 0.042264 |
| 100463289 | MTRNR2L5 | MT-RNR2 like 5 | 0.341662 | 3.312982 | 0.001435 | 0.042311 |
| 8318 | CDC45 | cell division cycle 45 | 0.277327 | 3.311876 | 0.00144 | 0.042401 |
|  |  |  | 0.220034 | 3.309323 | 0.001451 | 0.042612 |
| 55752 | SEPTIN11 | septin 11 | 0.309693 | 3.309025 | 0.001453 | 0.042612 |
| 18 | ABAT | 4-aminobutyrate aminotransferase | -0.31438 | -3.30982 | 0.001449 | 0.042612 |
|  |  |  | -0.9237 | -3.3086 | 0.001455 | 0.042612 |
| 58475 | MS4A7 | membrane spanning 4-domains A7 | 0.586874 | 3.307177 | 0.001461 | 0.042746 |
| 22794 | CASC3 | CASC3 exon junction complex subunit | -0.38261 | -3.30664 | 0.001463 | 0.042762 |
| 8861 | LDB1 | LIM domain binding 1 | 0.198904 | 3.303804 | 0.001476 | 0.043008 |
|  |  |  | 0.226709 | 3.303538 | 0.001478 | 0.043008 |
| 221357 | GSTA5 | glutathione S-transferase alpha 5 | 0.249736 | 3.303635 | 0.001477 | 0.043008 |
| 5710 | PSMD4 | proteasome 26S subunit, non-ATPase 4 | -0.26819 | -3.30265 | 0.001482 | 0.043071 |
|  |  |  | 0.215126 | 3.301466 | 0.001487 | 0.043174 |
| 493856 | CISD2 | CDGSH iron sulfur domain 2 | -0.37189 | -3.30094 | 0.00149 | 0.043189 |
| 2054 | STX2 | syntaxin 2 | 0.343269 | 3.299063 | 0.001498 | 0.043385 |
| 7036 | TFR2 | transferrin receptor 2 | -0.28515 | -3.29374 | 0.001523 | 0.044018 |
| 112869 | SGF29 | SAGA complex associated factor 29 | 0.197325 | 3.292851 | 0.001527 | 0.044018 |
| 202559 | KHDRBS2 | KH RNA binding domain containing, signal transduction associated 2 | 0.280038 | 3.293529 | 0.001524 | 0.044018 |
| 3716 | JAK1 | Janus kinase 1 | 0.235019 | 3.29273 | 0.001528 | 0.044018 |
| 84669 | USP32 | ubiquitin specific peptidase 32 | -0.24566 | -3.29178 | 0.001532 | 0.044036 |
| 57623 | ZFAT | zinc finger and AT-hook domain containing | -0.20453 | -3.29208 | 0.001531 | 0.044036 |
| 9879 | DDX46 | DEAD-box helicase 46 | -0.23787 | -3.29075 | 0.001537 | 0.04412 |
| 7178 | TPT1 | tumor protein, translationally-controlled 1 | 0.189779 | 3.28715 | 0.001554 | 0.044557 |
| 6147 | RPL23A | ribosomal protein L23a | 0.209374 | 3.285356 | 0.001563 | 0.044748 |
| 22863 | ATG14 | autophagy related 14 | -0.41497 | -3.28171 | 0.001581 | 0.045198 |
| 51635 | DHRS7 | dehydrogenase/reductase 7 | 0.344565 | 3.277952 | 0.001599 | 0.045667 |
| 54585 | LZTFL1 | leucine zipper transcription factor like 1 | -0.57721 | -3.27437 | 0.001617 | 0.045967 |
| 3611 | ILK | integrin linked kinase | 0.266736 | 3.273789 | 0.00162 | 0.045967 |
| 128077 | LIX1L | limb and CNS expressed 1 like | 0.238449 | 3.27415 | 0.001618 | 0.045967 |
| 57105 | CYSLTR2 | cysteinyl leukotriene receptor 2 | 0.272959 | 3.274983 | 0.001614 | 0.045967 |
| 9559 | VPS26A | VPS26, retromer complex component A | -0.3629 | -3.27497 | 0.001614 | 0.045967 |
| 28231 | SLCO4A1 | solute carrier organic anion transporter family member 4A1 | -0.31588 | -3.27329 | 0.001622 | 0.045981 |
| 5586 | PKN2 | protein kinase N2 | -0.24218 | -3.27206 | 0.001629 | 0.046097 |
| 130 | ADH6 | alcohol dehydrogenase 6 (class V) | -0.23263 | -3.26952 | 0.001641 | 0.046401 |
| 6307 | MSMO1 | methylsterol monooxygenase 1 | -0.56787 | -3.26904 | 0.001644 | 0.046412 |
| 54851 | ANKRD49 | ankyrin repeat domain 49 | -0.32046 | -3.2679 | 0.00165 | 0.046516 |
| 351 | APP | amyloid beta precursor protein | 0.303854 | 3.267293 | 0.001653 | 0.046545 |
| 408 | ARRB1 | arrestin beta 1 | 0.327543 | 3.266523 | 0.001657 | 0.046598 |
| 6450 | SH3BGR | SH3 domain binding glutamate rich protein | -0.28408 | -3.26516 | 0.001664 | 0.046677 |
| 2235 | FECH | ferrochelatase | -0.1947 | -3.26539 | 0.001662 | 0.046677 |
| 5879 | RAC1 | Rac family small GTPase 1 | 0.232524 | 3.263782 | 0.001671 | 0.046818 |
| 93594 | TBC1D31 | TBC1 domain family member 31 | -0.32307 | -3.26218 | 0.001679 | 0.04699 |
|  |  |  | 0.255491 | 3.260685 | 0.001687 | 0.047149 |
| 127396 | ZNF684 | zinc finger protein 684 | 0.204647 | 3.259178 | 0.001694 | 0.04731 |
| 4680 | CEACAM6 | carcinoembryonic antigen related cell adhesion molecule 6 | 0.315773 | 3.256727 | 0.001707 | 0.047363 |
| 164091 | PAQR7 | progestin and adipoQ receptor family member 7 | 0.298153 | 3.258311 | 0.001699 | 0.047363 |
| 126364 | LRRC25 | leucine rich repeat containing 25 | 0.31433 | 3.257753 | 0.001702 | 0.047363 |
| 57142 | RTN4 | reticulon 4 | 0.308167 | 3.256409 | 0.001709 | 0.047363 |
| 100271927 | RASA4B | RAS p21 protein activator 4B | 0.184196 | 3.256757 | 0.001707 | 0.047363 |
| 326624 | RAB37 | RAB37, member RAS oncogene family | 0.283013 | 3.256905 | 0.001706 | 0.047363 |
| 94056 | SYAP1 | synapse associated protein 1 | -0.31765 | -3.25554 | 0.001714 | 0.04743 |
| 84236 | RHBDD1 | rhomboid domain containing 1 | -0.35679 | -3.25163 | 0.001734 | 0.047946 |
| 197135 | PATL2 | PAT1 homolog 2 | 0.25528 | 3.249736 | 0.001744 | 0.048123 |
|  |  |  | 0.236972 | 3.249631 | 0.001745 | 0.048123 |
| 10247 | RIDA | reactive intermediate imine deaminase A homolog | -0.45804 | -3.24814 | 0.001753 | 0.048194 |
| 51271 | UBAP1 | ubiquitin associated protein 1 | -0.36897 | -3.24634 | 0.001763 | 0.048194 |
| 7247 | TSN | translin | -0.25141 | -3.24499 | 0.00177 | 0.048194 |
| 7791 | ZYX | zyxin | 0.236754 | 3.248652 | 0.00175 | 0.048194 |
| 51177 | PLEKHO1 | pleckstrin homology domain containing O1 | 0.245213 | 3.245066 | 0.00177 | 0.048194 |
| 79070 | POGLUT2 | protein O-glucosyltransferase 2 | 0.304504 | 3.244938 | 0.00177 | 0.048194 |
| 64208 | POPDC3 | popeye domain containing 3 | -0.25856 | -3.24401 | 0.001775 | 0.048194 |
|  |  |  | 0.245744 | 3.2443 | 0.001774 | 0.048194 |
|  |  |  | 0.245744 | 3.2443 | 0.001774 | 0.048194 |
|  |  |  | 0.245744 | 3.2443 | 0.001774 | 0.048194 |
|  |  |  | 0.245744 | 3.2443 | 0.001774 | 0.048194 |
|  |  |  | 0.245744 | 3.2443 | 0.001774 | 0.048194 |
|  |  |  | 0.245744 | 3.2443 | 0.001774 | 0.048194 |
| 135112 | NCOA7 | nuclear receptor coactivator 7 | 0.477561 | 3.242265 | 0.001785 | 0.048395 |
| 23657 | SLC7A11 | solute carrier family 7 member 11 | 0.601386 | 3.239403 | 0.001801 | 0.048763 |
| 339768 | ESPNL | espin like | 0.156418 | 3.238459 | 0.001806 | 0.048846 |
| 51319 | RSRC1 | arginine and serine rich coiled-coil 1 | -0.3642 | -3.23683 | 0.001815 | 0.04903 |
| 60684 | TRAPPC11 | trafficking protein particle complex 11 | 0.232549 | 3.236354 | 0.001817 | 0.049044 |
| 571 | BACH1 | BTB domain and CNC homolog 1 | -0.28787 | -3.23421 | 0.001829 | 0.049249 |
| 2309 | FOXO3 | forkhead box O3 | -0.25248 | -3.23441 | 0.001828 | 0.049249 |
|  |  |  | -0.35171 | -3.23199 | 0.001842 | 0.049525 |
| 23132 | RAD54L2 | RAD54 like 2 | 0.303138 | 3.231148 | 0.001847 | 0.049594 |
