# Supplementary figures and images for: Whole genome methylation and transcriptome analyses to identify risk for cerebral palsy (CP) in extremely low gestational age neonates (ELGAN)
Source: Sci Rep. 2021 Mar 5;11:5305. doi: 10.1038/s41598-021-84214-9 (PMC7935929; doi:10.1038/s41598-021-84214-9)

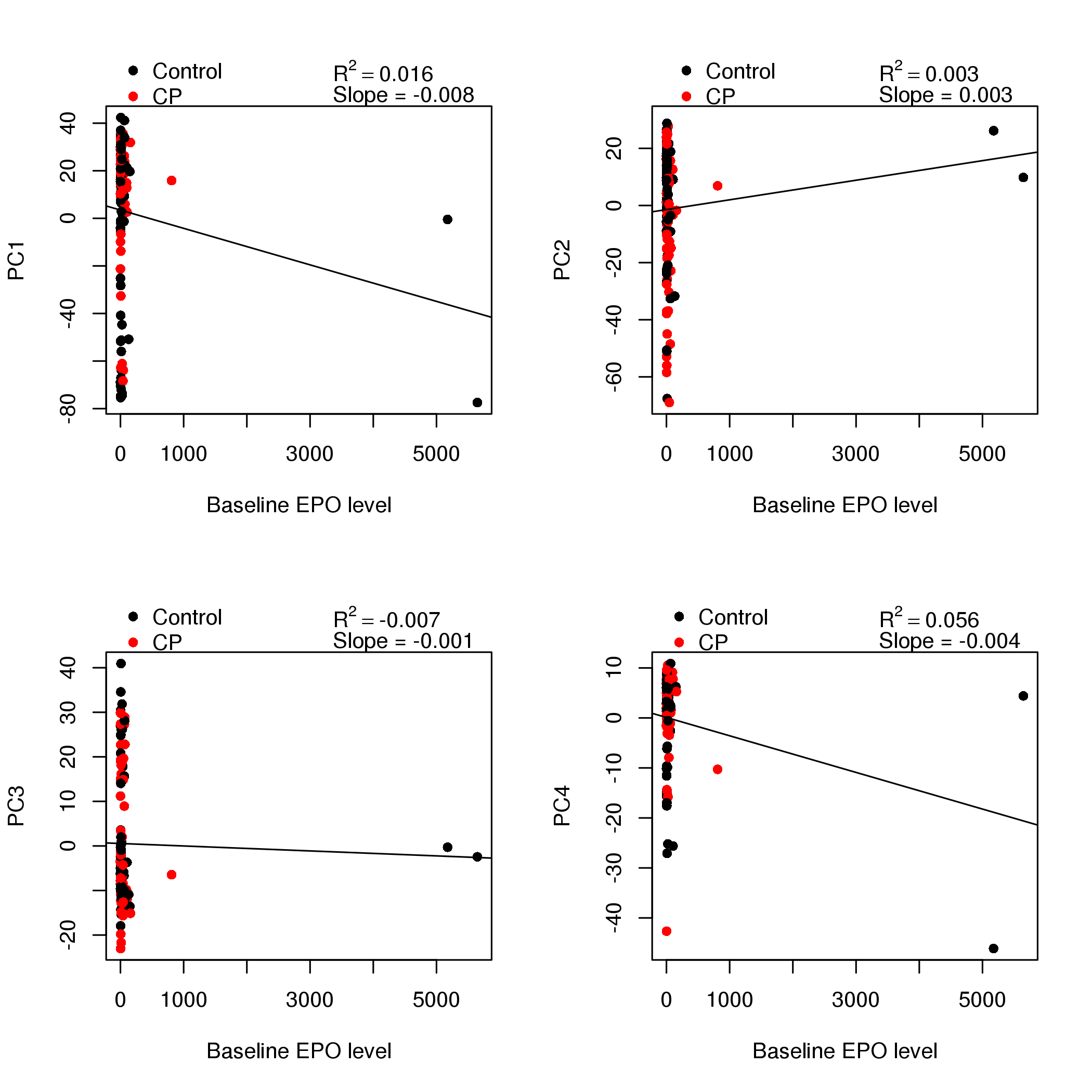

Supplement: Supplementary file 2 — Supplementary Figure S1. [file 41598_2021_84214_MOESM2_ESM.tiff]

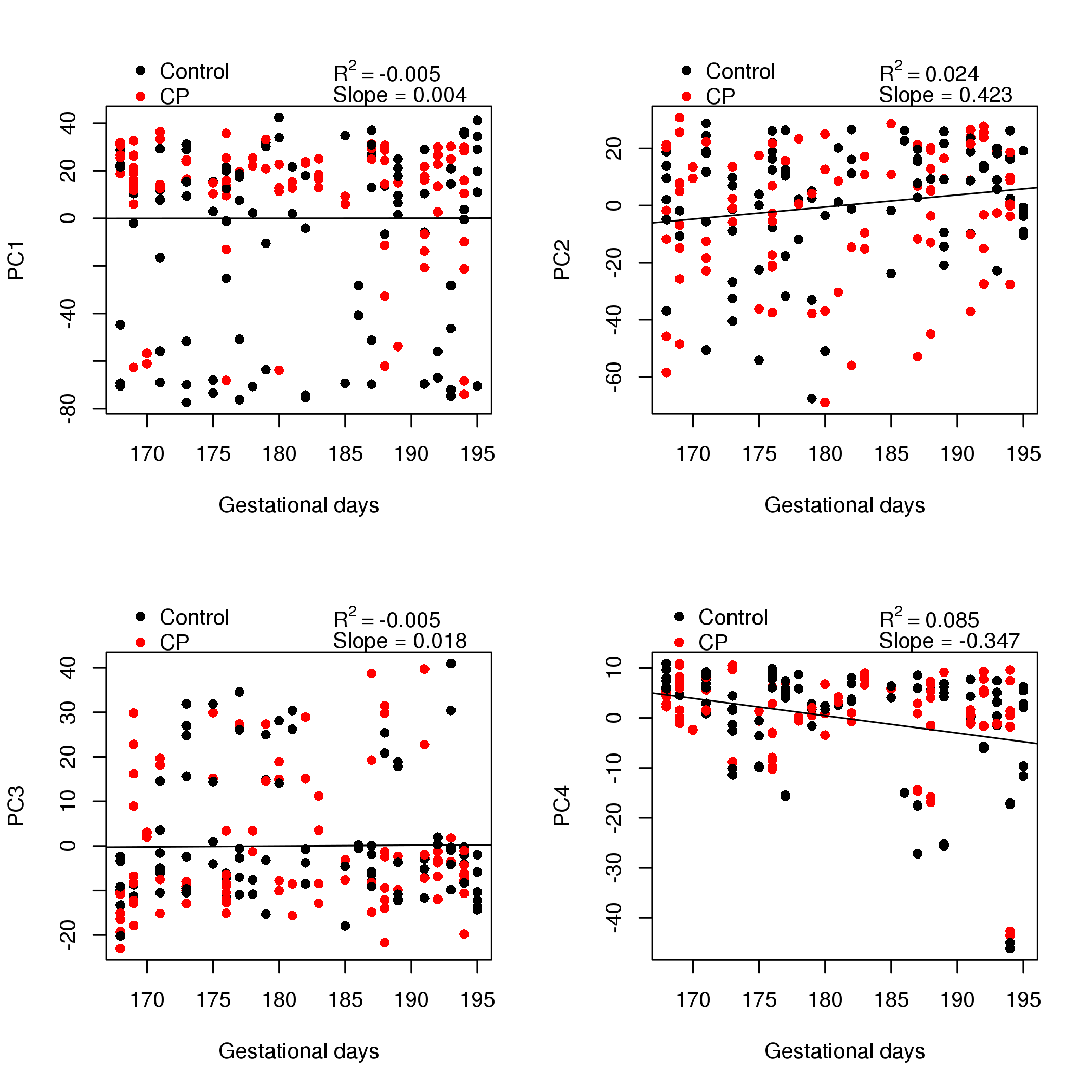

Supplement: Supplementary file 3 — Supplementary Figure S2. [file 41598_2021_84214_MOESM3_ESM.tiff]

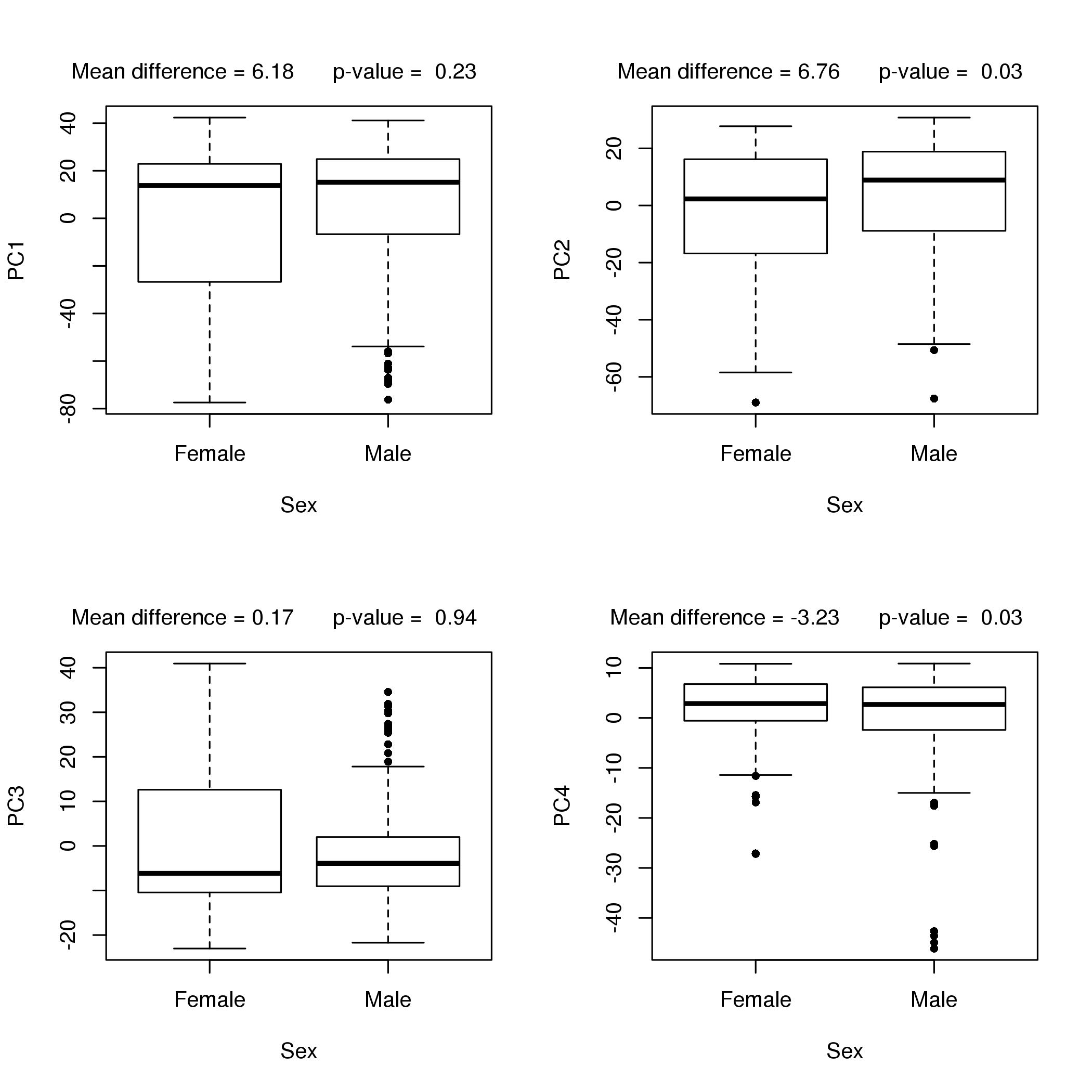

Supplement: Supplementary file 4 — Supplementary Figure S3. [file 41598_2021_84214_MOESM4_ESM.tiff]

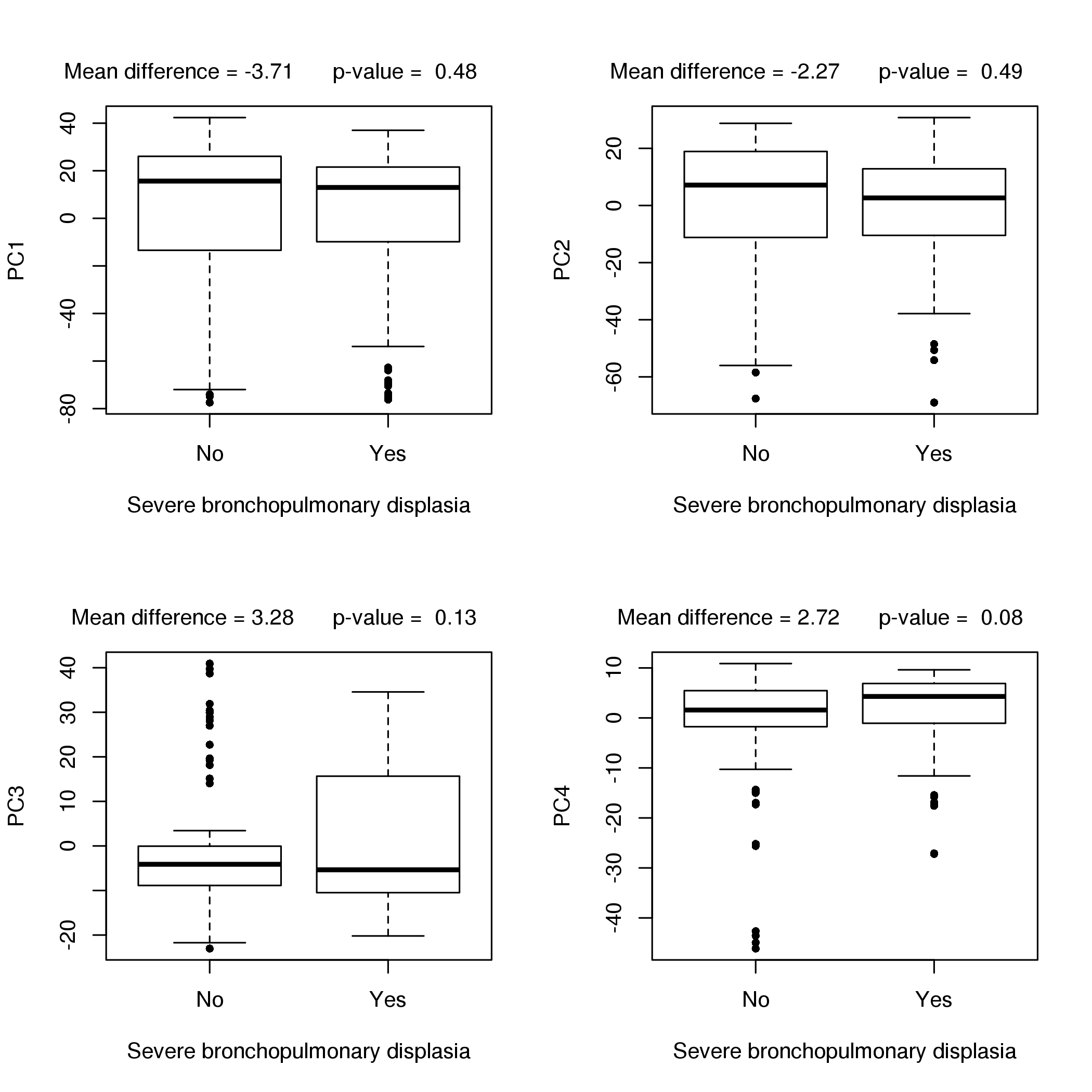

Supplement: Supplementary file 5 — Supplementary Figure S4. [file 41598_2021_84214_MOESM5_ESM.tiff]

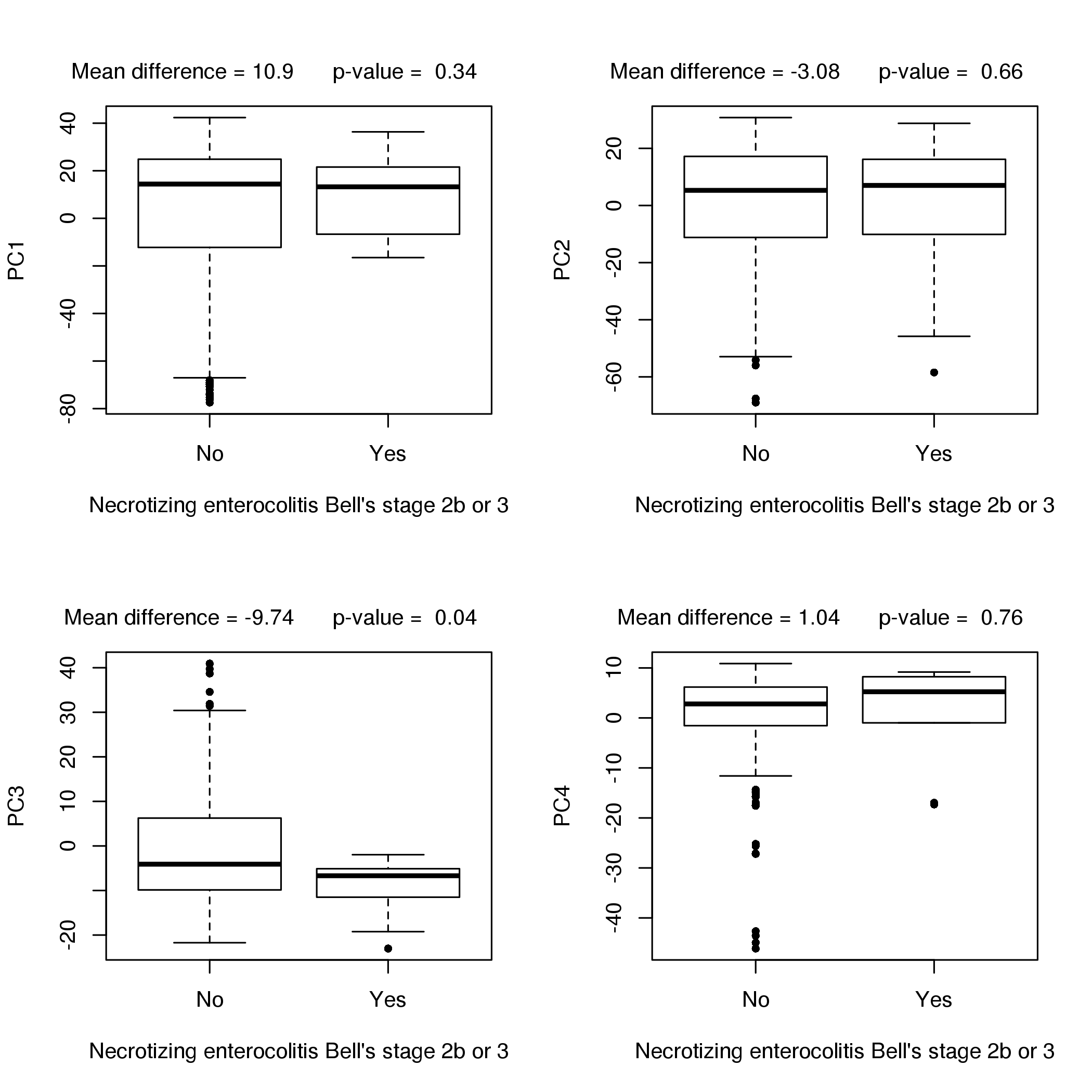

Supplement: Supplementary file 6 — Supplementary Figure S5. [file 41598_2021_84214_MOESM6_ESM.tiff]

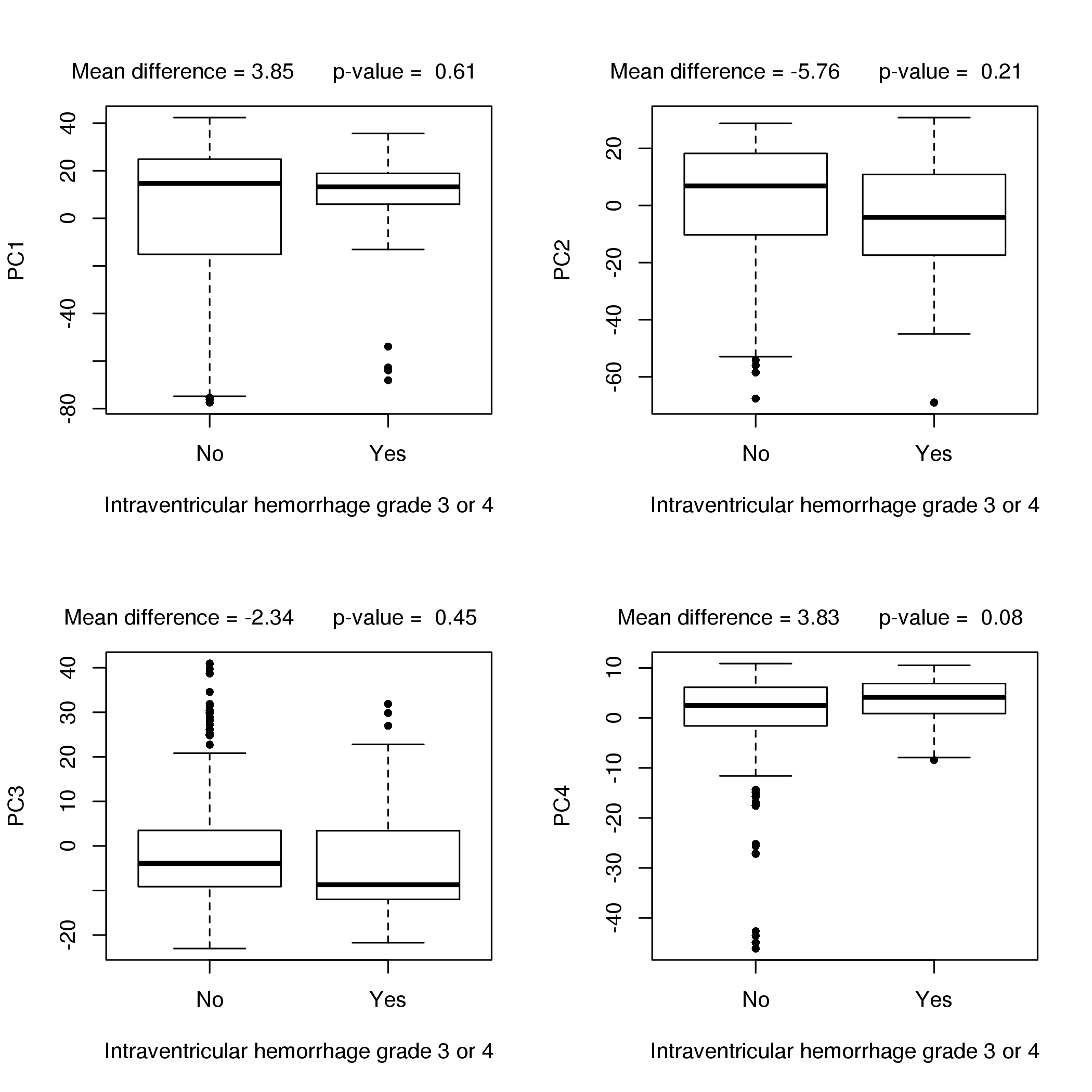

Supplement: Supplementary file 7 — Supplementary Figure S6. [file 41598_2021_84214_MOESM7_ESM.tiff]

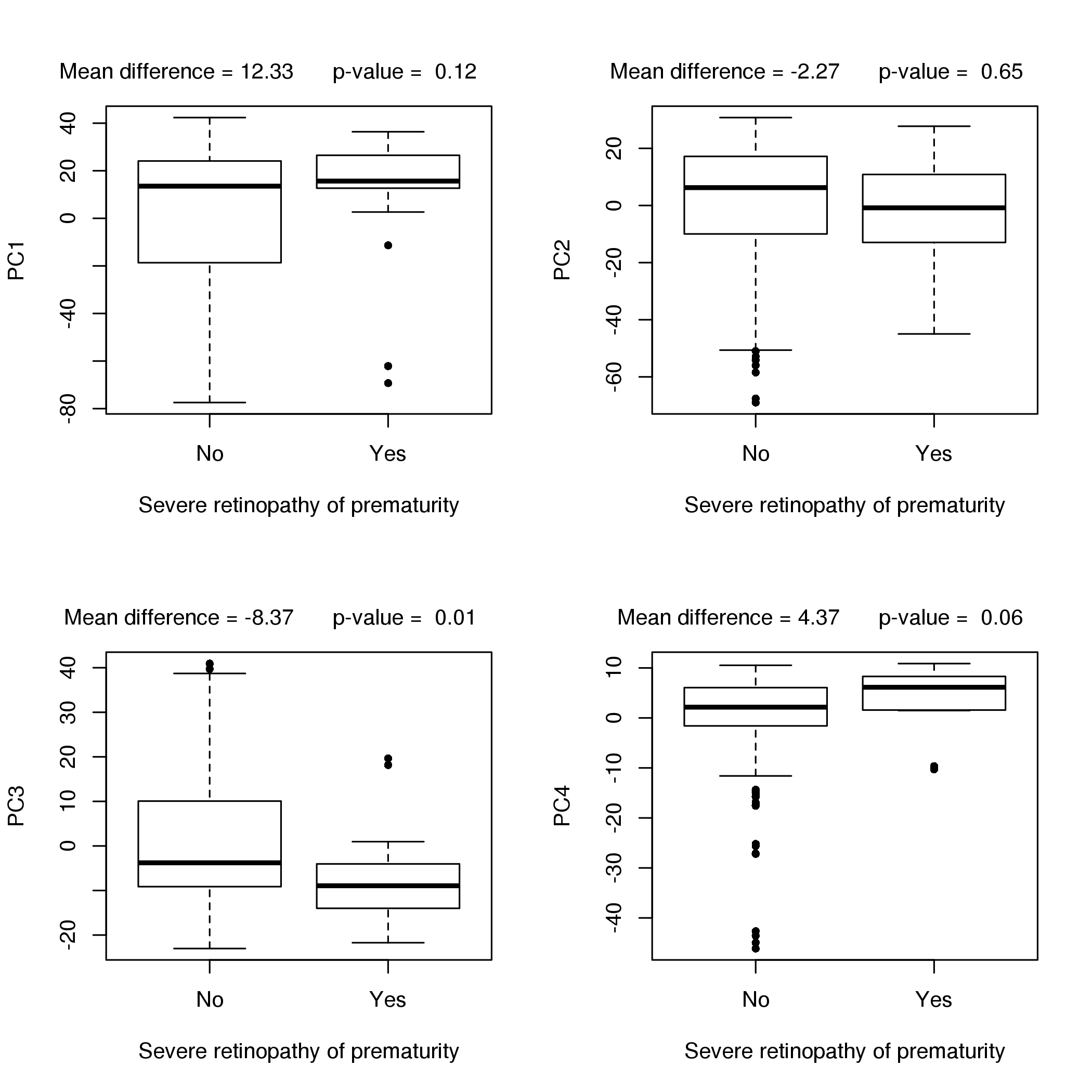

Supplement: Supplementary file 8 — Supplementary Figure S7. [file 41598_2021_84214_MOESM8_ESM.tiff]

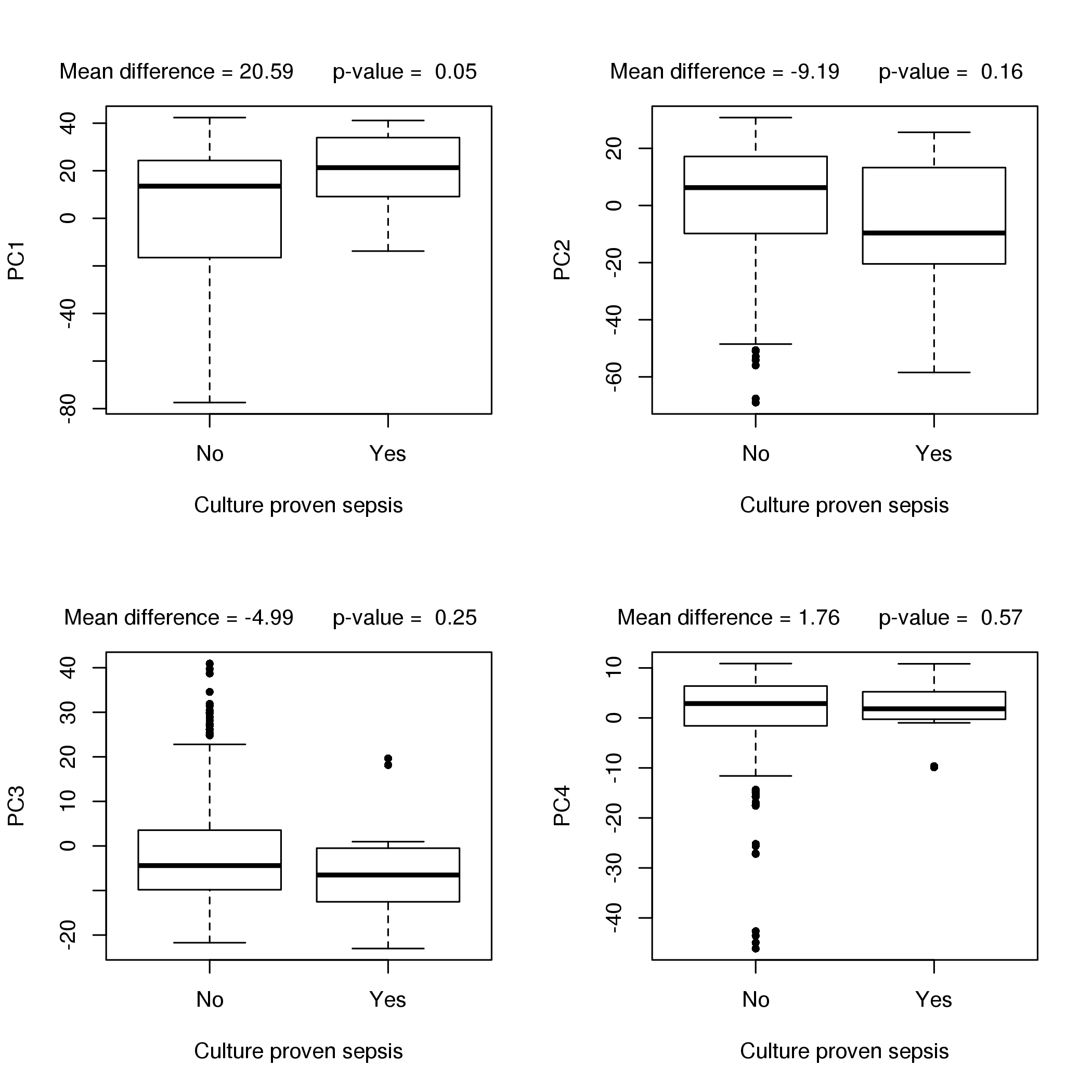

Supplement: Supplementary file 9 — Supplementary Figure S8. [file 41598_2021_84214_MOESM9_ESM.tiff]

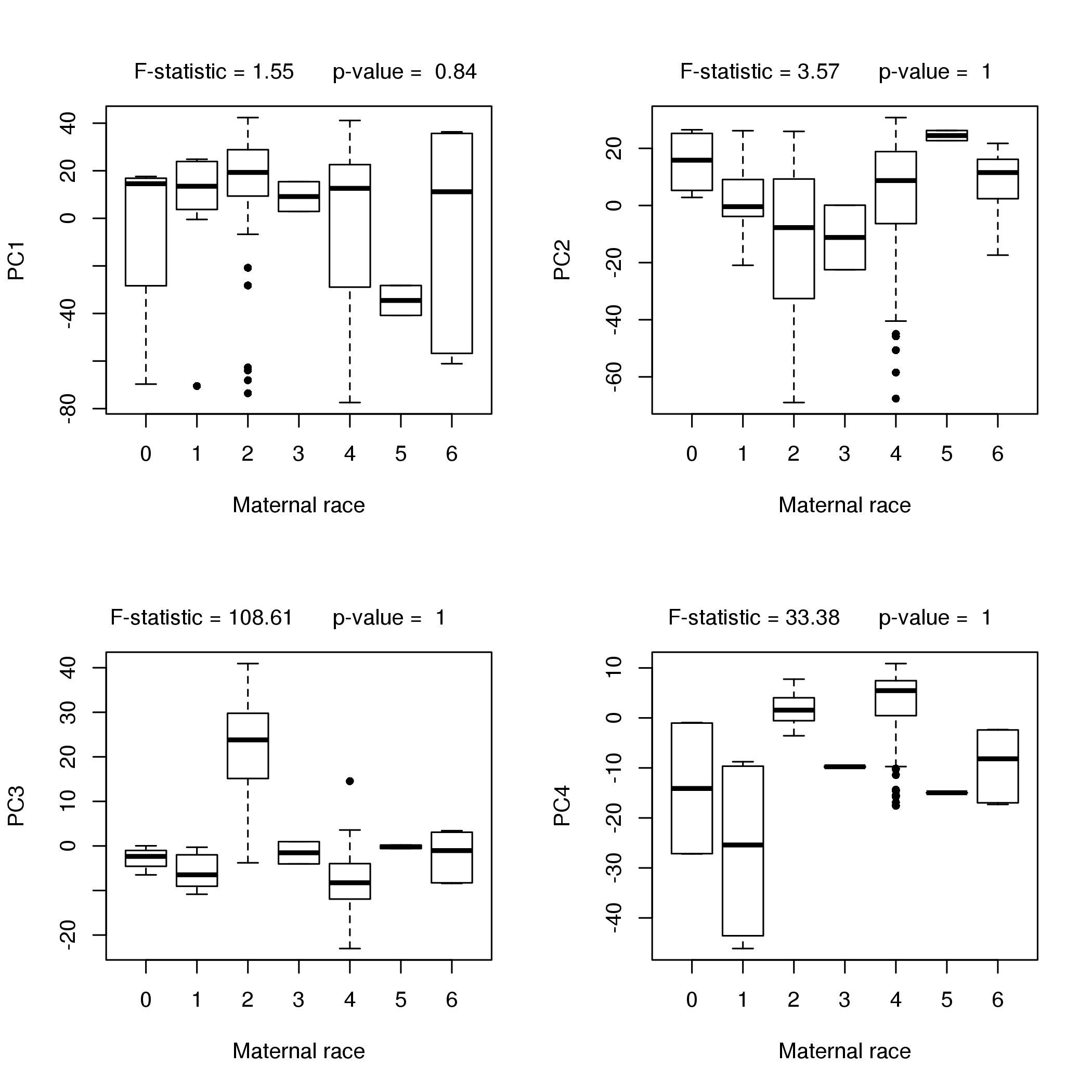

Supplement: Supplementary file 10 — Supplementary Figure S9. [file 41598_2021_84214_MOESM10_ESM.tiff]

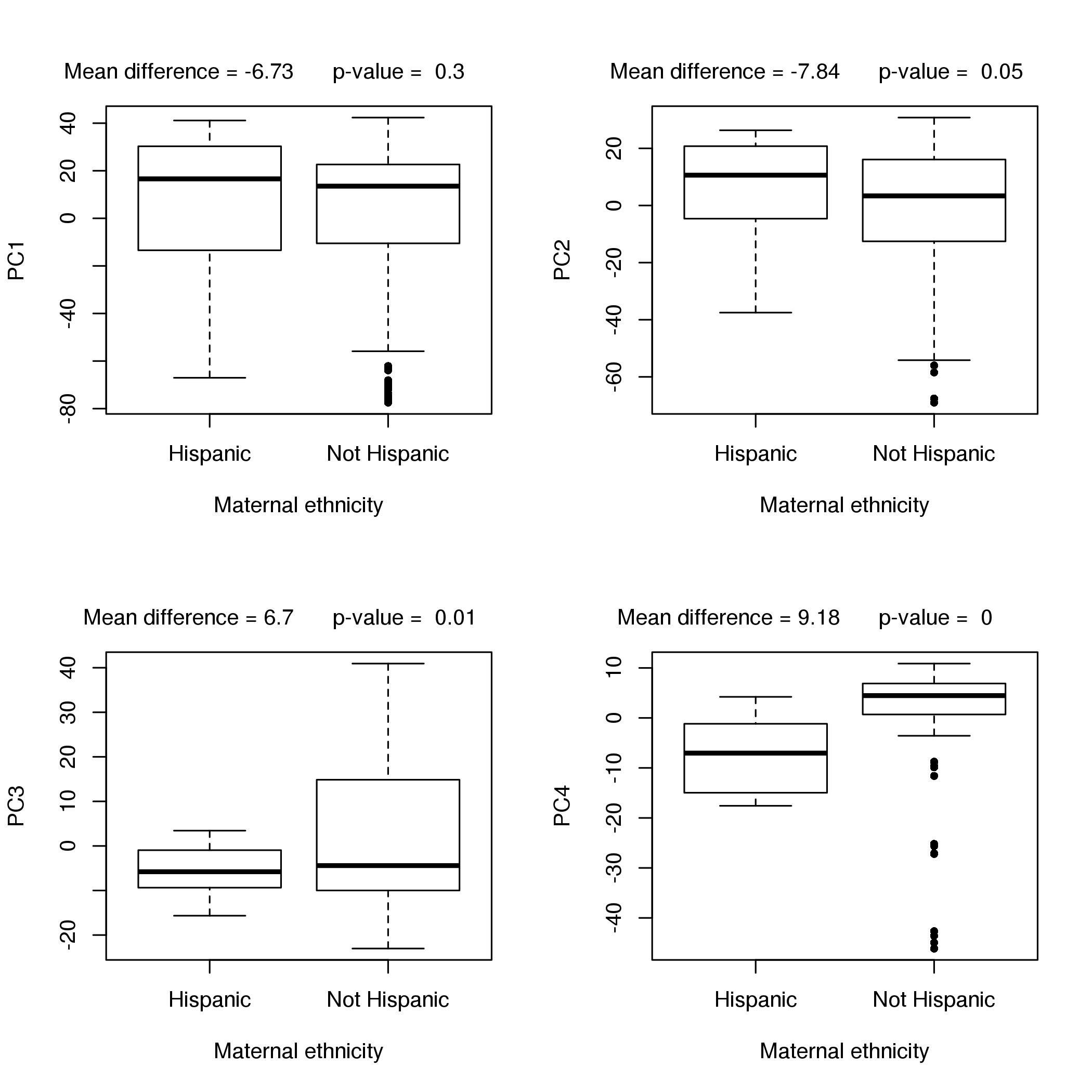

Supplement: Supplementary file 11 — Supplementary Figure S10. [file 41598_2021_84214_MOESM11_ESM.tiff]

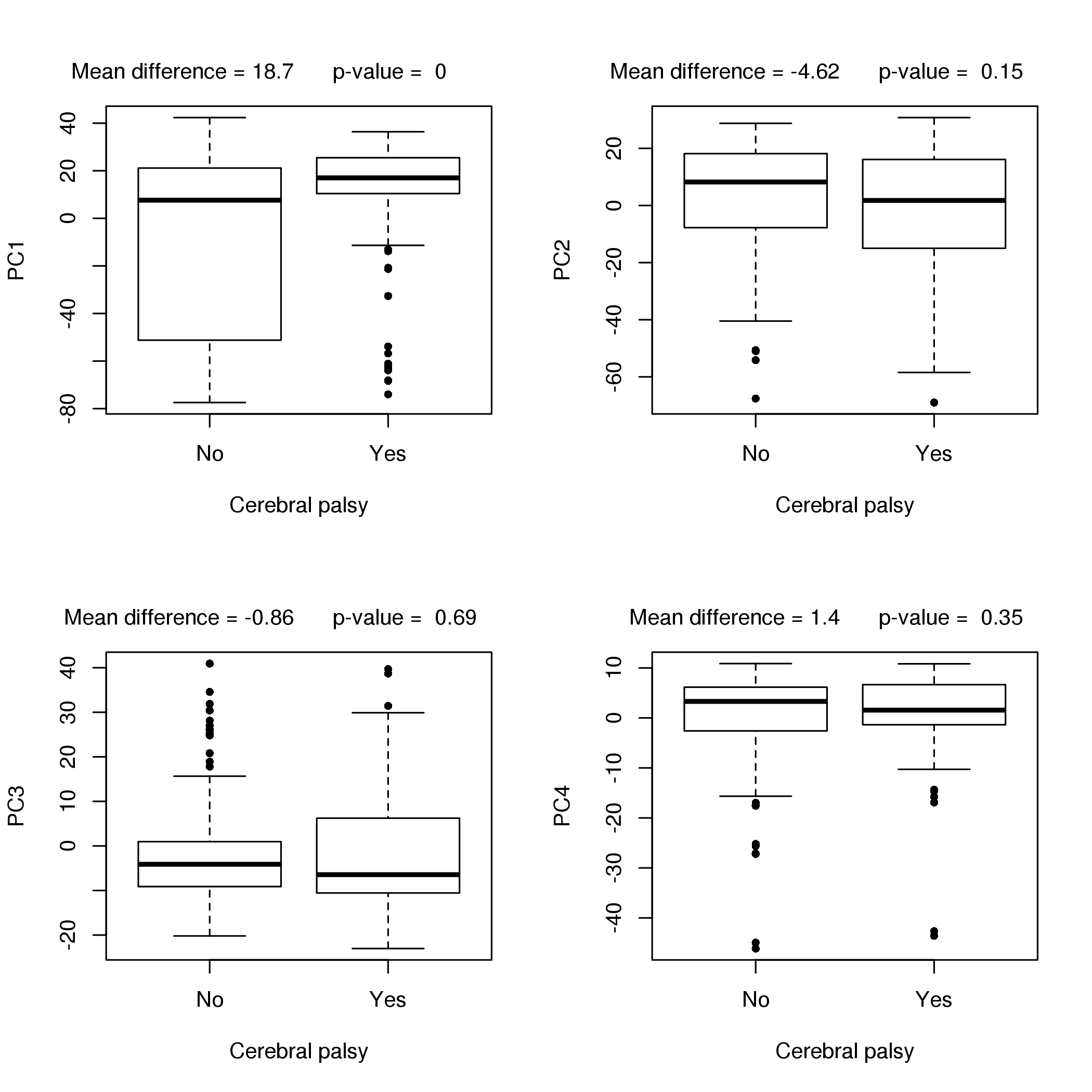

Supplement: Supplementary file 12 — Supplementary Figure S11. [file 41598_2021_84214_MOESM12_ESM.tiff]

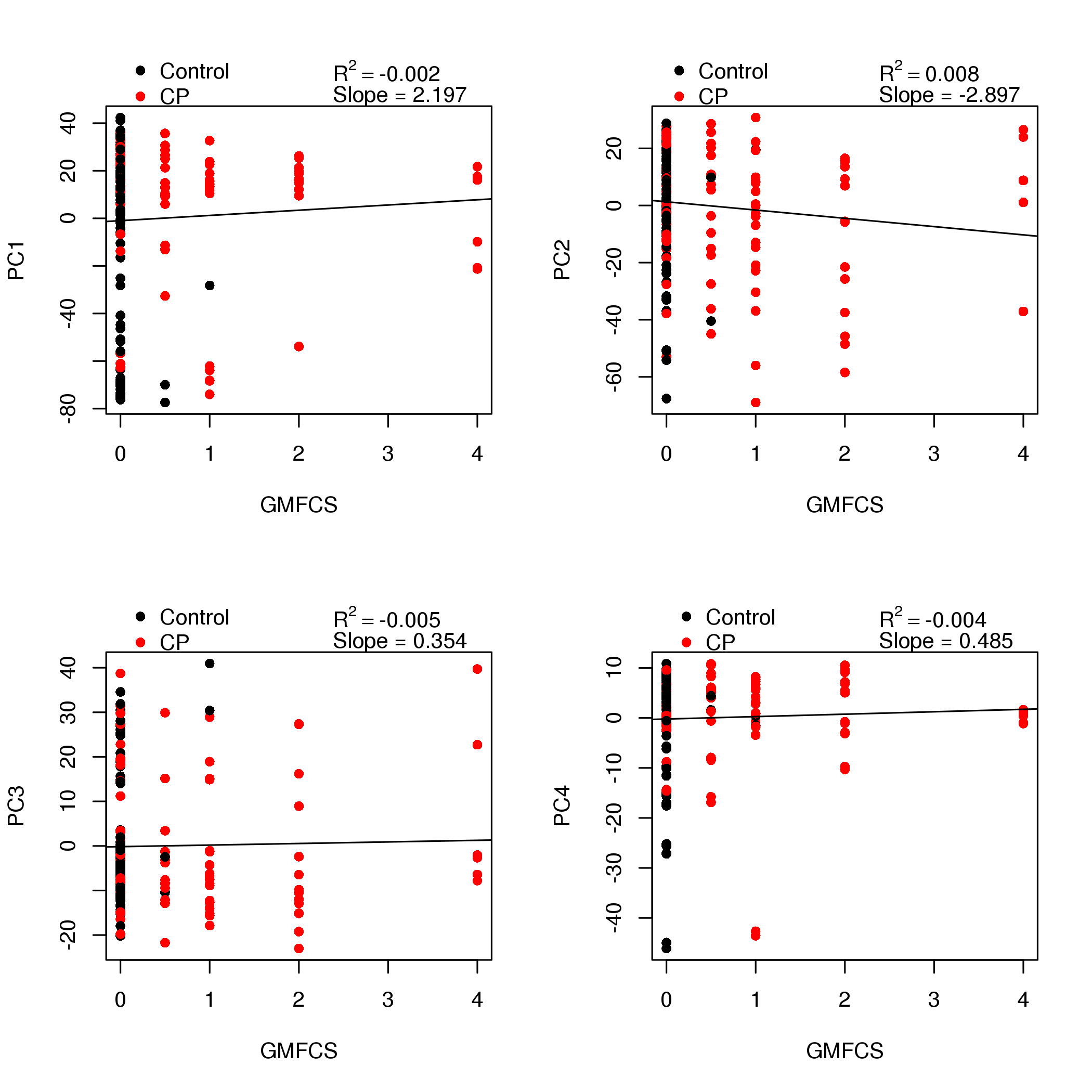

Supplement: Supplementary file 13 — Supplementary Figure S12. [file 41598_2021_84214_MOESM13_ESM.tiff]

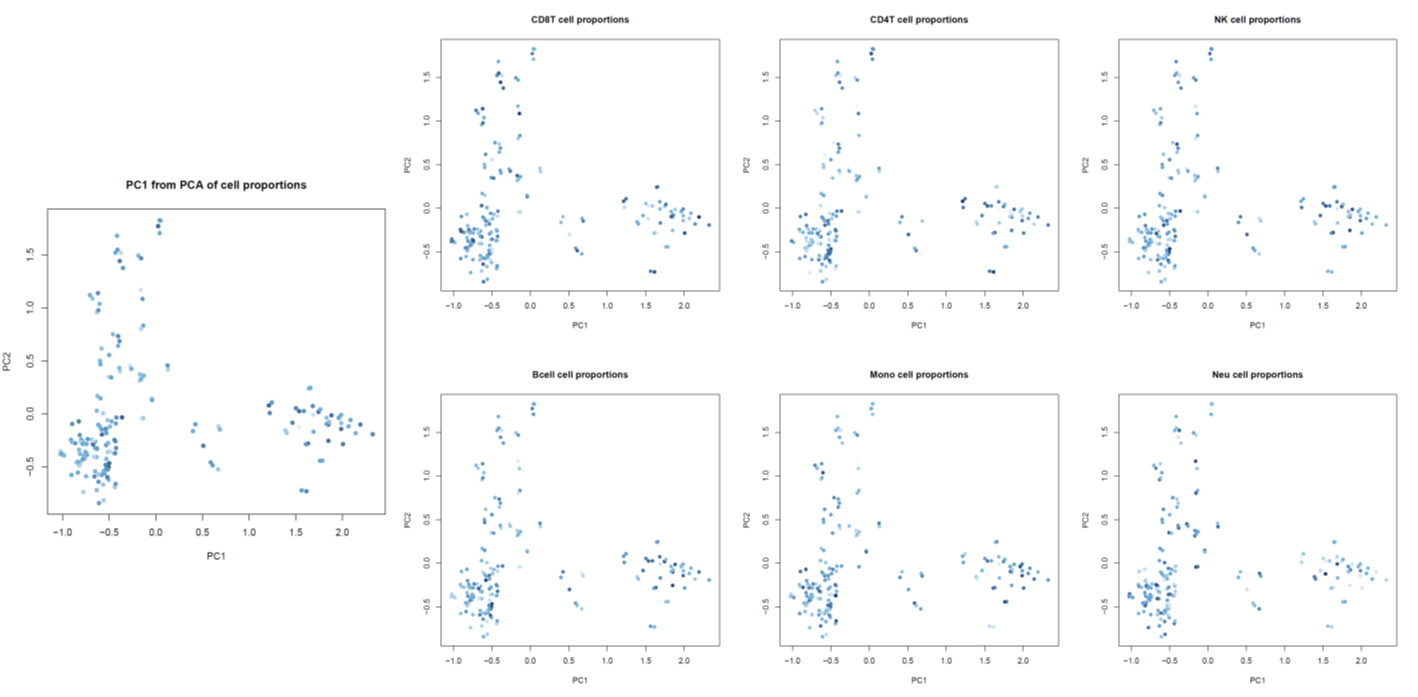

Supplement: Supplementary file 14 — Supplementary Figure S13. [file 41598_2021_84214_MOESM14_ESM.tif]
